# Construction of circRNA-Based ceRNA Network to Reveal the Role of circRNAs in the Progression and Prognosis of Hepatocellular Carcinoma

**DOI:** 10.3389/fgene.2021.626764

**Published:** 2021-02-26

**Authors:** Rong Deng, Xiaohan Cui, Yuxiang Dong, Yanqiu Tang, Xuewen Tao, Shuyu Wang, Jincheng Wang, Lin Chen

**Affiliations:** ^1^Department of General Surgery, Jiangsu Cancer Hospital & Jiangsu Institute of Cancer Research, The Affiliated Cancer Hospital of Nanjing Medical University, Nanjing, China; ^2^Department of General Surgery, The Affiliated Changzhou No. 2 People’s Hospital of Nanjing Medical University, Changzhou, China; ^3^Department of General Surgery, First Clinical Medical College, Nanjing Medical University, Nanjing, China; ^4^Department of Hepatobiliary Surgery of Drum Tower Clinical Medical College, Nanjing Medical University, Nanjing, China

**Keywords:** hepatocellular carcinoma, ceRNA, prognosis, circRNAs, bio-markers

## Abstract

**Background:**

Circular RNAs (circRNAs) are now under hot discussion as novel promising biomarkers for patients with hepatocellular carcinoma (HCC). The purpose of our study is to identify several competing endogenous RNA (ceRNA) networks related to the prognosis and progression of HCC and to further investigate the mechanism of their influence on tumor progression.

**Methods:**

First, we obtained gene expression data related to liver cancer from The Cancer Genome Atlas (TCGA) database (http://www.portal.gdc.cancer.gov/), including microRNA (miRNA) sequence, RNA sequence, and clinical information. A co-expression network was constructed through the Weighted Correlation Network Analysis (WGCNA) software package in R software. The differentially expressed messenger RNAs (DEmRNAs) in the key module were analyzed with the Database for Annotation Visualization and Integrated Discovery (DAVID) (https://david.ncifcrf.gov/summary.jsp) to perform functional enrichment analysis including Kyoto Encyclopedia of Genes and Genomes (KEGG) and Gene Ontology (GO). The data of miRNA expression and clinical information downloaded from TCGA were utilized for survival analysis to detach the prognostic value of the DEmiRNAs of the key module.

**Results:**

The 201 differentially expressed miRNAs (DEmiRNAs) and 3,783 DEmRNAs were preliminarily identified through differential expression analysis. The co-expression networks of DEmiRNAs and DEmRNAs were constructed with WGCNA. Further analysis confirmed four miRNAs in the most significant module (blue module) were associated with the overall survival (OS) of patients with liver cancer, including hsa-miR-92b-3p, hsa-miR-122-3p, hsa-miR-139-5p, and hsa-miR-7850-5p. DAVID was used for functional enrichment analysis of 286 co-expressed mRNAs. The GO analysis results showed that the top enriched GO terms were oxidation–reduction process, extracellular exosome, and iron ion binding. In KEGG pathway analysis, the top three enriched terms included metabolic pathways, fatty acid degradation, and valine, leucine, and isoleucine degradation. In addition, we intersected the miRNA–mRNA interaction prediction results with the differentially expressed and prognostic mRNAs. We found that hsa-miR-92b-3p can be related to CPEB3 and ACADL. By overlapping the data of predicted circRNAs by circBank and differentially expressed circRNAs of GSE94508, we screened has_circ_0077210 as the upstream regulatory molecule of hsa-miR-92b-3p. Hsa_circ_0077210/hsa-miR-92b-3p/cytoplasmic polyadenylation element binding protein-3 (CPEB3) and acyl-Coenzyme A dehydrogenase, long chain (ACADL) were validated in HCC tissue.

**Conclusion:**

Our research provides a mechanistic elucidation of the unknown ceRNA regulatory network in HCC. Hsa_circ_0077210 might serve a momentous therapeutic role to restrain the occurrence and development of HCC.

## Introduction

Liver cancer is a common malignant tumor worldwide and ranks second among cancer-related deaths (Torre et al., 2015). Although great efforts had been made in the prevention and treatment of hepatocellular carcinoma (HCC), the incidence and mortality of HCC are still on the rise (Siegel et al., 2017). The timely diagnosis of liver cancer is of great significance for improving the survival time of patients. For patients with Barcelona Clinic Liver Cancer (BCLC) stage 0 or A, the 5-year survival rate after surgery is 90 and 50–70%, respectively (Llovet et al., 1999; Cillo et al., 2006; Forner et al., 2010). Unfortunately, patients with BCLC staging of B, C, or D will not have a survival rate higher than 16% at 5 years after surgery (Forner, 2015). Based on the current status of diagnosis and treatment of HCC, it is very urgent to find effective molecular targets for HCC.

In fact, protein-coding genes account for less than 2% of the entire genome (Tay et al., 2014). A large amount of non-coding RNA (ncRNA), for a long time, was considered “transcriptional noise” with little function. However, evidences showed that ncRNAs, including microRNAs (miRNAs), long-chain non-coding RNAs (lncRNA), and circular RNAs (circRNAs) may play crucial roles in HCC (Giordano and Columbano, 2013; Klingenberg et al., 2017; Wang et al., 2018; Pea et al., 2020). Tay et al., 2014 put forward the hypothesis of competing endogenous RNA (ceRNA), which suggested that messenger RNA (mRNA), pseudogenes, and ncRNA are “crosstalked” through common miRNA response elements (MRE). Moreover, this hypothesis provided a theoretical basis for mRNA, miRNA, and circRNA to construct a regulatory network.

In the previous studies, circRNAs were considered as by-products of abnormal splicing and had little potential to work in physiological or pathological processes (Memczak et al., 2013). However, the role of circRNAs under physiological and pathological conditions has been partially explored (Li et al., 2018). Recent studies have shown that the synthesis of circRNAs depends on the spliceosome mechanism and was regulated by *cis-*complementary sequences and protein factors (Li et al., 2018). CircRNAs are known to be involved in the isolation of miRNAs or proteins, regulation of transcription and splicing, and translation of peptides (Li et al., 2018). Abovementioned functions are still a little part of the functions of circRNA, and there are a large number of functions related to circRNA that need to be explored.

In this study, we screened differentially expressed miRNAs (DEmiRNAs) and mRNAs (DEmRNAs) associated with survival through Weighted Correlation Network Analysis (WGCNA). In addition, we described the expression and lineage of circRNAs in HCC and investigated the mechanisms of ceRNA network in the development and progression of HCC.

## Materials and Methods

### Data Acquisition and Differential Expression Screening

First, we obtained gene expression data related to HCC from TCGA database(see text footnote 1), including miRNA-seq, RNA-seq, and corresponding clinical information. Using the edgeR R software package, we analyzed the expression data with clinical information and deleted duplicate data and obtained DEmRNA and DEmiRNA. |Log 2-fold change| ≥ 1.0 and *P* value < 0.05 were used as the criteria to get mRNAs and miRNAs for further analysis. The data obtained from TCGA in the study are publicly available, and the approval of the local ethics committee is not necessary. Then, we searched HCC-related circRNA expression microarray information from the Gene Expression Omnibus (GEO) database^1^. GSE94508 (data from five primary HCCs and five matched normal liver tissues) was utilized for further analysis. The Limma software package (version: 3.40.2) can be used to study the differential expression of circRNA [differentially expressed circRNA (DEcircRNA)]. Since the data types of miRNA-seq and RNA-seq were count profiles, we used edgeR software package to select DEmRNA and DEmiRNA. In addition, because the data types of circRNA expression were non-counted, we used Limma software package to select DEcircRNA. We set the threshold for circRNA differential expression screening to |Log 2-fold change| ≥ 1.0 and *P* value < 0.05. Probe sets with circbase ID^2^ will be included in the research, while probe sets without circbase ID will be deleted.

### WGCNA and Identification of the Liver Cancer Carcinogenesis Modules

A co-expression network was constructed using the WGCNA software package in R software, with the purpose of identifying important miRNAs and mRNAs related to liver cancer. The GoodSamplesGenes function can be used to check whether the DEmiRNA and DEmRNA of the data matrix meet the standard. We eliminated mRNA and miRNA data with missing values. According to this standard, we will exclude unqualified data. Then, in order to ensure a scale-free network, we use the pickSoft-Threshold function to calculate the β value (soft threshold power parameter). Next, the diagram of the tree was established by hierarchical clustering, and the correlation between module eigengenes (MEs) and clinical trait was calculated and used to filter out the MEs unrelated to the carcinogenesis and progression of HCC. Last, a core-module from all modules based on the highest correlation coefficient was used for further research.

### Functional Enrichment Analysis

The DEmRNAs in the key module were analyzed with Database for Annotation Visualization and Integrated Discovery (DAVID) (see text footnote 2) to perform functional enrichment analysis including Kyoto Encyclopedia of Genes and Genomes (KEGG) and Gene Ontology (GO). The pathways that had *p* < 0.05 were considered significantly enriched.

### Survival Analysis of the miRNAs in the Key Module and Functional Pathway Enriched

We utilized the count matrix of miRNA and clinical information downloaded from TCGA for survival analysis to detach the prognostic value of the DEmiRNAs of the key module. We screened out DEmiRNAs under the condition of the log-rank *P* value < 0.05 and drew survival curves of DEmiRNAs that were significant through the R package “Survminer.” The miRPath V3.0 tool was used to enrich pathways of prognosis-related DEmiRNAs.

### Prediction of miRNA–mRNA and circRNA–miRNA Interactions

We selected the first two key modules in WGCNA-DEmiRNAs for survival analysis, and DEmRNAs with a log-rank *P* value < 0.05 were considered potential DEmRNAs. Then, we used the online analysis tools DIANA^3^ and TargetScan^4^ to predict target mRNAs of potential DEmiRNAs. Target genes appearing in both databases were considered to be the predicted results of potential DEmiRNA target genes. Target mRNA that overlaps with potential DEmRNAs was considered potential mRNA and requires further analysis. We set two screening conditions (binding sites ≥ 3 and length < 2,000) and used the circBank database^5^ to predict potential DEmiRNA target circRNAs. We selected the overlapping part of the target circRNAs from GSE94508 and DEcircRNAs as potential circRNAs to improve the reliability of the analysis. In addition, we retrieved the target circRNA acting on the corresponding DEmiRNA from the circBank database. The part of the target gene that overlapped with DEcircRNAs with mRNA regulatory potential was defined as the key gene. The structure diagram of potential circRNA was detected and downloaded through the online website Cancer-Specific CircRNA Database (CSCD)^6^.

### Construction of circRNA–miRNA–mRNA Network

This regulatory network consisted of downstream regulatory genes of miRNA and circRNA that had potentially regulated miRNA. Then, we used Cytoscape software (version 3.7.2) to construct a visualized circRNA–miRNA–mRNA network.

### Survival Analysis of the Key Genes and Gene Set Enrichment Analysis

The survival analysis of key genes was constructed by the Kaplan–Meier plotter. Based on the known information of gene characteristics, location, and biological functions, we established a database of molecular characteristics by Gene Set Enrichment Analysis (GSEA). We set the cutoff value to *p* < 0.05 and detected the pathways that were significantly related to the key genes with GSEA analysis.

### RNA Extraction, Reverse Transcription Quantitative PCR, and Immunohistochemical Staining

A total of 15 paired fresh-frozen HCC tissues and normal tissues were obtained from patients diagnosed with Hepatocellular Carcinoma (HCC) at The Affiliated Cancer Hospital of Nanjing Medical University, Jiangsu Cancer Hospital and Jiangsu Institute of Cancer Research. Total cellular RNA from tissues was collected with TRIzol Reagent (Invitrogen). cDNA was reversed from total RNA with PrimeScript^TM^ RT reagent kit (Takara Bio, Inc., Otsu, Japan). Real-time quantitative PCR was utilized to assess the circ_RNA expression, which was carried out in triplicate by an SYBR Premix Ex Taq^TM^ kit (Takara Bio) and ABI 7900HT Real-Time PCR system (Applied Biosystems Life Technologies, Foster City, CA, United States). The final results were analyzed through the comparative cycle threshold values (2^–ΔΔCt^). Paraffin-embedded slices were utilized to conduct immunohistochemical (IHC) staining. First, 5% bovine serum albumin (BSA) were used to block the slices, and then slices were reacted with anti-CPEB3 and anti-ACADL rabbit polyclonal antibody, respectively (1:1,000, Abcam, United Kingdom), at 4°C overnight. Next, the slices were incubated with horseradish peroxidase (HRP)-conjugated rabbit secondary antibody for 120 min at room temperature.

### Statistical Analysis

The results are expressed in the form of mean ± standard deviation (SD). The statistical analysis of the data was performed using the non-parametric tests of SPSS v23.0 software (SPSS Inc., Chicago, IL, United States). To compare two independent groups, we utilized Mann–Whitney *U* test. *P* values < 0.05 of differences were considered statistically significant.

## Results

### Differential Expression Screening of miRNA and mRNA in TCGA-LIHC Database

**FIGURE 1:**
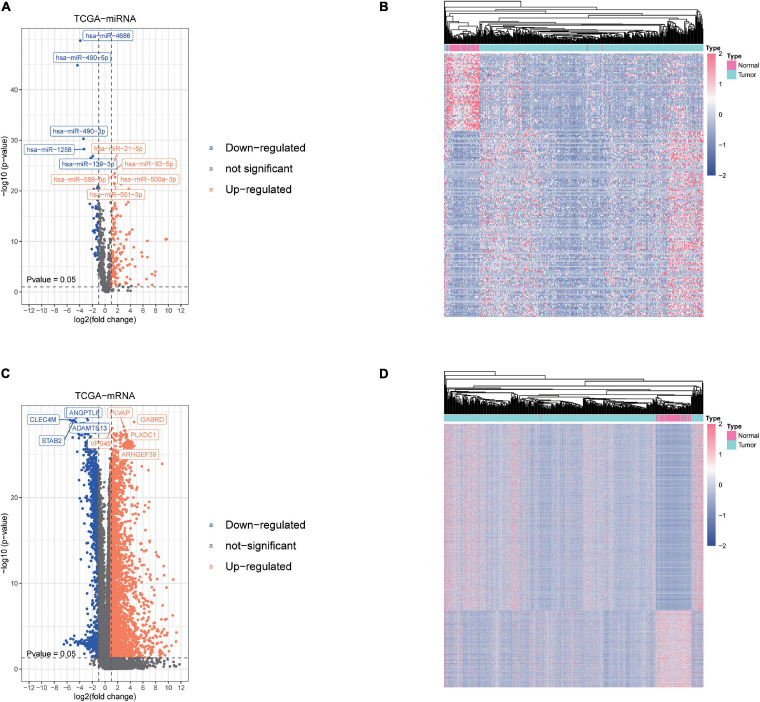
Screening of differentially expressed miRNAs and mRNAs. **(A,B)** The differential expression and heatmap of miRNAs in hepatocellular carcinoma (HCC) tissue compared with normal liver tissue. **(C,D)** The differential expression and heatmap of mRNAs in HCC tissue compared with normal liver tissue.

We obtained the transcriptome data and clinical information from The Cancer Genome Atlas Liver Hepatocellular Carcinoma (TCGA-LIHC) dataset, including 425 miRNA specimens (50 normal samples and 375 liver cancer samples) and 424 mRNA specimens (50 normal samples and 374 liver cancer samples). Then, DEmiRNAs and DEmRNAs were finally identified. The cutoff threshold of screening was set to |Log 2-fold change| ≥ 1.0 and *P* value < 0.05, which revealed 201 and 3,783 aberrantly expressed miRNAs and mRNAs, respectively (Supplementary Tables 1, 2). As shown in Volcano map and Heatmap, the 201 DEmiRNAs included 142 upregulated and 59 downregulated miRNAs (Figures 1A,B), while the 3,783 DEmRNAs included 2,677 upregulated and 1,106 downregulated mRNAs (Figures 1C,D).

### WGCNA and Identification of Liver Cancer Tumorigenesis Modules

**FIGURE 2:**
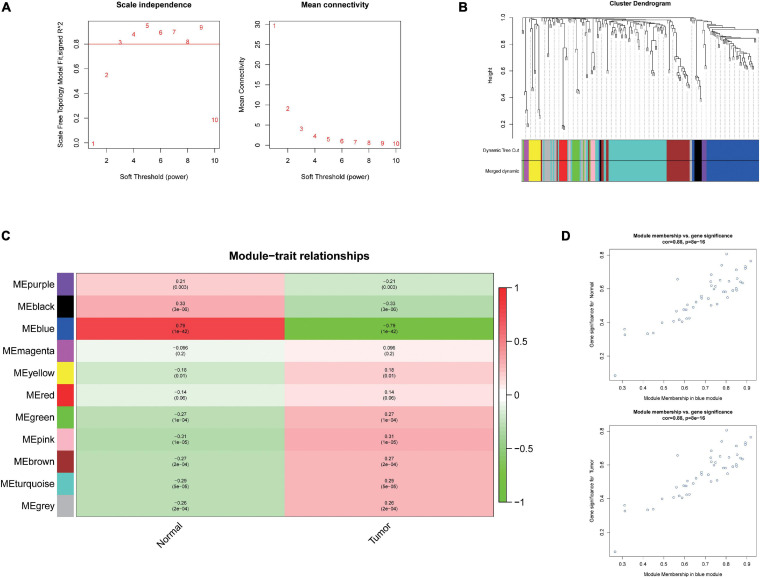
Weighted Correlation Network Analysis (WGCNA) analysis and identification of liver cancer tumorigenesis modules in miRNA expression profile. **(A)** Scale-free networks of scale independence and mean connectivity. **(B)** miRNA co-expression network modules. The miRNAs were organized into 11 co-expression network modules. Different colors represent different modules, and gray represents miRNAs that cannot be merged into any module. **(C)** Correlation between miRNA MEs disease characteristics. Each row corresponds to an ME, each column to a trait. Each cell contains the corresponding correlation and the *P* value. The table is color-coded by the correlation according to the color legend. **(D)** Scatter plots of gene significance (GS) vs. module membership (MM) in the blue modules using linear regression.

**FIGURE 3:**
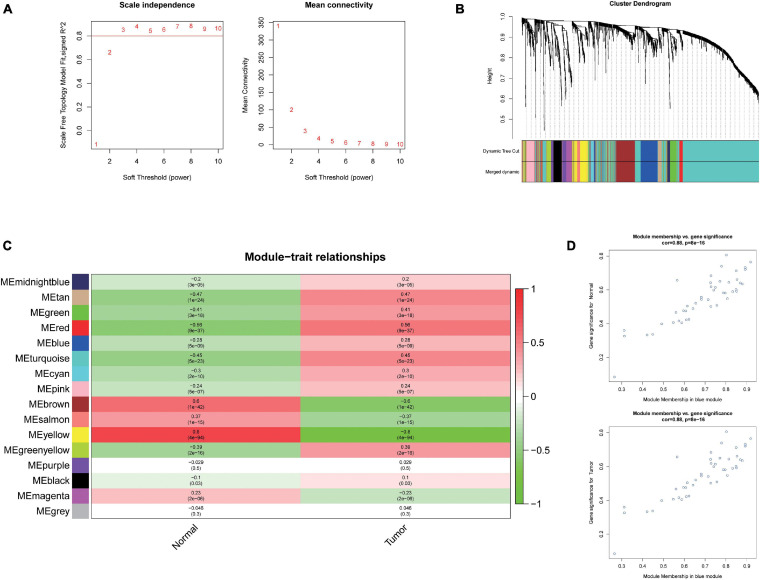
Weighted Correlation Network Analysis (WGCNA) and identification of liver cancer tumorigenesis modules in mRNA expression profile. **(A)** scale-free networks of scale independence and mean connectivity. **(B)** mRNA co-expression network modules. The mRNAs were organized into 16 co-expression network modules. Different colors represent different modules, and gray represents mRNAs that cannot be merged into any module. **(C)** Correlation between mRNA module eigengene (ME) disease characteristics. Each row corresponds to an ME, each column to a trait. Each cell contains the corresponding correlation and the *P* value. The table is color-coded by the correlation according to the color legend. **(D)** Scatter plots of gene significance (GS) vs. module membership (MM) in the yellow modules using linear regression.

To further investigate the carcinogenesis of DEmiRNAs and DEmRNAs, WGCNA package was utilized. Based on the value of soft threshold power β (β = 0.8), the connection between the genes in the gene network was found to be consistent with the results of scale-free network distribution. Next, the modules of DEmiRNA and DEmRNA were constructed co-expression networks by using WGCNA. Then, the modules with similar expression profiles were identified by the dynamic tree-cut diagram (Figures 2A,B, 3A,B). After analyzing and classifying, 11 modules for DEmiRNAs and 16 modules for DEmRNAs were identified. In view of the heatmap of module–trait relationships (Figure 2C), we could find that the blue module of DEmiRNAs was the highest positive correlation with tumor tissues (correlation coefficient = −0.79, *P* = 1e-42). In addition, the yellow modules of DEmRNAs showed the most significant positive correlation with tumor tissues (correlation coefficient = −8, *P* = 4e-94; Figure 3C). According to the miRNA and mRNA data of the most significant module among the constructed modules (Supplementary Tables 3, 4, 5), the correlations between gene significance for normal or tumor and module membership in blue and yellow were verified, respectively (Figures 2D,E, 3D,E).

### Survival Analysis and Pathway Analysis of the miRNA in the Most Significant Module

**FIGURE 4:**
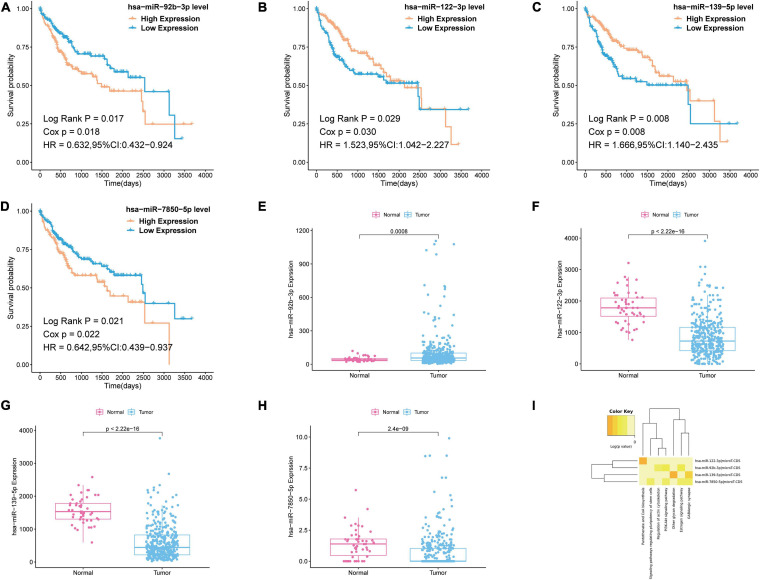
Survival analysis and pathway analysis of the miRNA in the most significant module. **(A–D)** Survival analysis of the miRNA in the most significant module. **(E–H)** Boxplots of expression level of the miRNA in the most significant module in the normal group and tumor group. **(I)** Pathway analysis of the miRNA in the most significant module by using DIANA-miRpath v.3.

To further reveal which of the DEmiRNAs were the most prognostic miRNA, we analyzed the association between the expression of all miRNAs in the most significant module (blue module) and overall survival (OS) of patients with liver cancer. Then, we found four miRNAs associated with the OS of patients with liver cancer, including hsa-miR-92b-3p, hsa-miR-122-3p, hsa-miR-139-5p, and hsa-miR-7850-5p (Figures 4A–D). As shown in Figures 4E–H, hsa-miR-92b-3p was upregulated in the tumor tissues; hsa-miR-122-3p, hsa-miR-139-5p, and hsa-miR-7850-5p were downregluated in the tumor tissues. Next, we established OS curves and found the high expression of hsa-miR-92b-3p and hsa-miR-7850-5p and the low expression of hsa-miR-122-3p and hsa-miR-139-5p had poor survival prognosis (Figures 4A–D). KEGG pathway analysis based on the four miRNAs was carried out using DIANA-miRpath v.3, a web tool. The results revealed that all miRNAs were related to tumor-associated pathways, especially phosphoinositide 3-kinase (PI3K)-Akt signaling pathway, pantothenate CoA biosynthesis, and signaling pathways regulating pluripotency of stem cells (Figure 4I).

### Functional Enrichment Analysis of the Co-expressed mRNAs in the Key Modules

In order to fully evaluate the contribution of DEmRNAs in the key modules (yellow and brown modules) to the development and progression of liver cancer, DAVID was used for functional enrichment analysis of 286 co-expressed mRNAs. The GO analysis results showed that the top three enriched biological processes were oxidation–reduction process, metabolic process, and lipid metabolic process (Supplementary Figure 1A); the top three enriched cell components were extracellular exosome, extracellular region, and extracellular space (Supplementary Figure 1A); the top three enriched molecular functions were iron ion binding, heme binding, and electron carrier activity (Supplementary Figure 1A). In KEGG pathway analysis, the top three enriched terms included metabolic pathways, fatty acid degradation, and valine, leucine, and isoleucine degradation (Supplementary Figure 1B).

### Screen of miRNA–mRNA Interactions and Pathway Analysis of Target mRNAs

**FIGURE 5:**
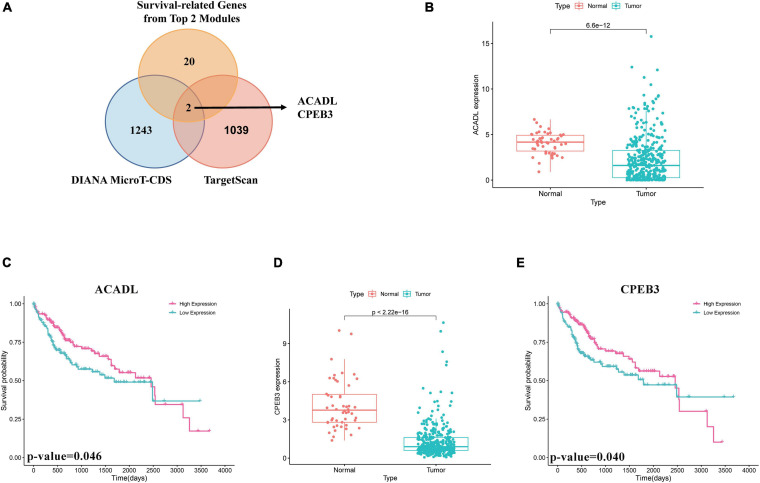
Screen of miRNA–mRNA interactions and target mRNAs. **(A)** The intersection result between the target predicted by DIANA and TargetScan of the four differentially expressed and prognostic miRNAs and the overall survival result of mRNAs in the top two significant modules in WGCNA-mRNA. **(B)** Boxplots of expression level of ACADL in the normal group and the tumor group. **(C)** Survival analysis of ACADL. **(D)** Boxplots of expression level of CPEB3 in the normal group and the tumor group. **(E)** Survival analysis of CPEB3.

**FIGURE 6:**
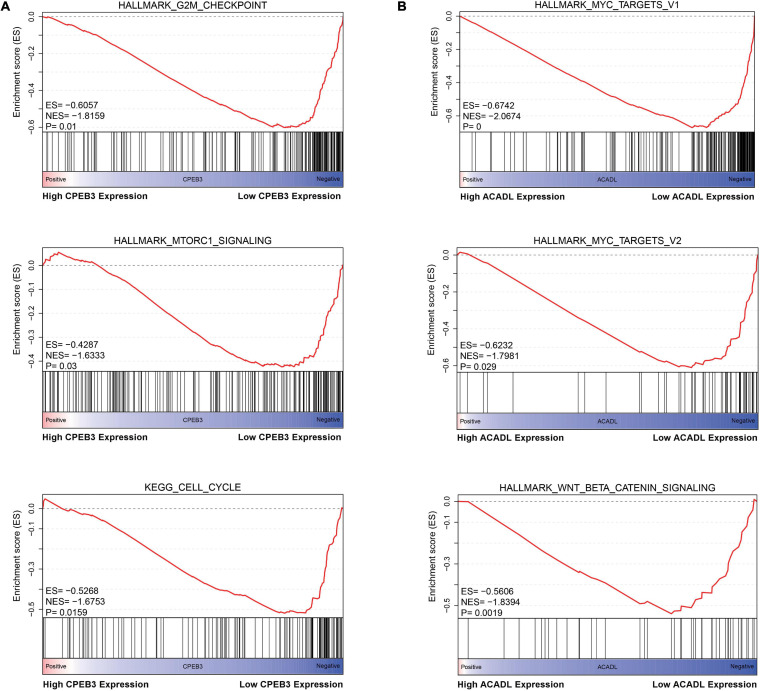
Identification the biological functions of ACADL and CPEB3. **(A)** The result of Gene Set Enrichment Analysis of ACADL indicated that G2M/CHECKPOINT, MTORC1/SIGNALING, and CELL/CYCLE pathways can be actively regulated by CPEB3. **(B)** The result of Gene Set Enrichment Analysis of ACADL indicated that MYC/TARGET/V1, MYC/TARGET/V2, and WNT/BETA/CATENIN/SIGNALING pathways can be actively regulated by ACADL.

First, we performed OS analysis and plotted survival curves of the top two significant modules in WGCNA-mRNA (Supplementary Figure 2 and Table 1). Second, Targetscan and DIANA were utilized to predict the target mRNAs of the four differentially expressed and prognostic miRNAs, which might regulate the downstream pathway. Then, we crossed the prediction results with the differentially expressed and prognostic mRNAs and found that hsa-miR-92b-3p can be related to CPEB3 and ACADL (Figure 5A). Base on the clinical information, the high expression of CEPE3 and ACADL predicted better OS (Figures 5C,F). The expressions of CPEB3 and ACADL were downregulated in tumor tissues (Figures 5B,D). The result was consistent with the expression trend. In fact, the low expression of CPEB3 and ACADL could be associated with higher tumor grade but could not be associated with gender, tumor stage, or TNM (Supplementary Figure 3). Moreover, to understand the functional pathway of CPEB3 and ACADL, GSEA was performed. Three pathways including G2M checkpoint, mtorc1 signaling, and cell cycle showed a positive correlation with low CPEB3 expression (Figure 6A). Meanwhile, the GSEA of ACADL showed the three most significant enriched pathways, including myc targets v1, myc targets v2, and wnt beta catenin signaling (Figure 6B). In addition, we used web tool TIMER to analyze the correlation between expression levels of CPEB3 and ACADL and immune cells. The results confirmed that CPEB3 and ACADL were significantly related to tumor immunity: CPEB3 was related to the content of B cells and neutrophil; ACADL was related to the infiltration of B cells, CD8^+^ T cells, CD4^+^ T cells, macrophages, and dendritic cells (Supplementary Figures 4A,B).

**TABLE 1:** 22 survival-related genes in brown and yellow module.

Gene	KM (*P*-value)
N4BP2L1	0.0231917680013911
C6	0.0324979505945558
DMGDH	0.00345549731847261
ACADL	0.0458976763753126
CYP7A1	0.015599256837466
MANF	0.0354210475561331
SARDH	0.0458207466346884
TUBE1	0.0171553602956993
ANXA10	0.0281889814075095
SLC38A4	0.0209045132009409
C8B	0.0298561967458564
LPA	0.0172195797768141
MAT1A	0.0461625356948938
ADH4	0.00572238509386069
SLC10A1	0.025877998862526
FNIP2	0.0474647059410264
CYP4F2	0.0298914997690577
TTC36	0.0224862889024342
ADRA1A	0.0466515861358356
GYS2	0.0334809963912731
ABAT	0.0259103935446722
CPEB3	0.0403726764983748

### Exploring the circRNA That Regulates hsa-miR-92b-3p

**FIGURE 7:**
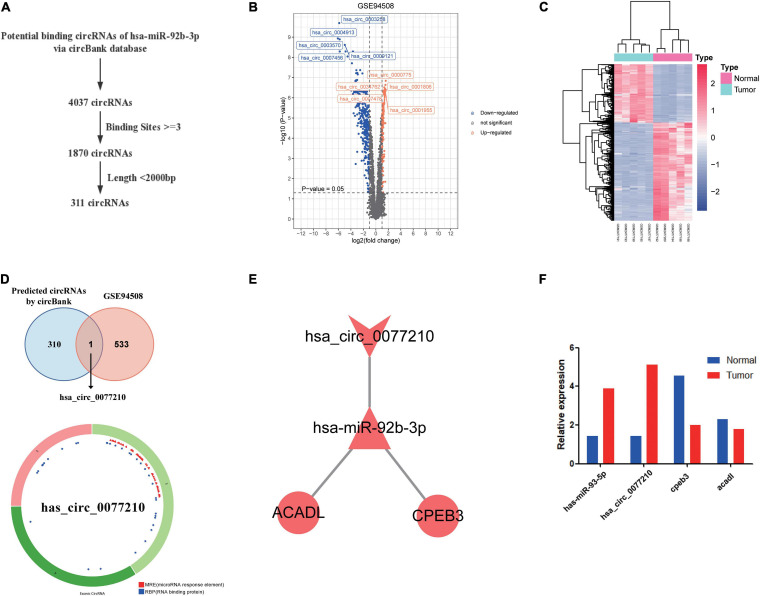
Construction of ceRNA regulatory axis. **(A)** Flowchart of the ceRNA regulatory axis. **(B)** Volcano plot of differentially expressed circRNAs in GSE94508. **(C)** The heatmap of differentially expressed circRNAs in GSE94508. **(D)** The intersection result between the target predicted by circBank database and the differentially expressed circRNAs in GSE94508. **(E)** Flowchart of the chosen circRNAs by using circBank database. **(F)** Expression validation of ceRNA regulatory axis by qRT-PCR.

According to above results, hsa-miR-92b-3p had the potential to regulate CPEB3 and ACADL. Base on the hypothesis of ceRNA regulation, there may be potential circRNA regulating hsa-miR-92b-3p. Therefore, we searched for possible upstream circRNA of hsa-miR-92b-3p through circbank database and GEO liver cancer dataset GSE94508. First, we explored the DEcircRNAs between liver cancer and normal tissues. The cutoff threshold of screening was set to |Log 2-fold change| ≥ 1.0 and adjusted *P* value < 0.05. As shown in Volcano map and Heatmap, the 534 DEcircRNAs included 199 upregulated and 335 downregulated circRNAs (Figures 7A,B). Through the circBank database, setting the binding site ≥ 3 and length <2,000 bp as the screening conditions, we screened 311 potential circRNAs that might regulate has-miR-92b-3p (Figure 7C). By overlapping the data of predicted circRNAs by circBank and DEcircRNAs of GSE94508, we screened has_circ_0077210 as the upstream regulatory molecule of hsa-miR-92b-3p (Figure 7D). Then, the structure and information of has_circ_0077210 were explored by CSCD and circBank database (Figure 7D and Supplementary Table 6).

### Construction of ceRNA Regulatory Axis and Expression Validation by qRT-PCR

**FIGURE 8:**
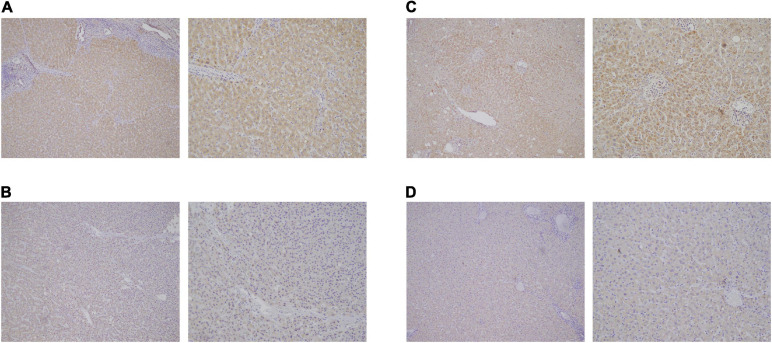
Expression validation of ACADL **(A,B)** and CPEB3 **(C,D)** *via* immunohistochemical (IHC) staining in HCC tissue than normal liver tissues.

By using Cytoscape, we mapped the regulatory axis of hsa_circ_0077210/hsa-miR-92b-3p/CPEB3, ACADL (Figure 7E). Then, the relative expression levels of hsa_circ_0077210, hsa-miR-92b-3p, CPEB3, and ACADL were different between HCC tissue and normal tissue (*P* < 0.05) (Figure 7F). Additionally, IHC staining showed that ACADL (Figures 8A,B) and CPEB3 (Figures 8C,D) expression level was significantly lower in HCC tissue than normal liver tissues.

## Discussion

In developing countries, HCC is the most common pathological type of liver cancer. Most HCC patients fail to prolong their life due to tumor metastasis or recurrence, and the specific mechanism remains unclear. Although it has been proven that radical liver cancer resection is an effective treatment for early HCC, most patients remain uncured, since patients could not be diagnosed in time. It is well known that the identification of molecular mechanisms and applicable prognostic factors may be a key step for the treatment of HCC. ncRNA, including lncRNA, miRNA, and circRNA, is transcribed from the genome without being translated into protein. ncRNA can control gene expression at various levels, including epigenetic modification (Wang et al., 2019b; Yu et al., 2019), transcription (Wang et al., 2019b), RNA splicing (Fan et al., 2019; Jin et al., 2019), scaffold assembly (Fan et al., 2019), etc. Consequently, they may participate in the development of various human malignancies. Understanding the roles of circRNAs in HCC remains a fundamental unmet medical need. Considering this, we screened the circRNAs relevant to the progression of HCC, and a ceRNA regulation network was conducted.

Based on public databases (GEO and TCGA), we compared the differentially expressed genes in HCC. Based on multiple databases, a ceRNA axis (circRNA/miRNA/mRNA) in HCC was established. Additionally, functional analysis was performed to infer the biological functions of key genes in HCC. Finally, the key tumor suppression axis (hsa_circ_0077210/hsa-miR-92b-3p/CPEB3 and ACADL) was constructed. Our data provided a new avenue to mine circRNAs related to the ceRNA axis in HCC and identified potential prognostic and therapeutic targets.

In recent years, ncRNA has received extensive attention. With the ceRNA hypothesis forward, the role of circRNA in the occurrence of HCC has more motivation to be detached. Previous studies have shown that circRNA has transcriptional disorder in many cancers, including HCC, which might be related to tumor proliferation and metastasis. Zheng et al., 2020 found that *via* inhibiting the expression of circSEPT9, the proliferation, migration, and invasion of triple-negative breast cancer (TNBC) may be inhibited, and the apoptosis and autophagy of TNBC cells could be induced. The result showed that circRNA played a significant role in the occurrence and development of refractory breast cancer. Yang et al., 2020 verified the RNA expression level of circPTK2 in colon cancer and normal colon tissues and verified that the expression content of circPTK2 is related to the clinical stage of colon cancer patients both *in vitro* and in patient-derived tumor xenograft (PDTX) models. In addition, they confirmed that increased expression of circPTK2 promoted the metastasis of colon cancer. The results of Chen et al., 2020 confirmed that circSnx5 could act as a miR-544 sponge to reduce SOCS1 target inhibition and inhibit the nuclear transport of PU.1, thus regulating the activation and function of dendritic cells (DCs). However, hsa_circ_0077210 found in our ceRNA network has not been explored. Therefore, the regulatory mechanism of this circRNA in HCC needs to be further studied.

MicroRNA, as a kind of non-coding RNA, could affect the pathological process of various cancers through interfering with the expression level of oncogenes or tumor suppressor genes. Xing et al., 2021 investigated that miR-4521 inhibited the activation of AKT/GSK3β/Snai1 pathway by regulating the expression of Insulin Like Growth Factor 2(IGF2) and Forkhead Box M1(FOXM1), thus restraining the epithelial–mesenchymal transition (EMT) process and metastasis of tumor cells. In fact, the role of hsa-miR-92b-3p had been partly detected in many cancers. For example, Wang et al., 2019a confirmed that upregulated hsa-miR-92b-3p could weaken the G0/G1 phase and induce migration and invasion of esophageal squamous cell carcinoma (ESCC) cells *in vitro* by directly targeting KLF4 and DSC2. However, the relationship between has-miR-92b-3p and HCC and its regulatory mechanism have not been elucidated.

CPEB3 and ACADL were predicted as target genes of hsa-miR-92b-3p through the downstream prediction with a series of screening conditions. In fact, CPEB3 and ACADL might participate in the malignant progress of HCC. Tang et al., 2017 found that CPEB3 was involved in the regulation of mir-452-3p in HCC, and knockdown of CPEB3 could induce autophagy of HCC cells and increase the migration, invasion, and proliferation abilities of HCC cells. Zhao et al., 2020 found that, in liver cancer, the expression levels of ACADL and YAP are negatively correlated. Huang et al., 2014 confirmed that the lack of ACADL can promote the occurrence of HCC by reducing the expression level of PTEN. It had been demonstrated that Yes1 Associated Transcriptional Regulator (YAP) suppresses Phosphatase and Tensin Homolog (PTEN) *via* regulating miR-29 (Tumaneng et al., 2012). Therefore, ACADL may inhibit the occurrence of liver cancer through YAP/PTEN signaling pathway. Based on the GSEA of CPEB3 and ACADL, a visual overview of the mechanisms by which CPEB3 and ACADL regulate HCC development. The results show that CPEB3 and ACADL can actively regulate the cell cycle, G2M checkpoint, mTORC1 signaling, MYC TARGET V1, MYC TARGET V2, and WNT BETA CATENIN signaling pathways. Rapamycin Complex 1 (mTORC1) can sense the level of nutrients in cells (including amino acids, glucose, growth factors, and oxygen) to regulate the activity of downstream substrates (Rogala et al., 2019). In the background of HCC, the downstream of mTORC1 includes (S6K1, also known as p70S6 kinase) S6K1, rpS6, 4E-BP1, and EMT processes (Dong et al., 2020). C-myc is a transcription factor, which has abnormal expression and regulation in most human malignant tumors (Madden et al., 2021). Its direct inhibition has been proved to induce rapid regression of various tumor models in mice (Bulaeva et al., 2020; Liu et al., 2021; Wu et al., 2021). In Sanchez-Vega’s research, based on the data of mRNA expression, mutation, copy number change, gene fusion, and DNA methylation, the mechanism and pattern of somatic changes in 9,125 cases of tumors described in TCGA were studied and analyzed. Multiple carcinogenic pathways, cell cycle, hippo, Myc, Notch, Nrf2, PI-3-kinase/Akt, RTK-RAS, TGF-β signal transduction, p53, and β-catenin/Wnt, were identified (Sanchez-Vega et al., 2018). Consequently, CPEB3 and ACADL might inhibit the occurrence of HCC through inhibiting a variety of cancer signaling pathways related to tumorigenesis and progression.

There remain some limitations in our research. First, our ceRNA regulatory axis was based on bioinformatics analysis, so the circRNA/miRNA/mRNA regulatory network needs to be confirmed *in vitro* and *in vivo*. Second, the expression level of hsa_circ_0077210 needs to be verified in large RNA samples from HCC patients. It needs to be proven that the transcription level of hsa_circ_0077210 is related to the prognosis in the external verification set.

Our research provides a mechanistic elucidation of the unknown ceRNA regulatory network in HCC. Multivariate analysis showed that CPEB3 and ACADL were independent prognostic factors, suggesting that hsa_circ_0077210/hsa-miR-92b-3p/CPEB3 and ACADL axis might play an inhibitory role in the occurrence and progression of HCC. Therefore, hsa_circ_0077210 might become a potential target to inhibit the occurrence and development of HCC.

## Data Availability Statement

The datasets presented in this study can be found in online repositories. The names of the repository/repositories and accession number(s) can be found in the article/Supplementary Material.

## Ethics Statement

All the patients provided written informed consent, and the protocol was approved by the ethical committee of the Affiliated Cancer Hospital of Nanjing Medical University, Jiangsu Cancer Hospital, and Jiangsu Institute of Cancer Research.

## Author Contributions

LC and JW designed this work. RD and XC performed the experiment and wrote the manuscript. YD and YT performed the bioinformatics analysis. XC and SW performed the pathology experiment and data review. All authors have read and approved the manuscript.

## Conflict of Interest

The authors declare that the research was conducted in the absence of any commercial or financial relationships that could be construed as a potential conflict of interest.

## Footnotes

1 http://www.ncbi.nlm.nih.gov/geo/

2 http://www.circbase.org

3 http://diana.imis.athena-innovation.gr/

4 http://www.targetscan.org/

5 http://www.circbank.cn/

6 http://gb.whu.edu.cn/CSCD/

## Supplementary Material

Supplementary Figure 1 Functional enrichment analysis of the co-expressed mRNAs in the key modules. **(A)** GO functional enrichment analysis of selected co-expressed mRNAs. **(B)** KEGG functional enrichment analysis of selected co-expressed mRNAs.

Supplementary Figure 2 Overall survival (OS) analysis and plotted survival curves of the top two significant modules in WGCNA-mRNA.

Supplementary Figure 3 The different expression of CPEB3 **(A)** and ACADL **(B)** in clinical TNM stage, pathological stage and grade of patient with LIHC.

Supplementary Figure 4 The correlation between expression levels of CPEB3 **(A)** and ACADL **(B)** and immune cells.

Supplementary Table 1 Different Expressed miRNA in liver cancer.

Supplementary Table 2 Different Expressed mRNA in liver cancer.

Supplementary Table 3 46 hub miRNAs in blue module.

Supplementary Table 4 119 hub genes in yellow module.

Supplementary Table 5 167 hub genes in brown module.

Supplementary Table 6 Basic information about hsa_circ_0077210 via circbase database.
