# Supplementary figures and images for: Construction of circRNA-Based ceRNA Network to Reveal the Role of circRNAs in the Progression and Prognosis of Hepatocellular Carcinoma
Source: Front Genet. 2021 Feb 26;12:626764. doi: 10.3389/fgene.2021.626764 (PMC7953168; doi:10.3389/fgene.2021.626764)

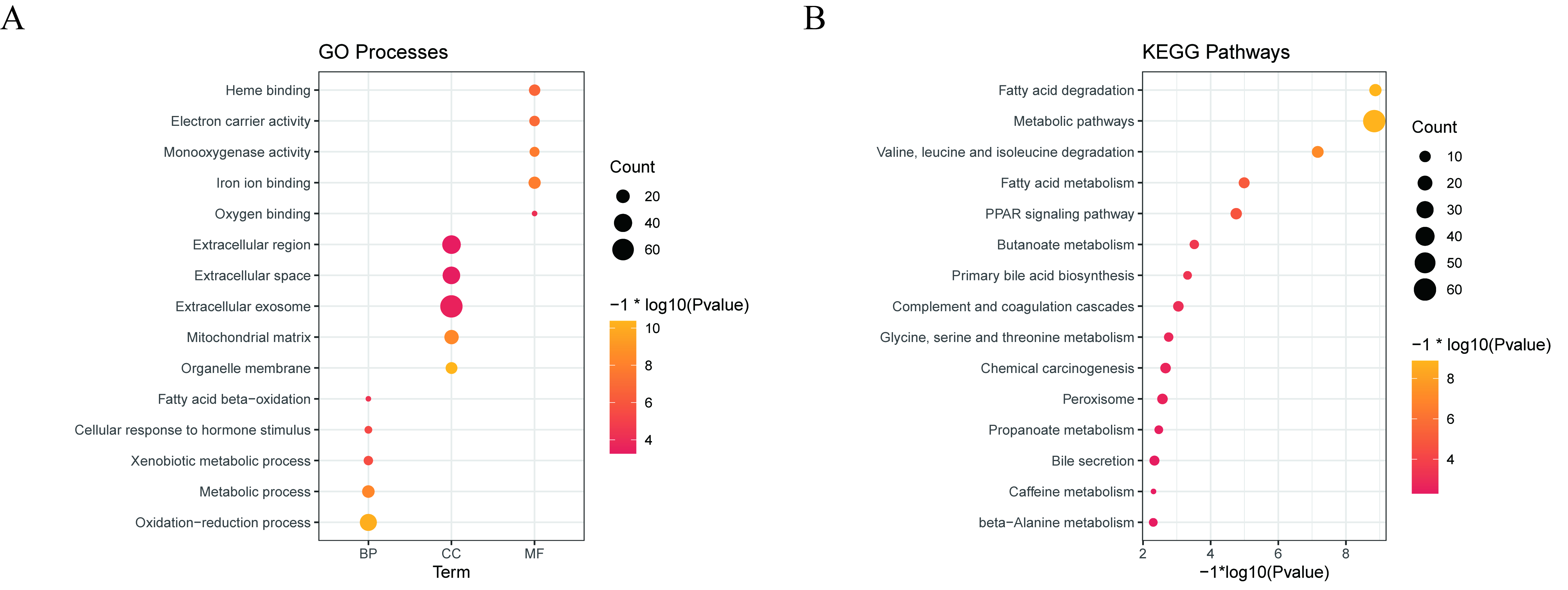

Supplement: Supplementary Figure 1 — Functional enrichment analysis of the co-expressed mRNAs in the key modules. (A) GO functional enrichment analysis of selected co-expressed mRNAs. (B) KEGG functional enrichment analysis of selected co-expressed mRNAs. [file Image_1.TIF]

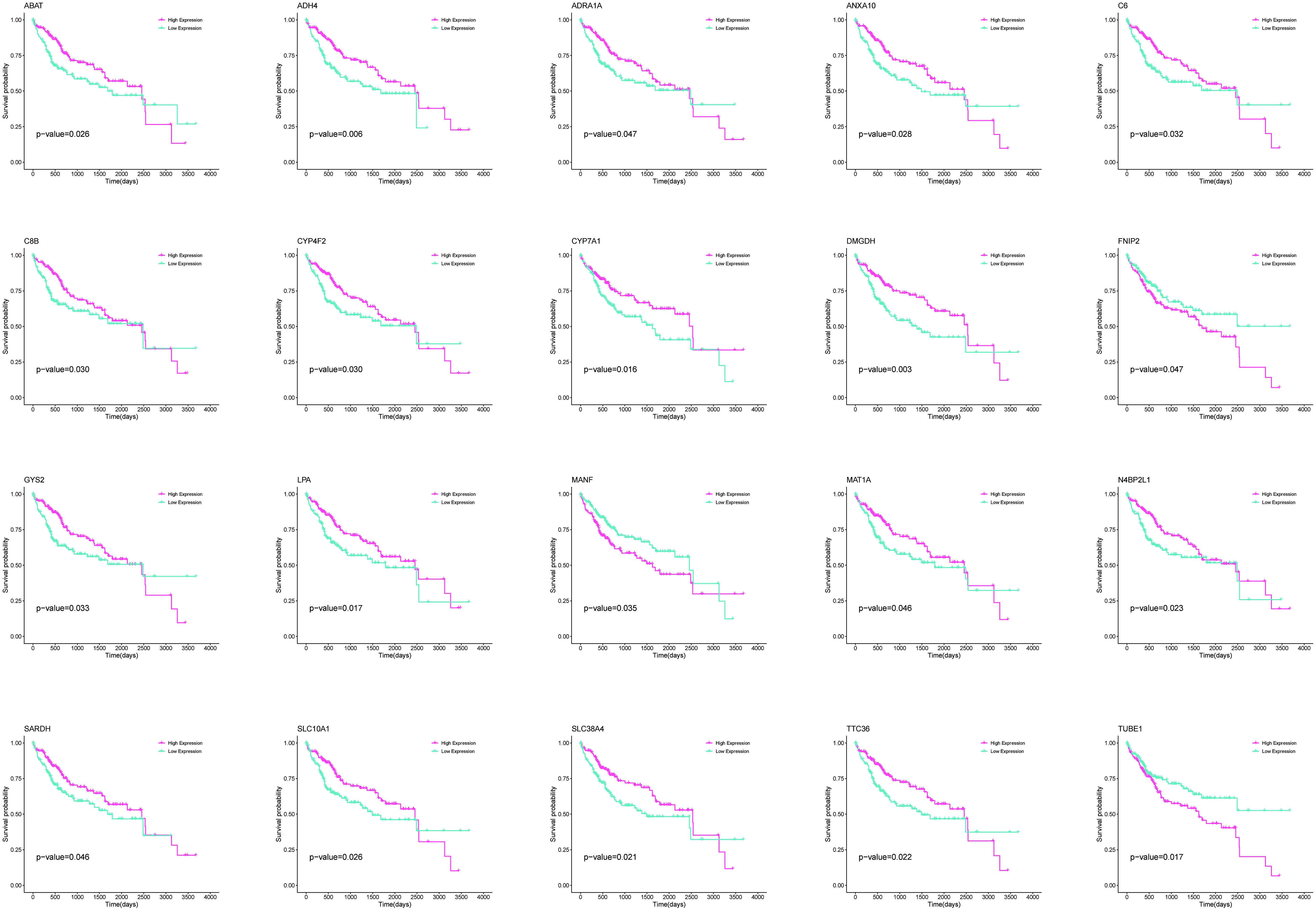

Supplement: Supplementary Figure 2 — Overall survival (OS) analysis and plotted survival curves of the top two significant modules in WGCNA-mRNA. [file Image_2.TIF]

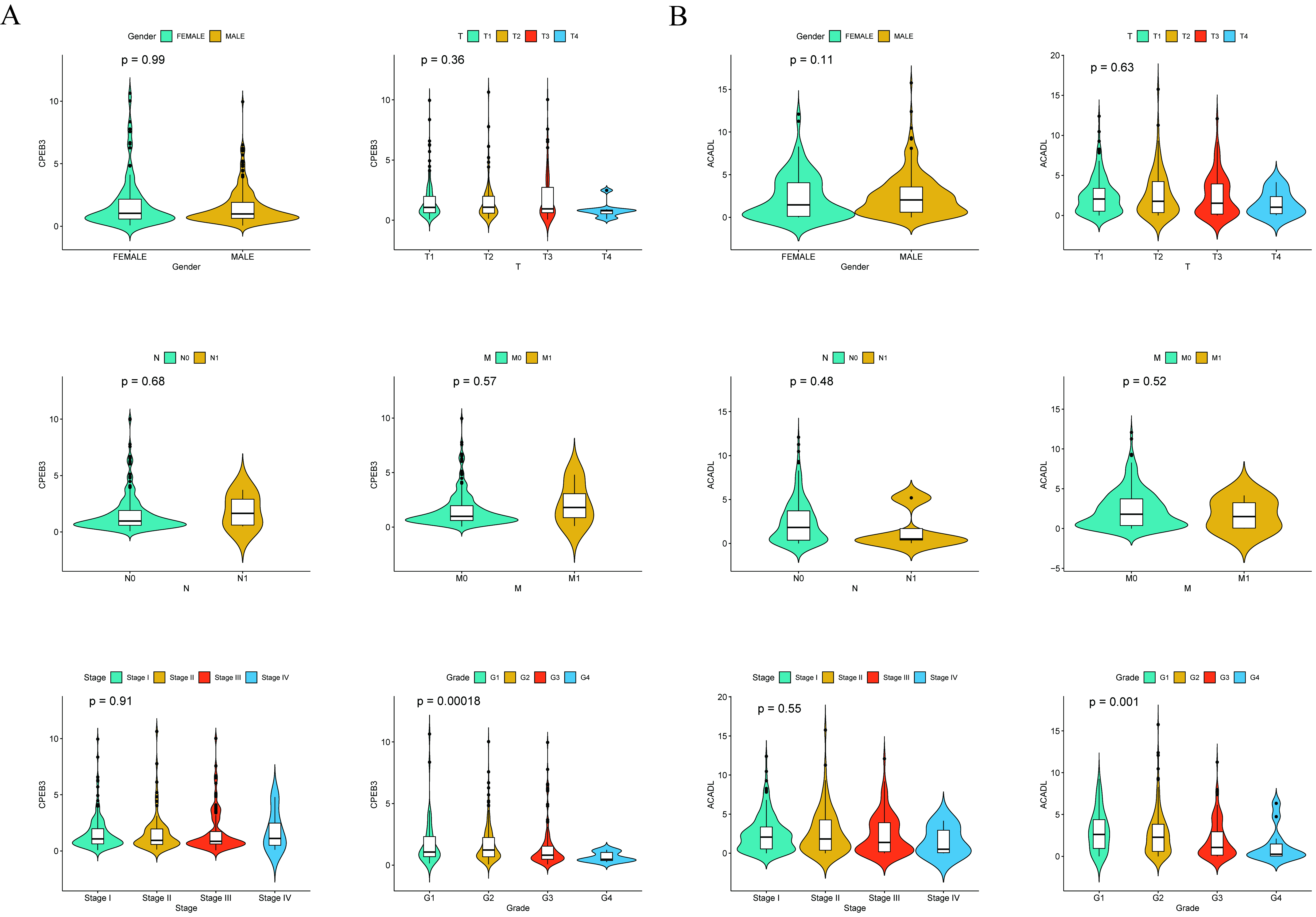

Supplement: Supplementary Figure 3 — The different expression of CPEB3 (A) and ACADL (B) in clinical TNM stage, pathological stage and grade of patient with LIHC. [file Image_3.TIF]

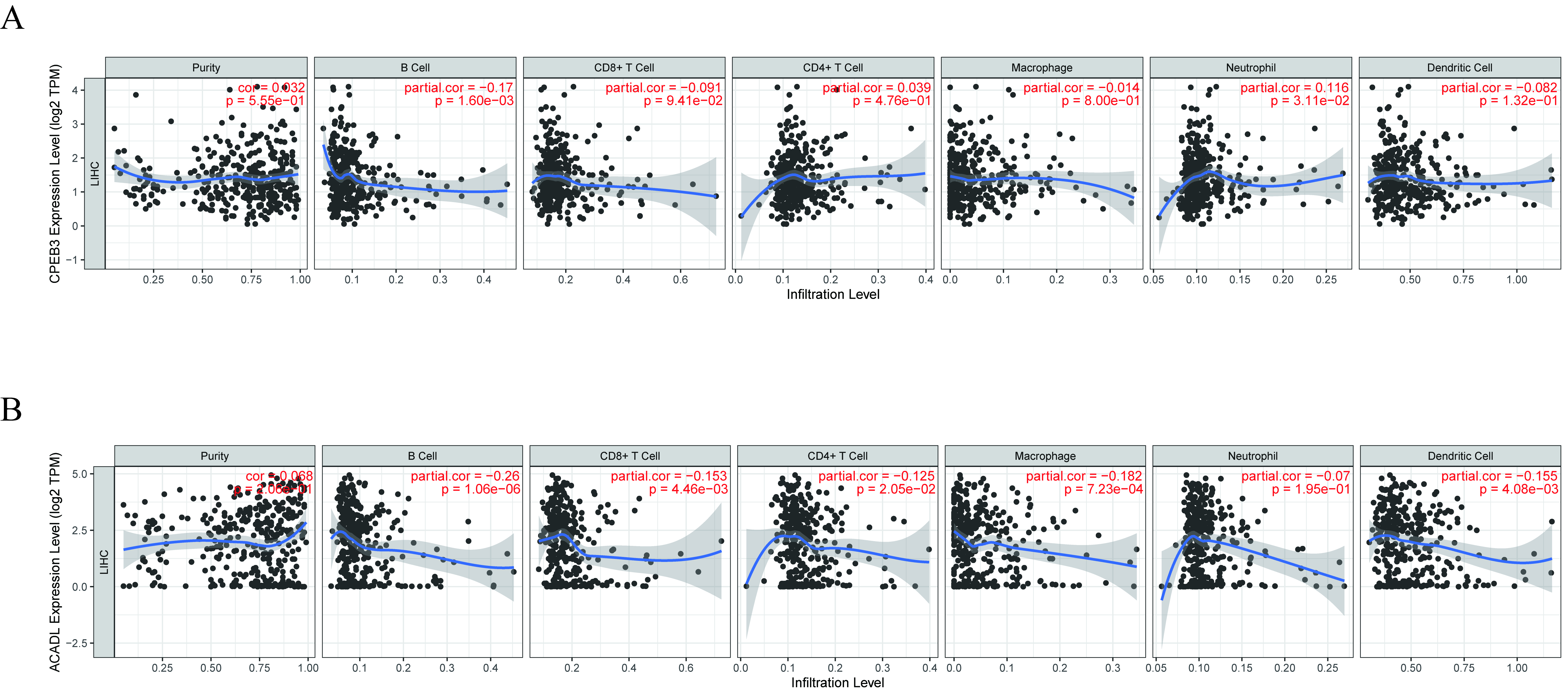

Supplement: Supplementary Figure 4 — The correlation between expression levels of CPEB3 (A) and ACADL (B) and immune cells. [file Image_4.TIF]
