# Supplementary material for: Construction of circRNA-Based ceRNA Network to Reveal the Role of circRNAs in the Progression and Prognosis of Hepatocellular Carcinoma
Source: Front Genet. 2021 Feb 26;12:626764. doi: 10.3389/fgene.2021.626764 (PMC7953168; doi:10.3389/fgene.2021.626764)
Supplement: Supplementary Table 1 — Different Expressed miRNA in liver cancer. [file Table_1.docx]

**Table S1. Different Expressed miRNA in liver cancer**

| gene | Con Mean | Treat Mean | logFC | P Value | FDR |
| --- | --- | --- | --- | --- | --- |
| hsa-miR-421 | 6.651623529 | 18.21578039 | 1.453410382 | 1.55E-13 | 1.02E-12 |
| hsa-miR-501-3p | 121.8296941 | 330.472502 | 1.439664422 | 3.56E-22 | 1.67E-20 |
| hsa-miR-1269a | 64.39856471 | 3542.013239 | 5.78139726 | 1.48E-08 | 4.59E-08 |
| hsa-miR-1307-3p | 3691.508047 | 8873.845556 | 1.265349141 | 2.42E-17 | 3.29E-16 |
| hsa-miR-581 | 1.417294118 | 3.896015686 | 1.458860308 | 4.77E-10 | 1.75E-09 |
| hsa-miR-142-5p | 582.3656706 | 289.2007686 | -1.009853927 | 3.24E-13 | 1.94E-12 |
| hsa-miR-337-5p | 3.189223529 | 1.016269804 | -1.649921752 | 1.82E-12 | 9.18E-12 |
| hsa-miR-34c-3p | 1.115105882 | 8.632944314 | 2.952671978 | 9.25E-10 | 3.34E-09 |
| hsa-miR-25-5p | 0.474282353 | 1.384458039 | 1.545503234 | 0.000638967 | 0.001050593 |
| hsa-miR-5589-3p | 81.70489412 | 37.45719843 | -1.125179499 | 8.16E-13 | 4.44E-12 |
| hsa-miR-7850-5p | 1.407905882 | 0.663871373 | -1.084575247 | 2.43E-09 | 8.27E-09 |
| hsa-miR-877-5p | 2.929835294 | 9.933810196 | 1.761527618 | 1.22E-15 | 1.07E-14 |
| hsa-miR-939-5p | 2.128541176 | 5.022635294 | 1.238579522 | 1.19E-07 | 3.15E-07 |
| hsa-miR-184 | 3.853741176 | 104.0680784 | 4.755124117 | 9.10E-06 | 1.85E-05 |
| hsa-miR-221-3p | 238.5336 | 703.6718463 | 1.560710293 | 2.64E-18 | 4.74E-17 |
| hsa-let-7c-3p | 121.5744 | 50.70145882 | -1.261740308 | 3.34E-17 | 4.10E-16 |
| hsa-miR-532-5p | 2898.155765 | 8346.655404 | 1.526063073 | 3.99E-22 | 1.74E-20 |
| hsa-miR-34b-3p | 0.163741176 | 3.250092549 | 4.310991729 | 3.47E-07 | 8.43E-07 |
| hsa-miR-6502-5p | 3.131011765 | 1.191865098 | -1.393407977 | 3.28E-11 | 1.37E-10 |
| hsa-miR-3614-5p | 28.87312941 | 13.22992 | -1.125923145 | 3.03E-13 | 1.83E-12 |
| hsa-miR-103a-3p | 70069.93195 | 156628.6458 | 1.160480691 | 4.69E-19 | 9.53E-18 |
| hsa-miR-362-3p | 10.05162353 | 22.30558431 | 1.149976398 | 2.63E-11 | 1.11E-10 |
| hsa-miR-34a-5p | 794.1552 | 2052.725211 | 1.370047631 | 4.90E-14 | 3.36E-13 |
| hsa-miR-4800-3p | 1.451364706 | 0.392269804 | -1.887491903 | 8.73E-13 | 4.68E-12 |
| hsa-miR-3677-3p | 6.5008 | 20.94096941 | 1.687639055 | 1.29E-12 | 6.74E-12 |
| hsa-miR-10b-5p | 8225.079835 | 109228.9263 | 3.731181473 | 4.23E-21 | 1.35E-19 |
| hsa-miR-4326 | 9.736682353 | 29.97989961 | 1.622493371 | 3.21E-12 | 1.52E-11 |
| hsa-miR-502-3p | 34.69752941 | 72.19091451 | 1.056984339 | 7.13E-16 | 6.69E-15 |
| hsa-miR-130a-3p | 524.2476471 | 255.675131 | -1.035936642 | 6.22E-20 | 1.58E-18 |
| hsa-miR-134-3p | 6.961858824 | 2.936875294 | -1.245190551 | 8.12E-16 | 7.50E-15 |
| hsa-miR-93-5p | 9613.195812 | 34034.34462 | 1.823903303 | 3.87E-25 | 2.95E-23 |
| hsa-miR-490-3p | 36.63884706 | 3.489192157 | -3.392409149 | 5.17E-31 | 1.05E-28 |
| hsa-miR-5187-5p | 1.837482353 | 3.96448 | 1.109401253 | 7.64E-05 | 0.000139118 |
| hsa-miR-1266-5p | 9.888352941 | 39.57463529 | 2.000773912 | 6.65E-16 | 6.34E-15 |
| hsa-miR-577 | 1.097882353 | 5.578801569 | 2.345231771 | 0.026430287 | 0.03437628 |
| hsa-let-7c-5p | 22223.04927 | 11021.4203 | -1.011746633 | 1.29E-17 | 1.83E-16 |
| hsa-miR-520a-3p | 1.026611765 | 173.1812329 | 7.39824809 | 0.000296776 | 0.000507096 |
| hsa-miR-4742-3p | 2.038517647 | 4.992542745 | 1.292254334 | 4.82E-08 | 1.37E-07 |
| hsa-miR-1295a | 2.909882353 | 13.66333176 | 2.231276593 | 0.042444936 | 0.053165115 |
| hsa-miR-4536-3p | 2.197129412 | 0.910541176 | -1.270823682 | 1.24E-11 | 5.39E-11 |
| hsa-miR-224-3p | 9.343647059 | 37.89667137 | 2.020013451 | 4.15E-09 | 1.38E-08 |
| hsa-miR-130a-5p | 5.638 | 2.447563922 | -1.20383694 | 5.34E-13 | 3.05E-12 |
| hsa-miR-196b-5p | 44.46223529 | 481.0308298 | 3.435476976 | 0.016590821 | 0.02223928 |
| hsa-miR-3923 | 0.323788235 | 80.88928941 | 7.96475431 | 9.82E-05 | 0.000176243 |
| hsa-miR-512-3p | 0.555082353 | 130.278582 | 7.874682378 | 0.000486625 | 0.000808831 |
| hsa-miR-4433b-5p | 1.083058824 | 0.252414118 | -2.101247093 | 3.41E-09 | 1.14E-08 |
| hsa-miR-17-5p | 1930.575365 | 4659.578776 | 1.271168667 | 2.13E-11 | 9.10E-11 |
| hsa-miR-338-3p | 1218.236894 | 3156.129145 | 1.373361538 | 8.38E-06 | 1.71E-05 |
| hsa-miR-3200-3p | 3.929670588 | 21.58709647 | 2.457688923 | 3.36E-10 | 1.24E-09 |
| hsa-miR-3117-3p | 0.611835294 | 2.450798431 | 2.002036596 | 0.001636108 | 0.002513918 |
| hsa-miR-6844 | 0.243717647 | 1.329374118 | 2.447464548 | 5.94E-05 | 0.000109469 |
| hsa-miR-7-5p | 1.943882353 | 3.916316863 | 1.010556589 | 0.007793863 | 0.011186485 |
| hsa-miR-1292-5p | 1.020682353 | 2.428727843 | 1.25066688 | 2.89E-06 | 6.34E-06 |
| hsa-miR-3682-3p | 3.953647059 | 8.57961098 | 1.117728146 | 8.09E-08 | 2.22E-07 |
| hsa-miR-6783-3p | 0.217294118 | 1.074836078 | 2.306395628 | 2.85E-05 | 5.46E-05 |
| hsa-miR-19a-3p | 108.7191529 | 330.3500518 | 1.60338945 | 2.52E-13 | 1.56E-12 |
| hsa-miR-30d-5p | 32864.96819 | 78395.78525 | 1.254225505 | 2.49E-15 | 2.00E-14 |
| hsa-miR-188-5p | 6.729858824 | 19.25863843 | 1.516857564 | 1.91E-14 | 1.37E-13 |
| hsa-miR-1294 | 1.232235294 | 0.586236863 | -1.07172217 | 5.64E-05 | 0.000104164 |
| hsa-miR-122-3p | 1785.090306 | 867.6245051 | -1.040854355 | 4.14E-18 | 6.82E-17 |
| hsa-miR-526b-5p | 3.099529412 | 295.1243639 | 6.573130023 | 0.002790638 | 0.004224043 |
| hsa-miR-190b-5p | 0.636658824 | 6.590086275 | 3.371704988 | 4.28E-11 | 1.73E-10 |
| hsa-miR-589-3p | 3.622964706 | 9.911523137 | 1.451936027 | 1.68E-13 | 1.08E-12 |
| hsa-miR-653-5p | 24.24755294 | 80.12423529 | 1.724399525 | 0.037465807 | 0.047415232 |
| hsa-miR-4800-5p | 0.982117647 | 0.18652549 | -2.396523054 | 3.82E-18 | 6.47E-17 |
| hsa-miR-335-5p | 107.1552235 | 49.75306667 | -1.106844821 | 1.51E-14 | 1.09E-13 |
| hsa-miR-4683 | 1.655152941 | 0.582321569 | -1.507076572 | 1.96E-08 | 5.95E-08 |
| hsa-miR-222-3p | 57.49771765 | 164.5156643 | 1.516648361 | 4.34E-18 | 6.96E-17 |
| hsa-miR-937-3p | 5.036117647 | 15.76950588 | 1.646753565 | 9.15E-07 | 2.08E-06 |
| hsa-miR-6503-5p | 3.851529412 | 1.250224314 | -1.623244478 | 5.87E-13 | 3.31E-12 |
| hsa-miR-4442 | 1.696282353 | 0.711814902 | -1.252802291 | 5.46E-08 | 1.53E-07 |
| hsa-miR-199a-3p | 11543.31266 | 4849.450293 | -1.251164177 | 3.36E-17 | 4.10E-16 |
| hsa-miR-9-5p | 586.5518353 | 6894.212511 | 3.555055256 | 5.60E-06 | 1.18E-05 |
| hsa-miR-551a | 2.030164706 | 0.818214902 | -1.311045059 | 1.41E-09 | 4.95E-09 |
| hsa-miR-660-5p | 190.3279294 | 451.5872063 | 1.246517331 | 5.07E-17 | 5.72E-16 |
| hsa-miR-3934-5p | 0.452588235 | 1.387099608 | 1.615800403 | 0.008078026 | 0.011540038 |
| hsa-miR-6514-5p | 0.472658824 | 1.359673725 | 1.524389404 | 2.28E-05 | 4.44E-05 |
| hsa-miR-144-3p | 245.4967529 | 71.70193255 | -1.775620034 | 4.42E-21 | 1.35E-19 |
| hsa-miR-6726-3p | 0.718305882 | 0.247065098 | -1.539707109 | 7.47E-08 | 2.06E-07 |
| hsa-miR-133b | 3.943717647 | 1.961038431 | -1.007938456 | 8.74E-13 | 4.68E-12 |
| hsa-miR-105-5p | 0.288305882 | 220.8478494 | 9.581236796 | 4.19E-11 | 1.70E-10 |
| hsa-miR-6788-3p | 1.110329412 | 2.763673725 | 1.315599546 | 0.000237128 | 0.000409768 |
| hsa-miR-20a-5p | 1565.974071 | 4036.98187 | 1.366216783 | 5.92E-12 | 2.64E-11 |
| hsa-miR-3680-3p | 0.426564706 | 1.401791373 | 1.716435144 | 0.000229714 | 0.000398084 |
| hsa-miR-33b-3p | 8.226423529 | 3.260668235 | -1.335097692 | 1.14E-15 | 1.02E-14 |
| hsa-miR-130b-3p | 66.32816471 | 158.2085051 | 1.254133647 | 1.03E-08 | 3.31E-08 |
| hsa-miR-490-5p | 2.3864 | 0.115689412 | -4.366507147 | 1.49E-45 | 4.56E-43 |
| hsa-miR-3662 | 0.614282353 | 4.86032 | 2.98407746 | 2.31E-09 | 7.90E-09 |
| hsa-miR-6734-5p | 0.420282353 | 1.060448627 | 1.335243946 | 0.000532337 | 0.000880015 |
| hsa-miR-18a-5p | 36.35512941 | 113.3270243 | 1.640261096 | 3.46E-12 | 1.62E-11 |
| hsa-miR-942-3p | 0.901105882 | 2.266999216 | 1.33101535 | 4.62E-05 | 8.64E-05 |
| hsa-miR-122b-5p | 2.891576471 | 16.21509961 | 2.487409725 | 3.37E-08 | 9.90E-08 |
| hsa-miR-424-3p | 38.17185882 | 15.34570039 | -1.314674949 | 8.76E-23 | 4.86E-21 |
| hsa-miR-376a-2-5p | 4.037482353 | 1.172969412 | -1.783290562 | 2.82E-15 | 2.23E-14 |
| hsa-miR-1276 | 0.412070588 | 1.576392157 | 1.935663077 | 5.61E-06 | 1.18E-05 |
| hsa-miR-199b-3p | 10746.88268 | 4553.37494 | -1.238910077 | 3.10E-17 | 3.94E-16 |
| hsa-miR-1248 | 6.277717647 | 1.600047059 | -1.972125806 | 1.86E-17 | 2.58E-16 |
| hsa-miR-6516-5p | 0.6916 | 1.916269804 | 1.470290928 | 9.56E-05 | 0.000172573 |
| hsa-miR-500b-3p | 4.429341176 | 11.05225412 | 1.319176606 | 3.79E-12 | 1.75E-11 |
| hsa-miR-183-3p | 0.196494118 | 1.939686275 | 3.3032653 | 3.30E-07 | 8.05E-07 |
| hsa-miR-1251-5p | 0.174423529 | 10.32133333 | 5.886890873 | 9.44E-08 | 2.54E-07 |
| hsa-miR-3691-5p | 0.847458824 | 2.024454902 | 1.256318328 | 0.000215759 | 0.000374967 |
| hsa-miR-96-5p | 5.327811765 | 75.40588235 | 3.823062054 | 3.51E-12 | 1.63E-11 |
| hsa-miR-4746-5p | 3.357976471 | 19.61667765 | 2.546416696 | 4.94E-22 | 2.01E-20 |
| hsa-miR-7845-5p | 0.998917647 | 0.319752157 | -1.643411653 | 3.18E-08 | 9.42E-08 |
| hsa-miR-4664-5p | 0.715623529 | 2.047780392 | 1.516788278 | 9.31E-05 | 0.000168448 |
| hsa-miR-20b-5p | 38.01797647 | 109.8153412 | 1.530325962 | 0.000103406 | 0.000184978 |
| hsa-miR-500a-5p | 13.70475294 | 31.66553725 | 1.208237237 | 4.18E-15 | 3.15E-14 |
| hsa-miR-3074-5p | 17.68065882 | 36.14305569 | 1.031546449 | 2.74E-05 | 5.28E-05 |
| hsa-miR-1270 | 2.559717647 | 11.91544784 | 2.218776591 | 3.70E-06 | 8.02E-06 |
| hsa-miR-147b-3p | 0.635388235 | 1.998682353 | 1.653338923 | 0.026904809 | 0.034919008 |
| hsa-miR-552-5p | 6.400352941 | 249.7622494 | 5.286260162 | 0.0340635 | 0.043561289 |
| hsa-miR-139-5p | 1569.836306 | 596.1912502 | -1.396767023 | 1.28E-22 | 6.51E-21 |
| hsa-miR-4454 | 1.838023529 | 0.79427451 | -1.210445626 | 3.93E-07 | 9.33E-07 |
| hsa-miR-301b-3p | 1.922941176 | 6.54115451 | 1.766230662 | 1.30E-08 | 4.14E-08 |
| hsa-miR-21-5p | 394777.0651 | 1064216.509 | 1.430681606 | 1.46E-26 | 1.27E-24 |
| hsa-miR-34c-5p | 0.782023529 | 15.31262118 | 4.291365435 | 4.64E-13 | 2.72E-12 |
| hsa-miR-760 | 0.857058824 | 2.828348235 | 1.722493629 | 1.76E-05 | 3.45E-05 |
| hsa-miR-766-5p | 0.544376471 | 1.168894118 | 1.102467635 | 0.019081772 | 0.025304089 |
| hsa-miR-92b-3p | 45.04543529 | 100.0371482 | 1.151083014 | 0.000801323 | 0.00129657 |
| hsa-miR-1258 | 64.59814118 | 6.511312941 | -3.310472269 | 5.61E-29 | 8.55E-27 |
| hsa-miR-548d-3p | 0.469576471 | 1.883231373 | 2.003778234 | 6.20E-06 | 1.29E-05 |
| hsa-miR-1226-3p | 2.384352941 | 7.35478902 | 1.625086152 | 5.20E-07 | 1.21E-06 |
| hsa-miR-362-5p | 65.88585882 | 138.9191906 | 1.076205154 | 8.28E-09 | 2.67E-08 |
| hsa-miR-4664-3p | 0.3396 | 2.239796078 | 2.721459025 | 1.24E-08 | 3.95E-08 |
| hsa-miR-301a-3p | 14.81814118 | 37.9144251 | 1.355382363 | 2.21E-14 | 1.56E-13 |
| hsa-miR-139-3p | 471.1385647 | 124.4433694 | -1.920662065 | 1.40E-27 | 1.71E-25 |
| hsa-miR-500b-5p | 13.70475294 | 31.67904941 | 1.208852725 | 3.98E-15 | 3.10E-14 |
| hsa-miR-6516-3p | 0.451694118 | 1.261888627 | 1.482166555 | 0.001009161 | 0.001603095 |
| hsa-miR-10b-3p | 3.8256 | 48.36719373 | 3.660270894 | 1.72E-18 | 3.29E-17 |
| hsa-miR-182-5p | 5039.918259 | 52526.62183 | 3.381576562 | 4.50E-12 | 2.06E-11 |
| hsa-miR-4686 | 4.348470588 | 0.289935686 | -3.906703255 | 2.03E-50 | 1.24E-47 |
| hsa-miR-205-5p | 3.091388235 | 49.22835765 | 3.993162856 | 0.002931335 | 0.004426026 |
| hsa-miR-4791 | 3.2748 | 1.538723137 | -1.08967313 | 4.77E-09 | 1.57E-08 |
| hsa-miR-34a-3p | 2.656164706 | 6.343083922 | 1.25583982 | 4.50E-08 | 1.28E-07 |
| hsa-miR-5586-5p | 7.606635294 | 17.75351216 | 1.222774119 | 2.07E-07 | 5.21E-07 |
| hsa-miR-146b-5p | 3642.963318 | 8628.516816 | 1.244000123 | 4.13E-05 | 7.78E-05 |
| hsa-miR-589-5p | 183.7694353 | 528.7956549 | 1.524813486 | 3.67E-24 | 2.49E-22 |
| hsa-miR-542-5p | 19.42644706 | 8.698792157 | -1.159135069 | 7.71E-19 | 1.52E-17 |
| hsa-miR-6716-3p | 0.717247059 | 1.818817255 | 1.342458544 | 0.000164307 | 0.000288009 |
| hsa-miR-508-3p | 23.82670588 | 110.1859545 | 2.209288921 | 2.50E-09 | 8.48E-09 |
| hsa-miR-501-5p | 7.048564706 | 19.84177569 | 1.493139724 | 4.18E-15 | 3.15E-14 |
| hsa-miR-301b-5p | 0.654635294 | 2.116338824 | 1.692807329 | 0.001747229 | 0.002677913 |
| hsa-miR-3677-5p | 2.317717647 | 5.807190588 | 1.325135561 | 1.03E-06 | 2.32E-06 |
| hsa-miR-10a-3p | 13.23207059 | 6.102839216 | -1.116486349 | 2.49E-14 | 1.75E-13 |
| hsa-miR-514a-3p | 12.44738824 | 63.75933804 | 2.35679359 | 1.79E-08 | 5.46E-08 |
| hsa-miR-671-5p | 8.074847059 | 18.71717333 | 1.212855736 | 2.32E-13 | 1.46E-12 |
| hsa-miR-1180-3p | 38.29327059 | 131.44 | 1.779241596 | 3.70E-17 | 4.43E-16 |
| hsa-miR-4661-5p | 10.26336471 | 42.17863843 | 2.039008748 | 2.83E-11 | 1.19E-10 |
| hsa-miR-7974 | 0.253411765 | 1.803739608 | 2.831435674 | 1.65E-05 | 3.26E-05 |
| hsa-miR-2114-3p | 0.607388235 | 5.948316863 | 3.291790631 | 0.023077346 | 0.030273508 |
| hsa-miR-2114-5p | 1.429388235 | 15.61703843 | 3.449651167 | 0.001141583 | 0.001804056 |
| hsa-miR-765 | 0.550305882 | 2.169901176 | 1.979323685 | 2.56E-07 | 6.37E-07 |
| hsa-miR-214-3p | 38.86934118 | 14.27545725 | -1.445095698 | 1.15E-17 | 1.71E-16 |
| hsa-miR-503-3p | 7.505552941 | 3.684787451 | -1.026376958 | 8.75E-11 | 3.38E-10 |
| hsa-miR-509-3p | 5.183152941 | 29.97434039 | 2.531826137 | 8.25E-07 | 1.89E-06 |
| hsa-miR-376c-5p | 8.341058824 | 3.443595294 | -1.276314932 | 1.63E-13 | 1.06E-12 |
| hsa-miR-636 | 0.346188235 | 1.02854902 | 1.57098195 | 0.001050134 | 0.001663849 |
| hsa-miR-454-3p | 18.6656 | 40.73471373 | 1.125876885 | 5.31E-13 | 3.05E-12 |
| hsa-miR-195-5p | 374.1033176 | 156.4994949 | -1.257278759 | 2.75E-19 | 5.96E-18 |
| hsa-miR-5010-3p | 2.402047059 | 5.278782745 | 1.135940876 | 1.73E-07 | 4.47E-07 |
| hsa-miR-1269b | 5.731623529 | 594.6467012 | 6.696945109 | 1.25E-07 | 3.30E-07 |
| hsa-miR-643 | 0.621717647 | 2.120062745 | 1.769775529 | 1.74E-07 | 4.47E-07 |
| hsa-miR-150-3p | 11.85524706 | 5.926625882 | -1.000242833 | 3.32E-08 | 9.80E-08 |
| hsa-miR-5003-3p | 0.561529412 | 1.923563922 | 1.776348277 | 6.40E-07 | 1.48E-06 |
| hsa-miR-219b-3p | 0.752988235 | 2.235090196 | 1.569633822 | 2.96E-07 | 7.27E-07 |
| hsa-miR-4685-3p | 1.360070588 | 0.672439216 | -1.016205762 | 3.80E-05 | 7.19E-05 |
| hsa-miR-135a-5p | 2.2428 | 33.79492392 | 3.913433687 | 3.31E-06 | 7.21E-06 |
| hsa-miR-541-3p | 0.974258824 | 9.105656471 | 3.224386034 | 0.013595381 | 0.018762856 |
| hsa-miR-376b-5p | 8.023576471 | 3.167014902 | -1.341121799 | 6.06E-14 | 4.07E-13 |
| hsa-miR-1301-3p | 18.60042353 | 56.00481882 | 1.590215495 | 1.07E-17 | 1.64E-16 |
| hsa-miR-326 | 46.63284706 | 20.82567843 | -1.162983018 | 2.67E-19 | 5.96E-18 |
| hsa-miR-4751 | 1.040447059 | 0.487686275 | -1.093178283 | 1.45E-08 | 4.50E-08 |
| hsa-miR-6854-5p | 1.5484 | 3.129910588 | 1.015343232 | 0.001178443 | 0.001857494 |
| hsa-miR-103a-2-5p | 3.063929412 | 8.254804706 | 1.429851024 | 4.97E-11 | 1.97E-10 |
| hsa-miR-519a-5p | 0.949882353 | 165.1866824 | 7.442132823 | 0.039062251 | 0.049333277 |
| hsa-miR-452-3p | 11.73811765 | 62.82339137 | 2.420100753 | 1.24E-12 | 6.51E-12 |
| hsa-miR-454-5p | 1.285858824 | 2.944956863 | 1.195514246 | 1.77E-06 | 3.98E-06 |
| hsa-miR-18a-3p | 4.170588235 | 9.58742902 | 1.200893111 | 0.000156783 | 0.000277211 |
| hsa-miR-183-5p | 1319.0456 | 19670.07856 | 3.898436374 | 1.58E-12 | 8.16E-12 |
| hsa-miR-450a-1-3p | 0.948729412 | 0.448941176 | -1.079470248 | 4.50E-05 | 8.45E-05 |
| hsa-miR-92a-1-5p | 10.77484706 | 22.43238275 | 1.057915479 | 0.000240436 | 0.000413144 |
| hsa-miR-101-3p | 226941.6525 | 97527.79792 | -1.218436036 | 2.94E-21 | 9.98E-20 |
| hsa-miR-891a-5p | 0.863294118 | 88.77536627 | 6.684163439 | 7.78E-11 | 3.02E-10 |
| hsa-miR-511-5p | 142.2664706 | 65.03983686 | -1.129200143 | 3.87E-20 | 1.03E-18 |
| hsa-miR-424-5p | 2663.257741 | 563.0962824 | -2.241738523 | 3.43E-27 | 3.48E-25 |
| hsa-miR-369-5p | 58.11388235 | 16.70611451 | -1.798506607 | 3.72E-20 | 1.03E-18 |
| hsa-miR-3127-5p | 7.223835294 | 16.84164706 | 1.221196332 | 7.41E-13 | 4.11E-12 |
| hsa-miR-7705 | 1.929952941 | 4.377995294 | 1.181704734 | 5.40E-05 | 0.000100684 |
| hsa-miR-224-5p | 96.51432941 | 968.2141678 | 3.326511146 | 4.99E-18 | 7.80E-17 |
| hsa-miR-500a-3p | 716.2272 | 2143.340718 | 1.581371994 | 1.98E-23 | 1.21E-21 |
| hsa-miR-450a-5p | 94.32931765 | 43.42610196 | -1.119143773 | 1.32E-21 | 5.02E-20 |
| hsa-miR-4677-3p | 46.30237647 | 94.75747451 | 1.033153506 | 6.65E-13 | 3.72E-12 |
| hsa-miR-33b-5p | 104.7655059 | 37.79529725 | -1.470885146 | 2.90E-17 | 3.76E-16 |
| hsa-miR-3144-3p | 0.163811765 | 3.806255686 | 4.5382616 | 1.42E-09 | 4.97E-09 |
| hsa-miR-1229-3p | 1.217576471 | 3.465477647 | 1.509041828 | 1.47E-05 | 2.94E-05 |
| hsa-miR-5589-5p | 124.5769647 | 54.34775843 | -1.196744889 | 3.71E-16 | 3.84E-15 |
| hsa-miR-452-5p | 264.0544 | 1456.898946 | 2.463993725 | 1.04E-15 | 9.43E-15 |
| hsa-miR-502-5p | 3.716470588 | 8.81468549 | 1.245975904 | 2.00E-08 | 6.04E-08 |
| hsa-miR-7706 | 3.217341176 | 13.20632157 | 2.037287844 | 6.63E-20 | 1.62E-18 |
| hsa-miR-767-5p | 0.158470588 | 137.07088 | 9.756491291 | 3.50E-11 | 1.44E-10 |
