# Supplementary material for: Construction of circRNA-Based ceRNA Network to Reveal the Role of circRNAs in the Progression and Prognosis of Hepatocellular Carcinoma
Source: Front Genet. 2021 Feb 26;12:626764. doi: 10.3389/fgene.2021.626764 (PMC7953168; doi:10.3389/fgene.2021.626764)
Supplement: Supplementary Table 2 — Different Expressed mRNA in liver cancer. [file Table_2.docx]

**Table S2. Different Expressed mRNA in liver cancer**

| gene | Con Mean | Treat Mean | logFC | P Value | FDR |
| --- | --- | --- | --- | --- | --- |
| C11orf16 | 0.008796587 | 0.023753907 | 1.433149097 | 1.06E-08 | 3.17E-08 |
| C1QTNF7 | 0.268756237 | 0.108578027 | -1.307566066 | 9.25E-21 | 1.68E-19 |
| STPG3 | 0.254505461 | 0.744733875 | 1.549028367 | 1.81E-05 | 3.66E-05 |
| RNFT2 | 0.044717029 | 0.317037737 | 2.82575834 | 2.96E-21 | 5.88E-20 |
| ZNF43 | 0.174650243 | 0.459563514 | 1.395795613 | 0.000272998 | 0.000474138 |
| MFAP3L | 7.360342179 | 2.113508419 | -1.800132979 | 2.94E-22 | 7.43E-21 |
| SMIM17 | 0.027499955 | 0.059026002 | 1.101921349 | 0.021606564 | 0.02836768 |
| NR2C2AP | 3.612185385 | 9.176930252 | 1.345139706 | 8.72E-24 | 3.28E-22 |
| CYP8B1 | 149.4849451 | 68.60436862 | -1.123627842 | 4.57E-13 | 2.43E-12 |
| FANCG | 1.012416211 | 2.804667037 | 1.470026995 | 1.55E-23 | 5.40E-22 |
| ARMC9 | 0.150104452 | 0.330144052 | 1.137128882 | 1.02E-10 | 3.94E-10 |
| TAS2R60 | 0.048622018 | 0.023634918 | -1.040689947 | 1.41E-05 | 2.88E-05 |
| MAPK12 | 0.252546364 | 1.160481917 | 2.200103865 | 2.20E-14 | 1.40E-13 |
| DPPA4 | 0.118181004 | 0.032425707 | -1.865788238 | 2.70E-16 | 2.24E-15 |
| PCDHB8 | 0.035809441 | 0.209687177 | 2.549826754 | 0.029542051 | 0.038041328 |
| ATP4A | 0.003994716 | 0.049783592 | 3.639505359 | 0.041615648 | 0.052357776 |
| LRRC69 | 0.060620616 | 0.27483986 | 2.18071083 | 8.46E-16 | 6.53E-15 |
| HERC5 | 4.814297701 | 1.902732423 | -1.339252663 | 7.32E-14 | 4.33E-13 |
| CDKN2B | 0.777122159 | 1.978164845 | 1.34794935 | 6.85E-14 | 4.08E-13 |
| ARHGAP22 | 0.084448621 | 0.256723611 | 1.604070227 | 1.38E-16 | 1.21E-15 |
| SLC22A11 | 0.200932265 | 3.69120173 | 4.199309436 | 0.001078602 | 0.001726962 |
| NT5DC4 | 0.010149001 | 0.030758617 | 1.59965289 | 0.000175364 | 0.000312451 |
| SSX5 | 0.00090592 | 0.13817517 | 7.252898983 | 0.020043785 | 0.026475885 |
| SCGB1C2 | 0.001250734 | 0.000534072 | -1.227668232 | 0.003058991 | 0.004559036 |
| DHRS7C | 0.052766219 | 0.003755797 | -3.812423727 | 1.63E-13 | 9.16E-13 |
| MUC13 | 1.092808276 | 44.3107967 | 5.341546047 | 4.36E-11 | 1.76E-10 |
| NAT9 | 2.851294297 | 6.614812575 | 1.214083325 | 9.22E-24 | 3.43E-22 |
| PKN3 | 1.154945936 | 3.107628567 | 1.427988759 | 1.60E-17 | 1.67E-16 |
| OR13C5 | 0.022419367 | 0.007415731 | -1.596084619 | 7.65E-07 | 1.82E-06 |
| PRR25 | 0.003940611 | 0.017781231 | 2.173864033 | 0.00126529 | 0.002001107 |
| VNN1 | 84.27830916 | 41.90942378 | -1.007886685 | 1.24E-12 | 6.21E-12 |
| CRHR2 | 0.026691705 | 0.101516888 | 1.9272564 | 5.05E-13 | 2.67E-12 |
| HIST1H2BH | 0.045146736 | 0.316190435 | 2.808100126 | 5.60E-05 | 0.000106298 |
| DNAH10OS | 0.118906143 | 0.270335384 | 1.184927108 | 2.33E-05 | 4.63E-05 |
| SPINK5 | 0.043182249 | 0.426268096 | 3.303250781 | 9.86E-14 | 5.72E-13 |
| ECM1 | 18.09278869 | 2.461815252 | -2.877620389 | 4.73E-29 | 4.80E-26 |
| CYP39A1 | 22.83707617 | 4.67812234 | -2.287376458 | 1.93E-25 | 1.30E-23 |
| AC023055.1 | 0.031390442 | 0.071260805 | 1.182783451 | 1.46E-12 | 7.25E-12 |
| MYO1A | 0.091041823 | 0.778288496 | 3.095703686 | 4.58E-11 | 1.85E-10 |
| BHLHA9 | 0.199897218 | 0.034669914 | -2.527502223 | 1.17E-12 | 5.89E-12 |
| GTSF1 | 0.119272065 | 2.36941376 | 4.312202056 | 0.048657135 | 0.060667391 |
| HMMR | 0.230292978 | 2.253531502 | 3.290645288 | 4.74E-26 | 4.01E-24 |
| GRM4 | 0.002387593 | 0.01002667 | 2.070213945 | 0.000919605 | 0.001487237 |
| ITPKA | 0.708721203 | 4.091281847 | 2.529262811 | 6.92E-14 | 4.11E-13 |
| CTSV | 0.045722448 | 1.275576545 | 4.802103031 | 3.02E-10 | 1.10E-09 |
| STRIP2 | 0.06185559 | 0.397764717 | 2.684939421 | 8.62E-18 | 9.33E-17 |
| SATB1 | 1.574001392 | 0.742991182 | -1.083019823 | 4.38E-18 | 4.97E-17 |
| MSRA | 14.98714756 | 6.168390073 | -1.280759922 | 2.03E-23 | 6.81E-22 |
| TMEM178B | 0.029235278 | 0.212928593 | 2.864587488 | 3.75E-05 | 7.29E-05 |
| DAGLA | 0.296240659 | 0.830762845 | 1.487667031 | 1.29E-06 | 2.99E-06 |
| EGF | 0.008066076 | 0.2067465 | 4.679852057 | 0.000252887 | 0.000441623 |
| TDRD5 | 0.005640048 | 0.08227586 | 3.866689775 | 0.021155348 | 0.027815001 |
| HIST1H4H | 0.500258197 | 3.089102644 | 2.626443004 | 7.56E-14 | 4.46E-13 |
| SPAG5 | 2.019550508 | 5.303080056 | 1.3927963 | 3.56E-13 | 1.93E-12 |
| TRIB1 | 68.26847948 | 21.49726487 | -1.667066504 | 6.67E-24 | 2.57E-22 |
| MYO19 | 0.941361726 | 2.171539202 | 1.205896895 | 1.88E-23 | 6.36E-22 |
| COL25A1 | 0.243025392 | 0.041925158 | -2.535218941 | 8.08E-25 | 4.26E-23 |
| CCDC154 | 0.085329665 | 0.360583731 | 2.079215012 | 4.28E-14 | 2.62E-13 |
| APOBEC3A | 0.357685001 | 0.106705456 | -1.745055673 | 1.03E-16 | 9.23E-16 |
| CDH24 | 0.191759851 | 0.916205005 | 2.256369749 | 1.85E-27 | 3.34E-25 |
| CHST4 | 4.272989025 | 1.357407299 | -1.654391936 | 1.61E-23 | 5.61E-22 |
| OR5M1 | 0.000482092 | 2.63E-05 | -4.198259488 | 0.000664705 | 0.001097143 |
| NPHS1 | 0.003197598 | 0.085739225 | 4.744894908 | 1.56E-05 | 3.18E-05 |
| SOX4 | 2.536604913 | 6.665613071 | 1.393838743 | 0.000608735 | 0.001009884 |
| MS4A6E | 0.09259028 | 0.027471873 | -1.752905455 | 0.031569708 | 0.040454783 |
| TERB2 | 0.638809795 | 0.268743397 | -1.249157124 | 1.30E-13 | 7.43E-13 |
| WNT10B | 0.046602635 | 0.112581883 | 1.272491246 | 0.001612286 | 0.002505641 |
| STC2 | 0.417750887 | 1.848874751 | 2.145932697 | 3.19E-16 | 2.63E-15 |
| CSDC2 | 0.340556965 | 0.150981473 | -1.173524616 | 5.99E-11 | 2.38E-10 |
| MAP1B | 0.549123458 | 1.200307246 | 1.128201294 | 1.32E-07 | 3.46E-07 |
| INSRR | 0.004601727 | 0.043514166 | 3.241237848 | 3.28E-05 | 6.42E-05 |
| TLR3 | 2.402606282 | 1.086812767 | -1.144496833 | 1.32E-17 | 1.40E-16 |
| TREH | 2.815017127 | 1.361996702 | -1.04742049 | 3.39E-09 | 1.07E-08 |
| GPRIN1 | 0.145569662 | 0.971860696 | 2.73903982 | 2.22E-21 | 4.52E-20 |
| S100P | 0.889430636 | 55.09210291 | 5.952819625 | 4.29E-05 | 8.27E-05 |
| HMGCLL1 | 0.149498242 | 0.02872533 | -2.379733151 | 1.09E-27 | 2.27E-25 |
| C2orf82 | 2.444581333 | 7.253497015 | 1.569089301 | 0.033777008 | 0.043081053 |
| LAMB4 | 0.009736139 | 0.069101576 | 2.827296973 | 4.20E-11 | 1.71E-10 |
| KCNE1B | 0.024985873 | 0.006826796 | -1.871832021 | 1.96E-10 | 7.29E-10 |
| MLYCD | 3.704860107 | 1.612688088 | -1.199951637 | 6.18E-23 | 1.85E-21 |
| AXDND1 | 0.008207934 | 0.037000205 | 2.172442175 | 2.27E-08 | 6.53E-08 |
| TTYH3 | 5.772848601 | 12.96871931 | 1.167680722 | 3.01E-07 | 7.53E-07 |
| ZBTB32 | 0.051736273 | 0.126931964 | 1.294807386 | 4.35E-08 | 1.21E-07 |
| KLK1 | 0.010719823 | 0.053321603 | 2.314439096 | 0.009955329 | 0.013768837 |
| DACH2 | 0.000693392 | 0.032885292 | 5.567627372 | 1.36E-09 | 4.53E-09 |
| TP53I3 | 3.771035284 | 13.77650372 | 1.869177245 | 2.08E-17 | 2.13E-16 |
| C11orf84 | 1.302286243 | 3.069203843 | 1.236817878 | 8.13E-16 | 6.29E-15 |
| GLYAT | 71.46274637 | 21.34912609 | -1.743014344 | 3.77E-22 | 9.24E-21 |
| NUGGC | 8.398969298 | 3.871432752 | -1.117344712 | 6.78E-16 | 5.32E-15 |
| CD70 | 0.077357628 | 0.289067898 | 1.901792932 | 0.002861299 | 0.004286162 |
| GRIN2C | 0.029324732 | 0.078159899 | 1.414310664 | 3.19E-12 | 1.51E-11 |
| PRR16 | 0.119958992 | 0.317995358 | 1.406464396 | 5.78E-11 | 2.30E-10 |
| SPG20 | 2.989860516 | 1.215833947 | -1.298131976 | 5.83E-19 | 7.56E-18 |
| TRIM71 | 0.002808002 | 0.991587572 | 8.464052226 | 8.46E-14 | 4.95E-13 |
| CDK5R1 | 0.190075975 | 0.485022318 | 1.35147494 | 1.96E-13 | 1.09E-12 |
| MAGEB17 | 0.008233298 | 1.082220906 | 7.03830894 | 2.07E-11 | 8.76E-11 |
| AL049634.2 | 0.004965866 | 0.001674145 | -1.568621014 | 0.000385065 | 0.000656962 |
| ASNS | 0.684024896 | 2.338082597 | 1.773205157 | 5.18E-06 | 1.12E-05 |
| SPP2 | 150.9549924 | 63.3693968 | -1.252260283 | 3.55E-16 | 2.90E-15 |
| TP53AIP1 | 0.005555702 | 0.015829707 | 1.510593437 | 0.009104707 | 0.012666689 |
| NAAA | 11.3914674 | 3.922177366 | -1.538226918 | 1.90E-26 | 1.93E-24 |
| ASPG | 17.58508149 | 4.635870064 | -1.923439985 | 8.48E-21 | 1.55E-19 |
| ABCA8 | 5.794558792 | 2.199749922 | -1.397359295 | 1.38E-18 | 1.68E-17 |
| KDELR3 | 5.897210341 | 12.32781898 | 1.063813025 | 0.000211572 | 0.000372736 |
| RAMP1 | 31.40635677 | 76.27896205 | 1.280228617 | 5.34E-06 | 1.15E-05 |
| GFY | 0.000795487 | 0.027492106 | 5.111034922 | 0.001233071 | 0.001953786 |
| ALPI | 0.012789733 | 4.135005713 | 8.336759399 | 2.49E-05 | 4.94E-05 |
| MCM5 | 2.908097869 | 7.909271043 | 1.443468913 | 2.33E-19 | 3.23E-18 |
| BGLAP | 0.16393762 | 0.616575357 | 1.911130269 | 1.53E-15 | 1.13E-14 |
| INS-IGF2 | 3.969388383 | 0.228823711 | -4.116608268 | 1.18E-27 | 2.42E-25 |
| AMHR2 | 0.265357612 | 0.031996218 | -3.051964662 | 4.27E-27 | 6.16E-25 |
| LCE3D | 0.051614588 | 0.013369142 | -1.948872053 | 0.0067451 | 0.009550443 |
| IGF2BP1 | 0.01267206 | 1.684089026 | 7.054173577 | 1.12E-15 | 8.51E-15 |
| SERPINI1 | 0.782254899 | 3.092837529 | 1.983220355 | 3.57E-12 | 1.68E-11 |
| ZNF714 | 0.064553515 | 0.175992226 | 1.44694414 | 0.000181036 | 0.000321821 |
| WDYHV1 | 1.976318805 | 4.319244375 | 1.127963253 | 5.07E-14 | 3.07E-13 |
| TM4SF20 | 0.014744083 | 4.993752172 | 8.403844285 | 3.76E-05 | 7.30E-05 |
| TMEM26 | 0.42826769 | 0.095054239 | -2.171689966 | 9.44E-27 | 1.07E-24 |
| OR5D18 | 0.000505454 | 0.000150201 | -1.750690072 | 0.00113428 | 0.001809405 |
| SLC7A4 | 0.23323762 | 0.10093255 | -1.208409003 | 3.88E-21 | 7.55E-20 |
| PDE6C | 0.056821873 | 0.126411547 | 1.153609968 | 4.57E-07 | 1.12E-06 |
| CBSL | 1.485118693 | 0.459875828 | -1.691261964 | 3.12E-06 | 6.91E-06 |
| MSANTD1 | 0.015203972 | 0.035864241 | 1.238097844 | 5.97E-10 | 2.09E-09 |
| DUSP6 | 36.35415897 | 13.0532539 | -1.477710935 | 2.50E-21 | 5.04E-20 |
| BIRC5 | 0.373218139 | 6.350794861 | 4.08884616 | 3.21E-27 | 5.14E-25 |
| CYP2F1 | 0.002819812 | 0.009628102 | 1.771652218 | 0.01892498 | 0.025094307 |
| EFHC2 | 0.010841322 | 0.057186236 | 2.399127256 | 0.035406493 | 0.044997077 |
| FCAR | 0.155204333 | 0.03682885 | -2.075260572 | 5.66E-15 | 3.88E-14 |
| PLAU | 1.386866677 | 4.079103911 | 1.556423155 | 6.38E-10 | 2.23E-09 |
| OR1F1 | 0.003577077 | 0.08056851 | 4.493363168 | 0.000578917 | 0.000964324 |
| RPTN | 0.00820273 | 0.000848441 | -3.273218671 | 0.016091802 | 0.021586157 |
| MAPK11 | 1.224032978 | 3.23128066 | 1.400463637 | 1.63E-12 | 8.00E-12 |
| SYT10 | 0.050828073 | 0.005116306 | -3.312451128 | 3.44E-22 | 8.51E-21 |
| RFXANK | 5.453377319 | 11.57040452 | 1.08521742 | 7.64E-20 | 1.15E-18 |
| UBE2Q2L | 0.039482224 | 0.095054193 | 1.267546997 | 0.005936536 | 0.008482478 |
| AURKB | 0.313876026 | 3.804942909 | 3.599608063 | 1.26E-26 | 1.37E-24 |
| TACSTD2 | 5.782232015 | 2.83546345 | -1.028041939 | 4.75E-19 | 6.25E-18 |
| FREM2 | 0.87184231 | 0.175112903 | -2.31578183 | 2.11E-22 | 5.47E-21 |
| FOLR3 | 0.085706847 | 0.033418248 | -1.358774347 | 3.60E-08 | 1.01E-07 |
| ANKLE1 | 0.046908987 | 0.139254621 | 1.569788943 | 6.85E-08 | 1.86E-07 |
| TMEM198 | 0.955086539 | 2.559010528 | 1.421882718 | 2.28E-11 | 9.59E-11 |
| RHBDL2 | 0.092349139 | 0.197425439 | 1.096137488 | 5.34E-05 | 0.000101569 |
| NSMCE2 | 2.732885585 | 7.244887434 | 1.406538213 | 1.04E-23 | 3.82E-22 |
| HIST2H2AB | 0.041459531 | 0.091445056 | 1.141201355 | 0.006221141 | 0.008864073 |
| NDRG1 | 9.833828065 | 24.86652853 | 1.338380079 | 4.46E-05 | 8.58E-05 |
| RNF103-CHMP3 | 0.013714378 | 0.051902716 | 1.920120868 | 5.48E-07 | 1.33E-06 |
| FRMD6 | 2.023624246 | 0.970685753 | -1.059865208 | 1.54E-11 | 6.63E-11 |
| CLCNKA | 0.043608089 | 0.260236342 | 2.577154782 | 2.63E-09 | 8.44E-09 |
| VSX1 | 0.018425387 | 0.090963874 | 2.303598739 | 4.16E-05 | 8.03E-05 |
| BACH2 | 0.840337807 | 0.263572938 | -1.67276714 | 5.77E-16 | 4.56E-15 |
| SOWAHA | 1.114316698 | 4.100978243 | 1.879808772 | 3.11E-12 | 1.47E-11 |
| AKR1B10 | 23.60012563 | 375.6156471 | 3.992390718 | 5.43E-10 | 1.91E-09 |
| ACSL1 | 194.9822782 | 80.46432167 | -1.276921873 | 1.30E-18 | 1.59E-17 |
| SHISA4 | 3.330852195 | 8.729882447 | 1.390070891 | 1.36E-06 | 3.13E-06 |
| DACH1 | 0.552083101 | 0.088035388 | -2.648729966 | 1.15E-28 | 8.00E-26 |
| CDKN2C | 1.113420431 | 5.506490573 | 2.306134683 | 6.96E-24 | 2.68E-22 |
| SPC25 | 0.188098619 | 1.749142516 | 3.217086678 | 4.08E-26 | 3.61E-24 |
| OR2D3 | 0.000618862 | 0.000171639 | -1.850236161 | 0.001704308 | 0.002639684 |
| ATAD3C | 1.810149692 | 0.787729985 | -1.20033591 | 3.65E-11 | 1.49E-10 |
| PTHLH | 0.227238481 | 1.240098084 | 2.448175164 | 0.000714192 | 0.001174318 |
| FGL2 | 8.407027552 | 3.740080527 | -1.168526468 | 1.93E-15 | 1.41E-14 |
| SLC41A2 | 20.35675227 | 8.849514124 | -1.201837259 | 8.48E-21 | 1.55E-19 |
| CCL2 | 16.4015577 | 7.661646389 | -1.09810649 | 5.38E-13 | 2.83E-12 |
| IER2 | 39.91237739 | 18.89351417 | -1.07894515 | 1.61E-15 | 1.19E-14 |
| MRAP2 | 0.10183676 | 1.958513082 | 4.265428428 | 2.37E-13 | 1.31E-12 |
| EEF1A2 | 2.788191658 | 46.9169009 | 4.07270608 | 2.38E-06 | 5.33E-06 |
| ART4 | 7.708606383 | 3.77074678 | -1.031619792 | 1.69E-15 | 1.25E-14 |
| AOC1 | 2.749297349 | 0.729143452 | -1.914788365 | 3.28E-05 | 6.42E-05 |
| TTC9B | 0.024399668 | 0.062655132 | 1.360571196 | 3.53E-06 | 7.76E-06 |
| STXBP5L | 0.000708573 | 0.003901359 | 2.460989062 | 0.001306684 | 0.002061867 |
| ACBD6 | 1.91206629 | 4.052850566 | 1.083804441 | 1.20E-21 | 2.62E-20 |
| PAGE2B | 0.063592658 | 5.646703116 | 6.472404755 | 8.12E-05 | 0.000151042 |
| FABP6 | 0.015029781 | 0.382035448 | 4.667810611 | 0.000115872 | 0.00021159 |
| SQSTM1 | 38.18051384 | 102.8874315 | 1.430158333 | 2.21E-10 | 8.17E-10 |
| CAP2 | 1.731231359 | 8.411804709 | 2.28061682 | 6.73E-23 | 1.98E-21 |
| ZKSCAN3 | 0.589361157 | 1.592604056 | 1.434163751 | 3.16E-20 | 5.18E-19 |
| CIB3 | 0.010021868 | 0.075466666 | 2.912688057 | 0.000631057 | 0.001045133 |
| RBM20 | 0.036389308 | 0.114161022 | 1.649483635 | 2.51E-05 | 4.98E-05 |
| CYP2C8 | 438.1497204 | 101.3685055 | -2.111814452 | 5.40E-26 | 4.42E-24 |
| ACRV1 | 0.013423769 | 0.06024278 | 2.165998569 | 5.71E-11 | 2.28E-10 |
| SEMA6C | 1.20091624 | 3.350514599 | 1.480247163 | 5.31E-12 | 2.43E-11 |
| HIST2H2AC | 1.214115344 | 2.464508088 | 1.021394228 | 4.91E-05 | 9.39E-05 |
| C2CD6 | 0.011576297 | 0.029777826 | 1.363064598 | 0.000126806 | 0.000230449 |
| FZD2 | 0.234495996 | 0.589810892 | 1.330689177 | 0.005506215 | 0.007907116 |
| SLC44A3 | 2.865182631 | 5.90131626 | 1.042409675 | 3.79E-05 | 7.36E-05 |
| DNAJC6 | 0.110751033 | 0.704883232 | 2.670064129 | 3.12E-20 | 5.14E-19 |
| HOXC9 | 0.00783961 | 0.469715072 | 5.904860122 | 4.82E-09 | 1.50E-08 |
| SCRT1 | 0.002306039 | 0.01762017 | 2.933739075 | 5.97E-09 | 1.84E-08 |
| KCNJ6 | 0.003367751 | 0.050094203 | 3.894786113 | 0.001199175 | 0.001903946 |
| SHARPIN | 14.04012639 | 28.10830206 | 1.001440385 | 1.75E-18 | 2.10E-17 |
| ADAMTS13 | 7.411618107 | 1.116995224 | -2.730165529 | 8.69E-30 | 2.52E-26 |
| TMSB15A | 0.066662259 | 0.33856103 | 2.344473817 | 6.96E-09 | 2.13E-08 |
| SLC38A5 | 0.19002809 | 0.550199259 | 1.533741502 | 0.023383674 | 0.030538394 |
| ACAA1 | 59.67082522 | 24.95638653 | -1.257616669 | 8.92E-23 | 2.52E-21 |
| GNG4 | 0.048618302 | 2.093091407 | 5.42799199 | 1.41E-08 | 4.14E-08 |
| PKM | 5.721150894 | 25.51906874 | 2.157198382 | 1.17E-08 | 3.50E-08 |
| GNB1L | 0.407186886 | 0.841977806 | 1.048091106 | 1.01E-11 | 4.48E-11 |
| HIST1H3C | 0.101162744 | 0.328750498 | 1.700315006 | 6.53E-07 | 1.57E-06 |
| FOXD4L1 | 0.011112544 | 0.026582007 | 1.258260872 | 0.000151281 | 0.000271844 |
| CLDN34 | 0.045222796 | 0.020820468 | -1.119047676 | 1.40E-07 | 3.67E-07 |
| NPY1R | 3.065760699 | 0.628557767 | -2.286127845 | 2.58E-26 | 2.47E-24 |
| CELA3A | 0.003114658 | 0.356093507 | 6.83703846 | 0.001901764 | 0.002921659 |
| CHRNA5 | 0.034854628 | 0.120323237 | 1.787493135 | 4.07E-07 | 1.00E-06 |
| MYBL1 | 0.234078794 | 0.572298326 | 1.289773145 | 2.27E-14 | 1.44E-13 |
| LRP8 | 0.052630448 | 0.160895871 | 1.612157721 | 4.53E-08 | 1.25E-07 |
| CCDC170 | 0.279231874 | 1.60997023 | 2.527498474 | 1.26E-07 | 3.30E-07 |
| ODF3L1 | 1.193381934 | 0.449174056 | -1.409709336 | 1.15E-08 | 3.41E-08 |
| ADAM23 | 0.05701883 | 0.566257951 | 3.311949049 | 0.000185065 | 0.000328719 |
| ABCD1 | 3.964212861 | 7.947943485 | 1.003547183 | 2.31E-12 | 1.11E-11 |
| VAMP1 | 0.796233549 | 1.648726017 | 1.050088109 | 2.89E-12 | 1.37E-11 |
| ARHGAP20 | 0.393717788 | 0.152398248 | -1.369315573 | 2.39E-17 | 2.42E-16 |
| TMEM269 | 0.011803036 | 0.043377207 | 1.87777916 | 2.95E-08 | 8.37E-08 |
| KRT12 | 0.003902472 | 0.761584016 | 7.608471268 | 0.000990669 | 0.001594486 |
| TMIGD1 | 0.00213016 | 0.026608082 | 3.64283107 | 0.005640781 | 0.008086921 |
| ADAM12 | 0.069291275 | 0.345356111 | 2.317339138 | 0.000165328 | 0.000295652 |
| S100A3 | 0.135742089 | 0.583827178 | 2.104673249 | 1.45E-16 | 1.27E-15 |
| ACYP1 | 0.663651579 | 1.458610918 | 1.136097176 | 1.64E-19 | 2.32E-18 |
| LDLR | 28.37355408 | 11.77348036 | -1.269006015 | 5.17E-14 | 3.13E-13 |
| MCM6 | 2.190787024 | 7.662018719 | 1.80627531 | 4.78E-23 | 1.46E-21 |
| FNDC9 | 0.001253117 | 0.009377868 | 2.903739117 | 0.001812831 | 0.002794692 |
| ADRA1D | 0.07449409 | 0.419763654 | 2.494379382 | 0.001941798 | 0.002979338 |
| ZIC4 | 0.024050219 | 0.268309535 | 3.479776373 | 0.000428794 | 0.000726154 |
| RUNDC3A | 0.026680964 | 0.100048314 | 1.906814156 | 1.12E-05 | 2.32E-05 |
| IL17D | 0.08537642 | 0.75724755 | 3.148855436 | 4.06E-09 | 1.28E-08 |
| REC114 | 0.008811166 | 0.112475858 | 3.674138634 | 0.032759096 | 0.041878884 |
| C1QTNF1 | 15.58389559 | 6.845018431 | -1.186929586 | 1.66E-21 | 3.50E-20 |
| LRG1 | 334.4754974 | 156.389474 | -1.096757114 | 9.77E-13 | 4.97E-12 |
| SPECC1 | 0.199613954 | 0.627518976 | 1.652446512 | 2.12E-09 | 6.91E-09 |
| NEUROD6 | 0.014024738 | 0.004407475 | -1.669949381 | 1.26E-05 | 2.59E-05 |
| SLC38A2 | 73.41695689 | 30.51214987 | -1.266729482 | 2.95E-17 | 2.93E-16 |
| TNNT2 | 0.020046494 | 0.097591515 | 2.2834058 | 0.001441357 | 0.002259418 |
| DRD4 | 0.24665645 | 1.081983056 | 2.133102988 | 4.37E-07 | 1.07E-06 |
| C1RL | 60.0853271 | 23.68641147 | -1.342953082 | 4.80E-26 | 4.04E-24 |
| BFSP1 | 0.132088496 | 0.400171675 | 1.599114228 | 3.10E-15 | 2.19E-14 |
| TMEM35A | 0.016231947 | 0.057893485 | 1.834564904 | 0.036273509 | 0.04603877 |
| KIF20B | 0.398841597 | 0.808630176 | 1.019664159 | 1.50E-11 | 6.47E-11 |
| MCCD1 | 0.029742301 | 2.284265945 | 6.263070564 | 0.000441815 | 0.000746961 |
| NDRG3 | 3.651989822 | 7.550319039 | 1.047854862 | 2.03E-20 | 3.44E-19 |
| ZNF296 | 0.165327793 | 0.81039901 | 2.293303133 | 8.59E-15 | 5.76E-14 |
| CAPN11 | 0.039382014 | 0.189166646 | 2.264048934 | 1.59E-22 | 4.30E-21 |
| COL5A2 | 3.488095889 | 7.114067656 | 1.028234994 | 0.000999444 | 0.001607832 |
| PIGZ | 0.829320868 | 1.670465165 | 1.010247598 | 2.35E-06 | 5.27E-06 |
| COL11A1 | 0.023689373 | 0.489049024 | 4.367667178 | 0.00269912 | 0.004062785 |
| SPSB2 | 1.418566269 | 3.973130835 | 1.485842754 | 3.34E-20 | 5.43E-19 |
| OR10G9 | 0.000590517 | 0.000158501 | -1.897490783 | 0.001364508 | 0.00214673 |
| CYR61 | 62.90161652 | 20.64869228 | -1.607046678 | 7.95E-19 | 1.00E-17 |
| CCDC85A | 0.022469308 | 0.04724318 | 1.072150419 | 0.00842578 | 0.011775807 |
| TMEM164 | 0.926749795 | 2.3803217 | 1.36090477 | 1.89E-16 | 1.63E-15 |
| LEF1 | 0.144380847 | 1.30856257 | 3.180031634 | 2.63E-16 | 2.19E-15 |
| C3orf35 | 0.070929102 | 0.155528362 | 1.132728102 | 3.57E-10 | 1.29E-09 |
| CELSR3 | 0.041093484 | 0.518757167 | 3.658077804 | 8.93E-26 | 6.71E-24 |
| PIMREG | 0.064457847 | 0.606191338 | 3.23334533 | 6.88E-18 | 7.56E-17 |
| HIST1H2BK | 37.52714839 | 90.44958604 | 1.269179233 | 1.20E-08 | 3.58E-08 |
| KRTAP10-4 | 0.002215994 | 0.017117902 | 2.949479835 | 0.048005789 | 0.059903277 |
| GDF5 | 0.004469178 | 0.016576945 | 1.891096674 | 0.001381095 | 0.002171289 |
| WNT11 | 4.422431825 | 1.697298292 | -1.38159977 | 7.42E-11 | 2.91E-10 |
| POLD1 | 1.776468842 | 4.354502193 | 1.293495414 | 3.00E-22 | 7.53E-21 |
| GDPD3 | 0.28248886 | 0.869517412 | 1.622020944 | 1.96E-12 | 9.50E-12 |
| BCAN | 0.026025385 | 0.709946897 | 4.76971968 | 8.80E-20 | 1.30E-18 |
| RHBDL1 | 0.425027863 | 1.301865472 | 1.61495105 | 7.11E-12 | 3.21E-11 |
| OR6K6 | 0.00055768 | 8.40E-05 | -2.731647653 | 0.001141667 | 0.001820255 |
| TMEM106C | 6.453063543 | 18.78438756 | 1.541477942 | 4.99E-21 | 9.54E-20 |
| LCE2C | 0.010542149 | 0.00297991 | -1.822828487 | 0.00033749 | 0.000579544 |
| PLA2G4F | 0.005934872 | 0.055943739 | 3.236687758 | 0.008425778 | 0.011775807 |
| CLIP2 | 1.145615641 | 2.729263701 | 1.2523887 | 0.001839655 | 0.002831274 |
| RAPSN | 0.085365416 | 0.235969328 | 1.466875736 | 4.33E-09 | 1.36E-08 |
| DLG5 | 0.505205975 | 1.536632004 | 1.604828099 | 7.05E-14 | 4.18E-13 |
| NEIL3 | 0.032414128 | 0.64527205 | 4.31521286 | 4.27E-27 | 6.16E-25 |
| TMEM150B | 1.247030095 | 6.25673062 | 2.326912708 | 7.17E-13 | 3.71E-12 |
| OR2B6 | 0.058339164 | 0.30030255 | 2.363880102 | 1.49E-11 | 6.42E-11 |
| FAM149A | 7.1857431 | 2.918934243 | -1.299695648 | 1.09E-21 | 2.40E-20 |
| IQCD | 0.086333001 | 0.602525106 | 2.803037309 | 1.17E-18 | 1.45E-17 |
| GLB1L3 | 0.004620854 | 0.048291142 | 3.38552729 | 0.008441039 | 0.011795014 |
| L3MBTL1 | 0.084373785 | 0.247185899 | 1.550729715 | 5.14E-19 | 6.72E-18 |
| LCTL | 0.028716783 | 0.067440269 | 1.231716146 | 4.90E-05 | 9.36E-05 |
| SYDE2 | 0.705069833 | 0.303324548 | -1.216903896 | 1.32E-18 | 1.62E-17 |
| NPAP1 | 0.005982411 | 0.00245348 | -1.285897413 | 2.15E-06 | 4.86E-06 |
| SNX15 | 0.048940934 | 0.125683179 | 1.360678034 | 2.39E-16 | 2.01E-15 |
| NMNAT2 | 0.024114612 | 0.132447418 | 2.457440208 | 0.000110466 | 0.000202121 |
| CKMT1A | 0.009204994 | 0.12815087 | 3.799282639 | 8.17E-05 | 0.000151746 |
| PBK | 0.252417635 | 2.771171859 | 3.456611572 | 2.98E-25 | 1.85E-23 |
| ADCY6 | 1.575316008 | 3.592590506 | 1.18938324 | 5.78E-23 | 1.74E-21 |
| ESPL1 | 0.473126845 | 1.223126404 | 1.37027458 | 8.02E-11 | 3.12E-10 |
| ANKRD33 | 0.006649409 | 0.277359683 | 5.382388173 | 0.017333693 | 0.023117237 |
| SP6 | 0.037510123 | 0.353723091 | 3.237268485 | 1.88E-23 | 6.36E-22 |
| GRAPL | 0.038244931 | 0.01338535 | -1.514613636 | 2.16E-09 | 7.02E-09 |
| KRT33A | 0.000824566 | 0.000367896 | -1.164339488 | 0.000452626 | 0.0007641 |
| HMOX1 | 55.35583869 | 26.94517309 | -1.03870864 | 3.45E-14 | 2.13E-13 |
| TNFSF9 | 0.177861563 | 0.512012205 | 1.525423433 | 0.034873653 | 0.044377907 |
| COG2 | 1.1009798 | 2.212167158 | 1.006672405 | 7.29E-23 | 2.12E-21 |
| GOLGA6L10 | 0.021951078 | 0.058139485 | 1.405226467 | 1.66E-09 | 5.47E-09 |
| IGFALS | 71.86496444 | 11.09756789 | -2.695045066 | 5.77E-24 | 2.27E-22 |
| DEFB125 | 0.001468212 | 0.00058651 | -1.323833371 | 0.005327832 | 0.007662267 |
| MAP3K9 | 0.195981988 | 0.504391977 | 1.363824259 | 8.90E-20 | 1.32E-18 |
| FGF17 | 0.015057692 | 0.181796892 | 3.593754929 | 1.97E-08 | 5.72E-08 |
| LIN37 | 0.636616569 | 1.279930526 | 1.007568892 | 6.38E-18 | 7.05E-17 |
| DISP2 | 0.06866831 | 0.222299041 | 1.694785368 | 0.000147949 | 0.000266258 |
| SLCO4C1 | 2.158521338 | 0.792612189 | -1.445356297 | 1.01E-16 | 9.05E-16 |
| MEX3A | 0.296635892 | 1.090598606 | 1.878355143 | 9.95E-14 | 5.77E-13 |
| ZNF670-ZNF695 | 0.009393989 | 0.027064362 | 1.526584628 | 0.000342704 | 0.000588044 |
| COL8A1 | 0.222967916 | 0.735184318 | 1.721269868 | 8.41E-08 | 2.26E-07 |
| DCAF4L2 | 0.003407844 | 3.156491071 | 9.8552468 | 5.91E-05 | 0.00011193 |
| HAPLN3 | 0.339763582 | 0.95358933 | 1.488836868 | 6.37E-09 | 1.96E-08 |
| CD160 | 0.397442536 | 0.130762631 | -1.603795979 | 3.66E-19 | 4.93E-18 |
| CLIC6 | 1.293844324 | 0.532904336 | -1.279715564 | 1.20E-19 | 1.74E-18 |
| PGA4 | 0.000257724 | 0.000108856 | -1.243403832 | 0.006057466 | 0.008645267 |
| OTOGL | 0.005354299 | 0.013842079 | 1.370291156 | 0.008735731 | 0.01218383 |
| CTAG2 | 0.061586861 | 5.491756035 | 6.478501117 | 6.55E-06 | 1.40E-05 |
| KAZN | 0.55973757 | 0.203015998 | -1.463157164 | 3.17E-25 | 1.93E-23 |
| WNT3A | 0.002550378 | 0.771223269 | 8.240293677 | 3.12E-11 | 1.28E-10 |
| RIBC2 | 0.082456297 | 0.701745047 | 3.089245396 | 1.57E-16 | 1.36E-15 |
| C17orf47 | 0.010538619 | 0.02307663 | 1.130746686 | 0.0086105 | 0.012019999 |
| COCH | 0.07243647 | 1.550236177 | 4.419627967 | 5.50E-11 | 2.20E-10 |
| CR1 | 0.307030926 | 0.072050909 | -2.091295449 | 2.08E-18 | 2.48E-17 |
| CCR10 | 0.082296435 | 0.221061003 | 1.425542707 | 1.66E-05 | 3.35E-05 |
| TAZ | 2.556039379 | 5.320584929 | 1.057674797 | 6.30E-22 | 1.46E-20 |
| ZNF169 | 0.086014733 | 0.187243578 | 1.122260541 | 7.41E-17 | 6.77E-16 |
| TAL2 | 0.038063005 | 0.254079936 | 2.738821067 | 8.43E-13 | 4.32E-12 |
| CUZD1 | 0.027584351 | 0.452384563 | 4.035627736 | 6.33E-14 | 3.79E-13 |
| PRRX1 | 0.067522709 | 0.406136579 | 2.588520291 | 1.39E-17 | 1.47E-16 |
| OR5T2 | 0.001812864 | 0.000261239 | -2.794826275 | 0.001442585 | 0.002261106 |
| OR5P2 | 0.000497775 | 6.89E-05 | -2.852764565 | 0.001252532 | 0.001982995 |
| CHD3 | 1.5531158 | 3.126017617 | 1.009160508 | 9.28E-05 | 0.000171212 |
| CHKA | 4.851418043 | 11.12429866 | 1.197235977 | 4.70E-15 | 3.25E-14 |
| RSPH14 | 0.055713061 | 0.179238649 | 1.685794279 | 1.85E-06 | 4.20E-06 |
| HPCA | 0.008584319 | 0.036409039 | 2.084521005 | 5.76E-05 | 0.000109276 |
| FAM222A | 1.183542243 | 3.930242043 | 1.731506964 | 3.31E-16 | 2.72E-15 |
| CDKN3 | 0.312303555 | 4.84681366 | 3.956015721 | 9.94E-28 | 2.12E-25 |
| IL6 | 1.16889639 | 0.296419017 | -1.979437145 | 9.95E-09 | 2.99E-08 |
| MTHFD1 | 46.56509687 | 23.22560534 | -1.003532781 | 2.70E-16 | 2.24E-15 |
| PTK7 | 0.337431587 | 1.62551382 | 2.268228885 | 2.74E-05 | 5.42E-05 |
| PLXNA1 | 0.75442553 | 1.921953445 | 1.349122987 | 4.12E-15 | 2.87E-14 |
| FAM111B | 0.301973238 | 1.935091057 | 2.679908853 | 3.90E-22 | 9.53E-21 |
| KIAA0319 | 0.02074174 | 0.091983469 | 2.148837678 | 0.002308412 | 0.003506226 |
| TIGD7 | 0.162296775 | 0.393907612 | 1.279222963 | 3.48E-15 | 2.45E-14 |
| DBH | 14.92390085 | 1.774662701 | -3.072007929 | 8.37E-27 | 1.02E-24 |
| FAM160A1 | 0.205091978 | 0.087676379 | -1.226010943 | 4.34E-15 | 3.02E-14 |
| ATF3 | 26.44534463 | 8.977805363 | -1.558579052 | 3.98E-17 | 3.84E-16 |
| SMTNL2 | 0.022568531 | 0.089972735 | 1.995175372 | 0.000853238 | 0.001385521 |
| SLC52A3 | 0.183771364 | 0.606006893 | 1.721422226 | 0.020984074 | 0.027613146 |
| DHX34 | 1.858460488 | 4.135592744 | 1.153986106 | 5.96E-23 | 1.79E-21 |
| RPLP1 | 151.5303413 | 346.1814282 | 1.191921633 | 7.57E-21 | 1.40E-19 |
| C10orf142 | 0.11774631 | 0.040146399 | -1.552339372 | 2.34E-20 | 3.94E-19 |
| CLEC4M | 7.841020172 | 0.167976214 | -5.544712509 | 8.56E-30 | 2.52E-26 |
| ERC2 | 0.003498657 | 0.029419805 | 3.071914671 | 3.67E-06 | 8.05E-06 |
| CDH19 | 0.822820374 | 0.20116889 | -2.032170302 | 3.97E-23 | 1.24E-21 |
| PCDHGA1 | 0.023109559 | 0.128278691 | 2.472719896 | 1.26E-07 | 3.30E-07 |
| SYCP3 | 0.028113442 | 0.059666778 | 1.085667804 | 1.44E-07 | 3.75E-07 |
| KCND3 | 5.971263878 | 1.630094749 | -1.873080501 | 2.75E-23 | 8.89E-22 |
| LRRC19 | 0.646985114 | 0.139290425 | -2.215636427 | 2.41E-13 | 1.34E-12 |
| FUT1 | 0.386711602 | 0.783917789 | 1.019444313 | 8.00E-05 | 0.000148917 |
| HTR3B | 0.001167569 | 0.021959043 | 4.233235011 | 0.020908036 | 0.027522398 |
| ZNF57 | 0.67328018 | 1.545132807 | 1.198451945 | 8.39E-11 | 3.26E-10 |
| REG3A | 0.209232559 | 345.1661398 | 10.68796786 | 5.66E-07 | 1.37E-06 |
| TOMM40L | 3.043699949 | 10.17233854 | 1.740753333 | 6.62E-28 | 1.69E-25 |
| VPS45 | 2.066044893 | 4.529935037 | 1.132618758 | 8.11E-29 | 6.01E-26 |
| CACNB3 | 0.18532558 | 0.423761254 | 1.193189653 | 9.22E-13 | 4.70E-12 |
| NPM1 | 41.5079819 | 84.92167241 | 1.032743993 | 6.11E-18 | 6.77E-17 |
| BCAS4 | 0.174928472 | 0.60832874 | 1.798086036 | 2.59E-13 | 1.42E-12 |
| DEFA3 | 0.256135611 | 0.074897744 | -1.773913685 | 2.25E-15 | 1.63E-14 |
| TOP3B | 0.02540658 | 0.054842419 | 1.110090023 | 1.44E-08 | 4.22E-08 |
| KDM8 | 21.02465458 | 4.491915592 | -2.226679375 | 9.34E-24 | 3.45E-22 |
| HAPLN2 | 0.020794983 | 0.042096949 | 1.017480179 | 0.000179714 | 0.000319768 |
| CST1 | 0.079863849 | 18.62507121 | 7.865487627 | 3.96E-06 | 8.66E-06 |
| IGFL4 | 0.003697309 | 0.011055275 | 1.580187443 | 0.002639735 | 0.00398187 |
| GTPBP2 | 3.643319153 | 7.466863835 | 1.035249042 | 6.20E-19 | 8.00E-18 |
| SELE | 1.253477495 | 0.595586829 | -1.073552339 | 1.25E-14 | 8.22E-14 |
| PLIN1 | 6.720083408 | 2.338155299 | -1.523108383 | 4.40E-12 | 2.04E-11 |
| GNGT1 | 0.002303706 | 0.110216895 | 5.580244909 | 0.032658525 | 0.041754534 |
| MMP11 | 0.251121573 | 3.74742316 | 3.899441021 | 3.61E-24 | 1.51E-22 |
| MC1R | 0.147364823 | 0.35800572 | 1.280590453 | 2.34E-09 | 7.56E-09 |
| SLC4A5 | 0.018379333 | 0.038203696 | 1.055627799 | 7.18E-06 | 1.52E-05 |
| IGSF3 | 0.330983354 | 2.083189621 | 2.653963601 | 4.93E-14 | 3.00E-13 |
| MAFG | 1.561976335 | 3.854960232 | 1.303343382 | 8.76E-18 | 9.47E-17 |
| OR13C4 | 0.00050546 | 0.000129244 | -1.967500044 | 0.001536828 | 0.002397711 |
| PNMA1 | 2.49390561 | 4.990408541 | 1.000751064 | 1.45E-08 | 4.26E-08 |
| HIST1H2AI | 0.032100186 | 0.487804952 | 3.925650864 | 1.37E-15 | 1.03E-14 |
| PATE1 | 0.000529077 | 0.000221135 | -1.258548859 | 0.003372698 | 0.004996899 |
| CXCL17 | 0.098572167 | 1.918174841 | 4.282410074 | 0.000423856 | 0.000718418 |
| PLA2R1 | 0.298889749 | 0.136598134 | -1.129675646 | 2.49E-17 | 2.52E-16 |
| CDCA5 | 0.290209925 | 3.226480157 | 3.474792386 | 7.18E-28 | 1.75E-25 |
| ZNF273 | 0.096758257 | 0.199459295 | 1.043637663 | 3.73E-13 | 2.01E-12 |
| SPRR2A | 0.256876035 | 0.050902352 | -2.335268072 | 4.42E-05 | 8.51E-05 |
| HIST1H2AL | 0.010923154 | 0.155422168 | 3.830730836 | 1.89E-11 | 8.06E-11 |
| SF3A2 | 8.24554849 | 16.60553799 | 1.009977095 | 6.99E-18 | 7.67E-17 |
| DGKK | 0.004120719 | 0.065364096 | 3.987530527 | 0.03545853 | 0.045050604 |
| AGL | 10.28271905 | 3.846640214 | -1.418551003 | 1.65E-23 | 5.68E-22 |
| LELP1 | 0.002108031 | 0.000437669 | -2.267983791 | 0.00133607 | 0.002105396 |
| BOLA2B | 0.124677388 | 0.431352963 | 1.790669035 | 2.67E-14 | 1.68E-13 |
| GRIK4 | 0.017473279 | 0.114768436 | 2.715503612 | 0.000245118 | 0.000428825 |
| SH2D4B | 0.00242944 | 0.024287468 | 3.321516123 | 1.05E-12 | 5.31E-12 |
| TMC2 | 0.001262001 | 0.016938792 | 3.746545566 | 8.54E-13 | 4.38E-12 |
| ZGRF1 | 0.086752766 | 0.249498186 | 1.524047667 | 1.04E-21 | 2.30E-20 |
| GLA | 4.709402673 | 10.76649259 | 1.19293235 | 4.66E-17 | 4.44E-16 |
| LPAR4 | 0.003694717 | 0.019233177 | 2.380061155 | 7.42E-10 | 2.56E-09 |
| CGREF1 | 1.692378608 | 6.272362258 | 1.88995653 | 1.07E-08 | 3.22E-08 |
| AP5Z1 | 2.428318521 | 5.298635938 | 1.125663334 | 6.97E-26 | 5.42E-24 |
| ADGRG7 | 5.160332454 | 0.87802743 | -2.555126099 | 6.91E-27 | 8.73E-25 |
| RNASEH2A | 2.643259923 | 9.764992488 | 1.885300636 | 3.24E-24 | 1.39E-22 |
| STK39 | 0.673197262 | 2.811628865 | 2.062304956 | 5.61E-10 | 1.97E-09 |
| GNAZ | 0.250820022 | 3.106059838 | 3.630361198 | 5.70E-18 | 6.36E-17 |
| RCAN1 | 29.95662117 | 6.662312424 | -2.168779995 | 9.07E-27 | 1.06E-24 |
| KLKB1 | 69.69847118 | 27.33561807 | -1.350345015 | 6.23E-22 | 1.45E-20 |
| FAM186A | 0.017819373 | 0.053753946 | 1.592924104 | 2.15E-09 | 7.00E-09 |
| NPEPL1 | 0.57395425 | 1.318545736 | 1.199939967 | 1.23E-19 | 1.78E-18 |
| SSX1 | 0.013900636 | 7.616056373 | 9.097749501 | 3.78E-08 | 1.06E-07 |
| SYT5 | 0.010773484 | 0.069484292 | 2.689201939 | 4.87E-06 | 1.05E-05 |
| FLT3 | 0.229642691 | 0.108211148 | -1.085541727 | 2.41E-13 | 1.34E-12 |
| OR4F3 | 0.000480265 | 8.82E-05 | -2.445051727 | 0.001124143 | 0.001794524 |
| MYRF | 2.557756147 | 5.942210196 | 1.216120913 | 4.04E-08 | 1.13E-07 |
| ZNF239 | 0.233866763 | 0.618947269 | 1.404129665 | 0.038176134 | 0.048307791 |
| TCF19 | 0.739770913 | 5.232217933 | 2.822272151 | 7.99E-26 | 6.10E-24 |
| NMB | 1.049834717 | 4.571931237 | 2.122641494 | 3.33E-19 | 4.50E-18 |
| CTHRC1 | 0.260609611 | 4.648414766 | 4.156776606 | 6.53E-22 | 1.51E-20 |
| GALR3 | 0.118484716 | 0.334109937 | 1.495621926 | 0.009727548 | 0.013473072 |
| KNTC1 | 0.279327933 | 1.101309073 | 1.979187647 | 4.69E-23 | 1.44E-21 |
| PACSIN1 | 0.114328701 | 0.536665629 | 2.230835866 | 0.029680142 | 0.038206491 |
| AACS | 0.47766206 | 0.964209194 | 1.013355897 | 1.99E-14 | 1.27E-13 |
| EHMT2 | 2.842957553 | 7.172999453 | 1.335183962 | 6.14E-25 | 3.37E-23 |
| IFNA8 | 0.00025057 | 2.63E-05 | -3.254164918 | 0.00051079 | 0.000856344 |
| AC012184.2 | 0.001632975 | 0.000572182 | -1.512957243 | 0.000920295 | 0.001488197 |
| GSTA4 | 5.720194114 | 12.39425647 | 1.115535717 | 4.93E-08 | 1.36E-07 |
| ADAM21 | 0.023994281 | 0.085732402 | 1.837149987 | 4.69E-05 | 9.00E-05 |
| PLPP3 | 44.85706555 | 17.3934347 | -1.366792394 | 7.94E-23 | 2.28E-21 |
| NPPA | 0.060354869 | 0.123129629 | 1.028635911 | 0.000847601 | 0.001377518 |
| C21orf62 | 0.119837173 | 0.016616252 | -2.850408562 | 4.46E-24 | 1.80E-22 |
| IGF2BP3 | 0.015525713 | 0.423746887 | 4.770471297 | 2.65E-11 | 1.11E-10 |
| FOXF1 | 1.310012492 | 0.514436377 | -1.348516001 | 9.75E-21 | 1.76E-19 |
| SLC9A3 | 0.071751431 | 1.249660399 | 4.122384669 | 4.00E-07 | 9.89E-07 |
| CKAP4 | 14.98508659 | 34.14440166 | 1.188121632 | 4.47E-15 | 3.10E-14 |
| ILDR2 | 0.110294994 | 0.519008406 | 2.23439059 | 0.000419942 | 0.000712326 |
| KIFC1 | 0.327459353 | 4.64397691 | 3.825973055 | 1.75E-27 | 3.23E-25 |
| ADIG | 0.020184643 | 0.141224068 | 2.806656002 | 0.000917524 | 0.00148418 |
| GBP7 | 22.02317689 | 10.47022034 | -1.072730793 | 9.77E-14 | 5.67E-13 |
| PTPN13 | 0.460422821 | 0.227546564 | -1.016797541 | 6.90E-20 | 1.05E-18 |
| KLHL35 | 0.016642682 | 0.063965697 | 1.942410455 | 0.000152406 | 0.00027368 |
| ATP1B1 | 53.48547489 | 126.3460455 | 1.240161455 | 3.69E-06 | 8.10E-06 |
| WEE1 | 6.908054535 | 3.283141194 | -1.073202677 | 2.48E-11 | 1.04E-10 |
| RMI2 | 0.662581081 | 2.574058488 | 1.957875921 | 2.34E-16 | 1.97E-15 |
| ISM2 | 0.002111634 | 0.061843511 | 4.872190234 | 0.007920428 | 0.011113754 |
| TM4SF5 | 103.715789 | 211.6942903 | 1.029346821 | 0.011244902 | 0.015424029 |
| NDC80 | 0.209421126 | 2.202906711 | 3.394929509 | 6.62E-28 | 1.69E-25 |
| IGDCC3 | 0.003707918 | 0.250738978 | 6.079433329 | 1.83E-08 | 5.32E-08 |
| KIF18B | 0.075803412 | 1.360777563 | 4.166024667 | 6.23E-28 | 1.69E-25 |
| P2RY4 | 0.005625995 | 0.056425611 | 3.326170053 | 0.001619045 | 0.002515388 |
| HIST1H2AB | 0.020455113 | 0.09514348 | 2.217643287 | 9.16E-05 | 0.000169122 |
| RXFP1 | 0.222252345 | 0.067321283 | -1.723064062 | 1.40E-22 | 3.84E-21 |
| TNXB | 1.300101203 | 0.496479478 | -1.388817939 | 8.33E-21 | 1.53E-19 |
| IFT80 | 0.403176862 | 0.823214266 | 1.029855137 | 1.36E-14 | 8.87E-14 |
| CFP | 8.962017943 | 0.927993013 | -3.271637767 | 1.22E-28 | 8.00E-26 |
| ACSBG1 | 0.013161569 | 0.050648022 | 1.944174423 | 0.045317924 | 0.0567314 |
| CCL23 | 2.901698813 | 0.398016067 | -2.865999204 | 2.11E-28 | 1.02E-25 |
| CCR9 | 0.046733203 | 0.018346256 | -1.348962207 | 1.12E-14 | 7.41E-14 |
| AFF2 | 0.004311318 | 0.102140335 | 4.566279813 | 4.25E-05 | 8.19E-05 |
| ANKRD55 | 0.854453521 | 0.192895352 | -2.147183635 | 1.51E-22 | 4.14E-21 |
| HIST1H2AM | 0.101088819 | 0.5978904 | 2.564257608 | 2.64E-15 | 1.89E-14 |
| KRTAP5-5 | 0.00155017 | 0.174684131 | 6.816178493 | 0.004367878 | 0.006360488 |
| BSND | 0.002385406 | 0.040903502 | 4.099917437 | 1.81E-06 | 4.12E-06 |
| OLFML2A | 0.295361989 | 1.619827219 | 2.455283854 | 9.57E-24 | 3.53E-22 |
| EML6 | 0.044439243 | 0.202880323 | 2.190722803 | 4.85E-13 | 2.57E-12 |
| GRAMD1C | 3.428550862 | 1.178729823 | -1.540365849 | 1.44E-23 | 5.09E-22 |
| PPP4R3CP | 0.00130985 | 0.625697948 | 8.899921114 | 0.000763616 | 0.001251088 |
| IQANK1 | 0.181465325 | 0.552517612 | 1.60632655 | 3.86E-05 | 7.49E-05 |
| TNNT3 | 0.026208538 | 0.154775501 | 2.562068325 | 0.008798937 | 0.012266501 |
| ARR3 | 0.029243055 | 0.092326665 | 1.658653349 | 6.84E-12 | 3.09E-11 |
| KIF14 | 0.067812114 | 0.623982378 | 3.201890371 | 1.11E-25 | 8.01E-24 |
| TH | 0.164171358 | 0.040606128 | -2.015433076 | 1.18E-24 | 5.85E-23 |
| LARP1B | 5.802208822 | 2.822970155 | -1.039388343 | 3.72E-23 | 1.17E-21 |
| TEX22 | 0.037341384 | 0.178294013 | 2.25541095 | 3.42E-18 | 3.93E-17 |
| SPATA22 | 0.043617502 | 0.0183088 | -1.252369872 | 0.003335547 | 0.004946318 |
| MICB | 0.658927895 | 1.618773396 | 1.296708535 | 3.71E-11 | 1.52E-10 |
| N4BP2L1 | 10.0719903 | 3.917154214 | -1.362470967 | 2.38E-24 | 1.06E-22 |
| DMBT1 | 0.008860952 | 0.841921075 | 6.570079457 | 0.00012586 | 0.000228806 |
| GABRQ | 0.002212908 | 0.068113797 | 4.943931894 | 1.24E-21 | 2.68E-20 |
| AMH | 0.044549304 | 0.263902002 | 2.566527487 | 3.04E-05 | 5.98E-05 |
| ENO3 | 20.49161475 | 7.148807957 | -1.519259072 | 3.90E-17 | 3.77E-16 |
| BEND2 | 0.004294475 | 0.001047139 | -2.036028981 | 0.000213345 | 0.000375648 |
| IL3RA | 1.617560918 | 3.887758644 | 1.265118612 | 3.65E-18 | 4.19E-17 |
| OR6K3 | 0.001051752 | 0.000449034 | -1.227899335 | 0.006602392 | 0.009360307 |
| SOX6 | 0.973192466 | 0.383609595 | -1.343086345 | 2.75E-15 | 1.96E-14 |
| SLC5A7 | 0.01019468 | 0.005085804 | -1.003268746 | 1.37E-12 | 6.80E-12 |
| PLGLB2 | 4.591375613 | 1.285312981 | -1.836806755 | 1.82E-25 | 1.24E-23 |
| FHDC1 | 0.04983026 | 0.211521921 | 2.085713173 | 3.77E-12 | 1.76E-11 |
| SLC22A4 | 0.240300143 | 0.895975396 | 1.898621606 | 1.58E-07 | 4.10E-07 |
| F2RL3 | 0.252206885 | 1.240447309 | 2.29818089 | 5.99E-13 | 3.13E-12 |
| PLPP4 | 0.719700778 | 0.348043756 | -1.048128524 | 7.03E-23 | 2.06E-21 |
| SMIM24 | 23.13107588 | 9.144421901 | -1.338868498 | 8.73E-09 | 2.64E-08 |
| MYH1 | 0.005713883 | 0.046501829 | 3.024744042 | 0.016102252 | 0.021598313 |
| TMEM74 | 0.203237191 | 0.642521944 | 1.660581296 | 2.79E-06 | 6.20E-06 |
| CDKN2A | 0.214078087 | 3.775275442 | 4.140372875 | 7.94E-24 | 3.01E-22 |
| DEPDC1B | 0.146773086 | 1.465693466 | 3.319924066 | 1.60E-20 | 2.77E-19 |
| ESM1 | 0.223474392 | 4.149939282 | 4.214908805 | 8.51E-25 | 4.47E-23 |
| CAPN9 | 0.032225713 | 0.230587655 | 2.839031079 | 6.35E-09 | 1.96E-08 |
| OR6M1 | 0.001576946 | 0.000585014 | -1.430589848 | 0.00181087 | 0.002791945 |
| GAL3ST1 | 1.029753023 | 6.098519286 | 2.566160639 | 0.018190246 | 0.024186296 |
| MAFF | 9.448606143 | 4.309707383 | -1.132511601 | 7.31E-10 | 2.53E-09 |
| PZP | 17.66167361 | 1.035052283 | -4.092846509 | 9.25E-23 | 2.60E-21 |
| DRD3 | 0.00155881 | 0.000753377 | -1.049000394 | 0.004086686 | 0.005980123 |
| MYLK2 | 0.020293838 | 0.06316517 | 1.638087502 | 1.03E-07 | 2.72E-07 |
| PCDHB11 | 0.086831871 | 0.262840795 | 1.59789263 | 0.012747542 | 0.017339242 |
| CENPL | 0.162310077 | 0.900176421 | 2.471455201 | 2.59E-28 | 1.15E-25 |
| ACACB | 12.2310866 | 5.65422396 | -1.113151644 | 9.29E-18 | 1.00E-16 |
| SYNGR3 | 0.053878518 | 0.222761921 | 2.047720563 | 0.002289615 | 0.003479035 |
| PPP2R3B | 0.513896103 | 1.069372411 | 1.057215746 | 1.38E-16 | 1.21E-15 |
| ERFE | 0.065378627 | 0.426808762 | 2.7066988 | 6.41E-23 | 1.90E-21 |
| OR2B11 | 0.007878537 | 0.002692805 | -1.548818091 | 0.001222794 | 0.001939072 |
| CCDC180 | 0.012117175 | 0.046828819 | 1.950343297 | 9.73E-13 | 4.95E-12 |
| C11orf80 | 0.590997954 | 1.441797777 | 1.286643789 | 1.70E-13 | 9.56E-13 |
| WISP3 | 0.24325986 | 0.096052908 | -1.340597081 | 1.04E-16 | 9.27E-16 |
| ITGB4 | 1.050140842 | 3.262877285 | 1.635561898 | 8.31E-05 | 0.000154178 |
| RAD51AP1 | 0.304867192 | 1.553476027 | 2.349247167 | 2.10E-22 | 5.44E-21 |
| RAB4B-EGLN2 | 0.002377731 | 0.006021286 | 1.340485801 | 0.029129306 | 0.037559568 |
| MTBP | 0.079046144 | 0.316280625 | 2.000438178 | 4.91E-25 | 2.79E-23 |
| GSC | 0.010673711 | 0.076302725 | 2.837672664 | 7.52E-05 | 0.000140434 |
| ABCC9 | 5.851559959 | 2.082388972 | -1.490581706 | 2.94E-18 | 3.42E-17 |
| AC099489.1 | 0.053956755 | 0.1466219 | 1.442225126 | 1.83E-09 | 6.02E-09 |
| KRTAP5-6 | 0.348274498 | 1.09269912 | 1.64959946 | 7.56E-07 | 1.80E-06 |
| SEPT12 | 0.004654693 | 0.011913363 | 1.355822972 | 0.045973516 | 0.057514954 |
| PPP1R3B | 35.34858021 | 13.64272945 | -1.373519962 | 8.17E-17 | 7.41E-16 |
| ALKBH6 | 0.287204931 | 0.690367692 | 1.265284433 | 3.36E-17 | 3.30E-16 |
| TAS2R4 | 0.024018214 | 0.079505805 | 1.726931318 | 1.77E-13 | 9.91E-13 |
| MAGEA8 | 0.002524754 | 0.437288985 | 7.436300339 | 2.21E-05 | 4.40E-05 |
| MAEL | 0.062016779 | 1.108004582 | 4.159161437 | 0.002872812 | 0.004302167 |
| ZNF257 | 0.049110523 | 0.162667594 | 1.72782278 | 0.008486984 | 0.011853892 |
| PSRC1 | 0.607303269 | 1.809292428 | 1.574936563 | 4.24E-15 | 2.95E-14 |
| THBD | 5.606496596 | 2.533658754 | -1.145877309 | 8.59E-16 | 6.62E-15 |
| RAB3B | 0.075787067 | 1.090459083 | 3.846840156 | 3.24E-16 | 2.67E-15 |
| PRIMA1 | 0.169878935 | 0.060506643 | -1.489341517 | 9.12E-22 | 2.05E-20 |
| PDZRN4 | 0.218701167 | 0.040499166 | -2.432996817 | 3.72E-26 | 3.37E-24 |
| SCN5A | 0.005228858 | 0.019616132 | 1.907472726 | 0.011747958 | 0.016062672 |
| IRF8 | 8.059644137 | 2.913190311 | -1.468116188 | 2.40E-23 | 7.86E-22 |
| ANO9 | 0.237622354 | 0.7834309 | 1.721135469 | 0.012038258 | 0.016433402 |
| CRLF2 | 0.02316537 | 0.308723128 | 3.736271958 | 2.81E-05 | 5.54E-05 |
| C17orf78 | 0.008692059 | 0.021237449 | 1.288840651 | 0.011768046 | 0.016088512 |
| E2F7 | 0.024872177 | 0.311229265 | 3.645373002 | 1.88E-24 | 8.77E-23 |
| COL5A3 | 3.488663069 | 8.67218995 | 1.313722087 | 2.30E-05 | 4.59E-05 |
| CNGA1 | 7.591537535 | 3.292008339 | -1.205424118 | 2.31E-15 | 1.67E-14 |
| MCTP1 | 0.204179333 | 0.628839827 | 1.622855751 | 2.37E-06 | 5.31E-06 |
| CD177 | 0.060372896 | 0.379107906 | 2.650635625 | 0.020145276 | 0.026588073 |
| KCNV2 | 0.00287772 | 0.011783731 | 2.033798071 | 0.001005968 | 0.001617726 |
| LGI1 | 0.113331682 | 0.019096333 | -2.569183656 | 8.96E-17 | 8.08E-16 |
| MT1E | 445.9483736 | 98.91412888 | -2.172628187 | 2.20E-23 | 7.26E-22 |
| SGO1 | 0.08960126 | 0.79244992 | 3.144728846 | 4.72E-25 | 2.70E-23 |
| B9D1 | 1.370828223 | 2.748073543 | 1.003372814 | 4.41E-07 | 1.08E-06 |
| SLC35E4 | 0.135260263 | 0.364641557 | 1.430740928 | 2.02E-17 | 2.07E-16 |
| SRPK3 | 0.044049181 | 0.132367708 | 1.587364113 | 5.56E-07 | 1.35E-06 |
| SMARCD3 | 0.640958105 | 1.63547095 | 1.351404167 | 0.000339497 | 0.000582733 |
| ST6GALNAC4 | 1.497442265 | 3.021989558 | 1.012998295 | 1.34E-05 | 2.75E-05 |
| GMNN | 3.391584422 | 16.40176083 | 2.273819396 | 7.02E-26 | 5.43E-24 |
| CIB2 | 0.437041491 | 1.352799536 | 1.630105916 | 3.84E-06 | 8.42E-06 |
| TBXA2R | 3.601202847 | 1.104753939 | -1.70475379 | 7.53E-27 | 9.45E-25 |
| SYNE1 | 1.136856967 | 0.46494993 | -1.289903487 | 1.86E-24 | 8.71E-23 |
| RPL7 | 84.56469742 | 179.3589124 | 1.084722012 | 2.77E-12 | 1.31E-11 |
| PRRG3 | 0.010049711 | 0.055273616 | 2.45943701 | 3.99E-16 | 3.23E-15 |
| NES | 2.743398288 | 7.029242956 | 1.357405232 | 3.43E-14 | 2.12E-13 |
| CAMK4 | 0.266010851 | 0.080373774 | -1.726688366 | 2.33E-24 | 1.05E-22 |
| TYRP1 | 0.012960521 | 0.251942707 | 4.280900098 | 0.005793678 | 0.00828783 |
| EGLN3 | 0.488913439 | 1.41868383 | 1.536902138 | 0.039569729 | 0.049945306 |
| SERTAD1 | 18.04207006 | 6.725876129 | -1.423570762 | 1.19E-16 | 1.06E-15 |
| FAM72C | 0.01137433 | 0.116780909 | 3.359950992 | 4.52E-19 | 5.98E-18 |
| CD164L2 | 0.06204817 | 0.014965169 | -2.051780091 | 0.034083 | 0.043437537 |
| CDC25A | 0.228203804 | 1.03333016 | 2.178906536 | 6.47E-17 | 5.99E-16 |
| SPDYA | 0.035477613 | 0.109037699 | 1.619846166 | 1.97E-17 | 2.02E-16 |
| PRKAA2 | 0.401634445 | 1.50065402 | 1.901636491 | 1.60E-07 | 4.16E-07 |
| TMIE | 0.344435436 | 1.143942695 | 1.7317093 | 0.002327368 | 0.003532949 |
| AC018755.2 | 0.000886692 | 0.000105602 | -3.06980129 | 9.96E-05 | 0.000183081 |
| SLC6A8 | 0.740549137 | 5.900221528 | 2.994101753 | 1.05E-08 | 3.16E-08 |
| MYH4 | 2.298024097 | 8.864688737 | 1.947676049 | 0.005674828 | 0.008131986 |
| INMT-MINDY4 | 0.019489872 | 0.004682538 | -2.057362063 | 5.03E-11 | 2.03E-10 |
| NEB | 0.087471284 | 0.707443221 | 3.015732993 | 2.11E-10 | 7.84E-10 |
| ALPL | 37.75894983 | 14.99252514 | -1.332575248 | 7.30E-20 | 1.10E-18 |
| MYO16 | 0.577597887 | 0.285330476 | -1.017431618 | 6.88E-14 | 4.09E-13 |
| NUDT17 | 0.315491268 | 1.129983427 | 1.840629633 | 8.30E-22 | 1.88E-20 |
| RETNLB | 0.001815853 | 0.048464011 | 4.73819459 | 0.007935055 | 0.011130024 |
| FAM151A | 5.430187366 | 2.422348416 | -1.16459559 | 3.47E-15 | 2.44E-14 |
| DTX1 | 12.55070269 | 3.897047917 | -1.687314564 | 5.77E-19 | 7.49E-18 |
| AVIL | 0.357746838 | 1.565099497 | 2.129243457 | 0.000223678 | 0.000392689 |
| CCL26 | 0.056281067 | 0.548492616 | 3.28475062 | 3.01E-08 | 8.54E-08 |
| SLC7A11 | 0.039068584 | 0.824697532 | 4.399784201 | 9.17E-19 | 1.15E-17 |
| LIN28B | 0.000190929 | 0.477971463 | 11.28967306 | 5.45E-05 | 0.000103666 |
| LDHD | 66.88478606 | 32.17594772 | -1.055695443 | 5.40E-20 | 8.45E-19 |
| BMP4 | 1.155753518 | 3.919762432 | 1.761932465 | 0.010425118 | 0.014382725 |
| ADAM15 | 4.895947313 | 12.75541224 | 1.381449588 | 2.31E-21 | 4.70E-20 |
| HCAR1 | 0.012685672 | 0.057358201 | 2.176799879 | 0.000303831 | 0.000524523 |
| TMEM235 | 0.006619622 | 0.002343179 | -1.498281593 | 0.00012572 | 0.000228579 |
| TMEM145 | 0.02675096 | 0.434433108 | 4.021471481 | 3.92E-17 | 3.78E-16 |
| NPTX1 | 0.005560934 | 0.066036554 | 3.569865723 | 0.000210546 | 0.000371055 |
| C9orf57 | 0.013228295 | 0.212876486 | 4.008317558 | 0.001340626 | 0.002112021 |
| ALDOA | 27.0157005 | 73.25032572 | 1.439037084 | 1.06E-12 | 5.37E-12 |
| R3HDML | 0.017514961 | 0.060355412 | 1.784895363 | 0.015463143 | 0.020797255 |
| LY6K | 0.020458054 | 0.359120015 | 4.133725214 | 0.013149792 | 0.017856721 |
| HSF2BP | 0.043690624 | 0.215368058 | 2.301408674 | 9.63E-20 | 1.42E-18 |
| ALLC | 0.229707329 | 0.100203351 | -1.19686613 | 1.38E-18 | 1.68E-17 |
| IGFL2 | 0.004009933 | 0.036980749 | 3.205124424 | 0.00016944 | 0.000302761 |
| MROH6 | 0.996217444 | 2.446439403 | 1.29615097 | 0.002907581 | 0.004349211 |
| HTR1D | 0.050975203 | 1.070343675 | 4.392134664 | 6.86E-06 | 1.46E-05 |
| UCN2 | 0.003026631 | 0.085933868 | 4.82744232 | 8.66E-11 | 3.36E-10 |
| PSCA | 0.017026621 | 0.396772093 | 4.542446494 | 7.21E-11 | 2.83E-10 |
| MYCN | 0.074865661 | 1.029761261 | 3.781861955 | 8.66E-13 | 4.43E-12 |
| RNF24 | 0.508258767 | 1.052489673 | 1.050170975 | 2.53E-14 | 1.60E-13 |
| FBXL13 | 0.028911725 | 0.059993113 | 1.053142182 | 4.34E-05 | 8.36E-05 |
| B4GALNT1 | 0.052820217 | 1.000402898 | 4.243347098 | 6.41E-20 | 9.83E-19 |
| USH1G | 0.002670189 | 0.035291057 | 3.724288947 | 0.006992411 | 0.009877241 |
| RDH8 | 0.003158798 | 0.164244936 | 5.700329225 | 0.000105829 | 0.000194001 |
| TGIF2LX | 0.002133839 | 0.0848403 | 5.31322664 | 0.026809153 | 0.034759473 |
| DNAH6 | 0.541747782 | 0.264130255 | -1.036371777 | 9.59E-14 | 5.58E-13 |
| RANBP17 | 0.111974521 | 0.2695016 | 1.267123349 | 0.014253526 | 0.019271432 |
| TMEM52B | 0.016505989 | 0.180023396 | 3.447122913 | 0.004875535 | 0.007050244 |
| MT1F | 99.56676148 | 16.49769409 | -2.593399815 | 2.84E-24 | 1.25E-22 |
| APOBEC2 | 0.03952404 | 0.146444104 | 1.889547766 | 5.21E-08 | 1.43E-07 |
| KCNJ11 | 0.292934256 | 0.97986532 | 1.742006553 | 1.60E-06 | 3.67E-06 |
| MNX1 | 0.005068352 | 0.164864064 | 5.023616383 | 0.001497439 | 0.002341896 |
| XRCC3 | 0.32336023 | 1.023162067 | 1.661820525 | 5.20E-26 | 4.32E-24 |
| TMEM191C | 0.022457948 | 0.066783432 | 1.572264128 | 1.20E-08 | 3.56E-08 |
| MRPS21 | 22.09732854 | 45.43379294 | 1.039893786 | 4.28E-20 | 6.78E-19 |
| SAMD11 | 1.342451952 | 0.619287727 | -1.116188694 | 9.64E-16 | 7.38E-15 |
| PADI4 | 0.148393349 | 0.032050704 | -2.210998464 | 3.66E-16 | 2.98E-15 |
| P4HA2 | 1.519616551 | 4.09146195 | 1.428909104 | 2.60E-16 | 2.18E-15 |
| TRIM64B | 0.000502181 | 0.000223158 | -1.170145089 | 0.001589629 | 0.002473393 |
| C21orf58 | 0.148734112 | 0.761424973 | 2.355966323 | 3.05E-25 | 1.89E-23 |
| HIST2H4A | 0.047803154 | 0.231454846 | 2.275553068 | 1.60E-13 | 9.06E-13 |
| TET1 | 0.058127148 | 0.169874689 | 1.54718688 | 4.42E-08 | 1.23E-07 |
| RNASE3 | 0.042252705 | 0.016475822 | -1.358693239 | 1.37E-05 | 2.81E-05 |
| SERPINB8 | 5.030381033 | 1.911893695 | -1.395665374 | 3.21E-14 | 1.99E-13 |
| XKR5 | 0.002295548 | 0.008463409 | 1.882400429 | 0.026431622 | 0.034295335 |
| NTN4 | 7.376887718 | 3.269386973 | -1.173992129 | 1.11E-20 | 1.97E-19 |
| BOC | 0.080257751 | 0.196363508 | 1.290814218 | 0.000163305 | 0.000292172 |
| OPN4 | 3.31E-05 | 0.022518084 | 9.409876138 | 0.000117277 | 0.000213953 |
| PANO1 | 0.101563418 | 0.228295913 | 1.16852418 | 3.60E-08 | 1.01E-07 |
| ACAD11 | 1.043585959 | 0.336322518 | -1.633632159 | 1.17E-20 | 2.08E-19 |
| LOX | 0.525584228 | 1.679260126 | 1.675831839 | 8.94E-08 | 2.39E-07 |
| ERCC6L | 0.035833715 | 0.357756749 | 3.319589439 | 1.01E-25 | 7.45E-24 |
| HGH1 | 8.009567311 | 16.75760667 | 1.065019904 | 1.16E-13 | 6.68E-13 |
| NAT16 | 0.012481809 | 0.085131548 | 2.769866857 | 1.62E-05 | 3.30E-05 |
| GPR146 | 1.18820299 | 0.527547698 | -1.171407878 | 1.53E-15 | 1.14E-14 |
| EDIL3 | 0.170199772 | 1.237103803 | 2.861665548 | 3.45E-21 | 6.80E-20 |
| ZSCAN5C | 0.009262425 | 0.022569955 | 1.284941625 | 0.000124919 | 0.00022723 |
| TRIM45 | 0.179355978 | 0.954115519 | 2.411338123 | 7.88E-25 | 4.21E-23 |
| AKR1C2 | 21.37886686 | 48.47810551 | 1.181147932 | 0.00276517 | 0.004154965 |
| HTR1F | 0.002316535 | 0.016937936 | 2.870217452 | 0.001097419 | 0.001755369 |
| HIST3H2BB | 0.011320695 | 0.106477013 | 3.233507542 | 0.000124608 | 0.000226717 |
| NRBP2 | 10.93208357 | 21.9536623 | 1.005893236 | 3.68E-06 | 8.08E-06 |
| SPON2 | 14.17432654 | 32.13460623 | 1.180847604 | 1.07E-10 | 4.12E-10 |
| NTN5 | 0.041505565 | 0.107991664 | 1.379543257 | 6.76E-06 | 1.44E-05 |
| TSLP | 2.074952522 | 0.426180395 | -2.283542191 | 9.48E-25 | 4.92E-23 |
| MPEG1 | 10.79669446 | 5.308333561 | -1.024258748 | 8.41E-12 | 3.75E-11 |
| PGP | 3.621370573 | 7.793270248 | 1.105693031 | 3.33E-15 | 2.35E-14 |
| CRYGB | 0.002370627 | 0.001077413 | -1.13769674 | 0.002929437 | 0.004379377 |
| DOCK3 | 0.01179956 | 0.075438886 | 2.676575309 | 1.33E-18 | 1.63E-17 |
| ICAM5 | 0.023122287 | 0.140520059 | 2.603420067 | 0.022643637 | 0.02964154 |
| NELFE | 9.699755246 | 25.69549006 | 1.405494918 | 3.39E-23 | 1.07E-21 |
| SERPINE1 | 137.7672457 | 46.5204762 | -1.566295157 | 1.22E-13 | 6.96E-13 |
| MTMR11 | 0.746932617 | 2.818960206 | 1.916113108 | 4.59E-10 | 1.63E-09 |
| DDX53 | 0.00033192 | 0.173827738 | 9.032606956 | 4.26E-05 | 8.21E-05 |
| KIAA0907 | 1.871270031 | 4.761469462 | 1.34738912 | 2.39E-25 | 1.54E-23 |
| BCL2L15 | 0.039758273 | 0.132420479 | 1.735799238 | 9.53E-07 | 2.24E-06 |
| CFAP65 | 0.009400789 | 0.024306111 | 1.370465393 | 0.020277398 | 0.026742033 |
| LAPTM4B | 11.71731476 | 39.31202954 | 1.746328859 | 5.24E-12 | 2.40E-11 |
| SEMA3B | 0.986658486 | 2.902493505 | 1.556670125 | 0.030378723 | 0.039057282 |
| SCIN | 0.019594878 | 0.07299096 | 1.897241191 | 9.99E-06 | 2.08E-05 |
| COL1A1 | 12.13865405 | 55.57723273 | 2.19488554 | 0.000803722 | 0.001311549 |
| GPR19 | 0.030299454 | 0.201444387 | 2.733017898 | 3.73E-20 | 5.99E-19 |
| PCBP4 | 3.136398925 | 6.716586959 | 1.098619241 | 2.59E-16 | 2.17E-15 |
| CDH9 | 0.000309837 | 0.120998778 | 8.609268429 | 0.025044483 | 0.032600273 |
| GADD45G | 95.19206507 | 45.93715224 | -1.051179897 | 2.62E-12 | 1.25E-11 |
| HIST1H3G | 0.010008881 | 0.10513988 | 3.392957364 | 2.87E-05 | 5.66E-05 |
| DLX6 | 0.001652267 | 0.124048343 | 6.230311846 | 0.000612669 | 0.001016303 |
| ZNF775 | 0.842423326 | 2.591869736 | 1.621375922 | 4.77E-18 | 5.39E-17 |
| REXO5 | 0.485940409 | 1.265175037 | 1.380485683 | 8.18E-14 | 4.80E-13 |
| SPINK13 | 0.012730158 | 0.047305701 | 1.893763713 | 0.028546392 | 0.036863075 |
| HAAO | 94.06936089 | 44.92644204 | -1.066160091 | 1.44E-16 | 1.26E-15 |
| TIGD5 | 0.854375167 | 2.517385047 | 1.558984281 | 9.04E-21 | 1.64E-19 |
| AFP | 1.626112602 | 245.8703897 | 7.240327029 | 0.00659081 | 0.009344739 |
| FATE1 | 0.018017209 | 0.466772339 | 4.695271622 | 2.63E-23 | 8.53E-22 |
| SLC4A10 | 0.060496574 | 0.026032549 | -1.21653685 | 2.18E-05 | 4.35E-05 |
| SSC5D | 0.799747035 | 0.369359008 | -1.114519976 | 1.33E-16 | 1.17E-15 |
| PSKH2 | 0.00351163 | 0.001732634 | -1.019173835 | 0.011734499 | 0.016051111 |
| MKRN3 | 0.007956985 | 0.129546157 | 4.025100517 | 6.33E-10 | 2.21E-09 |
| MCM2 | 1.267156446 | 6.967196844 | 2.45898367 | 5.47E-23 | 1.66E-21 |
| TMEM45B | 11.08552854 | 24.25090925 | 1.129361282 | 8.21E-05 | 0.000152472 |
| ACAT1 | 97.49927404 | 47.12172814 | -1.048999027 | 7.82E-21 | 1.44E-19 |
| CHRND | 0.002296964 | 0.166039591 | 6.175655138 | 2.88E-07 | 7.21E-07 |
| FAM46A | 9.928214012 | 4.100989223 | -1.275562262 | 4.98E-17 | 4.73E-16 |
| C6 | 144.9823189 | 49.07089029 | -1.562937618 | 9.08E-23 | 2.56E-21 |
| TMEM56 | 30.66380745 | 13.58241135 | -1.174797214 | 1.76E-21 | 3.69E-20 |
| DLC1 | 6.375629378 | 2.784815604 | -1.194985964 | 9.92E-21 | 1.79E-19 |
| SLITRK3 | 2.238052797 | 0.362965944 | -2.624337976 | 1.30E-18 | 1.59E-17 |
| TNNI3 | 0.01866574 | 0.241079544 | 3.691044607 | 0.00465635 | 0.006753449 |
| C12orf75 | 1.070519697 | 5.426278485 | 2.34165175 | 3.35E-06 | 7.39E-06 |
| TAGAP | 1.160583977 | 0.542318928 | -1.097637488 | 5.83E-13 | 3.05E-12 |
| EZH2 | 0.49651226 | 2.687745128 | 2.436495092 | 4.22E-27 | 6.16E-25 |
| SMC1B | 0.018793151 | 0.269007056 | 3.839365098 | 9.64E-13 | 4.91E-12 |
| OSBPL3 | 0.291320996 | 1.066802767 | 1.872611883 | 5.34E-23 | 1.62E-21 |
| IL1RAPL2 | 0.425715333 | 0.106588705 | -1.997834482 | 2.61E-17 | 2.63E-16 |
| C9orf84 | 0.010970838 | 0.026278196 | 1.260192493 | 0.01146413 | 0.015708907 |
| PRAME | 0.021992502 | 2.747335878 | 6.964877752 | 3.17E-05 | 6.23E-05 |
| RPRML | 0.023585999 | 0.205452361 | 3.122801326 | 4.69E-05 | 9.00E-05 |
| MFSD6 | 0.800238818 | 1.917904919 | 1.261028682 | 1.56E-09 | 5.18E-09 |
| ZNF716 | 0.001486936 | 0.090008071 | 5.919640103 | 0.002130926 | 0.003252521 |
| POLA2 | 1.003802734 | 2.43245308 | 1.276936197 | 1.88E-20 | 3.22E-19 |
| DCST1 | 0.030783675 | 0.157299403 | 2.353275819 | 5.22E-17 | 4.93E-16 |
| DMGDH | 40.55731963 | 15.64201183 | -1.37453623 | 6.84E-21 | 1.28E-19 |
| CD248 | 2.325245238 | 5.330568649 | 1.196906562 | 1.84E-08 | 5.33E-08 |
| MSI1 | 0.215508164 | 2.47500841 | 3.521618997 | 9.21E-05 | 0.000169952 |
| MMP1 | 0.244359902 | 2.174939611 | 3.153895873 | 3.29E-07 | 8.19E-07 |
| PEMT | 64.11861784 | 30.15563183 | -1.088315863 | 8.27E-20 | 1.23E-18 |
| TSPAN10 | 0.261089052 | 0.981448363 | 1.910370402 | 2.58E-07 | 6.53E-07 |
| NCAPG | 0.130896919 | 1.995431055 | 3.930197385 | 1.43E-27 | 2.78E-25 |
| WBP2NL | 0.053006135 | 0.119349593 | 1.170962386 | 4.43E-10 | 1.58E-09 |
| RTL9 | 0.041607475 | 0.182680893 | 2.134411116 | 0.003866277 | 0.005682179 |
| MND1 | 0.210512058 | 1.744062588 | 3.050477038 | 9.22E-26 | 6.90E-24 |
| FOXD1 | 0.006121323 | 0.075616563 | 3.626786933 | 0.005291515 | 0.007612896 |
| LCE3A | 0.060217223 | 0.010714705 | -2.490584081 | 0.000850154 | 0.001381378 |
| COL7A1 | 0.295553103 | 1.879605695 | 2.668940773 | 6.45E-15 | 4.39E-14 |
| TREM2 | 1.604273286 | 4.968343539 | 1.630845009 | 8.94E-10 | 3.05E-09 |
| FEZ1 | 1.842139297 | 0.698970387 | -1.398078918 | 8.14E-21 | 1.50E-19 |
| GBP2 | 5.335533683 | 14.1764546 | 1.409792286 | 4.12E-13 | 2.20E-12 |
| ALDH2 | 169.5306667 | 71.05467094 | -1.254544872 | 3.10E-24 | 1.34E-22 |
| HEATR9 | 0.011358091 | 0.027599258 | 1.280909148 | 0.004114968 | 0.006014726 |
| CALN1 | 0.089616162 | 0.013530702 | -2.727522288 | 2.13E-26 | 2.11E-24 |
| CATSPER2 | 0.071309445 | 0.193104408 | 1.437216017 | 2.23E-16 | 1.89E-15 |
| CKS1B | 4.284509371 | 10.19048801 | 1.250021229 | 1.04E-21 | 2.30E-20 |
| PLA2G4E | 0.00643159 | 0.018253811 | 1.504950448 | 0.00067374 | 0.001110862 |
| SH2D5 | 0.014339133 | 0.063187588 | 2.139683432 | 1.80E-06 | 4.10E-06 |
| OR13A1 | 0.002220208 | 0.043026553 | 4.276460263 | 0.001041693 | 0.001671683 |
| TMEM200A | 0.096746663 | 0.209011547 | 1.111298844 | 0.008471649 | 0.011833589 |
| LHB | 0.032373095 | 0.137923261 | 2.090998572 | 0.019463277 | 0.025768843 |
| PLCXD3 | 0.65766691 | 0.186458265 | -1.818504336 | 6.94E-20 | 1.05E-18 |
| FAM24B | 0.103989605 | 0.507885458 | 2.288063848 | 2.00E-12 | 9.65E-12 |
| OR51E2 | 0.005100724 | 0.049220385 | 3.27048208 | 1.29E-14 | 8.48E-14 |
| TAS2R41 | 0.001364923 | 0.000427765 | -1.673929204 | 0.000584763 | 0.000973332 |
| SLC5A11 | 0.15738882 | 0.819736595 | 2.380827343 | 0.003382544 | 0.005010055 |
| TSGA10IP | 0.01429453 | 0.054344275 | 1.926664879 | 1.74E-07 | 4.51E-07 |
| KPNA2 | 6.494107431 | 19.14594105 | 1.559835416 | 6.70E-20 | 1.02E-18 |
| NRG1 | 1.678151967 | 0.468226534 | -1.84159477 | 4.79E-16 | 3.83E-15 |
| ATP6V1B1 | 0.057682846 | 0.233463477 | 2.016982621 | 5.07E-10 | 1.79E-09 |
| DERL3 | 1.077964228 | 2.504905448 | 1.216446844 | 0.00928617 | 0.012900637 |
| SNPH | 0.095803883 | 0.236328592 | 1.30263815 | 1.27E-06 | 2.94E-06 |
| BHMG1 | 0.005117929 | 0.013644997 | 1.414740046 | 0.013821133 | 0.018724241 |
| ASIC5 | 0.019605996 | 0.068466711 | 1.804107801 | 0.000371154 | 0.00063469 |
| AC013470.2 | 0.002453713 | 0.029209325 | 3.573390868 | 0.007362146 | 0.010367328 |
| SLITRK6 | 0.216143326 | 0.026320986 | -3.037702857 | 4.47E-26 | 3.82E-24 |
| GSTZ1 | 16.49943413 | 4.4551498 | -1.8888707 | 2.20E-24 | 1.00E-22 |
| FBL | 23.7649606 | 48.37817839 | 1.025520438 | 7.21E-14 | 4.27E-13 |
| TEAD2 | 2.536978751 | 7.036457098 | 1.471737769 | 4.03E-10 | 1.45E-09 |
| CRYBA2 | 0.011610802 | 0.325995636 | 4.811313117 | 0.000919365 | 0.001487003 |
| ADAM11 | 0.035817444 | 0.189843335 | 2.406075045 | 1.80E-12 | 8.76E-12 |
| LILRA5 | 2.771819835 | 0.668379366 | -2.052094387 | 7.28E-22 | 1.67E-20 |
| MTHFD2L | 1.919952932 | 0.768145037 | -1.3216203 | 8.01E-17 | 7.28E-16 |
| HOXD3 | 0.001768397 | 0.036360512 | 4.361858193 | 8.21E-16 | 6.35E-15 |
| ZNF736 | 0.204911217 | 0.431301135 | 1.073696551 | 1.75E-11 | 7.47E-11 |
| ZNF793 | 0.049076871 | 0.128060069 | 1.383705514 | 0.000625472 | 0.001036217 |
| C1orf64 | 0.012431949 | 0.488587352 | 5.296492138 | 0.028769479 | 0.037135614 |
| NDOR1 | 1.439057115 | 2.922337625 | 1.021999013 | 3.86E-22 | 9.44E-21 |
| DLX2 | 0.0025644 | 0.077146321 | 4.910904325 | 0.000152777 | 0.00027431 |
| DLK2 | 0.187746482 | 0.787597137 | 2.068671992 | 4.13E-18 | 4.70E-17 |
| ZNF781 | 0.072906408 | 0.241481869 | 1.727797344 | 0.001426128 | 0.002237114 |
| CLIC1 | 40.81917791 | 99.56979791 | 1.286461075 | 3.13E-14 | 1.95E-13 |
| BEST4 | 0.042382137 | 0.228470314 | 2.430478492 | 6.07E-14 | 3.64E-13 |
| HIST1H1E | 0.161124375 | 0.434583312 | 1.431458017 | 1.03E-08 | 3.09E-08 |
| NXF3 | 0.3486915 | 0.042962074 | -3.020815636 | 9.95E-27 | 1.11E-24 |
| HSP90AB1 | 130.0001388 | 382.9730016 | 1.558729527 | 1.43E-24 | 6.95E-23 |
| PIF1 | 0.084205782 | 0.620488731 | 2.881413809 | 5.73E-25 | 3.17E-23 |
| ZNF85 | 0.104218256 | 0.290623232 | 1.479542015 | 7.35E-09 | 2.25E-08 |
| GOLGA6L4 | 0.006697111 | 0.015926123 | 1.249784393 | 0.001420074 | 0.002228067 |
| DDX39B | 3.031879721 | 6.420553095 | 1.082485062 | 1.06E-12 | 5.35E-12 |
| FAM212B | 0.196322555 | 0.484323024 | 1.30274366 | 4.63E-17 | 4.42E-16 |
| WSCD1 | 0.182418549 | 0.507075932 | 1.474949361 | 8.55E-05 | 0.000158348 |
| ADM2 | 0.890901457 | 4.040408695 | 2.181163463 | 4.82E-13 | 2.56E-12 |
| FRMD3 | 0.226657336 | 0.854737893 | 1.914969228 | 0.000179274 | 0.000319051 |
| FDPS | 28.63419799 | 58.34327035 | 1.026827061 | 1.59E-10 | 5.99E-10 |
| H2AFB3 | 0.000851896 | 0.000152006 | -2.486547024 | 0.001549406 | 0.002415639 |
| C10orf95 | 0.04504067 | 0.117592497 | 1.384495809 | 1.09E-10 | 4.17E-10 |
| DEFB124 | 0.002612741 | 0.019129462 | 2.872160009 | 0.044006069 | 0.055195617 |
| WNT2B | 0.048511802 | 0.136036218 | 1.48758312 | 8.49E-11 | 3.30E-10 |
| ANKS3 | 0.687655963 | 1.447328637 | 1.073633679 | 3.01E-17 | 2.98E-16 |
| PITX1 | 0.027167265 | 1.506517209 | 5.793204022 | 1.25E-14 | 8.19E-14 |
| NFATC4 | 0.326374882 | 0.833737222 | 1.353062714 | 1.77E-07 | 4.58E-07 |
| CTRC | 0.004694694 | 0.024337022 | 2.374049683 | 9.86E-05 | 0.000181321 |
| CDCA4 | 0.978770444 | 2.612784284 | 1.416545576 | 1.85E-22 | 4.91E-21 |
| AGBL4 | 0.151350109 | 0.036723546 | -2.043112457 | 4.51E-16 | 3.62E-15 |
| ID1 | 54.75570659 | 19.39696264 | -1.49717857 | 6.41E-19 | 8.25E-18 |
| AC036214.3 | 0.038789841 | 0.081035931 | 1.062882854 | 8.68E-07 | 2.05E-06 |
| COL6A6 | 0.218677824 | 0.021304623 | -3.359568492 | 1.72E-28 | 9.21E-26 |
| PLCB1 | 0.343762538 | 1.000961031 | 1.541901569 | 3.61E-10 | 1.30E-09 |
| PANK1 | 15.31986367 | 5.623809473 | -1.445783835 | 3.56E-23 | 1.12E-21 |
| MKI67 | 0.229534036 | 2.522416913 | 3.45802675 | 3.06E-26 | 2.86E-24 |
| CLDN7 | 8.825675471 | 18.3891125 | 1.05907325 | 0.00307498 | 0.004580689 |
| PLA2G4C | 1.417021128 | 3.333079616 | 1.23399451 | 6.95E-10 | 2.41E-09 |
| PGLYRP1 | 0.242868805 | 0.061361442 | -1.984772896 | 7.96E-14 | 4.68E-13 |
| ANXA8 | 0.19498575 | 0.086796228 | -1.167664443 | 3.40E-19 | 4.60E-18 |
| PPP1R16A | 8.209144877 | 17.26276956 | 1.072360089 | 5.54E-12 | 2.53E-11 |
| ZBTB12 | 0.460849883 | 1.718303639 | 1.898616207 | 4.29E-21 | 8.28E-20 |
| WDR76 | 0.33341526 | 1.608894949 | 2.27067809 | 2.56E-22 | 6.54E-21 |
| RIMBP3 | 0.005564167 | 0.012582053 | 1.177129569 | 0.0035049 | 0.005182403 |
| APOBEC3B | 0.561285288 | 2.107510501 | 1.90873367 | 5.99E-09 | 1.85E-08 |
| CCDC102B | 0.223197266 | 0.576813185 | 1.369784789 | 3.98E-19 | 5.33E-18 |
| STRA6 | 0.007516895 | 0.108536204 | 3.851895766 | 2.02E-10 | 7.52E-10 |
| LAT | 0.034242217 | 0.075795283 | 1.146331952 | 1.13E-11 | 4.94E-11 |
| TRIM59 | 0.066456325 | 0.276830087 | 2.05852234 | 3.00E-22 | 7.53E-21 |
| NCR1 | 0.198459964 | 0.098567429 | -1.009665106 | 2.95E-15 | 2.09E-14 |
| UPK1A | 0.048319028 | 0.393323316 | 3.025052359 | 1.21E-05 | 2.49E-05 |
| DNAJB13 | 0.010433775 | 0.04453625 | 2.093718821 | 9.78E-07 | 2.30E-06 |
| MYO15B | 1.52310404 | 4.089616224 | 1.424950972 | 4.11E-12 | 1.91E-11 |
| SQLE | 6.738818984 | 22.82406894 | 1.759988332 | 1.26E-13 | 7.21E-13 |
| AC138696.1 | 0.010462785 | 0.035391925 | 1.758153266 | 2.34E-05 | 4.65E-05 |
| NEURL3 | 0.855145792 | 2.671840257 | 1.643591447 | 0.012164583 | 0.016598543 |
| MELTF | 0.404639476 | 1.336902851 | 1.724185654 | 0.02319676 | 0.03031459 |
| TRIM16 | 0.410993777 | 3.066212782 | 2.899269362 | 1.33E-15 | 9.99E-15 |
| MFGE8 | 1.63926953 | 3.867071971 | 1.238188532 | 2.35E-11 | 9.86E-11 |
| TONSL | 0.593455086 | 2.233639146 | 1.912185378 | 1.93E-24 | 8.94E-23 |
| GNA14 | 2.351303871 | 0.715778328 | -1.71587623 | 4.21E-26 | 3.70E-24 |
| PCLAF | 0.444424411 | 2.469352853 | 2.474123035 | 2.04E-22 | 5.31E-21 |
| AC009014.1 | 0.124707721 | 3.083029758 | 4.627726115 | 0.010972156 | 0.015075934 |
| FGF13 | 0.077422295 | 0.317774333 | 2.037181617 | 6.22E-06 | 1.33E-05 |
| FBP1 | 387.4666189 | 137.2432586 | -1.497336739 | 2.62E-21 | 5.25E-20 |
| EIF4E3 | 1.88441544 | 0.857617239 | -1.135711246 | 4.54E-17 | 4.34E-16 |
| GPD1 | 46.05699046 | 17.92752399 | -1.361243895 | 8.30E-17 | 7.51E-16 |
| RSPO3 | 2.06299047 | 0.694743492 | -1.570184836 | 2.62E-28 | 1.15E-25 |
| KLRD1 | 0.226577163 | 0.097552759 | -1.215747876 | 6.45E-18 | 7.11E-17 |
| PCSK2 | 0.043154616 | 0.005767974 | -2.903378426 | 6.67E-21 | 1.25E-19 |
| PABPC1 | 75.11466513 | 192.4965579 | 1.357666141 | 1.06E-15 | 8.08E-15 |
| SRRM4 | 0.000969366 | 0.005693661 | 2.55424373 | 0.009023222 | 0.012561361 |
| PLP1 | 0.078357541 | 0.021168042 | -1.888184279 | 1.83E-20 | 3.14E-19 |
| CDX2 | 0.041344052 | 0.089800295 | 1.119040385 | 9.40E-05 | 0.000173196 |
| SLC7A8 | 2.822367188 | 1.112743676 | -1.342784392 | 4.09E-20 | 6.52E-19 |
| ABCA12 | 0.00571057 | 0.034511622 | 2.595375633 | 6.75E-05 | 0.000126745 |
| CDA | 37.22510302 | 17.0832809 | -1.123690766 | 4.84E-16 | 3.86E-15 |
| SCAMP3 | 16.3914446 | 42.94595738 | 1.389579326 | 4.11E-28 | 1.33E-25 |
| TMEM262 | 0.076465801 | 0.19199672 | 1.328195113 | 3.31E-19 | 4.48E-18 |
| INHA | 0.047643739 | 1.28080619 | 4.748621737 | 3.28E-05 | 6.42E-05 |
| BAIAP2L2 | 0.78326013 | 6.325329204 | 3.01357714 | 2.12E-15 | 1.54E-14 |
| SYTL5 | 1.063309689 | 0.35406671 | -1.586468732 | 2.86E-21 | 5.70E-20 |
| CYP2B6 | 137.9677309 | 29.07521914 | -2.246468906 | 3.86E-22 | 9.44E-21 |
| ZPBP | 0.012261749 | 0.001725416 | -2.829148451 | 2.63E-14 | 1.65E-13 |
| CDC7 | 0.27687099 | 1.217314207 | 2.136415793 | 3.14E-24 | 1.35E-22 |
| TMEM225 | 0.001289906 | 0.00061767 | -1.062357111 | 0.00380535 | 0.005597394 |
| EFNA4 | 2.652357584 | 7.789826457 | 1.554315899 | 3.66E-21 | 7.17E-20 |
| MATN3 | 0.03083333 | 1.36770814 | 5.471125887 | 1.45E-13 | 8.23E-13 |
| ACADS | 85.50530186 | 31.69924738 | -1.431565291 | 5.55E-25 | 3.09E-23 |
| VSTM5 | 0.009427007 | 0.031747217 | 1.751758498 | 1.09E-06 | 2.56E-06 |
| TKT | 10.63539204 | 31.46046918 | 1.564666971 | 1.07E-11 | 4.69E-11 |
| PCDHA1 | 0.005099765 | 0.146562749 | 4.844943855 | 0.000443944 | 0.000750181 |
| SGIP1 | 0.058632302 | 0.147474849 | 1.330701314 | 0.000495845 | 0.000833088 |
| LOXHD1 | 0.003510458 | 0.02268017 | 2.691700345 | 8.78E-12 | 3.91E-11 |
| PGGHG | 3.596174972 | 11.79449107 | 1.713578046 | 2.98E-05 | 5.87E-05 |
| FIGNL1 | 0.652114049 | 1.391435418 | 1.093377744 | 1.97E-15 | 1.43E-14 |
| OR9G4 | 0.000339806 | 5.60E-05 | -2.600535282 | 0.00076029 | 0.001246033 |
| PHOSPHO1 | 0.199600051 | 0.089974082 | -1.149530702 | 7.72E-17 | 7.03E-16 |
| KLF13 | 3.097341262 | 6.632898871 | 1.098609183 | 1.17E-13 | 6.70E-13 |
| ANKRD63 | 0.004772078 | 0.002376494 | -1.005782771 | 0.016605446 | 0.02222918 |
| MS4A3 | 0.014705068 | 0.002926719 | -2.328957295 | 1.64E-10 | 6.14E-10 |
| MFRP | 0.00028457 | 2.63E-05 | -3.43773157 | 0.001173817 | 0.001867878 |
| CDC25C | 0.067998116 | 1.558161892 | 4.518206544 | 8.34E-28 | 1.91E-25 |
| RHBDL3 | 0.022997933 | 0.428453521 | 4.219562586 | 8.27E-05 | 0.000153482 |
| ZBED6CL | 3.265291011 | 7.163714833 | 1.133496335 | 1.44E-09 | 4.78E-09 |
| FAT2 | 0.007829223 | 0.018384366 | 1.231538446 | 9.83E-05 | 0.000180885 |
| TERT | 0.002629626 | 1.379407668 | 9.034975764 | 1.15E-24 | 5.76E-23 |
| MAN1C1 | 6.091134398 | 2.026018156 | -1.588063834 | 5.18E-23 | 1.58E-21 |
| SLC6A7 | 0.004647083 | 0.05553977 | 3.579124028 | 6.60E-07 | 1.58E-06 |
| HSF4 | 0.697518684 | 2.410430282 | 1.788986935 | 2.94E-18 | 3.42E-17 |
| NNAT | 0.082966795 | 0.183582473 | 1.145822358 | 0.000190104 | 0.000337244 |
| POLE2 | 0.504267438 | 1.546718891 | 1.616950043 | 1.75E-16 | 1.51E-15 |
| CSMD1 | 0.002852304 | 0.147839319 | 5.695758417 | 4.89E-08 | 1.35E-07 |
| CCL3 | 6.853998354 | 2.389974016 | -1.519950912 | 1.17E-15 | 8.87E-15 |
| TRIM77 | 0.00280553 | 0.000607039 | -2.208411845 | 0.000184973 | 0.00032863 |
| AC135586.2 | 0.696500608 | 0.344131169 | -1.017166044 | 2.68E-12 | 1.27E-11 |
| PIP | 0.008143184 | 0.154347775 | 4.244447879 | 0.029714735 | 0.038247857 |
| CFHR3 | 68.29533372 | 27.70955583 | -1.301403424 | 4.45E-11 | 1.80E-10 |
| TPBGL | 0.051347497 | 0.439573695 | 3.097739196 | 4.51E-11 | 1.82E-10 |
| PGF | 0.652128092 | 1.488356993 | 1.190493335 | 8.96E-07 | 2.12E-06 |
| VCX | 0.002170619 | 0.296143363 | 7.092045523 | 1.06E-05 | 2.20E-05 |
| HOXA13 | 0.010253804 | 1.300875117 | 6.987179445 | 3.12E-20 | 5.14E-19 |
| ZNF703 | 1.91718753 | 5.021866712 | 1.389232277 | 0.001168553 | 0.001860072 |
| CAMP | 0.121755034 | 0.051051596 | -1.253953453 | 7.57E-11 | 2.96E-10 |
| ZFP82 | 0.130926088 | 0.266135153 | 1.023406486 | 0.015701123 | 0.021093003 |
| P2RY13 | 2.33194473 | 0.631818584 | -1.883951317 | 6.33E-20 | 9.74E-19 |
| MAGEB5 | 0.002232632 | 0.0002606 | -3.09883461 | 0.001583383 | 0.002464412 |
| GRP | 0.004134003 | 0.10533598 | 4.671314958 | 0.019398739 | 0.025685581 |
| TNFSF4 | 0.27235337 | 0.960582082 | 1.818429179 | 1.56E-14 | 1.01E-13 |
| VAX2 | 0.040500304 | 0.441731124 | 3.447163835 | 6.74E-15 | 4.58E-14 |
| MSX1 | 0.279042079 | 1.180933684 | 2.081373352 | 1.34E-16 | 1.18E-15 |
| SPINK4 | 0.089175556 | 0.520916747 | 2.546332603 | 0.003724638 | 0.005487714 |
| HBG1 | 0.009614368 | 0.00176964 | -2.441736365 | 0.000344712 | 0.000591359 |
| RTEL1-TNFRSF6B | 0.064496817 | 0.154357104 | 1.258972013 | 2.58E-12 | 1.23E-11 |
| MYBPC2 | 0.010493007 | 0.063421023 | 2.595533025 | 0.003264068 | 0.004845183 |
| ZNF468 | 0.716025223 | 1.473835588 | 1.041493282 | 0.000135302 | 0.000244884 |
| TSPAN15 | 2.677441605 | 7.258385658 | 1.438793603 | 0.000314815 | 0.000542582 |
| STK32B | 0.044175341 | 0.125354696 | 1.504702867 | 7.46E-07 | 1.78E-06 |
| RHOXF1 | 0.050627191 | 0.146861732 | 1.536474168 | 0.00268821 | 0.004047583 |
| PFKFB4 | 0.212896592 | 0.700419438 | 1.718066263 | 3.48E-12 | 1.64E-11 |
| TTF2 | 0.305701701 | 0.626854215 | 1.036005379 | 1.20E-15 | 9.07E-15 |
| ABCB5 | 0.002306092 | 0.174258526 | 6.239635198 | 6.26E-09 | 1.93E-08 |
| FGF12 | 0.145308541 | 0.610504522 | 2.070882473 | 1.99E-06 | 4.49E-06 |
| STRA8 | 0.012589933 | 0.251872232 | 4.322349583 | 4.25E-10 | 1.52E-09 |
| CACNG3 | 0.005500605 | 0.0020902 | -1.395949301 | 9.48E-07 | 2.23E-06 |
| BLVRA | 4.655434142 | 11.33869066 | 1.284266436 | 2.38E-09 | 7.70E-09 |
| FAM171B | 0.121617454 | 0.291253599 | 1.259925589 | 1.22E-09 | 4.11E-09 |
| DRC1 | 0.011337695 | 0.067519274 | 2.57417201 | 3.55E-11 | 1.45E-10 |
| ENO1 | 118.0925678 | 253.4190534 | 1.101606827 | 1.75E-08 | 5.10E-08 |
| AC120114.5 | 0.029452415 | 0.059183968 | 1.00682049 | 0.002040659 | 0.003119964 |
| PAQR4 | 0.598102122 | 2.428057667 | 2.021338946 | 7.65E-19 | 9.71E-18 |
| HIST1H4K | 0.168435466 | 0.634467229 | 1.913349703 | 2.19E-09 | 7.12E-09 |
| CCDC138 | 0.276230339 | 0.553465254 | 1.002620964 | 3.33E-10 | 1.20E-09 |
| PABPC1L | 1.353508119 | 4.500252601 | 1.733302442 | 5.42E-17 | 5.08E-16 |
| JAKMIP3 | 0.020889082 | 0.072721639 | 1.799635631 | 0.001262678 | 0.001997635 |
| C1QL1 | 0.430759955 | 6.915281139 | 4.004831861 | 7.23E-07 | 1.73E-06 |
| LOXL2 | 0.841983361 | 2.705730215 | 1.684154368 | 1.92E-22 | 5.05E-21 |
| AC135050.2 | 0.073747303 | 0.028699149 | -1.361582345 | 5.03E-10 | 1.78E-09 |
| AC005324.3 | 0.003716033 | 0.026442543 | 2.831025915 | 7.13E-05 | 0.000133409 |
| TMPRSS4-AS1 | 0.037738186 | 0.007345697 | -2.36105378 | 4.99E-05 | 9.53E-05 |
| GSG2 | 0.03965132 | 0.292747409 | 2.884215613 | 1.74E-24 | 8.25E-23 |
| BNIPL | 0.088507795 | 0.310802947 | 1.812123764 | 3.42E-13 | 1.85E-12 |
| FAM46C | 3.920754314 | 1.834099116 | -1.096059636 | 9.11E-17 | 8.21E-16 |
| SAXO1 | 0.020013124 | 0.051592589 | 1.366217421 | 0.00187832 | 0.002887638 |
| CNFN | 0.414398102 | 1.647977262 | 1.991607037 | 4.21E-06 | 9.17E-06 |
| PLEKHH1 | 0.14465866 | 0.401476557 | 1.472663054 | 1.88E-15 | 1.38E-14 |
| TBX20 | 0.107066019 | 0.012721658 | -3.073142095 | 9.97E-21 | 1.79E-19 |
| SORT1 | 2.506877151 | 6.005795255 | 1.260463994 | 2.68E-12 | 1.27E-11 |
| PRIM1 | 1.400915621 | 3.439489868 | 1.295824543 | 8.47E-15 | 5.69E-14 |
| VSIG10L | 0.595829129 | 2.50344799 | 2.070945923 | 2.04E-05 | 4.08E-05 |
| PRR36 | 0.09396757 | 0.493502826 | 2.392823505 | 9.99E-11 | 3.85E-10 |
| NOV | 0.467148807 | 1.031414472 | 1.142670106 | 7.24E-05 | 0.000135344 |
| CCNE2 | 0.113393449 | 0.505743002 | 2.157067156 | 3.71E-20 | 5.97E-19 |
| PSPH | 2.946011368 | 8.477023819 | 1.524794843 | 9.96E-18 | 1.07E-16 |
| SOCS3 | 47.76561533 | 12.71349958 | -1.909611241 | 7.50E-15 | 5.06E-14 |
| AC233992.2 | 0.016253256 | 0.071678019 | 2.140802055 | 1.17E-09 | 3.95E-09 |
| PHYHD1 | 14.63643135 | 6.491426859 | -1.172956307 | 9.55E-17 | 8.58E-16 |
| MFSD2A | 44.73760261 | 10.73240435 | -2.059514633 | 7.06E-20 | 1.07E-18 |
| DNAJC5G | 0.006950296 | 0.039901017 | 2.521279274 | 0.000563526 | 0.000939908 |
| SVEP1 | 1.134809714 | 0.398845786 | -1.508547463 | 1.15E-19 | 1.68E-18 |
| SPEF2 | 0.126060448 | 0.264055653 | 1.06672633 | 6.64E-09 | 2.04E-08 |
| KLF10 | 27.93778758 | 12.00208637 | -1.21893256 | 1.09E-17 | 1.16E-16 |
| PNPLA5 | 0.144984429 | 0.045603295 | -1.668687989 | 0.003880432 | 0.005700827 |
| FXYD1 | 29.0001294 | 8.866142804 | -1.709680833 | 1.40E-20 | 2.45E-19 |
| FIBCD1 | 0.00367406 | 0.165932686 | 5.497079181 | 2.90E-13 | 1.58E-12 |
| NETO2 | 0.080449747 | 0.431593236 | 2.423512473 | 1.22E-23 | 4.41E-22 |
| RAB6B | 0.23893737 | 0.687396363 | 1.524509707 | 3.21E-20 | 5.25E-19 |
| TMEM184B | 3.606858303 | 7.423835163 | 1.04142193 | 8.38E-21 | 1.54E-19 |
| ARHGAP11B | 0.041879726 | 0.202958538 | 2.276861135 | 4.52E-24 | 1.82E-22 |
| CITED1 | 0.058073743 | 0.146815506 | 1.338046421 | 3.88E-07 | 9.59E-07 |
| FSIP2 | 0.009265076 | 0.026777466 | 1.531144714 | 0.043307087 | 0.054367115 |
| FAM171A2 | 0.086964114 | 0.391862947 | 2.171857074 | 3.16E-14 | 1.97E-13 |
| DTNA | 0.410097772 | 1.4432147 | 1.815246127 | 1.43E-11 | 6.20E-11 |
| ADAMTS6 | 0.036728442 | 0.09307746 | 1.34153416 | 1.18E-06 | 2.75E-06 |
| CALML6 | 0.062100496 | 0.251226205 | 2.016310265 | 4.12E-10 | 1.47E-09 |
| VGF | 0.109052955 | 0.442706256 | 2.021320894 | 1.37E-05 | 2.79E-05 |
| AVPR1A | 17.56857907 | 3.767731117 | -2.221229596 | 1.70E-17 | 1.77E-16 |
| CEP55 | 0.137978963 | 1.253780265 | 3.183764299 | 1.37E-25 | 9.65E-24 |
| PDE7B | 1.404436251 | 0.456170466 | -1.622346191 | 2.02E-22 | 5.28E-21 |
| CLEC4G | 37.96628673 | 1.058108176 | -5.165159869 | 9.72E-30 | 2.52E-26 |
| SPATA25 | 0.567756556 | 1.262033449 | 1.152405783 | 4.85E-09 | 1.51E-08 |
| NSD2 | 0.781547354 | 1.697732246 | 1.119203752 | 1.35E-21 | 2.90E-20 |
| MRO | 1.836189524 | 0.385172052 | -2.253140046 | 1.16E-24 | 5.78E-23 |
| RNF157 | 0.374864495 | 2.23518111 | 2.575950639 | 1.83E-12 | 8.91E-12 |
| USP21 | 1.818674153 | 4.123035056 | 1.180819645 | 3.43E-25 | 2.03E-23 |
| PNPLA7 | 2.578738886 | 1.036158514 | -1.315420972 | 7.82E-21 | 1.44E-19 |
| TTC39A | 0.144850741 | 1.30633479 | 3.172885713 | 1.65E-15 | 1.22E-14 |
| DLL3 | 0.002862597 | 0.058578762 | 4.354981359 | 0.000137334 | 0.000248302 |
| RPS20 | 125.8478241 | 278.3549823 | 1.145245634 | 3.23E-15 | 2.28E-14 |
| CDK3 | 0.146960306 | 0.327155332 | 1.154549246 | 9.35E-09 | 2.82E-08 |
| NPPB | 0.045246815 | 0.19614627 | 2.116041749 | 0.010742069 | 0.014780677 |
| S100A11 | 43.94376249 | 151.474437 | 1.785344039 | 0.029358657 | 0.03783356 |
| HTR4 | 0.015040328 | 0.05846259 | 1.958677678 | 0.00097997 | 0.00157879 |
| SMKR1 | 0.041622666 | 0.346681673 | 3.058170303 | 2.16E-08 | 6.23E-08 |
| PLPP7 | 0.113674443 | 0.352871707 | 1.634235828 | 0.000418967 | 0.000710904 |
| STIP1 | 11.95157314 | 24.46789537 | 1.033689485 | 3.34E-22 | 8.28E-21 |
| C21orf91 | 2.934228573 | 0.946891634 | -1.631710027 | 2.33E-26 | 2.28E-24 |
| NAA40 | 1.013116467 | 2.166404105 | 1.096502342 | 2.60E-23 | 8.44E-22 |
| WDR97 | 0.075539327 | 0.253736079 | 1.748028838 | 6.98E-14 | 4.14E-13 |
| PTGDR2 | 0.28562167 | 1.110830269 | 1.959461051 | 6.35E-06 | 1.35E-05 |
| SLC8A2 | 0.00700052 | 0.029314453 | 2.066078147 | 0.001355064 | 0.002132734 |
| PKDREJ | 0.026987097 | 0.05422652 | 1.006728788 | 0.025894796 | 0.033645532 |
| MPPED1 | 4.34921775 | 1.667031588 | -1.3834745 | 4.70E-15 | 3.25E-14 |
| SPRR2G | 0.134845745 | 0.019598232 | -2.782514608 | 0.002073344 | 0.003167427 |
| SPHK1 | 1.066055387 | 4.717176796 | 2.145641278 | 0.021121085 | 0.027774647 |
| SFRP1 | 1.541315129 | 0.291376232 | -2.403206755 | 4.27E-26 | 3.73E-24 |
| 4-Mar | 0.003551775 | 0.048208883 | 3.762686778 | 1.63E-22 | 4.37E-21 |
| CACNG4 | 0.104398012 | 1.711858445 | 4.035397265 | 0.019623984 | 0.025972772 |
| RBP2 | 0.057764276 | 1.302895671 | 4.495400204 | 0.002139735 | 0.003265354 |
| QRICH2 | 0.332515117 | 0.953172987 | 1.519318137 | 2.18E-14 | 1.38E-13 |
| ADRA2C | 0.276125279 | 2.708439762 | 3.294067126 | 2.99E-08 | 8.48E-08 |
| COA6 | 10.39476189 | 25.65172257 | 1.303198998 | 2.08E-23 | 6.90E-22 |
| GLI4 | 1.676576996 | 4.772213128 | 1.509139735 | 2.74E-21 | 5.50E-20 |
| GLDN | 0.046056261 | 0.714099836 | 3.954656578 | 4.53E-08 | 1.25E-07 |
| ZG16 | 11.31798501 | 3.525801324 | -1.682594044 | 4.89E-19 | 6.41E-18 |
| RBM24 | 0.298703971 | 1.804439785 | 2.594762677 | 6.58E-12 | 2.98E-11 |
| RPS21 | 117.3610436 | 311.9186161 | 1.410216054 | 1.52E-19 | 2.17E-18 |
| KLHL30 | 0.041133397 | 0.247991869 | 2.591910692 | 4.29E-06 | 9.33E-06 |
| PDE6A | 0.006232652 | 0.01856646 | 1.574780678 | 0.000118223 | 0.000215603 |
| CACNA2D3 | 0.049092617 | 0.223340631 | 2.185667765 | 0.033315958 | 0.042534911 |
| CHRNA1 | 0.035313461 | 0.290436947 | 3.039934865 | 0.011245324 | 0.015424029 |
| TRAM1L1 | 0.055957759 | 0.45903613 | 3.036197612 | 1.35E-06 | 3.13E-06 |
| ZDBF2 | 0.499794646 | 0.217380612 | -1.201112172 | 1.98E-17 | 2.03E-16 |
| RAET1E | 0.011883541 | 0.048730121 | 2.035848992 | 7.08E-14 | 4.20E-13 |
| SOHLH1 | 0.003398374 | 0.261436131 | 6.265470247 | 0.012856088 | 0.017480496 |
| MRAS | 1.177284921 | 2.495785827 | 1.08403062 | 4.81E-07 | 1.17E-06 |
| ZNF385D | 0.032688019 | 0.461308597 | 3.818898321 | 5.37E-05 | 0.00010209 |
| OIT3 | 38.10752279 | 4.713790401 | -3.015116312 | 1.29E-28 | 8.00E-26 |
| LYPD2 | 1.078133294 | 0.307638924 | -1.8092256 | 8.80E-21 | 1.60E-19 |
| THY1 | 1.471376197 | 8.711914494 | 2.565823636 | 1.14E-24 | 5.70E-23 |
| OLFML2B | 0.319386749 | 3.454351332 | 3.43503846 | 6.94E-28 | 1.74E-25 |
| TAC3 | 0.0199593 | 0.184301232 | 3.206932713 | 2.82E-05 | 5.56E-05 |
| ZNF788 | 0.091447756 | 0.221429595 | 1.275828385 | 0.000399931 | 0.000680982 |
| KCNMB3 | 0.059772462 | 0.15152282 | 1.341982227 | 6.55E-08 | 1.78E-07 |
| RPL8 | 223.4587897 | 886.0781387 | 1.987425134 | 1.22E-21 | 2.66E-20 |
| TMEM119 | 0.429169649 | 1.495009405 | 1.800534605 | 0.046319133 | 0.057919392 |
| BCHE | 40.63403462 | 14.4531751 | -1.491302157 | 4.53E-20 | 7.15E-19 |
| CUEDC1 | 1.627572518 | 3.41464315 | 1.069012991 | 1.04E-15 | 7.93E-15 |
| MESP2 | 0.071280974 | 0.770684664 | 3.434551737 | 9.42E-23 | 2.65E-21 |
| CNIH4 | 3.258426613 | 7.016644631 | 1.106605794 | 2.29E-20 | 3.86E-19 |
| BRIP1 | 0.230388876 | 0.627840958 | 1.446328089 | 3.17E-11 | 1.30E-10 |
| MSTO1 | 0.966038953 | 3.065608163 | 1.66602004 | 2.77E-26 | 2.61E-24 |
| C5orf52 | 0.044880372 | 0.00815753 | -2.459880263 | 1.24E-05 | 2.54E-05 |
| SLC30A3 | 0.012869534 | 0.374829201 | 4.864201625 | 2.58E-10 | 9.45E-10 |
| AL021997.3 | 0.02945998 | 0.060850142 | 1.046504181 | 0.005392796 | 0.007750015 |
| WHRN | 0.533389176 | 1.516638378 | 1.50761668 | 5.07E-18 | 5.70E-17 |
| ESR1 | 3.233344743 | 0.612335497 | -2.400633113 | 7.03E-25 | 3.81E-23 |
| SLC27A2 | 98.29063265 | 48.35408661 | -1.023416107 | 5.01E-16 | 3.99E-15 |
| OTOR | 0.002753035 | 0.00127502 | -1.11050301 | 0.000179718 | 0.000319768 |
| PTTG1 | 0.643028279 | 8.334415116 | 3.696126868 | 3.46E-26 | 3.19E-24 |
| CDH13 | 0.158549841 | 0.846827406 | 2.417131533 | 1.11E-26 | 1.23E-24 |
| CCL20 | 10.17004555 | 31.139863 | 1.614436456 | 7.33E-06 | 1.55E-05 |
| KCNK16 | 0.008963874 | 0.001401974 | -2.676662444 | 0.000208069 | 0.000366981 |
| HTR3A | 0.008660971 | 0.345983355 | 5.320029998 | 0.008052174 | 0.011281075 |
| DHODH | 21.31873243 | 7.016243183 | -1.603351004 | 1.23E-20 | 2.16E-19 |
| RHNO1 | 1.967205779 | 4.439467252 | 1.174238682 | 3.01E-22 | 7.56E-21 |
| DNER | 0.006812374 | 1.192738861 | 7.451904842 | 0.005568963 | 0.00799206 |
| SLC26A6 | 0.601971788 | 3.656299116 | 2.602616319 | 7.95E-28 | 1.85E-25 |
| UGT2B7 | 288.465852 | 112.289107 | -1.361182564 | 9.02E-19 | 1.13E-17 |
| CLCN2 | 0.809074754 | 1.654572152 | 1.032113295 | 1.73E-16 | 1.49E-15 |
| CEP250 | 0.706718423 | 1.506816179 | 1.092296006 | 3.45E-23 | 1.09E-21 |
| TNFSF15 | 0.074628791 | 0.295089528 | 1.983348505 | 4.35E-07 | 1.07E-06 |
| ZC3H3 | 4.174295385 | 9.247757735 | 1.147570911 | 7.81E-20 | 1.17E-18 |
| CXCR2 | 0.448896454 | 0.093468316 | -2.263833394 | 2.12E-19 | 2.95E-18 |
| CD226 | 0.274941141 | 0.101346557 | -1.439825726 | 6.33E-20 | 9.74E-19 |
| CMTM5 | 0.029393602 | 0.008972704 | -1.711887398 | 5.36E-18 | 6.01E-17 |
| TMEM249 | 0.01675909 | 0.075336637 | 2.168407793 | 4.34E-09 | 1.36E-08 |
| CALY | 0.009309527 | 0.187125741 | 4.329156279 | 9.29E-11 | 3.59E-10 |
| ATP13A4 | 0.140098918 | 0.034584519 | -2.018247539 | 5.30E-25 | 2.97E-23 |
| DRICH1 | 0.055801372 | 0.147082549 | 1.398253586 | 9.56E-07 | 2.25E-06 |
| APOD | 0.482170324 | 1.142845702 | 1.24501587 | 0.049150611 | 0.06125322 |
| AC003002.3 | 0.009500352 | 0.051576336 | 2.440656487 | 1.60E-06 | 3.66E-06 |
| PDZD7 | 0.024218819 | 0.07100153 | 1.55172161 | 1.83E-12 | 8.91E-12 |
| NOL12 | 0.484807672 | 1.097677889 | 1.178970325 | 2.98E-20 | 4.93E-19 |
| CCDC163 | 0.30464916 | 0.829869689 | 1.445736053 | 3.60E-17 | 3.51E-16 |
| KLK4 | 0.006996408 | 0.908605827 | 7.020896369 | 0.043879217 | 0.055045385 |
| ADH6 | 104.8726235 | 46.92671127 | -1.160156856 | 3.42E-17 | 3.35E-16 |
| MPPED2 | 0.063322184 | 0.176000053 | 1.474792935 | 0.002682829 | 0.004041388 |
| SAMD5 | 3.162964149 | 1.06663541 | -1.568210075 | 5.83E-18 | 6.48E-17 |
| PPP2R2C | 0.016122455 | 0.414968251 | 4.685857654 | 0.000157769 | 0.000282719 |
| PDGFA | 1.490510943 | 6.02532226 | 2.015231437 | 3.99E-16 | 3.23E-15 |
| CLGN | 1.581132315 | 4.316716335 | 1.448976189 | 0.003074991 | 0.004580689 |
| CAGE1 | 0.0059326 | 0.049927955 | 3.073111483 | 0.00032158 | 0.000553691 |
| KRTAP5-7 | 0.018077861 | 0.096619037 | 2.418083476 | 0.000588375 | 0.000978435 |
| SELP | 1.55146931 | 0.555543493 | -1.481663391 | 4.99E-21 | 9.54E-20 |
| WFDC1 | 0.41839228 | 0.195016406 | -1.101260735 | 6.08E-17 | 5.67E-16 |
| SLC22A8 | 0.000665071 | 0.13685638 | 7.684938646 | 4.60E-06 | 9.98E-06 |
| FBXW10 | 0.017314775 | 0.337724054 | 4.285769388 | 3.68E-06 | 8.08E-06 |
| NOTCH3 | 1.167125727 | 5.163076488 | 2.145270989 | 5.69E-20 | 8.83E-19 |
| ELAVL2 | 0.003913556 | 0.022502836 | 2.523554777 | 3.31E-09 | 1.05E-08 |
| MICAL1 | 1.039034513 | 2.199768468 | 1.082108107 | 1.77E-09 | 5.81E-09 |
| PHLDA3 | 2.632575179 | 6.414242496 | 1.284804166 | 0.002960462 | 0.004422784 |
| CAPN14 | 0.006151921 | 0.029724514 | 2.272544229 | 4.80E-06 | 1.04E-05 |
| SLC2A5 | 0.070630364 | 1.431735751 | 4.341332903 | 1.43E-10 | 5.39E-10 |
| GNE | 32.84357454 | 13.0009995 | -1.336988612 | 1.04E-22 | 2.90E-21 |
| SPDYE2 | 0.010368557 | 0.030684761 | 1.565307257 | 7.22E-05 | 0.000135027 |
| SLC5A10 | 0.064203249 | 0.180302581 | 1.489701829 | 0.026432399 | 0.034295335 |
| FANCD2 | 0.183270982 | 0.907608477 | 2.30809171 | 3.07E-25 | 1.90E-23 |
| ACTL10 | 0.1563221 | 0.400516031 | 1.357338241 | 1.11E-05 | 2.30E-05 |
| GPM6A | 1.589885956 | 0.177846931 | -3.160215302 | 3.95E-28 | 1.33E-25 |
| TCF15 | 0.09379808 | 0.499606444 | 2.413161789 | 9.17E-19 | 1.15E-17 |
| C20orf144 | 0.009796834 | 0.057540738 | 2.554196246 | 1.94E-17 | 2.00E-16 |
| COLEC11 | 31.85240278 | 14.1929924 | -1.166223412 | 1.40E-18 | 1.70E-17 |
| OR4C15 | 0.000425198 | 7.58E-05 | -2.488152372 | 0.001017178 | 0.001634503 |
| CSAG2 | 0.08674227 | 0.463337623 | 2.417256723 | 0.020745516 | 0.027320022 |
| MAGEB6 | 3.31E-05 | 0.036443862 | 10.10446792 | 0.008409958 | 0.011756647 |
| TM4SF19-TCTEX1D2 | 0.00231102 | 0.02123628 | 3.199929487 | 0.026140294 | 0.033936139 |
| ASPSCR1 | 3.373146783 | 7.820811746 | 1.213223258 | 4.15E-07 | 1.02E-06 |
| SRRM3 | 0.047534868 | 0.607032206 | 3.674715007 | 1.68E-07 | 4.34E-07 |
| PMP2 | 0.039126936 | 0.014576034 | -1.424563944 | 3.43E-18 | 3.94E-17 |
| MFAP4 | 19.3644898 | 8.444912264 | -1.197259151 | 5.45E-17 | 5.11E-16 |
| LYG2 | 0.021671709 | 0.140179834 | 2.693393988 | 2.84E-11 | 1.18E-10 |
| MLIP | 4.039419925 | 1.995181904 | -1.017627846 | 8.97E-08 | 2.40E-07 |
| ENTHD1 | 0.004026811 | 0.028054269 | 2.800510604 | 0.042173592 | 0.053029721 |
| OR7E24 | 0.001353989 | 0.00054707 | -1.307420034 | 0.005613168 | 0.008048817 |
| MXD3 | 0.31283602 | 1.996056575 | 2.673674075 | 7.48E-28 | 1.76E-25 |
| C1R | 412.2029767 | 173.6226507 | -1.24739975 | 2.98E-24 | 1.31E-22 |
| COL2A1 | 0.005734211 | 3.580810632 | 9.286475508 | 2.22E-05 | 4.44E-05 |
| PRELP | 6.899546803 | 3.396946562 | -1.022263078 | 6.75E-16 | 5.29E-15 |
| CSRNP1 | 35.0541516 | 8.839118126 | -1.987610973 | 4.02E-26 | 3.58E-24 |
| FOXE3 | 0.003672324 | 0.021398094 | 2.542716907 | 1.57E-05 | 3.20E-05 |
| ADGRA1 | 0.090192376 | 0.025601358 | -1.816785137 | 6.83E-23 | 2.00E-21 |
| PAX8 | 0.216921601 | 0.531474885 | 1.292827797 | 1.34E-07 | 3.51E-07 |
| KCTD7 | 0.292208628 | 0.749354429 | 1.358649467 | 3.34E-24 | 1.42E-22 |
| PVALB | 1.609860229 | 0.128579435 | -3.646203617 | 5.26E-28 | 1.50E-25 |
| DNAH17 | 0.031261291 | 0.098653337 | 1.657990486 | 6.81E-18 | 7.49E-17 |
| WFDC11 | 0.001255562 | 0.000564412 | -1.153512718 | 0.001316586 | 0.002076165 |
| HKDC1 | 2.908532029 | 10.02101019 | 1.784664853 | 3.16E-08 | 8.92E-08 |
| ZNF607 | 0.173412052 | 0.551358568 | 1.668786695 | 2.17E-09 | 7.07E-09 |
| PLA2G1B | 0.567349619 | 2.691988297 | 2.246362189 | 0.000192905 | 0.000341824 |
| DDIAS | 0.129122481 | 0.422409155 | 1.709900904 | 1.90E-19 | 2.67E-18 |
| NRK | 0.061905209 | 0.026227379 | -1.238987164 | 2.01E-17 | 2.06E-16 |
| ZNF675 | 0.189291303 | 0.395180011 | 1.061901847 | 5.27E-10 | 1.86E-09 |
| MMP24 | 0.288543126 | 0.873627068 | 1.598230594 | 0.026852529 | 0.034811403 |
| HOXB9 | 0.017008963 | 0.207473449 | 3.608559615 | 8.51E-09 | 2.58E-08 |
| NRG2 | 0.055859908 | 0.315888652 | 2.499530998 | 1.80E-06 | 4.09E-06 |
| GPR15 | 0.078218845 | 0.031060363 | -1.33244155 | 2.73E-06 | 6.08E-06 |
| SLC2A6 | 1.335297969 | 2.879413493 | 1.108613267 | 0.001099871 | 0.001758664 |
| GABBR2 | 0.028161996 | 0.714295771 | 4.664700088 | 1.01E-05 | 2.11E-05 |
| REG4 | 0.075275293 | 0.315278363 | 2.066377849 | 0.019818027 | 0.026211755 |
| LRRC26 | 0.009292309 | 0.166261834 | 4.161276123 | 0.000160494 | 0.000287337 |
| LMNB2 | 1.940025629 | 5.041500857 | 1.377777577 | 2.60E-21 | 5.23E-20 |
| PRR20E | 0.002780662 | 3.07E-05 | -6.502345562 | 0.001282449 | 0.002026441 |
| LKAAEAR1 | 0.004200991 | 0.107975 | 4.683823777 | 6.57E-06 | 1.40E-05 |
| IMPG2 | 0.011631507 | 0.02795181 | 1.264903632 | 1.11E-09 | 3.76E-09 |
| SNRPD2 | 31.67957503 | 78.76519378 | 1.314005263 | 1.08E-19 | 1.58E-18 |
| SOX21 | 0.002717236 | 0.062348237 | 4.520136735 | 0.001990589 | 0.003049088 |
| MDGA1 | 0.105403603 | 0.450576684 | 2.095848481 | 3.49E-05 | 6.81E-05 |
| JPH2 | 0.054392033 | 0.183055529 | 1.750814088 | 6.39E-07 | 1.54E-06 |
| PLEKHD1 | 0.008279853 | 0.022082093 | 1.415199903 | 0.01679261 | 0.022456757 |
| ACADL | 6.024604475 | 2.325626041 | -1.373247398 | 5.52E-18 | 6.17E-17 |
| PAQR5 | 0.527602426 | 1.958137195 | 1.891958745 | 0.010333289 | 0.014268689 |
| NNMT | 442.6019075 | 127.2026482 | -1.798880963 | 4.54E-19 | 6.00E-18 |
| LY6E | 144.9780027 | 48.85862742 | -1.569148779 | 1.06E-20 | 1.90E-19 |
| NCAPD2 | 1.652370999 | 4.598600932 | 1.476657361 | 1.69E-21 | 3.56E-20 |
| PIK3C2G | 3.319014444 | 1.471956874 | -1.173019505 | 3.93E-18 | 4.49E-17 |
| FEZF1 | 0.001804284 | 0.095133382 | 5.720453502 | 3.22E-05 | 6.31E-05 |
| CDT1 | 0.357019314 | 3.731687282 | 3.385754064 | 1.57E-25 | 1.09E-23 |
| BFSP2 | 0.013431525 | 0.127986826 | 3.252300311 | 1.92E-11 | 8.16E-11 |
| OR6J1 | 0.002291187 | 0.000774141 | -1.565426449 | 0.001331095 | 0.002097982 |
| CUL7 | 3.015831554 | 6.893905928 | 1.192765762 | 4.41E-23 | 1.36E-21 |
| C11orf91 | 0.041621636 | 0.085343182 | 1.035942232 | 0.001148523 | 0.001830081 |
| BPIFB4 | 0.00535759 | 0.104314357 | 4.283209689 | 2.15E-07 | 5.51E-07 |
| FEN1 | 3.670106158 | 11.97966173 | 1.706693473 | 3.19E-25 | 1.93E-23 |
| ACTL6B | 0.026992135 | 0.078869433 | 1.546927161 | 0.000707811 | 0.001164348 |
| CAND2 | 0.752658655 | 0.282954173 | -1.411427306 | 6.67E-20 | 1.02E-18 |
| AIRE | 0.002921731 | 0.025817783 | 3.143469959 | 1.04E-06 | 2.44E-06 |
| HP | 2614.394643 | 1201.589721 | -1.121532556 | 6.62E-16 | 5.20E-15 |
| FOXH1 | 0.008689924 | 0.18020203 | 4.37412783 | 2.65E-11 | 1.11E-10 |
| RBM3 | 15.46261036 | 33.16120481 | 1.100712531 | 7.34E-18 | 8.04E-17 |
| CYP2W1 | 0.020939311 | 0.175527275 | 3.067409325 | 2.74E-10 | 1.00E-09 |
| AK8 | 0.071674122 | 0.297488549 | 2.053309914 | 0.03616408 | 0.045903627 |
| AC011452.1 | 0.00072788 | 0.000208126 | -1.806244237 | 0.001257531 | 0.001990504 |
| MAPT | 0.064820813 | 0.54391735 | 3.068858428 | 7.68E-22 | 1.76E-20 |
| THEM5 | 1.045734811 | 3.498313367 | 1.742142484 | 7.80E-17 | 7.10E-16 |
| FITM1 | 5.61056313 | 1.435861504 | -1.966228981 | 1.19E-22 | 3.28E-21 |
| DDX11 | 0.588545156 | 1.42973651 | 1.280524277 | 6.07E-13 | 3.17E-12 |
| TMIGD3 | 1.40710033 | 0.619928286 | -1.182551962 | 6.42E-14 | 3.84E-13 |
| AC008687.4 | 0.013463336 | 0.061447166 | 2.190310597 | 0.019912937 | 0.026328331 |
| COMP | 0.127426335 | 1.016468936 | 2.995830755 | 0.000131973 | 0.000239223 |
| VWF | 2.695652889 | 7.808840881 | 1.534473678 | 1.56E-11 | 6.71E-11 |
| RAB41 | 0.158149115 | 0.318953548 | 1.012060847 | 6.73E-09 | 2.07E-08 |
| C21orf33 | 1.411766802 | 0.682440252 | -1.048727154 | 0.001506988 | 0.002354933 |
| OR4F21 | 0.000715705 | 0.000206265 | -1.794864458 | 0.002663197 | 0.004014146 |
| CTXN3 | 0.003177776 | 0.000646133 | -2.298113692 | 0.00096424 | 0.001555058 |
| DSCAM | 0.038658922 | 0.009864408 | -1.970497063 | 1.87E-18 | 2.23E-17 |
| PPP1R17 | 0.006285964 | 0.002369406 | -1.40760864 | 0.002351787 | 0.00356758 |
| DBT | 5.893081367 | 2.832948257 | -1.056717934 | 1.58E-21 | 3.33E-20 |
| CACNA1G | 0.003877015 | 0.013055104 | 1.751595837 | 0.030331579 | 0.039003158 |
| FANCA | 0.415175016 | 0.863619359 | 1.056675953 | 1.46E-12 | 7.22E-12 |
| PGLYRP2 | 98.79377438 | 43.13610897 | -1.195524085 | 2.21E-16 | 1.87E-15 |
| TTC23L | 0.029111645 | 0.062115542 | 1.093357911 | 4.79E-05 | 9.18E-05 |
| SOWAHC | 15.58923754 | 7.670304363 | -1.023194637 | 3.09E-16 | 2.55E-15 |
| AC243967.1 | 0.002023363 | 0.000189384 | -3.417371554 | 0.000996126 | 0.001602661 |
| AC024940.1 | 0.004004582 | 0.06689812 | 4.062242177 | 7.83E-08 | 2.11E-07 |
| OR7D4 | 0.001230417 | 0.000117535 | -3.387981219 | 0.000735266 | 0.001207438 |
| TRPM8 | 7.0543627 | 2.684827075 | -1.393686584 | 4.79E-16 | 3.83E-15 |
| PKN1 | 10.9945984 | 24.11565061 | 1.133174825 | 2.41E-15 | 1.73E-14 |
| SGO2 | 0.129473666 | 0.721258617 | 2.477857962 | 6.08E-26 | 4.87E-24 |
| FZD10 | 0.013428111 | 0.126268981 | 3.23317198 | 3.83E-07 | 9.48E-07 |
| CENPO | 0.396599589 | 1.18432295 | 1.578307453 | 6.52E-20 | 9.97E-19 |
| EGFL8 | 0.134768609 | 0.330507599 | 1.294198948 | 1.55E-11 | 6.65E-11 |
| CCL13 | 0.138810314 | 0.550297882 | 1.987098011 | 0.000738546 | 0.001212441 |
| ARHGEF26 | 9.240208565 | 4.112314205 | -1.167974917 | 2.34E-17 | 2.38E-16 |
| CEP152 | 0.113727366 | 0.327519089 | 1.525999544 | 1.51E-21 | 3.20E-20 |
| EID2B | 0.271505843 | 0.574860661 | 1.082229063 | 5.70E-13 | 2.99E-12 |
| DCAF4L1 | 0.017866035 | 0.113037349 | 2.661508144 | 5.01E-16 | 3.99E-15 |
| AMIGO3 | 0.005707947 | 0.014784629 | 1.373054228 | 0.000834573 | 0.001358332 |
| JAG2 | 0.589591174 | 1.920756209 | 1.703887583 | 2.77E-18 | 3.24E-17 |
| CA4 | 0.065748633 | 0.62527867 | 3.249466502 | 0.000423856 | 0.000718418 |
| AC134669.2 | 0.018180936 | 0.007621461 | -1.254286983 | 0.000283453 | 0.000491251 |
| OR4Q3 | 0.001889441 | 2.63E-05 | -6.168837298 | 0.000496925 | 0.000834725 |
| FAM25A | 0.055722854 | 0.008760791 | -2.669136178 | 8.16E-06 | 1.72E-05 |
| GSPT2 | 5.13225125 | 2.482540274 | -1.047774677 | 8.56E-17 | 7.74E-16 |
| CCDC188 | 0.047892313 | 0.141368587 | 1.561595555 | 2.86E-15 | 2.03E-14 |
| PFDN6 | 9.015858512 | 18.736095 | 1.055283515 | 6.92E-19 | 8.89E-18 |
| C19orf81 | 0.010648428 | 0.388351968 | 5.188652406 | 1.89E-06 | 4.29E-06 |
| CCR1 | 4.401840082 | 1.634813751 | -1.42898045 | 5.80E-16 | 4.58E-15 |
| SPAG6 | 0.004353211 | 0.034025538 | 2.966466222 | 3.76E-05 | 7.30E-05 |
| GHR | 35.06383225 | 7.691864711 | -2.188578389 | 1.98E-24 | 9.14E-23 |
| ACSM3 | 11.97117023 | 3.114576085 | -1.942456467 | 3.19E-25 | 1.93E-23 |
| RDH16 | 136.3682549 | 43.38090829 | -1.652375674 | 3.92E-21 | 7.63E-20 |
| CPLX2 | 0.014442237 | 8.783865722 | 9.248417991 | 9.99E-13 | 5.07E-12 |
| PNMA5 | 0.008816206 | 1.281058427 | 7.182962614 | 0.01507649 | 0.020313557 |
| BARD1 | 0.249987424 | 0.61529223 | 1.299416252 | 4.18E-15 | 2.91E-14 |
| SIGLEC8 | 0.47796575 | 0.202386325 | -1.239795432 | 2.57E-07 | 6.51E-07 |
| FAM72A | 0.036453115 | 0.148708448 | 2.028372603 | 2.77E-19 | 3.81E-18 |
| EYA1 | 0.00349055 | 0.058863003 | 4.075834865 | 2.58E-05 | 5.12E-05 |
| NGFR | 9.471400919 | 2.565532891 | -1.88431931 | 6.33E-23 | 1.88E-21 |
| SPC24 | 0.366208905 | 4.03082935 | 3.46033793 | 1.54E-26 | 1.61E-24 |
| KRTAP13-4 | 0.000589917 | 0.000125221 | -2.236039895 | 0.000891799 | 0.001444219 |
| LGI3 | 0.02043131 | 0.214251069 | 3.390448783 | 0.015542884 | 0.02089847 |
| ZNF320 | 0.316995993 | 0.905035262 | 1.513509398 | 0.001746106 | 0.002699316 |
| DBF4B | 0.215858066 | 0.654623247 | 1.600581834 | 6.34E-24 | 2.48E-22 |
| DSN1 | 2.551136354 | 6.266138235 | 1.296436586 | 3.49E-23 | 1.10E-21 |
| CNGB3 | 0.00394977 | 0.023674204 | 2.583475375 | 5.50E-05 | 0.00010446 |
| PTPRR | 0.017256837 | 0.076449581 | 2.147340513 | 1.60E-06 | 3.66E-06 |
| RPS12 | 373.9859986 | 999.2101264 | 1.417803839 | 4.74E-13 | 2.52E-12 |
| OR4D5 | 0.0006011 | 0.000265473 | -1.179039262 | 0.002344509 | 0.003557581 |
| NPIPB13 | 0.006718238 | 0.021349002 | 1.668013716 | 3.13E-05 | 6.15E-05 |
| MNS1 | 0.341856104 | 2.27917187 | 2.737048628 | 1.17E-09 | 3.93E-09 |
| NQO1 | 2.032490898 | 56.56242612 | 4.798523206 | 6.89E-11 | 2.72E-10 |
| ATAD2 | 2.02416717 | 6.80516206 | 1.749301076 | 7.08E-19 | 9.06E-18 |
| SLC45A2 | 0.105908938 | 0.400929292 | 1.920523482 | 0.047682727 | 0.059519245 |
| MPZ | 0.925803419 | 7.136860451 | 2.946511768 | 6.93E-05 | 0.000129878 |
| C17orf53 | 0.268971344 | 1.044463753 | 1.957238041 | 1.26E-19 | 1.82E-18 |
| TRIP13 | 0.13668791 | 1.584649364 | 3.535206104 | 4.72E-28 | 1.38E-25 |
| PFKP | 1.374521211 | 4.568391701 | 1.732757185 | 0.009153929 | 0.012729446 |
| VSTM1 | 0.06255198 | 0.020000746 | -1.645001725 | 4.24E-11 | 1.72E-10 |
| TMEM89 | 0.00559359 | 0.045364873 | 3.019729318 | 1.35E-05 | 2.77E-05 |
| PRKAR2B | 1.192342706 | 0.379483009 | -1.651691762 | 8.03E-25 | 4.26E-23 |
| TPX2 | 1.838836216 | 8.451062986 | 2.200339831 | 2.05E-17 | 2.10E-16 |
| KMO | 11.68130954 | 3.542269019 | -1.721456329 | 4.00E-22 | 9.71E-21 |
| RPL13A | 264.3143542 | 636.2681044 | 1.267380026 | 7.01E-17 | 6.45E-16 |
| AL355315.1 | 0.002567502 | 0.000403814 | -2.668602638 | 0.004608142 | 0.006689072 |
| DUOX1 | 0.086877135 | 0.437929003 | 2.333648571 | 7.91E-15 | 5.32E-14 |
| PMFBP1 | 0.025174314 | 0.190890748 | 2.922722804 | 4.54E-22 | 1.08E-20 |
| CLEC18B | 0.021468938 | 0.053547629 | 1.318571866 | 1.17E-06 | 2.73E-06 |
| CISH | 13.27258928 | 6.416258664 | -1.048645637 | 2.59E-09 | 8.32E-09 |
| SERTAD4 | 0.045497337 | 0.180861115 | 1.991028239 | 0.033571389 | 0.042829329 |
| CR1L | 0.091777362 | 0.043216754 | -1.086547616 | 1.13E-16 | 1.00E-15 |
| BLK | 0.274510357 | 0.106194101 | -1.370156955 | 3.51E-16 | 2.88E-15 |
| LIN9 | 0.313616617 | 0.942359855 | 1.587276077 | 2.79E-21 | 5.59E-20 |
| LIG1 | 2.024342384 | 4.514046397 | 1.156967929 | 5.62E-20 | 8.74E-19 |
| CYP7A1 | 7.255726145 | 28.51821729 | 1.97469189 | 0.012992065 | 0.017653323 |
| CD302 | 17.17881367 | 5.808864436 | -1.564302344 | 8.84E-25 | 4.63E-23 |
| BSN | 0.106136972 | 0.28746564 | 1.437462229 | 1.89E-05 | 3.80E-05 |
| RHEBL1 | 0.27739444 | 0.642846913 | 1.212536348 | 6.73E-14 | 4.01E-13 |
| SCN11A | 0.060321059 | 0.024497872 | -1.300005349 | 6.11E-17 | 5.69E-16 |
| TNR | 0.186926016 | 0.060040342 | -1.638463274 | 9.42E-15 | 6.29E-14 |
| SEMA3A | 0.044286063 | 0.129764237 | 1.55096819 | 0.042486496 | 0.053392964 |
| AKR7A3 | 83.00557911 | 35.94407657 | -1.207454268 | 1.83E-17 | 1.89E-16 |
| FAM221B | 0.024066275 | 0.058138189 | 1.272473288 | 3.20E-05 | 6.28E-05 |
| ASIC3 | 0.141408248 | 0.600731353 | 2.08685369 | 2.11E-16 | 1.79E-15 |
| HSPB1 | 82.97013851 | 369.4417597 | 2.15468285 | 5.01E-23 | 1.53E-21 |
| LAMA4 | 0.756735844 | 2.228891713 | 1.55846484 | 5.74E-23 | 1.74E-21 |
| CCDC187 | 0.038166237 | 0.589519846 | 3.949171537 | 1.82E-06 | 4.14E-06 |
| KIF21B | 0.297198311 | 0.890069345 | 1.582491822 | 2.07E-08 | 5.99E-08 |
| MTUS2 | 0.284578101 | 0.068441115 | -2.055889482 | 8.35E-19 | 1.05E-17 |
| TMEM82 | 29.44743767 | 9.891147557 | -1.57393229 | 6.19E-20 | 9.55E-19 |
| DEPDC1 | 0.060242518 | 0.872866137 | 3.856906435 | 8.10E-26 | 6.15E-24 |
| SEMA5B | 0.145075521 | 0.743827549 | 2.358164075 | 9.34E-26 | 6.96E-24 |
| ZFP36 | 176.5504645 | 58.33776341 | -1.597578635 | 3.21E-20 | 5.25E-19 |
| SPRR2E | 0.277743624 | 0.040176542 | -2.789328504 | 0.003913037 | 0.005746557 |
| LRP12 | 0.21240715 | 0.454122287 | 1.096248511 | 0.005621726 | 0.008060346 |
| LRRC39 | 0.159180364 | 0.423900828 | 1.413064408 | 1.26E-08 | 3.74E-08 |
| ANKRD34A | 0.076135954 | 0.188187708 | 1.30552259 | 3.27E-15 | 2.31E-14 |
| MANF | 20.51192533 | 43.90856096 | 1.098039338 | 3.55E-16 | 2.90E-15 |
| TLR4 | 3.578999183 | 1.329357772 | -1.428826783 | 2.19E-21 | 4.48E-20 |
| HOXA6 | 0.0023472 | 0.05578463 | 4.57085504 | 6.58E-05 | 0.000123716 |
| EN1 | 0.001073815 | 0.064071604 | 5.898868216 | 0.002027925 | 0.003102517 |
| RBMS3 | 0.946231525 | 0.33181268 | -1.511824206 | 3.56E-25 | 2.08E-23 |
| DIO2 | 0.021987702 | 0.481943715 | 4.454095918 | 3.26E-06 | 7.19E-06 |
| OR4F5 | 0.000518121 | 9.01E-05 | -2.524035689 | 0.001454028 | 0.002276748 |
| CACYBP | 5.583465061 | 11.94780073 | 1.097512449 | 2.66E-20 | 4.45E-19 |
| AC093668.1 | 0.01488968 | 0.001774587 | -3.068757726 | 0.002111119 | 0.003224186 |
| PCDH11Y | 0.00108042 | 0.00046591 | -1.213469458 | 0.001910878 | 0.002933923 |
| CYB561D1 | 0.395963564 | 0.950761429 | 1.263715694 | 2.77E-17 | 2.77E-16 |
| ZNF625-ZNF20 | 0.00399181 | 0.00803663 | 1.009547674 | 0.033673728 | 0.042956368 |
| CEACAM20 | 0.034421058 | 1.241359873 | 5.172486164 | 1.61E-05 | 3.27E-05 |
| MEOX2 | 0.153075871 | 0.49208636 | 1.684664638 | 4.41E-08 | 1.22E-07 |
| ACLY | 8.443272452 | 18.96160384 | 1.167206824 | 1.60E-20 | 2.77E-19 |
| PIM1 | 26.49909015 | 12.32279759 | -1.104613003 | 2.55E-06 | 5.69E-06 |
| ZSCAN10 | 0.001808844 | 0.008300378 | 2.198108959 | 0.002308301 | 0.003506226 |
| HOXB13 | 0.003112607 | 0.247377731 | 6.312448412 | 6.21E-05 | 0.000117326 |
| PHF19 | 0.661705015 | 1.810322185 | 1.45198636 | 5.71E-21 | 1.08E-19 |
| KRT1 | 0.379964904 | 0.130487966 | -1.541949406 | 0.00011643 | 0.000212532 |
| USP17L2 | 0.001216212 | 0.000422255 | -1.526207502 | 0.000764194 | 0.001251903 |
| ZNF695 | 0.002827639 | 0.08021098 | 4.826129767 | 1.60E-10 | 6.03E-10 |
| HAO1 | 187.126585 | 91.95263385 | -1.025051732 | 1.13E-19 | 1.65E-18 |
| LINC01125 | 0.104743772 | 0.238828596 | 1.189111117 | 2.09E-10 | 7.75E-10 |
| WNT6 | 0.056890558 | 0.413970907 | 2.863268251 | 1.06E-06 | 2.49E-06 |
| ADAMDEC1 | 0.23493152 | 0.729295558 | 1.634263316 | 3.96E-06 | 8.66E-06 |
| ZNF705G | 0.001965595 | 7.42E-05 | -4.728249665 | 0.000736575 | 0.00120946 |
| ERICH3 | 0.067632752 | 0.033776964 | -1.001682397 | 4.98E-13 | 2.64E-12 |
| WDR72 | 6.422243051 | 2.816100674 | -1.189378355 | 2.76E-17 | 2.76E-16 |
| HSD17B13 | 183.7223785 | 55.80770579 | -1.718991122 | 3.34E-20 | 5.43E-19 |
| TTK | 0.075612026 | 1.143670692 | 3.918912187 | 6.13E-27 | 7.94E-25 |
| CEMIP | 0.122185729 | 0.823284564 | 2.752315381 | 1.20E-12 | 6.01E-12 |
| SLCO1C1 | 0.003792164 | 0.064135535 | 4.08003083 | 3.08E-24 | 1.34E-22 |
| TLX2 | 0.00218693 | 0.043570378 | 4.316368827 | 0.000174483 | 0.000311094 |
| ATP13A5 | 0.003748909 | 0.000784664 | -2.256323097 | 1.98E-06 | 4.47E-06 |
| HES5 | 0.043325374 | 0.153232318 | 1.822436481 | 2.14E-09 | 6.97E-09 |
| VCX3A | 0.002282844 | 0.452542469 | 7.631077214 | 0.002504278 | 0.00378488 |
| ARHGAP39 | 0.303242342 | 0.901018055 | 1.571084804 | 1.87E-18 | 2.23E-17 |
| ITLN1 | 2.241058428 | 0.397201146 | -2.496238574 | 3.26E-25 | 1.95E-23 |
| SLC6A14 | 0.00366336 | 0.180159049 | 5.619959741 | 0.013470307 | 0.018266584 |
| MEGF11 | 0.006142813 | 0.015112594 | 1.298779886 | 0.035352709 | 0.044936067 |
| OR6V1 | 0.000850898 | 0.000184047 | -2.208914297 | 0.000268379 | 0.000466686 |
| OR10G7 | 0.000718323 | 2.63E-05 | -4.773582653 | 0.000800203 | 0.001306354 |
| P3H4 | 1.275752637 | 3.671695715 | 1.525097879 | 5.39E-14 | 3.25E-13 |
| NAMPT | 38.29208712 | 16.40178476 | -1.223193486 | 1.11E-12 | 5.58E-12 |
| HOXD4 | 0.002525207 | 0.119270879 | 5.561696323 | 2.28E-15 | 1.65E-14 |
| PROK2 | 0.21057666 | 0.027932299 | -2.91433929 | 2.13E-15 | 1.55E-14 |
| ZNF883 | 0.058238491 | 0.458708102 | 2.977531511 | 2.92E-08 | 8.28E-08 |
| CCDC78 | 0.037532696 | 0.216116192 | 2.52558735 | 2.24E-17 | 2.29E-16 |
| UROC1 | 50.97418814 | 12.27470691 | -2.054078316 | 1.20E-21 | 2.62E-20 |
| SPATA18 | 1.920255528 | 0.897580688 | -1.097184762 | 3.73E-13 | 2.01E-12 |
| TMEM253 | 0.024737615 | 0.092996501 | 1.910470025 | 1.50E-12 | 7.40E-12 |
| SLC22A12 | 0.115046802 | 3.929045913 | 5.093886245 | 0.012313222 | 0.016791051 |
| GPR63 | 0.009582508 | 0.025080545 | 1.388093552 | 6.28E-05 | 0.000118486 |
| PLAC8 | 2.661035608 | 0.585309751 | -2.184715596 | 1.45E-24 | 7.04E-23 |
| DNASE1L2 | 0.068178681 | 0.467197973 | 2.776641425 | 3.73E-13 | 2.01E-12 |
| NEGR1 | 0.040086318 | 0.018584008 | -1.109048258 | 4.55E-16 | 3.65E-15 |
| PTGDR | 0.226563858 | 0.104672049 | -1.114041499 | 1.19E-13 | 6.82E-13 |
| ASPA | 1.890853418 | 0.515676542 | -1.874499203 | 3.93E-22 | 9.56E-21 |
| DNMT3B | 0.191266701 | 0.635756825 | 1.732889317 | 6.35E-18 | 7.02E-17 |
| NOS2 | 0.14138611 | 0.414994128 | 1.553450531 | 1.08E-08 | 3.25E-08 |
| KRTAP5-10 | 0.019980524 | 0.100998052 | 2.337661153 | 1.18E-07 | 3.10E-07 |
| LPCAT1 | 2.375560098 | 6.470527339 | 1.44561559 | 7.51E-10 | 2.59E-09 |
| OR5AK2 | 0.009997542 | 0.001506373 | -2.73049491 | 3.60E-07 | 8.94E-07 |
| CYP3A43 | 3.923907738 | 1.496757948 | -1.390450189 | 1.68E-16 | 1.45E-15 |
| RBL1 | 0.381924915 | 0.942606844 | 1.303367119 | 2.14E-17 | 2.19E-16 |
| SEMA6D | 0.601503522 | 0.270548239 | -1.15268733 | 1.61E-17 | 1.68E-16 |
| ELL3 | 0.057681332 | 0.150930869 | 1.387711525 | 1.40E-15 | 1.05E-14 |
| SLC45A1 | 0.147896126 | 0.308622769 | 1.061260243 | 1.17E-08 | 3.48E-08 |
| B3GALT1 | 0.006068951 | 0.130031184 | 4.421266639 | 0.031043364 | 0.039832797 |
| FBXL16 | 0.150509782 | 0.842795773 | 2.485325828 | 0.046792521 | 0.058469042 |
| MAGEE1 | 0.182547533 | 0.367821025 | 1.01073178 | 8.86E-06 | 1.86E-05 |
| FGF22 | 0.020306174 | 0.070250729 | 1.790594724 | 0.000626885 | 0.001038337 |
| TMEM155 | 0.006341185 | 0.070266051 | 3.470003458 | 2.76E-05 | 5.44E-05 |
| SLC6A3 | 0.004785507 | 0.147456607 | 4.945474922 | 0.004819842 | 0.006978177 |
| KRBA1 | 0.381902913 | 0.817624369 | 1.098232269 | 7.07E-05 | 0.000132446 |
| C8orf44 | 0.375360608 | 0.914350848 | 1.284470597 | 8.53E-18 | 9.25E-17 |
| KRTCAP2 | 3.54389803 | 7.909308393 | 1.158214454 | 3.54E-20 | 5.73E-19 |
| CTAGE4 | 0.033627935 | 0.06943877 | 1.046081196 | 0.018832923 | 0.02497891 |
| AC096887.1 | 0.061110096 | 0.131069247 | 1.100846576 | 8.70E-11 | 3.38E-10 |
| FBN3 | 0.021804209 | 0.266520762 | 3.611569342 | 1.80E-06 | 4.10E-06 |
| NR4A2 | 7.129238982 | 2.102703937 | -1.761502352 | 7.81E-16 | 6.07E-15 |
| DBN1 | 1.575433138 | 5.59302634 | 1.827880597 | 1.10E-09 | 3.71E-09 |
| BORA | 0.24105479 | 0.517456395 | 1.1020762 | 3.32E-12 | 1.57E-11 |
| PKDCC | 2.031932838 | 5.182797106 | 1.350878199 | 2.33E-06 | 5.22E-06 |
| PFKFB2 | 0.570480108 | 1.33469908 | 1.226266022 | 1.46E-13 | 8.30E-13 |
| COL15A1 | 0.254638142 | 3.798185477 | 3.898789916 | 2.66E-28 | 1.15E-25 |
| TTLL10 | 0.007811358 | 0.01800564 | 1.204803643 | 9.65E-06 | 2.02E-05 |
| PRDM7 | 0.003917797 | 0.059202723 | 3.9175489 | 2.48E-12 | 1.18E-11 |
| LYVE1 | 10.26756446 | 1.512996433 | -2.762613513 | 4.47E-26 | 3.82E-24 |
| CEACAM3 | 0.103508663 | 0.035012727 | -1.563800195 | 6.28E-18 | 6.95E-17 |
| ITGA11 | 0.193858197 | 0.418276912 | 1.109456627 | 0.000133295 | 0.000241478 |
| TYRO3 | 0.281015372 | 0.931310972 | 1.728613924 | 0.000480153 | 0.000808116 |
| A1BG | 45.12804796 | 22.33230794 | -1.014892021 | 1.83E-16 | 1.57E-15 |
| PRRX2 | 0.030208066 | 0.23220649 | 2.942402559 | 2.34E-05 | 4.66E-05 |
| VN1R2 | 0.001967466 | 0.000948156 | -1.053142207 | 0.000744846 | 0.001222267 |
| TRAF5 | 0.257361517 | 0.769835859 | 1.580754529 | 2.84E-21 | 5.68E-20 |
| GINS3 | 0.260981031 | 0.721038562 | 1.466131467 | 2.14E-20 | 3.61E-19 |
| C6orf48 | 13.72052161 | 35.0481739 | 1.353003949 | 4.57E-18 | 5.18E-17 |
| FAM71F2 | 0.049627759 | 0.141441789 | 1.510989203 | 9.05E-09 | 2.73E-08 |
| OCM | 0.026078934 | 0.076111968 | 1.545238453 | 4.10E-05 | 7.93E-05 |
| C5AR1 | 5.290305929 | 2.613913202 | -1.017139917 | 3.12E-11 | 1.28E-10 |
| CD1A | 0.037325132 | 0.139972637 | 1.906925564 | 0.020611328 | 0.027152502 |
| CHRNB4 | 0.013656943 | 0.062726606 | 2.199442927 | 9.20E-12 | 4.09E-11 |
| AC007192.1 | 0.002407605 | 0.013060486 | 2.439537892 | 1.64E-06 | 3.74E-06 |
| EGR2 | 4.216620874 | 0.73884799 | -2.512737831 | 2.69E-22 | 6.85E-21 |
| OR51E1 | 0.018104279 | 0.161378672 | 3.156047331 | 3.56E-25 | 2.08E-23 |
| SLC5A5 | 0.009369263 | 0.058512509 | 2.642737534 | 0.016021811 | 0.021499684 |
| JRK | 0.223643632 | 0.800556236 | 1.839801071 | 2.31E-25 | 1.51E-23 |
| TUBB3 | 0.017668259 | 0.082229657 | 2.218498921 | 3.84E-09 | 1.21E-08 |
| SPEM1 | 0.002793529 | 0.001394582 | -1.002256697 | 0.049389387 | 0.061526149 |
| TDRD12 | 0.007752589 | 0.018352487 | 1.243225416 | 0.004489273 | 0.006527487 |
| DCAF13 | 1.913374134 | 3.933865342 | 1.039828574 | 1.19E-13 | 6.85E-13 |
| SOX11 | 0.00237254 | 0.025127331 | 3.404753332 | 8.10E-07 | 1.92E-06 |
| SCML2 | 0.323693172 | 0.812969548 | 1.328574379 | 1.37E-11 | 5.93E-11 |
| OR8D4 | 0.001063631 | 0.00039518 | -1.428414221 | 0.000810932 | 0.00132262 |
| CCNE1 | 0.181871442 | 3.528462631 | 4.278048803 | 1.40E-26 | 1.50E-24 |
| PPP1R14C | 0.019758383 | 0.401526505 | 4.344958405 | 0.001831996 | 0.002820045 |
| CHST10 | 0.29322556 | 0.706348511 | 1.268369316 | 0.040945344 | 0.051560312 |
| TRPM5 | 0.004651378 | 0.039766134 | 3.095810122 | 0.000118818 | 0.000216637 |
| LGALS4 | 53.22961157 | 135.3471942 | 1.346364036 | 0.003609854 | 0.005326053 |
| C15orf48 | 1.024114449 | 5.220360807 | 2.349772571 | 0.000251677 | 0.000439708 |
| SLC8A1 | 0.323031445 | 0.127818146 | -1.337581945 | 7.34E-23 | 2.13E-21 |
| CHRNG | 0.004232469 | 0.012348083 | 1.544715784 | 0.000430774 | 0.000729348 |
| ZNF382 | 0.054389691 | 0.155914562 | 1.519350543 | 7.60E-07 | 1.81E-06 |
| OIP5 | 0.367808686 | 1.750330023 | 2.250599511 | 8.68E-21 | 1.58E-19 |
| ACSM5 | 61.60646773 | 20.34442002 | -1.598448667 | 2.00E-21 | 4.12E-20 |
| ZNF517 | 0.918723642 | 2.97573356 | 1.695542497 | 2.53E-22 | 6.48E-21 |
| B3GALNT1 | 0.324578744 | 0.649696036 | 1.00119638 | 2.76E-06 | 6.15E-06 |
| SYT3 | 0.021781487 | 0.661255467 | 4.924033392 | 0.005527056 | 0.007936312 |
| HLA-DOB | 0.540638887 | 1.261948425 | 1.222915759 | 0.035032769 | 0.044554878 |
| FBXO32 | 0.557335755 | 1.790509299 | 1.683751396 | 2.32E-10 | 8.54E-10 |
| DYTN | 0.00174573 | 0.000574966 | -1.602281172 | 0.002169438 | 0.003307409 |
| SARDH | 43.71623675 | 18.47872189 | -1.24230424 | 1.87E-22 | 4.94E-21 |
| SLC38A1 | 1.853168221 | 3.983051321 | 1.103880221 | 0.013916525 | 0.01884035 |
| PRAMEF8 | 0.005022775 | 0.087165769 | 4.117205186 | 0.016512533 | 0.022112114 |
| PTGIS | 3.164966088 | 1.112174888 | -1.508806374 | 5.02E-21 | 9.57E-20 |
| RECQL4 | 0.807025247 | 4.904344433 | 2.60337459 | 1.53E-24 | 7.27E-23 |
| OR10K1 | 0.000775805 | 0.000176645 | -2.134842649 | 0.002310018 | 0.003508323 |
| SMARCA4 | 3.416468522 | 6.868875026 | 1.007568001 | 8.90E-25 | 4.64E-23 |
| FAM47B | 0.001081492 | 0.000188809 | -2.518022356 | 0.004968703 | 0.007178963 |
| GPR35 | 0.126130901 | 1.070778111 | 3.085665878 | 7.29E-08 | 1.97E-07 |
| HIST1H1C | 67.11929607 | 231.5423303 | 1.786476479 | 1.36E-08 | 4.00E-08 |
| DLX1 | 0.003256691 | 0.156709091 | 5.588538307 | 2.14E-09 | 6.97E-09 |
| ADRA2B | 2.533009546 | 0.56664587 | -2.160333216 | 2.30E-25 | 1.51E-23 |
| MPP3 | 0.134107019 | 0.517911537 | 1.949320942 | 6.24E-13 | 3.25E-12 |
| KLRF1 | 0.856229929 | 0.254405184 | -1.750870196 | 1.28E-20 | 2.25E-19 |
| TMSB10 | 287.4074479 | 1382.109141 | 2.265702192 | 9.75E-12 | 4.32E-11 |
| MPC1 | 102.2588362 | 45.16712578 | -1.178880496 | 4.22E-22 | 1.02E-20 |
| DLG2 | 0.191865394 | 0.092202841 | -1.057211417 | 2.70E-12 | 1.28E-11 |
| AHRR | 0.052480449 | 0.229390687 | 2.127954837 | 0.015230544 | 0.020501675 |
| OR1A2 | 0.000734669 | 0.000177849 | -2.046443 | 0.001102431 | 0.001762394 |
| AC007998.2 | 0.008434612 | 0.076335548 | 3.177961368 | 2.23E-05 | 4.45E-05 |
| TRPC4 | 0.046666793 | 0.1392649 | 1.577363442 | 3.19E-07 | 7.96E-07 |
| SERPINB10 | 0.003950757 | 0.001876742 | -1.073898851 | 0.000350291 | 0.000600135 |
| FNDC10 | 0.462678282 | 1.517853244 | 1.713951021 | 0.007892569 | 0.011076365 |
| AC010325.1 | 0.02310356 | 0.085675742 | 1.890771614 | 0.009386315 | 0.013031616 |
| AKR1C3 | 23.55556129 | 82.1083112 | 1.801460553 | 4.42E-20 | 7.00E-19 |
| AC003688.1 | 0.001933194 | 0.000723359 | -1.41820315 | 0.00157008 | 0.00244542 |
| ANLN | 0.14957018 | 1.994203509 | 3.736918166 | 1.25E-26 | 1.37E-24 |
| TUBE1 | 4.523042627 | 1.345347689 | -1.749314524 | 1.07E-25 | 7.82E-24 |
| GNMT | 145.9966148 | 69.81005182 | -1.064428231 | 4.35E-13 | 2.32E-12 |
| PLXNA3 | 0.478031975 | 1.293237073 | 1.435807745 | 2.58E-12 | 1.23E-11 |
| DNAH14 | 0.139370259 | 0.362266098 | 1.378127077 | 1.57E-19 | 2.23E-18 |
| FAM83F | 0.311646101 | 0.090460681 | -1.784545904 | 1.15E-20 | 2.04E-19 |
| HMGA2 | 0.004525521 | 0.210327449 | 5.538409423 | 0.043370769 | 0.054442668 |
| SHMT1 | 64.24399528 | 31.98897297 | -1.005986941 | 2.40E-17 | 2.43E-16 |
| IL17B | 0.007470703 | 0.058347234 | 2.965348444 | 5.76E-10 | 2.03E-09 |
| SCUBE3 | 0.029071104 | 0.08340888 | 1.520615127 | 8.63E-10 | 2.96E-09 |
| C5orf34 | 0.091602937 | 0.542361177 | 2.565788152 | 3.62E-28 | 1.31E-25 |
| BUB1 | 0.107220519 | 1.327952605 | 3.630550727 | 4.48E-27 | 6.33E-25 |
| GPHN | 9.3861278 | 4.240968313 | -1.1461364 | 9.00E-20 | 1.33E-18 |
| CCL4 | 5.529850253 | 2.269010456 | -1.285177156 | 2.03E-15 | 1.48E-14 |
| DBNDD1 | 2.445587331 | 7.102927058 | 1.538232686 | 5.13E-10 | 1.81E-09 |
| DDIT4L | 0.08008582 | 0.443409105 | 2.469019675 | 0.012420635 | 0.016925658 |
| ENTPD2 | 0.559910884 | 1.812519983 | 1.69472777 | 3.36E-07 | 8.37E-07 |
| NAT2 | 33.26323333 | 5.04773901 | -2.720219188 | 2.44E-27 | 4.07E-25 |
| FOSL1 | 2.5972157 | 0.900860055 | -1.527590924 | 0.000443946 | 0.000750181 |
| JCHAIN | 31.99104638 | 11.72062585 | -1.448618574 | 9.35E-17 | 8.42E-16 |
| DUSP1 | 224.2200588 | 103.2206382 | -1.119183892 | 2.31E-15 | 1.67E-14 |
| PYGO2 | 5.959950476 | 14.6695354 | 1.299450932 | 2.34E-27 | 3.96E-25 |
| TSPAN5 | 0.285683643 | 1.294420779 | 2.179816333 | 0.007526442 | 0.01058646 |
| TEX38 | 0.012742764 | 0.04700305 | 1.883076076 | 1.80E-05 | 3.64E-05 |
| MASP1 | 21.64586421 | 7.808383537 | -1.470995578 | 7.59E-26 | 5.81E-24 |
| TDO2 | 98.99541436 | 36.32808177 | -1.44627651 | 1.92E-18 | 2.29E-17 |
| SAC3D1 | 3.588280665 | 8.55968707 | 1.254265316 | 4.35E-19 | 5.78E-18 |
| KIAA1522 | 4.209918556 | 12.77707477 | 1.601693349 | 6.56E-21 | 1.23E-19 |
| CYP27B1 | 0.098182721 | 0.347412362 | 1.823108033 | 6.38E-13 | 3.32E-12 |
| TMC1 | 0.008051036 | 0.02011829 | 1.321261296 | 0.000950387 | 0.001533829 |
| GPA33 | 0.069024378 | 0.024953298 | -1.467875477 | 2.96E-06 | 6.57E-06 |
| MPIG6B | 0.072737698 | 0.034392566 | -1.080606486 | 8.35E-13 | 4.29E-12 |
| KCNH6 | 0.007335217 | 0.230556155 | 4.974134716 | 0.000667713 | 0.001101738 |
| WDR38 | 0.00295489 | 0.024348515 | 3.042657225 | 1.11E-05 | 2.30E-05 |
| CFAP44 | 0.176013048 | 0.360578295 | 1.03463017 | 1.76E-10 | 6.59E-10 |
| CXorf36 | 0.315757146 | 1.515017704 | 2.262447364 | 6.36E-28 | 1.69E-25 |
| AC090360.1 | 0.042703468 | 0.092208614 | 1.1105483 | 0.000899608 | 0.001456107 |
| SLC4A11 | 0.067429041 | 0.499968523 | 2.890395292 | 2.07E-08 | 5.97E-08 |
| NUTM2G | 0.012742877 | 0.025888646 | 1.022628533 | 5.27E-05 | 0.000100332 |
| GDF2 | 9.624173303 | 0.366950602 | -4.713004851 | 3.68E-29 | 4.09E-26 |
| THRSP | 99.98842636 | 44.95056359 | -1.153421909 | 2.88E-14 | 1.80E-13 |
| UBE2S | 1.478809695 | 5.612264133 | 1.924146502 | 1.17E-21 | 2.56E-20 |
| TAX1BP3 | 4.640016713 | 9.391196371 | 1.017178957 | 1.20E-05 | 2.47E-05 |
| SFI1 | 0.507138015 | 1.24544731 | 1.296213661 | 1.29E-17 | 1.37E-16 |
| PLGLB1 | 1.734167231 | 0.776707489 | -1.158799747 | 7.39E-14 | 4.37E-13 |
| ALOX15B | 0.176134489 | 1.714731872 | 3.283233664 | 6.31E-05 | 0.000119032 |
| CRIP3 | 0.740334431 | 3.197272402 | 2.110592633 | 7.03E-16 | 5.49E-15 |
| RPL39L | 1.875343432 | 5.915869474 | 1.657435401 | 0.004369666 | 0.006361305 |
| AC004233.2 | 0.018040842 | 0.078522106 | 2.121832193 | 0.011403139 | 0.015632217 |
| E2F2 | 0.056845022 | 0.509076128 | 3.162775495 | 2.74E-25 | 1.73E-23 |
| ZNF213 | 0.54162329 | 1.116299655 | 1.043362668 | 1.24E-16 | 1.09E-15 |
| KIAA1462 | 0.697612023 | 2.493809313 | 1.837854345 | 8.05E-21 | 1.48E-19 |
| C3orf36 | 0.038261945 | 0.109038537 | 1.510855988 | 4.23E-05 | 8.18E-05 |
| AKR1B15 | 0.124813463 | 1.914024041 | 3.938763491 | 3.13E-07 | 7.82E-07 |
| H1F0 | 72.22620084 | 160.7257868 | 1.154007223 | 1.08E-14 | 7.15E-14 |
| PRRT2 | 0.053048671 | 0.17946275 | 1.758295921 | 2.34E-17 | 2.38E-16 |
| CKLF-CMTM1 | 0.029265157 | 0.066066442 | 1.174733609 | 2.46E-07 | 6.24E-07 |
| ZNF431 | 0.112377121 | 0.261449589 | 1.218184458 | 3.30E-10 | 1.19E-09 |
| MYBPHL | 0.005769815 | 0.317784603 | 5.783380405 | 7.70E-11 | 3.01E-10 |
| BMP7 | 0.018918038 | 0.265452839 | 3.810621209 | 0.002677412 | 0.004034009 |
| CCNO | 0.098417142 | 0.970583736 | 3.30187116 | 9.33E-07 | 2.20E-06 |
| HIST1H4D | 0.065626762 | 0.2597145 | 1.984570412 | 1.29E-08 | 3.80E-08 |
| RASGRF2 | 0.159441298 | 0.621123143 | 1.96185396 | 2.08E-23 | 6.90E-22 |
| MYCBPAP | 0.009897975 | 0.035787286 | 1.854241828 | 4.44E-12 | 2.06E-11 |
| RACGAP1 | 0.617310313 | 3.364322312 | 2.446248127 | 1.35E-27 | 2.65E-25 |
| C20orf96 | 1.428802608 | 2.902214144 | 1.022347356 | 1.98E-17 | 2.03E-16 |
| ADM | 14.59142037 | 6.708140541 | -1.121135507 | 5.38E-09 | 1.67E-08 |
| KCNN4 | 0.263415737 | 0.555466203 | 1.076357596 | 0.003843668 | 0.005650551 |
| C19orf48 | 6.148783571 | 15.74733878 | 1.356735109 | 1.07E-16 | 9.55E-16 |
| ZNF605 | 0.399979254 | 0.886515956 | 1.148221426 | 3.24E-19 | 4.40E-18 |
| LCE3E | 0.019714753 | 0.004162288 | -2.243827058 | 0.00263949 | 0.00398187 |
| EDDM13 | 0.027336815 | 0.059612197 | 1.124762408 | 2.12E-05 | 4.23E-05 |
| ADAMTS20 | 0.000701939 | 0.01308498 | 4.220422302 | 0.013172357 | 0.017885802 |
| PARPBP | 0.100025363 | 0.713922223 | 2.835401046 | 8.89E-27 | 1.06E-24 |
| APOA5 | 224.2084921 | 108.6595591 | -1.045025824 | 8.34E-14 | 4.89E-13 |
| PMCH | 0.007965626 | 0.029304038 | 1.879239849 | 0.002978153 | 0.00444639 |
| BRINP2 | 0.082814816 | 0.02259483 | -1.873896219 | 3.14E-21 | 6.23E-20 |
| CEP72 | 0.600987056 | 1.545047835 | 1.362245681 | 1.69E-17 | 1.76E-16 |
| FRZB | 1.252114318 | 2.838486734 | 1.180755713 | 6.43E-05 | 0.000121111 |
| SSTR3 | 0.008274793 | 0.070458726 | 3.089983208 | 0.004829117 | 0.006989634 |
| RGS7 | 0.085637341 | 0.032134434 | -1.414119942 | 0.00979702 | 0.01356068 |
| ARTN | 0.047567542 | 0.171945304 | 1.853900329 | 4.67E-13 | 2.49E-12 |
| ERICH4 | 0.005036974 | 0.148564491 | 4.882388362 | 1.17E-05 | 2.41E-05 |
| POU4F3 | 0.006613902 | 0.017326573 | 1.389412668 | 0.035458125 | 0.045050604 |
| PLXDC1 | 0.098370647 | 0.906893345 | 3.204633095 | 1.48E-28 | 8.50E-26 |
| MAGEB2 | 0.001411715 | 2.061924972 | 10.51232711 | 0.001839627 | 0.002831274 |
| UNKL | 0.542578131 | 1.111450929 | 1.034541449 | 6.53E-13 | 3.39E-12 |
| CELA3B | 0.003776981 | 0.074074569 | 4.293672976 | 0.013490479 | 0.018292214 |
| NOX4 | 0.031185757 | 0.241359363 | 2.952223614 | 4.59E-28 | 1.38E-25 |
| IL1RL1 | 1.393832405 | 0.142923296 | -3.285744104 | 1.06E-17 | 1.13E-16 |
| MCIDAS | 0.002518917 | 0.044730942 | 4.150397597 | 0.000173646 | 0.000309719 |
| CLEC1B | 5.801043623 | 0.159725944 | -5.182641894 | 1.13E-29 | 2.52E-26 |
| UGT2B10 | 212.1749725 | 92.41851111 | -1.199000738 | 3.25E-14 | 2.02E-13 |
| OR4F6 | 0.000373658 | 2.63E-05 | -3.830666857 | 0.000537151 | 0.000898698 |
| S100A10 | 32.57181921 | 96.1146087 | 1.561131427 | 2.83E-15 | 2.01E-14 |
| SMPX | 0.11184996 | 1.112233822 | 3.313823468 | 1.42E-06 | 3.27E-06 |
| AL662899.3 | 0.015366974 | 0.03159786 | 1.039993761 | 1.63E-05 | 3.31E-05 |
| CELF3 | 0.000763471 | 0.021250472 | 4.798777457 | 2.02E-12 | 9.77E-12 |
| PLK1 | 0.19789194 | 2.431154095 | 3.618856583 | 5.58E-27 | 7.48E-25 |
| IL18R1 | 2.484685285 | 0.909851102 | -1.449360758 | 7.51E-24 | 2.88E-22 |
| MTFR2 | 0.071290257 | 0.660879002 | 3.212609333 | 6.00E-26 | 4.84E-24 |
| ALX4 | 0.001934105 | 0.030269127 | 3.968108965 | 0.001572001 | 0.002448167 |
| MYPN | 0.003642712 | 0.007834179 | 1.104769016 | 0.030661921 | 0.039385759 |
| WDR62 | 0.115711225 | 0.72610435 | 2.649648066 | 1.79E-25 | 1.22E-23 |
| FSHR | 0.003722938 | 0.001357729 | -1.455245923 | 6.54E-06 | 1.39E-05 |
| NRXN1 | 0.018760504 | 0.003990645 | -2.233004781 | 2.25E-20 | 3.79E-19 |
| ZWINT | 1.288339737 | 7.30973567 | 2.504306153 | 2.12E-23 | 7.02E-22 |
| SRC | 2.149647576 | 4.910775741 | 1.191850784 | 3.84E-06 | 8.42E-06 |
| CASQ2 | 0.172162023 | 0.467935268 | 1.44254203 | 1.10E-06 | 2.58E-06 |
| CDCP2 | 0.00317427 | 0.007145753 | 1.170661276 | 0.023232378 | 0.030357986 |
| SLC38A4 | 129.302654 | 62.28133503 | -1.053880113 | 8.81E-16 | 6.77E-15 |
| MIA | 0.022241473 | 0.073402057 | 1.722568149 | 0.008688612 | 0.012121428 |
| ZNF454 | 0.03542091 | 0.08849508 | 1.320995978 | 0.006117476 | 0.008726911 |
| SGCG | 0.003314366 | 0.028076015 | 3.082533261 | 0.033365271 | 0.042594312 |
| C19orf57 | 0.262726585 | 0.677936402 | 1.367587743 | 3.88E-10 | 1.39E-09 |
| TRNP1 | 3.106092861 | 10.72592209 | 1.787928812 | 0.025046347 | 0.032600273 |
| SCARA3 | 1.728256522 | 5.3740382 | 1.636689207 | 2.34E-05 | 4.65E-05 |
| FAM50A | 14.73475969 | 32.46938964 | 1.139856733 | 1.71E-18 | 2.06E-17 |
| C1orf100 | 0.017938671 | 0.076181218 | 2.086362373 | 2.41E-08 | 6.92E-08 |
| SPRY2 | 9.761321077 | 3.644768655 | -1.421249167 | 2.25E-24 | 1.01E-22 |
| RFPL1 | 0.10527752 | 0.006581103 | -3.999724151 | 1.22E-20 | 2.16E-19 |
| LY6H | 0.019798813 | 0.360946883 | 4.188300667 | 4.05E-22 | 9.81E-21 |
| TCHH | 0.113296538 | 0.037897993 | -1.579910447 | 1.54E-19 | 2.19E-18 |
| FOXO1 | 13.36835964 | 4.518534808 | -1.564895509 | 1.04E-21 | 2.30E-20 |
| PRDM15 | 0.262460574 | 0.654772134 | 1.318892205 | 4.23E-19 | 5.64E-18 |
| STEAP1B | 0.011456444 | 0.136308529 | 3.572644629 | 6.16E-16 | 4.85E-15 |
| C6orf229 | 0.01605753 | 0.06502606 | 2.017768021 | 0.006013344 | 0.008585446 |
| OR5H15 | 0.000412488 | 0.000151337 | -1.446586708 | 0.001609846 | 0.002502347 |
| MYBL2 | 0.340309377 | 8.296104025 | 4.607515176 | 9.07E-27 | 1.06E-24 |
| CDC37L1 | 17.25697785 | 6.36822722 | -1.438216115 | 3.23E-25 | 1.94E-23 |
| C19orf67 | 0.009790921 | 0.078170648 | 2.997110501 | 1.54E-12 | 7.62E-12 |
| AFM | 173.9291153 | 61.96837506 | -1.488895413 | 2.80E-20 | 4.66E-19 |
| CNIH2 | 0.082806113 | 0.300199166 | 1.85811079 | 1.93E-12 | 9.39E-12 |
| CYTL1 | 0.079203275 | 0.264358012 | 1.738861063 | 9.12E-09 | 2.75E-08 |
| RDH5 | 6.953686202 | 2.490242059 | -1.481491981 | 3.29E-20 | 5.36E-19 |
| GPC2 | 0.020529574 | 0.098409983 | 2.261100994 | 3.58E-19 | 4.83E-18 |
| PLPPR2 | 3.739181408 | 8.50400757 | 1.185420415 | 9.74E-19 | 1.21E-17 |
| MYO1H | 0.0065143 | 0.024649288 | 1.919863934 | 1.11E-08 | 3.32E-08 |
| NR4A3 | 2.436441866 | 0.543736051 | -2.16379741 | 1.06E-17 | 1.13E-16 |
| OR6T1 | 0.021921856 | 0.010280066 | -1.092520396 | 0.00306005 | 0.004560178 |
| BCAT1 | 0.170363627 | 0.626535911 | 1.878779851 | 5.30E-16 | 4.20E-15 |
| OR2T33 | 0.000699146 | 0.00028454 | -1.296960701 | 0.003879707 | 0.0057003 |
| SPRR4 | 0.010034895 | 0.00134215 | -2.902407454 | 0.001049033 | 0.001682389 |
| LIPG | 6.092444577 | 2.934042292 | -1.054131556 | 1.36E-12 | 6.75E-12 |
| JUNB | 109.4410612 | 52.53767917 | -1.058729748 | 2.17E-15 | 1.58E-14 |
| DLL4 | 1.378955211 | 2.979205793 | 1.111352185 | 8.18E-14 | 4.80E-13 |
| FOXD4 | 0.033107115 | 0.108211484 | 1.708640402 | 7.77E-09 | 2.37E-08 |
| LAMA3 | 0.437651018 | 2.143388354 | 2.292040439 | 2.15E-07 | 5.51E-07 |
| HIST1H3D | 0.356495975 | 1.697762781 | 2.251677199 | 2.00E-11 | 8.49E-11 |
| CD244 | 1.060012749 | 0.338588343 | -1.646477408 | 1.51E-20 | 2.63E-19 |
| S100A12 | 1.993643786 | 0.280069085 | -2.83155301 | 2.50E-20 | 4.18E-19 |
| PHGDH | 30.60165762 | 12.99688925 | -1.23544344 | 6.64E-17 | 6.14E-16 |
| CCNI2 | 0.025028059 | 0.085739635 | 1.776415889 | 2.47E-07 | 6.26E-07 |
| UPK2 | 0.016396703 | 0.081241586 | 2.308812646 | 6.02E-06 | 1.29E-05 |
| ARHGAP10 | 1.444462494 | 0.529899666 | -1.446741621 | 7.07E-17 | 6.50E-16 |
| KIF7 | 0.271409254 | 0.645082572 | 1.24901393 | 6.59E-06 | 1.40E-05 |
| SCP2 | 90.30574685 | 44.87461368 | -1.008918281 | 1.18E-16 | 1.05E-15 |
| DNAH8 | 0.004875452 | 0.02089119 | 2.099286924 | 3.83E-05 | 7.43E-05 |
| ACSL4 | 9.312100675 | 46.94229365 | 2.333709775 | 2.14E-09 | 6.97E-09 |
| OR4D1 | 0.001871189 | 0.000369216 | -2.341417477 | 0.00026977 | 0.000469001 |
| CPE | 7.708894039 | 21.57563224 | 1.484807033 | 1.95E-05 | 3.92E-05 |
| IL9R | 0.011329259 | 0.022913997 | 1.016175676 | 0.000130013 | 0.000235861 |
| FHL5 | 0.098478112 | 0.252857764 | 1.360451077 | 0.005495818 | 0.007892914 |
| TINAG | 0.014590362 | 2.289390983 | 7.293804413 | 1.49E-08 | 4.38E-08 |
| TEX11 | 0.023193262 | 0.408512141 | 4.138601294 | 0.001706362 | 0.002642076 |
| GPR160 | 0.493903763 | 1.002400905 | 1.021157758 | 0.030709473 | 0.039443407 |
| ZNF233 | 0.082802319 | 0.272202307 | 1.716936225 | 9.22E-09 | 2.78E-08 |
| ULK4 | 0.491161107 | 1.082847879 | 1.140562354 | 0.001123954 | 0.001794524 |
| CCDC34 | 0.95794025 | 3.050400197 | 1.670990951 | 6.90E-20 | 1.05E-18 |
| ALOX15 | 0.029027734 | 0.095356133 | 1.715893762 | 0.003087265 | 0.004597652 |
| KCNK12 | 0.002208851 | 0.032455894 | 3.877112423 | 7.22E-05 | 0.000135027 |
| C4orf46 | 0.421863425 | 0.97060594 | 1.202109675 | 1.42E-21 | 3.04E-20 |
| SSPO | 0.047241771 | 0.14306837 | 1.59856979 | 5.24E-05 | 9.99E-05 |
| HIST1H2BO | 0.135161597 | 0.559652637 | 2.049846353 | 5.39E-10 | 1.90E-09 |
| SPATC1L | 0.326400759 | 2.432952956 | 2.897992106 | 2.33E-15 | 1.67E-14 |
| ANXA10 | 26.30850639 | 8.287457852 | -1.666527812 | 2.68E-20 | 4.47E-19 |
| TAGLN3 | 0.001882486 | 0.018950699 | 3.331539975 | 0.001831861 | 0.002820045 |
| ADGRL1 | 0.362559089 | 1.331390707 | 1.876645957 | 2.66E-05 | 5.26E-05 |
| LAMC1 | 6.26322986 | 18.27459815 | 1.544860951 | 1.07E-19 | 1.56E-18 |
| CXCL14 | 20.45437091 | 2.153262919 | -3.247812774 | 9.55E-28 | 2.09E-25 |
| IL33 | 8.092957981 | 2.947297852 | -1.457274239 | 2.14E-21 | 4.39E-20 |
| CACNA1E | 0.006236299 | 0.054500896 | 3.127517854 | 4.25E-05 | 8.19E-05 |
| RFPL3S | 0.03683342 | 0.08062241 | 1.130165575 | 1.06E-05 | 2.20E-05 |
| SPARC | 37.71817321 | 97.72843788 | 1.373518627 | 1.25E-10 | 4.77E-10 |
| RCVRN | 0.030056036 | 0.006500979 | -2.20892583 | 1.11E-16 | 9.91E-16 |
| MYOT | 0.080802674 | 0.029096798 | -1.473542667 | 3.29E-14 | 2.04E-13 |
| ACADM | 40.70022225 | 20.01846791 | -1.023705109 | 1.40E-18 | 1.70E-17 |
| MUSTN1 | 0.077060991 | 0.30882284 | 2.00270681 | 0.002028063 | 0.003102517 |
| CD200R1 | 0.426738861 | 0.183187989 | -1.220028582 | 1.69E-15 | 1.24E-14 |
| KRTAP24-1 | 0.001078778 | 6.15E-05 | -4.133549963 | 0.000604991 | 0.00100421 |
| PCDH9 | 0.167432472 | 0.051862116 | -1.69082637 | 1.82E-22 | 4.84E-21 |
| TBX4 | 0.002561156 | 0.673232114 | 8.038164904 | 2.45E-14 | 1.55E-13 |
| ASCL1 | 2.540335218 | 1.195256219 | -1.087698973 | 0.00108094 | 0.001730349 |
| ZNF813 | 0.284671817 | 0.594875761 | 1.063288725 | 0.006117474 | 0.008726911 |
| GTSE1 | 0.098129205 | 1.23133429 | 3.649396097 | 2.69E-27 | 4.41E-25 |
| RNF43 | 1.953651553 | 4.245055583 | 1.119610268 | 0.00560058 | 0.008032988 |
| CACNA1C | 0.065172088 | 0.158589704 | 1.282972998 | 4.54E-09 | 1.42E-08 |
| POLQ | 0.035084237 | 0.304227868 | 3.116257435 | 3.68E-25 | 2.13E-23 |
| CHST1 | 0.475804752 | 1.23366341 | 1.374507242 | 2.40E-05 | 4.77E-05 |
| AKAP14 | 0.004980689 | 0.057489178 | 3.528873168 | 9.73E-05 | 0.000179036 |
| SRSF12 | 0.109663911 | 0.246643112 | 1.169336162 | 1.47E-05 | 3.00E-05 |
| KRT76 | 0.00122731 | 0.000552238 | -1.152138882 | 0.008817332 | 0.012291018 |
| FANCE | 0.471839106 | 1.499000067 | 1.667633548 | 3.86E-20 | 6.18E-19 |
| SMC4 | 0.863859417 | 2.113500842 | 1.290766233 | 1.14E-09 | 3.86E-09 |
| KIAA1841 | 0.168039238 | 0.523892045 | 1.640471405 | 9.75E-27 | 1.10E-24 |
| CAVIN4 | 0.042211212 | 0.155408751 | 1.880369588 | 7.65E-19 | 9.71E-18 |
| HIVEP1 | 2.918968673 | 1.356239704 | -1.105846543 | 1.24E-15 | 9.36E-15 |
| ABCA3 | 0.438092616 | 1.333779722 | 1.606212617 | 5.11E-05 | 9.74E-05 |
| FOXM1 | 0.273459866 | 3.538913104 | 3.69390532 | 7.84E-27 | 9.75E-25 |
| GATA3 | 0.99948818 | 0.410774724 | -1.282842094 | 6.03E-15 | 4.12E-14 |
| TMEM201 | 0.613049581 | 1.521430493 | 1.311352762 | 6.49E-23 | 1.92E-21 |
| CEL | 0.049983826 | 0.506857102 | 3.342045817 | 1.03E-11 | 4.55E-11 |
| DSE | 1.128816237 | 0.422486871 | -1.417832229 | 1.96E-25 | 1.31E-23 |
| MSS51 | 0.132660474 | 0.344349597 | 1.376135404 | 7.33E-17 | 6.70E-16 |
| TESMIN | 0.082764989 | 0.460275966 | 2.475406592 | 9.90E-18 | 1.06E-16 |
| ZNF572 | 0.226910599 | 0.626721543 | 1.465700588 | 3.37E-14 | 2.09E-13 |
| C1QTNF3 | 0.468794 | 7.152114959 | 3.931343917 | 3.79E-07 | 9.39E-07 |
| KANK4 | 0.670591205 | 0.304590972 | -1.138560381 | 3.51E-14 | 2.17E-13 |
| AMY1A | 0.000808832 | 0.000225597 | -1.842093946 | 0.000793647 | 0.001297013 |
| PPOX | 1.820882914 | 4.029836516 | 1.146083155 | 4.43E-22 | 1.06E-20 |
| RFLNA | 0.036326148 | 1.052383638 | 4.856508533 | 5.44E-09 | 1.68E-08 |
| H1FNT | 0.00295717 | 0.051453128 | 4.120969763 | 0.000935867 | 0.001511722 |
| FAM163B | 2.673559247 | 0.540927338 | -2.305254931 | 8.10E-22 | 1.84E-20 |
| SLC51B | 2.688004156 | 9.588200581 | 1.834724721 | 6.53E-11 | 2.58E-10 |
| RANBP3L | 2.282602137 | 0.498207369 | -2.195861151 | 3.42E-20 | 5.55E-19 |
| SAA2 | 391.8912218 | 121.6942564 | -1.687192179 | 5.58E-08 | 1.53E-07 |
| CAMK2B | 1.475945062 | 0.49098959 | -1.587874681 | 7.22E-17 | 6.62E-16 |
| SPDL1 | 0.389431422 | 1.386643926 | 1.832156168 | 3.45E-28 | 1.31E-25 |
| OSBP2 | 0.197467259 | 0.680320925 | 1.784601994 | 2.15E-11 | 9.07E-11 |
| DCN | 23.70119288 | 5.596331927 | -2.082406233 | 8.94E-24 | 3.34E-22 |
| MCMDC2 | 0.107483089 | 0.282071799 | 1.391952749 | 1.39E-11 | 6.02E-11 |
| ARFGEF3 | 0.069643155 | 0.572434763 | 3.039057821 | 2.55E-06 | 5.71E-06 |
| SLCO1B3 | 20.01053851 | 5.300114495 | -1.916664562 | 3.29E-19 | 4.46E-18 |
| TRPV4 | 4.254024377 | 1.856154512 | -1.196511491 | 8.13E-16 | 6.29E-15 |
| ZNF726 | 0.154752894 | 0.388060539 | 1.326315345 | 0.000425825 | 0.000721362 |
| CUBN | 0.026235551 | 0.060589932 | 1.207555015 | 3.37E-11 | 1.38E-10 |
| KRTAP4-9 | 0.000564403 | 0.00023811 | -1.245098879 | 0.001450945 | 0.002272382 |
| PITX2 | 0.002893456 | 0.095731743 | 5.048131797 | 0.041494096 | 0.052209069 |
| C8B | 224.9390581 | 105.0368266 | -1.098638956 | 6.12E-21 | 1.16E-19 |
| ALG1L | 0.794880906 | 7.700846111 | 3.27620634 | 1.06E-13 | 6.12E-13 |
| XKRX | 0.014484903 | 0.056721152 | 1.969336784 | 1.16E-05 | 2.40E-05 |
| ANO2 | 0.021209591 | 0.19363345 | 3.190539522 | 7.95E-13 | 4.10E-12 |
| C7orf61 | 0.061607214 | 0.154408976 | 1.325585413 | 0.001357958 | 0.002136862 |
| GLMP | 13.54590109 | 28.71780161 | 1.084088947 | 4.47E-15 | 3.10E-14 |
| HAGHL | 0.064175891 | 0.504934818 | 2.975993831 | 6.50E-22 | 1.50E-20 |
| PIANP | 0.016742798 | 0.051632199 | 1.624730432 | 0.008214693 | 0.011499399 |
| SEPT5 | 0.345940359 | 1.362344724 | 1.977496566 | 1.20E-14 | 7.93E-14 |
| MYRFL | 0.024414225 | 0.081254656 | 1.734728521 | 1.69E-05 | 3.42E-05 |
| ZBED8 | 0.430434691 | 1.023156702 | 1.249160858 | 3.16E-17 | 3.11E-16 |
| ATAD5 | 0.130037127 | 0.440206718 | 1.759257577 | 4.38E-22 | 1.05E-20 |
| SRXN1 | 0.546492278 | 1.418643695 | 1.376239274 | 4.26E-07 | 1.05E-06 |
| CASKIN1 | 0.004920647 | 0.053244385 | 3.43570955 | 2.06E-10 | 7.63E-10 |
| DSCR8 | 0.003053182 | 1.055973315 | 8.434044298 | 4.09E-05 | 7.92E-05 |
| TAS2R5 | 0.045961001 | 0.118308794 | 1.364075185 | 3.08E-07 | 7.70E-07 |
| HOXC6 | 0.004871708 | 0.156960543 | 5.00983058 | 3.77E-09 | 1.19E-08 |
| SLC19A3 | 6.999587153 | 2.681602254 | -1.384174565 | 8.30E-22 | 1.88E-20 |
| ASPHD1 | 0.452228631 | 2.964431214 | 2.712631084 | 0.017802046 | 0.023710661 |
| SIX2 | 0.006718468 | 0.537966276 | 6.323239548 | 7.95E-20 | 1.19E-18 |
| CBFA2T2 | 0.83978029 | 1.922736832 | 1.195077479 | 2.82E-24 | 1.24E-22 |
| EGR3 | 1.061168554 | 0.238822465 | -2.15164337 | 5.90E-22 | 1.39E-20 |
| TMEM206 | 0.58708542 | 1.503654974 | 1.356831233 | 7.12E-25 | 3.84E-23 |
| ST6GALNAC1 | 0.162357735 | 0.075395862 | -1.10661886 | 1.21E-13 | 6.94E-13 |
| DCAF8L1 | 0.00045049 | 0.170193727 | 8.561466734 | 0.01102945 | 0.015151714 |
| PAQR8 | 0.519629137 | 1.314349463 | 1.338794679 | 0.002351249 | 0.003567113 |
| DCAF8L2 | 0.000147193 | 0.127817231 | 9.762155405 | 0.00011587 | 0.00021159 |
| NRIP2 | 0.463798343 | 1.059956109 | 1.192434956 | 6.84E-15 | 4.63E-14 |
| SAMD12 | 0.505215006 | 0.219701298 | -1.201354095 | 8.30E-14 | 4.87E-13 |
| SLC5A4 | 0.040141904 | 0.114608651 | 1.513534989 | 3.90E-07 | 9.65E-07 |
| PKMYT1 | 0.127403511 | 1.23251429 | 3.27412743 | 1.09E-27 | 2.27E-25 |
| SLC22A10 | 25.91225764 | 6.305760697 | -2.038892392 | 1.43E-19 | 2.05E-18 |
| BIK | 0.493324383 | 1.79297359 | 1.861745738 | 0.008304616 | 0.01161795 |
| RASEF | 0.611804668 | 1.368858752 | 1.161830567 | 0.006615327 | 0.009376083 |
| SLC6A17 | 0.005733225 | 0.050216735 | 3.130749483 | 3.37E-09 | 1.07E-08 |
| FAM189B | 2.060273285 | 6.603098386 | 1.680307426 | 2.34E-27 | 3.96E-25 |
| QRFPR | 0.011182318 | 0.061827505 | 2.467029543 | 0.000984064 | 0.001585221 |
| CHRNA3 | 0.004015501 | 0.071844877 | 4.161233407 | 0.000116455 | 0.000212532 |
| PPP1R1B | 0.054017796 | 0.67511285 | 3.643622003 | 0.000270406 | 0.000470053 |
| OR13C8 | 0.001294447 | 0.000184021 | -2.814394902 | 0.001124144 | 0.001794524 |
| NUSAP1 | 1.598294814 | 8.088115828 | 2.339270112 | 4.23E-23 | 1.31E-21 |
| TESC | 2.191831938 | 15.55348821 | 2.827029086 | 0.007018783 | 0.009912693 |
| SASS6 | 0.42253798 | 0.87484864 | 1.04995241 | 3.86E-14 | 2.38E-13 |
| SLX1B | 0.003195797 | 0.011160755 | 1.804187112 | 0.003981 | 0.005839783 |
| LY6G6F | 0.008014713 | 0.003857102 | -1.05513349 | 0.000115939 | 0.000211686 |
| PTPRD | 1.39908918 | 0.682826213 | -1.034897577 | 7.05E-13 | 3.65E-12 |
| KLRC3 | 0.019703128 | 0.008723355 | -1.175469617 | 6.51E-05 | 0.000122554 |
| MBL2 | 65.18099525 | 22.63249262 | -1.526055897 | 8.13E-17 | 7.38E-16 |
| NPY5R | 0.158538405 | 0.063617172 | -1.317344222 | 5.94E-22 | 1.39E-20 |
| SLCO5A1 | 0.018150674 | 0.052763549 | 1.539518503 | 0.008410551 | 0.011756647 |
| IYD | 5.533145869 | 1.898823036 | -1.5429945 | 4.20E-20 | 6.69E-19 |
| CLSPN | 0.043717446 | 0.342618786 | 2.970323225 | 1.98E-22 | 5.17E-21 |
| CASTOR2 | 0.236592959 | 0.708575127 | 1.582513682 | 2.64E-16 | 2.20E-15 |
| MMS22L | 0.076010678 | 0.206237509 | 1.440032744 | 2.30E-15 | 1.66E-14 |
| AD000671.1 | 0.010215719 | 0.022897042 | 1.164370391 | 0.026681533 | 0.034609913 |
| IGF1 | 2.448374095 | 1.024997591 | -1.25620349 | 1.93E-13 | 1.08E-12 |
| GPX8 | 0.571097596 | 1.213450318 | 1.087305826 | 0.000141492 | 0.000255346 |
| ARHGEF16 | 1.375559095 | 2.776949165 | 1.013482647 | 0.02895024 | 0.037356542 |
| HMGB4 | 0.000368659 | 0.000128835 | -1.516759113 | 0.002784707 | 0.004181092 |
| SCN4B | 0.200979754 | 0.497750252 | 1.308371871 | 3.28E-08 | 9.26E-08 |
| USP54 | 0.3365883 | 0.739915603 | 1.136375694 | 0.003609926 | 0.005326053 |
| PRC1 | 0.349333457 | 3.380992694 | 3.274770174 | 3.77E-28 | 1.33E-25 |
| SPEF1 | 0.039446759 | 0.11613032 | 1.557766033 | 0.000156606 | 0.000280732 |
| CDCA3 | 0.132477119 | 1.362278707 | 3.362206779 | 8.43E-27 | 1.02E-24 |
| AGXT2 | 29.02246508 | 9.316773078 | -1.639267803 | 4.12E-23 | 1.28E-21 |
| LRRC10B | 0.067642537 | 0.230864114 | 1.771041253 | 9.10E-13 | 4.64E-12 |
| GCH1 | 30.41171162 | 12.3482296 | -1.300322803 | 2.97E-20 | 4.91E-19 |
| PSMB4 | 64.62147305 | 138.962754 | 1.104612709 | 9.28E-28 | 2.06E-25 |
| GATS | 0.497066649 | 1.507423513 | 1.600573587 | 1.97E-20 | 3.36E-19 |
| SIGLEC14 | 1.475639905 | 0.460211377 | -1.680972154 | 1.91E-11 | 8.13E-11 |
| MAG | 0.220617898 | 0.030378252 | -2.860439072 | 9.40E-08 | 2.50E-07 |
| COLCA2 | 0.199188953 | 1.396371524 | 2.809473295 | 8.66E-18 | 9.37E-17 |
| KRTAP4-12 | 0.002171021 | 0.000221361 | -3.29390068 | 0.001159073 | 0.001846116 |
| LINC00890 | 2.890372231 | 1.101302468 | -1.392044546 | 1.10E-09 | 3.74E-09 |
| OR4A16 | 0.000684683 | 0.000157332 | -2.12162859 | 0.001777621 | 0.002744763 |
| GCM1 | 0.003447008 | 0.06439591 | 4.223552421 | 9.76E-08 | 2.59E-07 |
| FCRL6 | 0.596713561 | 0.269921092 | -1.144500849 | 5.04E-12 | 2.32E-11 |
| HIGD1B | 0.154214021 | 1.354958048 | 3.135242341 | 4.54E-27 | 6.36E-25 |
| TARBP1 | 1.147691213 | 3.993165294 | 1.798798257 | 5.37E-26 | 4.42E-24 |
| GJB3 | 0.97169272 | 0.337878988 | -1.523993527 | 5.12E-12 | 2.35E-11 |
| GABRA5 | 0.072916859 | 0.034273457 | -1.089160683 | 0.000845104 | 0.001373889 |
| OGDHL | 34.54303078 | 14.60952706 | -1.241485194 | 1.07E-19 | 1.57E-18 |
| SUCO | 2.071665895 | 4.649079704 | 1.166153806 | 1.60E-22 | 4.32E-21 |
| CREB3L1 | 0.362081514 | 2.069354581 | 2.514794443 | 0.002699123 | 0.004062785 |
| RMDN2 | 3.303599391 | 1.537602941 | -1.10335575 | 2.38E-21 | 4.82E-20 |
| NXPH4 | 0.054611056 | 2.943109393 | 5.752004295 | 3.65E-26 | 3.34E-24 |
| LY6G5B | 0.13552188 | 0.376425067 | 1.473836915 | 7.14E-13 | 3.70E-12 |
| MAP2K1 | 20.25463624 | 9.778479522 | -1.050570115 | 4.87E-22 | 1.15E-20 |
| HIST1H2BE | 0.101415122 | 0.34555037 | 1.768623234 | 6.30E-10 | 2.20E-09 |
| CCT3 | 32.86801698 | 86.99010303 | 1.404166856 | 3.28E-28 | 1.30E-25 |
| SAA1 | 3232.344618 | 1207.447398 | -1.42062068 | 2.80E-06 | 6.23E-06 |
| AQP3 | 44.3315813 | 19.00638252 | -1.221850854 | 3.74E-19 | 5.03E-18 |
| FCN3 | 59.59267179 | 4.23619642 | -3.814293541 | 2.24E-28 | 1.06E-25 |
| CDC6 | 0.27602566 | 2.626410547 | 3.250218155 | 6.16E-26 | 4.89E-24 |
| SPARCL1 | 6.505882107 | 44.81196401 | 2.784067373 | 4.47E-10 | 1.59E-09 |
| AGRN | 7.800087216 | 17.02867056 | 1.126403647 | 1.35E-07 | 3.53E-07 |
| THBS4 | 0.081478008 | 4.6134954 | 5.823305705 | 2.26E-24 | 1.02E-22 |
| ZNF724 | 0.041028485 | 0.123081206 | 1.5849127 | 3.77E-10 | 1.36E-09 |
| CXCL2 | 54.8354726 | 22.50592866 | -1.284804367 | 2.56E-15 | 1.83E-14 |
| EBF2 | 0.004108368 | 0.17678046 | 5.42724949 | 1.54E-28 | 8.54E-26 |
| CDC45 | 0.242149507 | 2.157379074 | 3.155309722 | 3.59E-25 | 2.09E-23 |
| CPT2 | 32.81973236 | 16.31469127 | -1.008391787 | 1.87E-21 | 3.87E-20 |
| PI15 | 0.017507095 | 0.314775623 | 4.168312207 | 2.12E-11 | 8.96E-11 |
| CCDC28B | 0.402070312 | 2.056594979 | 2.354737982 | 1.37E-21 | 2.93E-20 |
| SLC26A2 | 0.456477227 | 1.071294576 | 1.230740444 | 3.90E-20 | 6.25E-19 |
| PLCB4 | 0.073750807 | 0.168453432 | 1.191619088 | 7.23E-06 | 1.53E-05 |
| SLC38A8 | 0.006075335 | 0.236082341 | 5.280182397 | 0.000735191 | 0.001207438 |
| NLRP12 | 0.05796583 | 0.024608929 | -1.236020856 | 4.29E-12 | 1.99E-11 |
| C2orf66 | 0.007299431 | 0.025844848 | 1.82402086 | 6.57E-06 | 1.40E-05 |
| DRP2 | 0.001139692 | 0.016450474 | 3.85141282 | 1.50E-13 | 8.48E-13 |
| NCKAP5 | 0.622431382 | 0.258494714 | -1.26778002 | 6.14E-17 | 5.71E-16 |
| SPRR2D | 0.10499279 | 0.039556574 | -1.408300868 | 0.023785023 | 0.03104164 |
| DDN | 0.009166034 | 0.041055309 | 2.16319925 | 3.90E-05 | 7.57E-05 |
| CCDC42 | 0.111740503 | 0.044703356 | -1.321697169 | 4.79E-15 | 3.31E-14 |
| TIMD4 | 5.103886705 | 0.493429607 | -3.370680116 | 3.10E-24 | 1.34E-22 |
| SEMA7A | 0.900661997 | 2.31484553 | 1.361858231 | 6.33E-07 | 1.52E-06 |
| NCAM2 | 0.305130659 | 0.151014942 | -1.014735846 | 1.49E-14 | 9.63E-14 |
| CAPN12 | 0.846194166 | 2.496677651 | 1.560948922 | 6.66E-07 | 1.60E-06 |
| ODAM | 0.022418128 | 3.936933699 | 7.456262821 | 0.000931794 | 0.001505881 |
| GPR65 | 0.853488109 | 0.379834652 | -1.167999526 | 4.61E-17 | 4.41E-16 |
| CYP27C1 | 0.049770282 | 0.161200939 | 1.695503678 | 0.001287283 | 0.002033874 |
| TM6SF2 | 13.53306002 | 6.514421439 | -1.05477913 | 3.65E-14 | 2.25E-13 |
| RTBDN | 0.006543737 | 0.254088477 | 5.279072427 | 8.20E-09 | 2.49E-08 |
| KIF24 | 0.09483912 | 0.384070087 | 2.017815427 | 2.33E-22 | 6.00E-21 |
| GPR61 | 0.012561987 | 0.034428503 | 1.454538737 | 2.68E-05 | 5.29E-05 |
| TMEM132E | 0.361695675 | 0.12244274 | -1.562669118 | 2.82E-19 | 3.86E-18 |
| OR4K17 | 0.000359113 | 7.52E-05 | -2.256166737 | 0.000834909 | 0.001358735 |
| HOXB8 | 0.003752853 | 0.081563459 | 4.441863373 | 1.53E-09 | 5.09E-09 |
| SCN7A | 0.238855283 | 0.077160842 | -1.630196007 | 2.78E-17 | 2.78E-16 |
| ERICH5 | 7.211345942 | 23.43422959 | 1.700276909 | 5.16E-05 | 9.84E-05 |
| MYOM2 | 0.94892662 | 0.233856032 | -2.020675887 | 2.05E-23 | 6.86E-22 |
| NUF2 | 0.097667956 | 1.968823922 | 4.333304973 | 2.81E-28 | 1.18E-25 |
| HPS5 | 11.65769248 | 4.417469754 | -1.399990088 | 2.35E-18 | 2.77E-17 |
| PABPC3 | 0.044508342 | 0.113774935 | 1.354035098 | 2.49E-07 | 6.31E-07 |
| CD69 | 2.093670185 | 0.808761034 | -1.372248797 | 1.91E-13 | 1.07E-12 |
| KAZALD1 | 0.255008391 | 0.881890875 | 1.790055431 | 3.53E-08 | 9.91E-08 |
| KCNQ3 | 0.010507326 | 0.044930492 | 2.096299323 | 2.41E-14 | 1.53E-13 |
| COL9A1 | 0.006378651 | 0.092782255 | 3.862525777 | 4.35E-22 | 1.05E-20 |
| OR51B6 | 0.001900465 | 0.000544674 | -1.802887901 | 0.00079919 | 0.001305248 |
| MECOM | 0.305161135 | 0.703236609 | 1.204438942 | 6.74E-10 | 2.34E-09 |
| FOLH1 | 7.044301695 | 2.955668473 | -1.252972242 | 5.56E-17 | 5.21E-16 |
| PTGES3L | 0.023632698 | 0.080680141 | 1.771429281 | 2.82E-11 | 1.17E-10 |
| KCNN2 | 4.852214973 | 0.46635778 | -3.37913438 | 1.03E-24 | 5.25E-23 |
| UCN | 0.638370947 | 1.624155427 | 1.347222801 | 2.68E-08 | 7.63E-08 |
| KRT20 | 0.034630181 | 4.415913218 | 6.994538068 | 2.06E-07 | 5.29E-07 |
| MAGEA11 | 0.000412946 | 0.181393663 | 8.778953597 | 0.019208562 | 0.025447463 |
| SYT9 | 1.487879842 | 0.296304 | -2.328108015 | 2.84E-22 | 7.20E-21 |
| TPPP2 | 2.364772942 | 0.578309446 | -2.031788095 | 2.31E-23 | 7.62E-22 |
| AC110275.1 | 0.005822537 | 0.002636915 | -1.142797051 | 0.002255691 | 0.003430502 |
| COL24A1 | 0.007665231 | 0.109382214 | 3.834905046 | 1.04E-10 | 3.98E-10 |
| AL360181.3 | 0.012295108 | 0.037426434 | 1.605973144 | 5.95E-06 | 1.27E-05 |
| CACNB4 | 0.009182945 | 0.087272706 | 3.248501668 | 5.78E-12 | 2.63E-11 |
| TYMS | 2.297916169 | 8.278716041 | 1.849080868 | 4.37E-16 | 3.52E-15 |
| FBF1 | 0.160937503 | 0.465625045 | 1.532668105 | 2.11E-19 | 2.94E-18 |
| SULT1C2 | 0.175167561 | 2.126727824 | 3.601827879 | 5.73E-12 | 2.61E-11 |
| CKAP2L | 0.093973309 | 0.923656436 | 3.297033364 | 5.65E-25 | 3.14E-23 |
| GAREM2 | 0.090976327 | 0.43374215 | 2.25327455 | 8.07E-14 | 4.74E-13 |
| ITGAD | 0.705927071 | 0.350962506 | -1.008202234 | 7.00E-10 | 2.43E-09 |
| ABLIM2 | 0.317347639 | 0.892011405 | 1.490998044 | 7.19E-08 | 1.94E-07 |
| FAM110C | 7.960412302 | 3.628658735 | -1.133406774 | 1.45E-19 | 2.08E-18 |
| CHEK1 | 0.385662283 | 1.220739382 | 1.662345262 | 4.18E-19 | 5.59E-18 |
| SP5 | 0.926501346 | 5.067984572 | 2.451547156 | 1.78E-05 | 3.60E-05 |
| ACBD7 | 0.041670256 | 0.137946929 | 1.727023463 | 2.54E-06 | 5.68E-06 |
| TMEM71 | 0.38452534 | 0.17954165 | -1.098760115 | 9.17E-19 | 1.15E-17 |
| TRIM73 | 0.022522465 | 0.065490819 | 1.539927964 | 2.92E-05 | 5.75E-05 |
| KCNJ15 | 0.410517154 | 0.159438069 | -1.364446366 | 6.71E-17 | 6.19E-16 |
| OR9G1 | 0.000503034 | 0.000159688 | -1.655402168 | 0.00094426 | 0.001524575 |
| FBXL19 | 0.934149369 | 1.977186751 | 1.081723987 | 8.97E-23 | 2.53E-21 |
| MAST1 | 0.128857167 | 0.354045038 | 1.458160108 | 8.95E-05 | 0.000165435 |
| KIAA1211L | 0.213216572 | 0.554207217 | 1.378105924 | 8.93E-13 | 4.56E-12 |
| AC023509.3 | 0.169924117 | 0.368124379 | 1.115302674 | 4.76E-12 | 2.20E-11 |
| B4GALNT2 | 0.002882576 | 0.505741662 | 7.454898189 | 2.09E-08 | 6.03E-08 |
| COX7B2 | 0.009517548 | 6.810145995 | 9.48288001 | 1.77E-06 | 4.04E-06 |
| RCOR2 | 0.058618306 | 0.323859321 | 2.465944075 | 0.000252887 | 0.000441623 |
| PRR18 | 2.935128214 | 1.117144479 | -1.393607746 | 3.74E-17 | 3.63E-16 |
| VIPR1 | 4.512085792 | 0.429721352 | -3.39232113 | 2.16E-29 | 3.13E-26 |
| LPA | 17.50756613 | 3.81421179 | -2.198521678 | 1.48E-24 | 7.13E-23 |
| GPR156 | 0.005156829 | 0.0105651 | 1.034750306 | 0.009286095 | 0.012900637 |
| SEMA4F | 0.326033299 | 0.86377714 | 1.405639818 | 9.18E-10 | 3.13E-09 |
| HIST1H4A | 0.016203712 | 0.065985723 | 2.025829538 | 0.010721673 | 0.014755222 |
| RNF180 | 1.210929758 | 0.544626081 | -1.152777205 | 5.67E-19 | 7.39E-18 |
| CDH11 | 0.190754807 | 0.597835491 | 1.64802913 | 0.000205499 | 0.000362698 |
| NFE2L3 | 0.862402819 | 1.847808616 | 1.099381541 | 3.36E-06 | 7.41E-06 |
| LIMK2 | 2.499030375 | 5.185867388 | 1.053216877 | 7.82E-09 | 2.38E-08 |
| RNF165 | 0.396706109 | 0.123009922 | -1.689295932 | 4.25E-20 | 6.76E-19 |
| SCX | 0.109184286 | 1.100362469 | 3.333141697 | 5.12E-24 | 2.04E-22 |
| EFCAB8 | 0.00951398 | 0.022916427 | 1.26826122 | 0.000185971 | 0.000330217 |
| OR10AG1 | 0.001790734 | 0.000281115 | -2.671320369 | 0.002485911 | 0.003758217 |
| CFAP61 | 0.003300866 | 0.026971929 | 3.030542104 | 4.15E-07 | 1.02E-06 |
| PLP2 | 10.05021268 | 32.81258851 | 1.707023377 | 6.69E-08 | 1.82E-07 |
| CU639417.2 | 6.492298014 | 3.112838084 | -1.060498689 | 7.39E-10 | 2.56E-09 |
| LINC00672 | 0.080565565 | 0.23013844 | 1.514266734 | 5.23E-13 | 2.76E-12 |
| LAIR2 | 0.085611849 | 0.34845241 | 2.025079239 | 6.96E-07 | 1.66E-06 |
| TRIM16L | 1.139286934 | 5.792880174 | 2.346149681 | 1.20E-05 | 2.47E-05 |
| OR10G6 | 0.000621672 | 0.000160868 | -1.950278706 | 0.002153829 | 0.003284579 |
| DENND4B | 2.321002997 | 4.924391369 | 1.08519704 | 3.10E-24 | 1.34E-22 |
| FAT4 | 0.809726115 | 0.354207511 | -1.192839203 | 1.07E-17 | 1.14E-16 |
| ECEL1 | 0.039839756 | 3.45756437 | 6.43940348 | 0.002152991 | 0.003283623 |
| EPB41L4B | 16.22034547 | 5.956423793 | -1.445286238 | 2.03E-24 | 9.32E-23 |
| CLVS1 | 0.013730594 | 0.104758363 | 2.931599488 | 2.65E-10 | 9.70E-10 |
| PDE1C | 0.011983279 | 0.072503663 | 2.597031132 | 7.54E-15 | 5.08E-14 |
| ANKRD29 | 0.408474051 | 1.342623812 | 1.716738798 | 2.85E-09 | 9.13E-09 |
| IFNL2 | 0.01440573 | 0.004387591 | -1.715141759 | 0.0028777 | 0.004309073 |
| AC087289.3 | 0.003367804 | 0.011361795 | 1.754310515 | 2.54E-07 | 6.43E-07 |
| OR8J3 | 0.000713954 | 2.63E-05 | -4.764781792 | 0.000800203 | 0.001306354 |
| IFNA17 | 0.000344062 | 0.000117381 | -1.551472504 | 0.0011245 | 0.001794909 |
| SLC4A3 | 0.076660394 | 0.543313004 | 2.825230251 | 0.014647467 | 0.019771412 |
| NCAPH | 0.209631344 | 1.784411477 | 3.089521982 | 1.34E-26 | 1.45E-24 |
| OR4A15 | 0.000641228 | 8.24E-05 | -2.959500527 | 0.001212778 | 0.001924365 |
| RNF222 | 0.002187581 | 0.00560153 | 1.356484456 | 0.003903892 | 0.005733669 |
| INPP5J | 0.057746551 | 0.256767675 | 2.152656914 | 2.35E-09 | 7.59E-09 |
| MYL4 | 0.089535719 | 0.183087201 | 1.031995693 | 4.56E-06 | 9.89E-06 |
| OR1L1 | 0.00083522 | 6.53E-05 | -3.677641202 | 0.000364317 | 0.000623478 |
| CENPK | 0.091138184 | 0.620051569 | 2.766260686 | 4.46E-24 | 1.80E-22 |
| P3H2 | 1.7074239 | 0.701944244 | -1.282392934 | 1.05E-23 | 3.84E-22 |
| GBA | 8.090117389 | 21.71373704 | 1.424375501 | 6.67E-24 | 2.57E-22 |
| CA12 | 0.473605605 | 3.668869041 | 2.953577349 | 6.25E-08 | 1.70E-07 |
| SHCBP1 | 0.080080762 | 0.896141773 | 3.484199377 | 1.91E-26 | 1.93E-24 |
| SCAMP5 | 1.430346771 | 3.787093416 | 1.404726055 | 1.39E-07 | 3.62E-07 |
| BCL9 | 1.23876232 | 3.192259925 | 1.36567872 | 2.18E-18 | 2.58E-17 |
| CENPU | 0.723787165 | 3.371001543 | 2.219539859 | 9.46E-22 | 2.12E-20 |
| APLP1 | 0.15531727 | 1.302942457 | 3.06848321 | 0.000456424 | 0.000770095 |
| TMEM81 | 1.252192499 | 2.544401559 | 1.022870012 | 5.36E-15 | 3.68E-14 |
| KIR2DL1 | 0.063538437 | 0.025786159 | -1.301032707 | 2.28E-06 | 5.12E-06 |
| CAD | 1.598732209 | 3.910404216 | 1.290389441 | 1.32E-23 | 4.73E-22 |
| SAPCD1 | 0.036692918 | 0.317738691 | 3.114267239 | 4.12E-22 | 9.95E-21 |
| TMEM191B | 0.032601468 | 0.091352797 | 1.48651196 | 3.41E-05 | 6.67E-05 |
| BANF2 | 0.004326319 | 0.198424608 | 5.519307051 | 1.70E-11 | 7.30E-11 |
| MAGEC1 | 0.002179177 | 1.048921409 | 8.910907406 | 6.41E-05 | 0.000120812 |
| GPR1 | 0.010189667 | 0.0938448 | 3.203169927 | 1.71E-05 | 3.45E-05 |
| SPRYD4 | 11.48918907 | 4.913703291 | -1.225394324 | 1.80E-23 | 6.14E-22 |
| HIST1H2BM | 0.004564677 | 0.042309675 | 3.212402866 | 0.00026451 | 0.000460575 |
| LMO1 | 0.001794182 | 0.038752128 | 4.432877505 | 0.049143144 | 0.06125322 |
| MFSD10 | 3.688136941 | 8.427785816 | 1.192261426 | 3.23E-09 | 1.03E-08 |
| SPRR2F | 0.010654525 | 0.004744869 | -1.167025912 | 0.006221601 | 0.008864073 |
| TAGLN2 | 69.41198989 | 178.4000797 | 1.361859466 | 6.43E-17 | 5.96E-16 |
| SIRPB1 | 0.268914636 | 0.126727292 | -1.085421023 | 5.78E-13 | 3.02E-12 |
| KLF6 | 40.9889891 | 19.53600147 | -1.069101195 | 1.77E-14 | 1.14E-13 |
| ZNF467 | 1.861165225 | 4.125554733 | 1.148381985 | 0.000360027 | 0.000616543 |
| C1QTNF3-AMACR | 0.005555008 | 0.014232361 | 1.357313974 | 0.00024453 | 0.000427844 |
| DRC7 | 0.075569764 | 0.20075498 | 1.409554747 | 3.13E-06 | 6.93E-06 |
| ZNF738 | 0.107714689 | 0.346252866 | 1.684611004 | 6.75E-11 | 2.67E-10 |
| GP6 | 0.0778302 | 0.033297766 | -1.224904686 | 1.61E-13 | 9.08E-13 |
| AGAP5 | 0.026458247 | 0.05398816 | 1.028925587 | 6.78E-06 | 1.44E-05 |
| LRCOL1 | 8.24223719 | 3.16131769 | -1.382509958 | 3.59E-16 | 2.92E-15 |
| PCNX2 | 0.098583072 | 0.287679677 | 1.54505146 | 4.89E-07 | 1.19E-06 |
| MYCNOS | 0.007892887 | 0.063938714 | 3.018064815 | 0.000156965 | 0.000281344 |
| CETP | 17.16535606 | 2.800747939 | -2.615615728 | 9.97E-26 | 7.39E-24 |
| SLC16A8 | 0.07110418 | 0.193482572 | 1.444197348 | 2.21E-07 | 5.65E-07 |
| NRXN3 | 0.07325647 | 0.225174563 | 1.620015769 | 0.041988069 | 0.052804988 |
| GNAL | 0.166478643 | 0.726679853 | 2.125982798 | 1.76E-12 | 8.63E-12 |
| GTF2IRD1 | 0.963880341 | 2.064643179 | 1.098966507 | 6.09E-15 | 4.15E-14 |
| CD24 | 9.60319814 | 48.35069834 | 2.331949877 | 6.80E-05 | 0.000127687 |
| RSPH10B2 | 0.006188166 | 0.002552632 | -1.277525947 | 1.25E-05 | 2.56E-05 |
| BOP1 | 6.78235006 | 20.63150345 | 1.604991803 | 3.78E-19 | 5.08E-18 |
| CCNB1 | 0.877565425 | 8.641249583 | 3.29966136 | 3.74E-27 | 5.65E-25 |
| OR4K5 | 0.000952822 | 0.000223454 | -2.092227882 | 0.00119756 | 0.001901575 |
| OR56B4 | 0.009647201 | 0.024681329 | 1.355237763 | 0.005221441 | 0.007517338 |
| PCK1 | 309.4074033 | 98.61411471 | -1.649641657 | 3.33E-20 | 5.41E-19 |
| SOX2 | 0.030247967 | 0.360555385 | 3.575310792 | 0.023990712 | 0.031286462 |
| MKRN2OS | 0.081511007 | 0.250976538 | 1.622485705 | 0.001194022 | 0.001896935 |
| MIOX | 0.036910671 | 0.697578834 | 4.240246393 | 4.13E-10 | 1.48E-09 |
| NR0B1 | 0.002675167 | 0.366909644 | 7.099652001 | 0.002629045 | 0.003966898 |
| CKAP2 | 1.056620384 | 2.522083725 | 1.255159022 | 4.31E-16 | 3.47E-15 |
| ZNF556 | 0.007273019 | 0.041146489 | 2.500143045 | 0.01328631 | 0.018029517 |
| ADH1A | 380.1177229 | 164.4479222 | -1.208815513 | 3.66E-17 | 3.56E-16 |
| HOGA1 | 14.89792231 | 4.60201981 | -1.694772045 | 7.94E-24 | 3.01E-22 |
| ZNF534 | 0.005881528 | 0.042663591 | 2.858742452 | 0.032860495 | 0.041998159 |
| PADI3 | 0.002824046 | 0.831523895 | 8.201850315 | 3.48E-05 | 6.79E-05 |
| HIST1H4E | 0.10598898 | 0.675049051 | 2.671078064 | 1.23E-14 | 8.12E-14 |
| CA10 | 0.001536297 | 0.01519889 | 3.306436649 | 0.001820363 | 0.002804913 |
| DUSP9 | 0.312782054 | 10.6896588 | 5.094914253 | 1.29E-19 | 1.86E-18 |
| PGBD1 | 0.760640862 | 1.584864067 | 1.059071757 | 3.13E-07 | 7.82E-07 |
| LRRC71 | 0.015585653 | 0.033365242 | 1.098127379 | 2.19E-05 | 4.37E-05 |
| ADGRG1 | 1.41465968 | 3.175952426 | 1.166734271 | 0.002844117 | 0.004262066 |
| ABI3BP | 0.736414457 | 0.366384742 | -1.007158526 | 1.91E-14 | 1.22E-13 |
| E2F8 | 0.056604633 | 0.814860146 | 3.847560433 | 1.78E-25 | 1.22E-23 |
| SLC4A9 | 0.006754759 | 0.020124632 | 1.57498619 | 8.46E-05 | 0.000156831 |
| PTGER3 | 0.064968047 | 0.137068647 | 1.077096371 | 0.004829115 | 0.006989634 |
| CST2 | 0.058896094 | 0.54818016 | 3.21840626 | 4.29E-06 | 9.33E-06 |
| HIST1H4B | 0.056286769 | 0.129010219 | 1.196617609 | 0.004550149 | 0.006609926 |
| HES6 | 1.484965555 | 3.880491758 | 1.385810024 | 3.51E-14 | 2.17E-13 |
| CHML | 0.581508944 | 1.943623526 | 1.740875514 | 7.17E-16 | 5.60E-15 |
| KIRREL2 | 0.004226014 | 1.065372117 | 7.977844244 | 0.00012492 | 0.00022723 |
| TACR1 | 0.02084202 | 0.202765567 | 3.282245645 | 0.0115446 | 0.015809425 |
| CCDC71L | 15.16989355 | 5.163172133 | -1.554881361 | 1.33E-14 | 8.71E-14 |
| BICDL1 | 0.472420384 | 2.354598608 | 2.317338023 | 2.66E-16 | 2.21E-15 |
| ATP6V1F | 38.37324874 | 76.83014882 | 1.001571637 | 3.41E-21 | 6.73E-20 |
| SCN4A | 0.04971012 | 0.245456126 | 2.30385369 | 1.48E-15 | 1.10E-14 |
| MOGAT3 | 5.140519208 | 10.75159579 | 1.064564817 | 0.001717125 | 0.002657683 |
| HLA-A | 232.6880416 | 491.317509 | 1.078258584 | 1.04E-10 | 3.98E-10 |
| LRRC14 | 1.621536374 | 4.521720251 | 1.479510352 | 3.98E-28 | 1.33E-25 |
| SFRP4 | 0.176546584 | 1.522750459 | 3.108558729 | 8.62E-13 | 4.42E-12 |
| DCLK1 | 0.038967674 | 0.094530081 | 1.278495665 | 0.00085323 | 0.001385521 |
| GPSM2 | 0.203205086 | 0.630134925 | 1.632724262 | 4.65E-22 | 1.10E-20 |
| LRGUK | 0.037279778 | 0.107790627 | 1.531766583 | 1.99E-08 | 5.75E-08 |
| UTS2R | 0.003793271 | 0.048936561 | 3.689398441 | 0.004602656 | 0.006681732 |
| CATIP | 0.051477197 | 0.113782255 | 1.144270186 | 0.000346753 | 0.000594339 |
| KLK12 | 0.013994701 | 0.005399956 | -1.373861219 | 0.034696224 | 0.044173801 |
| PSMC3IP | 0.229102507 | 0.723740809 | 1.659479875 | 3.49E-21 | 6.87E-20 |
| TTYH2 | 0.407858392 | 0.923729519 | 1.179402135 | 8.29E-05 | 0.000153803 |
| DPYSL5 | 0.000445751 | 0.015132123 | 5.085231708 | 0.009621757 | 0.013339449 |
| RAD54L | 0.067718283 | 0.771156057 | 3.509405537 | 3.15E-25 | 1.93E-23 |
| FOXD2 | 0.125142809 | 0.559381609 | 2.160257429 | 7.13E-16 | 5.57E-15 |
| NPM2 | 0.546648387 | 2.015950191 | 1.882774922 | 0.0040436 | 0.005924876 |
| ZNF730 | 0.005746966 | 0.043524647 | 2.920960234 | 3.83E-05 | 7.43E-05 |
| CKS2 | 10.38649302 | 29.41233298 | 1.50171261 | 6.23E-16 | 4.90E-15 |
| CLEC9A | 0.219215736 | 0.098648443 | -1.15198318 | 2.74E-10 | 1.00E-09 |
| AHSA2 | 0.986935785 | 2.552543858 | 1.370907626 | 1.53E-19 | 2.18E-18 |
| PLEKHG4 | 0.080017661 | 0.417833659 | 2.384538354 | 4.14E-06 | 9.02E-06 |
| KIF18A | 0.050082713 | 0.570855096 | 3.51073995 | 3.20E-26 | 2.98E-24 |
| CLK2 | 3.959568242 | 8.562152282 | 1.11263037 | 4.19E-24 | 1.72E-22 |
| MAT1A | 479.7912703 | 205.5321998 | -1.223042476 | 4.40E-22 | 1.06E-20 |
| HIST1H3E | 0.498954504 | 1.636416764 | 1.713560043 | 2.99E-10 | 1.09E-09 |
| FCGR2B | 2.538332843 | 0.778543136 | -1.705032376 | 8.88E-24 | 3.34E-22 |
| SPINK1 | 24.71458005 | 222.2640219 | 3.16884014 | 7.02E-06 | 1.49E-05 |
| LCE3B | 0.001667179 | 2.63E-05 | -5.988286828 | 0.00081997 | 0.001336242 |
| NUPR1 | 20.90641424 | 47.05179687 | 1.17030418 | 4.44E-07 | 1.09E-06 |
| RIT2 | 0.013479204 | 0.004491732 | -1.585391685 | 1.12E-05 | 2.32E-05 |
| CDCA8 | 0.373286379 | 3.495514281 | 3.227149957 | 4.08E-27 | 6.04E-25 |
| EXO1 | 0.085283228 | 1.158402723 | 3.76373105 | 6.08E-27 | 7.94E-25 |
| KHDC1 | 0.018117242 | 0.069324533 | 1.936002645 | 0.011585045 | 0.015863416 |
| ACPT | 0.02117023 | 0.377040396 | 4.154610226 | 2.30E-10 | 8.48E-10 |
| HRCT1 | 1.329054784 | 8.713349599 | 2.712826855 | 9.89E-12 | 4.37E-11 |
| MAP2 | 0.649693727 | 1.954320949 | 1.588835732 | 4.63E-05 | 8.88E-05 |
| GGT5 | 10.34137596 | 4.251440309 | -1.282404568 | 7.86E-20 | 1.18E-18 |
| KRTAP1-1 | 0.117289778 | 0.032064951 | -1.8710082 | 5.62E-05 | 0.000106772 |
| PDZK1IP1 | 5.362005474 | 45.83970436 | 3.095753142 | 0.003498004 | 0.005172697 |
| TXN | 116.0395321 | 261.3116483 | 1.171155053 | 1.12E-15 | 8.51E-15 |
| GJA10 | 0.000624922 | 0.254639987 | 8.670566257 | 0.006013913 | 0.00858547 |
| C5orf58 | 0.031503666 | 0.318984049 | 3.33989266 | 0.00274853 | 0.004132357 |
| CTNNA2 | 0.01802859 | 1.109932632 | 5.944041702 | 0.000252276 | 0.000440661 |
| TSSK6 | 0.451435613 | 1.190281226 | 1.398710337 | 8.35E-19 | 1.05E-17 |
| PCDHB10 | 0.079690598 | 0.196268435 | 1.300346738 | 0.002122201 | 0.003239839 |
| CACNA1F | 0.01392597 | 0.02850412 | 1.033392576 | 0.032310899 | 0.041333171 |
| SLC9B2 | 10.81941474 | 4.22660691 | -1.356050614 | 4.52E-24 | 1.82E-22 |
| SYNGR4 | 0.056302917 | 0.275197949 | 2.289188135 | 1.23E-07 | 3.23E-07 |
| TSPEAR | 0.047987615 | 0.198890067 | 2.051237198 | 3.73E-06 | 8.19E-06 |
| PHACTR3 | 0.110083003 | 0.024764543 | -2.152243802 | 7.31E-29 | 5.99E-26 |
| EPHX1 | 499.2869206 | 1066.136218 | 1.094450761 | 8.15E-07 | 1.93E-06 |
| OTUB2 | 0.191276283 | 0.59895375 | 1.646786602 | 3.12E-18 | 3.59E-17 |
| ANKRD52 | 1.318445732 | 3.602030017 | 1.449972014 | 1.52E-25 | 1.06E-23 |
| GRXCR1 | 0.001529256 | 0.000371237 | -2.042416999 | 0.000431871 | 0.000730888 |
| UGT2B11 | 1.399378954 | 13.52758735 | 3.273045953 | 9.09E-08 | 2.42E-07 |
| TMEM61 | 0.067000413 | 0.363049451 | 2.437924189 | 0.004737519 | 0.006864704 |
| KBTBD11 | 3.756700371 | 0.767593531 | -2.291051594 | 1.92E-25 | 1.29E-23 |
| VWA5B2 | 0.017986709 | 0.097246986 | 2.434722267 | 3.35E-08 | 9.44E-08 |
| NR3C2 | 2.553225076 | 1.016612574 | -1.328550742 | 1.90E-21 | 3.94E-20 |
| GLS2 | 6.999738591 | 1.8815221 | -1.895400809 | 7.90E-19 | 9.99E-18 |
| ANK3 | 0.638789735 | 0.294933184 | -1.114952974 | 8.90E-20 | 1.32E-18 |
| TSPO2 | 0.176140393 | 0.81973546 | 2.218432615 | 2.29E-10 | 8.45E-10 |
| DNASE1 | 0.218650704 | 0.484189071 | 1.14694252 | 5.12E-17 | 4.84E-16 |
| REEP2 | 0.043982164 | 0.396106765 | 3.170898849 | 1.96E-16 | 1.68E-15 |
| TPH1 | 0.015987901 | 0.060494422 | 1.919821605 | 2.81E-08 | 7.99E-08 |
| B3GNT3 | 1.788247011 | 7.636113201 | 2.09429246 | 0.015727683 | 0.021125036 |
| EIF5A2 | 0.458305465 | 1.203794534 | 1.393207777 | 0.003617056 | 0.005336066 |
| ST6GALNAC5 | 0.010639158 | 0.074476721 | 2.807405531 | 0.000119715 | 0.000218221 |
| DUSP13 | 0.003237185 | 0.187400272 | 5.85523944 | 2.10E-09 | 6.86E-09 |
| GLP2R | 0.293889489 | 0.052832032 | -2.475788953 | 3.36E-24 | 1.43E-22 |
| GADD45B | 149.0182795 | 52.3507671 | -1.509206729 | 6.84E-18 | 7.52E-17 |
| XG | 0.68775294 | 0.29483505 | -1.221982359 | 3.44E-12 | 1.62E-11 |
| EFNA1 | 57.05639481 | 124.2378463 | 1.122644229 | 5.17E-12 | 2.37E-11 |
| KCNJ14 | 0.111431702 | 0.251353498 | 1.173558031 | 3.65E-08 | 1.02E-07 |
| LYPD8 | 0.016867267 | 0.611239977 | 5.179440726 | 1.76E-12 | 8.63E-12 |
| COL4A1 | 7.276897798 | 28.79462951 | 1.984404306 | 2.98E-18 | 3.46E-17 |
| GSDMC | 0.012705659 | 0.203791799 | 4.003552908 | 5.12E-18 | 5.76E-17 |
| BDKRB1 | 0.040131787 | 0.308560407 | 2.942735638 | 1.59E-13 | 8.98E-13 |
| FLNC | 0.251308121 | 2.503215477 | 3.316253286 | 0.028815121 | 0.037191442 |
| ASS1 | 561.0151421 | 246.3456456 | -1.18735574 | 7.43E-19 | 9.48E-18 |
| ISL2 | 0.010710384 | 0.202234527 | 4.238947172 | 8.02E-13 | 4.13E-12 |
| ZAN | 0.014852715 | 0.006424734 | -1.209018175 | 2.82E-07 | 7.08E-07 |
| FOXI2 | 0.004037516 | 0.020333681 | 2.332331514 | 2.11E-05 | 4.22E-05 |
| ST6GAL2 | 0.583806089 | 0.12242104 | -2.253637734 | 2.09E-25 | 1.38E-23 |
| TSSK3 | 0.048917513 | 0.098020904 | 1.002738394 | 6.27E-06 | 1.34E-05 |
| IL10 | 0.358564067 | 0.121526656 | -1.560958117 | 2.75E-18 | 3.22E-17 |
| AQP5 | 0.025059114 | 0.18634763 | 2.89458918 | 0.000315552 | 0.000543737 |
| NANOS1 | 0.057661666 | 0.406672903 | 2.818184439 | 5.12E-14 | 3.10E-13 |
| IL13RA2 | 3.016187199 | 1.175056339 | -1.359996042 | 1.23E-23 | 4.42E-22 |
| PLA2G2C | 0.009592576 | 0.021765681 | 1.182064945 | 0.010554622 | 0.014543336 |
| OR1E1 | 0.00524988 | 0.001206549 | -2.121398398 | 0.000190231 | 0.000337432 |
| PLVAP | 5.799740648 | 44.78294654 | 2.948889162 | 5.21E-29 | 4.80E-26 |
| ZAR1L | 0.004823339 | 0.016362515 | 1.762290309 | 0.014923162 | 0.020123478 |
| C6orf163 | 0.045063796 | 0.097504644 | 1.113502079 | 5.31E-07 | 1.29E-06 |
| FGF23 | 0.3330126 | 0.024603269 | -3.758654863 | 2.73E-20 | 4.55E-19 |
| TCF23 | 0.019141321 | 0.054688195 | 1.514539057 | 0.004524008 | 0.006575535 |
| CABP1 | 0.016930026 | 0.059480876 | 1.812841736 | 6.19E-11 | 2.46E-10 |
| EFHD1 | 14.13353145 | 6.361882882 | -1.151596269 | 8.24E-10 | 2.83E-09 |
| INCENP | 0.823945441 | 1.989992792 | 1.27214249 | 3.74E-17 | 3.63E-16 |
| TBC1D16 | 1.197625332 | 3.899670819 | 1.703175704 | 5.87E-25 | 3.24E-23 |
| UBE2C | 0.686891527 | 12.58816858 | 4.195842306 | 8.37E-27 | 1.02E-24 |
| CFAP74 | 0.132679444 | 0.050634374 | -1.389755853 | 5.91E-05 | 0.00011193 |
| CHN1 | 0.315939767 | 0.714663945 | 1.177615469 | 1.37E-06 | 3.16E-06 |
| NDUFA4L2 | 1.374883376 | 10.65446437 | 2.954076914 | 2.36E-25 | 1.52E-23 |
| TTLL3 | 0.260051577 | 0.54845934 | 1.076586882 | 4.26E-15 | 2.96E-14 |
| SRRM5 | 0.090586983 | 0.187646183 | 1.050639286 | 1.32E-10 | 5.02E-10 |
| TRPC5OS | 0.008324272 | 0.033593228 | 2.012774516 | 0.019240304 | 0.025484935 |
| TMPRSS2 | 12.48473768 | 5.626474646 | -1.149862342 | 4.89E-14 | 2.97E-13 |
| CCBE1 | 1.63236489 | 0.136171933 | -3.583462311 | 1.81E-28 | 9.22E-26 |
| AL121594.3 | 0.014289006 | 0.032047106 | 1.165288494 | 1.56E-05 | 3.18E-05 |
| C16orf74 | 0.183439505 | 0.421891658 | 1.201568197 | 0.035032733 | 0.044554878 |
| SIX1 | 0.007183024 | 0.227120379 | 4.982721955 | 1.03E-21 | 2.28E-20 |
| EBF3 | 0.041886777 | 0.219604899 | 2.390343448 | 4.11E-17 | 3.95E-16 |
| HHIP | 1.678686793 | 0.159479023 | -3.395894505 | 1.84E-28 | 9.22E-26 |
| EYA2 | 0.868584795 | 0.346459666 | -1.325979291 | 1.33E-13 | 7.60E-13 |
| GGH | 29.87109699 | 62.01017373 | 1.053754711 | 0.000870352 | 0.001411995 |
| SOCS7 | 0.364076906 | 1.059092155 | 1.540512993 | 6.18E-23 | 1.85E-21 |
| LIPN | 0.047269816 | 0.015927177 | -1.569428669 | 5.40E-10 | 1.90E-09 |
| CEACAM4 | 0.344810858 | 0.158412392 | -1.122120005 | 6.98E-11 | 2.75E-10 |
| FAM166A | 0.048508163 | 0.192414636 | 1.987919088 | 2.52E-06 | 5.63E-06 |
| ADGRA3 | 11.12439324 | 5.240183952 | -1.086037287 | 8.05E-17 | 7.31E-16 |
| LMNA | 27.74419037 | 56.60757106 | 1.028809318 | 3.13E-19 | 4.27E-18 |
| GPC3 | 4.033294543 | 291.4724114 | 6.175256759 | 4.35E-19 | 5.78E-18 |
| FBLN1 | 2.33762641 | 7.191634475 | 1.621275313 | 0.023723577 | 0.030964045 |
| MEP1B | 0.839195188 | 0.227795862 | -1.881264864 | 3.92E-17 | 3.78E-16 |
| ANGPTL6 | 10.72946477 | 1.473132861 | -2.864618653 | 4.85E-30 | 2.52E-26 |
| KCNF1 | 0.127327464 | 0.680412239 | 2.417865461 | 2.41E-06 | 5.40E-06 |
| SLC17A8 | 0.151265186 | 0.026205061 | -2.529162594 | 1.48E-23 | 5.21E-22 |
| FGF6 | 0.000935067 | 0.000408098 | -1.196154774 | 0.00233852 | 0.003548838 |
| CLTCL1 | 0.638006494 | 1.416017303 | 1.150195881 | 1.85E-07 | 4.77E-07 |
| CPED1 | 6.120935503 | 1.566992833 | -1.965753585 | 2.02E-25 | 1.34E-23 |
| GINS1 | 0.31776033 | 2.179459804 | 2.777959665 | 1.03E-23 | 3.79E-22 |
| AKR1D1 | 60.43760164 | 20.26902217 | -1.576169919 | 1.32E-18 | 1.62E-17 |
| WFS1 | 4.369119693 | 9.044213411 | 1.049652406 | 3.12E-18 | 3.59E-17 |
| ARL17B | 0.044368134 | 0.091057529 | 1.037254416 | 4.43E-05 | 8.52E-05 |
| EPPK1 | 0.063734248 | 0.684107508 | 3.42408234 | 4.23E-09 | 1.33E-08 |
| MCM3 | 6.321509774 | 20.08965937 | 1.668112038 | 7.65E-24 | 2.92E-22 |
| ADRB1 | 0.4753462 | 0.198636847 | -1.258845361 | 7.55E-12 | 3.39E-11 |
| RAD51 | 0.209674095 | 1.098507384 | 2.389324035 | 3.47E-23 | 1.10E-21 |
| AC009086.2 | 0.001033323 | 0.000244634 | -2.07859431 | 0.001559255 | 0.002429533 |
| SHC1 | 17.03106151 | 35.3726907 | 1.054467606 | 3.09E-20 | 5.09E-19 |
| ZNF618 | 0.687902206 | 1.43332861 | 1.059094017 | 2.39E-14 | 1.51E-13 |
| UBAP2L | 6.761716615 | 14.42232335 | 1.092842134 | 4.04E-24 | 1.67E-22 |
| ETFDH | 30.6414335 | 11.01075608 | -1.476570254 | 6.24E-26 | 4.93E-24 |
| DPF1 | 0.007572469 | 0.042283192 | 2.481248674 | 2.16E-13 | 1.20E-12 |
| KRT81 | 0.066293081 | 0.346116202 | 2.384326266 | 0.001320774 | 0.002082136 |
| HOXD9 | 0.039365657 | 1.10615791 | 4.812475987 | 7.44E-19 | 9.48E-18 |
| HAO2 | 101.6176561 | 25.80196348 | -1.977598331 | 3.44E-22 | 8.51E-21 |
| SHISA6 | 0.006034042 | 0.019787405 | 1.713385763 | 0.00225697 | 0.003432112 |
| SLC16A4 | 3.370661038 | 1.149574732 | -1.551931297 | 1.94E-17 | 2.00E-16 |
| RGS17 | 0.019335477 | 0.057101967 | 1.562290116 | 6.69E-14 | 3.99E-13 |
| CPLX1 | 1.542005648 | 4.359162153 | 1.499242821 | 0.000580437 | 0.000966754 |
| SEMA3F | 1.771521885 | 4.308605523 | 1.28223173 | 3.44E-26 | 3.18E-24 |
| PXDNL | 0.016162989 | 0.138692295 | 3.101121706 | 6.70E-20 | 1.02E-18 |
| SEZ6L2 | 0.828761723 | 9.102976394 | 3.457309062 | 0.000417028 | 0.000708001 |
| RFC3 | 1.391840794 | 2.92870738 | 1.073269856 | 3.41E-16 | 2.80E-15 |
| ZFPM2 | 0.530843741 | 0.205410521 | -1.369777173 | 5.81E-23 | 1.75E-21 |
| ALPK3 | 0.393646827 | 1.654203545 | 2.07116301 | 8.97E-08 | 2.40E-07 |
| SOGA1 | 0.393355365 | 1.133874476 | 1.527355771 | 1.93E-22 | 5.07E-21 |
| HGF | 3.989175056 | 0.980344419 | -2.024729837 | 2.02E-24 | 9.29E-23 |
| ADGRB3 | 0.127371986 | 0.031237869 | -2.027680045 | 1.69E-24 | 8.02E-23 |
| MATN1 | 0.010570223 | 0.028565252 | 1.434255458 | 0.000195267 | 0.000345816 |
| C9orf172 | 0.328405564 | 1.221605276 | 1.895227723 | 2.98E-23 | 9.53E-22 |
| DLX4 | 0.013061915 | 0.087643134 | 2.746274615 | 4.34E-05 | 8.36E-05 |
| PIK3R2 | 0.132737911 | 0.340323741 | 1.358327323 | 2.61E-10 | 9.56E-10 |
| KCNJ9 | 0.001769248 | 0.02288743 | 3.693347352 | 3.74E-05 | 7.27E-05 |
| TMEM59L | 0.036635019 | 0.115112966 | 1.651755076 | 0.017392087 | 0.023190443 |
| MCF2L2 | 0.00817939 | 0.054653241 | 2.740241876 | 2.75E-09 | 8.81E-09 |
| ZIC2 | 0.015146118 | 1.757893424 | 6.858755719 | 6.38E-24 | 2.49E-22 |
| TMEM266 | 0.065823688 | 0.295202309 | 2.165025244 | 1.42E-15 | 1.06E-14 |
| EGFL6 | 0.006961666 | 0.135457337 | 4.282262143 | 9.26E-20 | 1.36E-18 |
| LIMK1 | 1.07526038 | 2.574989256 | 1.259880354 | 1.00E-14 | 6.68E-14 |
| SIPA1L2 | 1.886233841 | 4.25208008 | 1.172660226 | 0.000595104 | 0.000988853 |
| FAM71B | 0.000825755 | 0.000228568 | -1.853091069 | 0.000147559 | 0.000265694 |
| GHRHR | 0.040812957 | 0.576382943 | 3.819928506 | 0.000287688 | 0.000498091 |
| DMC1 | 0.025725675 | 0.135300628 | 2.394887688 | 1.34E-09 | 4.49E-09 |
| HIST1H2BN | 0.144853843 | 0.43599248 | 1.58970529 | 2.49E-11 | 1.04E-10 |
| TCOF1 | 1.641381302 | 3.396091625 | 1.048964958 | 2.92E-22 | 7.40E-21 |
| BLM | 0.086445879 | 0.5219045 | 2.593916754 | 7.16E-23 | 2.09E-21 |
| HGFAC | 75.65320678 | 26.56018738 | -1.510135912 | 3.94E-17 | 3.80E-16 |
| ASRGL1 | 0.678415458 | 1.571669581 | 1.212056997 | 3.75E-05 | 7.29E-05 |
| SYP | 0.135751623 | 0.50039025 | 1.882084234 | 3.42E-12 | 1.61E-11 |
| TEX26 | 0.16637393 | 0.009201837 | -4.17636362 | 3.59E-28 | 1.31E-25 |
| CCL14 | 3.05948787 | 0.883862887 | -1.791395691 | 4.25E-24 | 1.74E-22 |
| ZNF365 | 0.005045148 | 0.015288975 | 1.599523193 | 0.00069837 | 0.001149883 |
| SCGB1D2 | 0.015169943 | 0.908690868 | 5.904501988 | 0.026222303 | 0.03403692 |
| RAD54B | 0.093644893 | 0.322440368 | 1.783760144 | 2.46E-22 | 6.32E-21 |
| FLVCR1 | 0.505398148 | 2.322758621 | 2.200346955 | 4.35E-26 | 3.76E-24 |
| PLAC8L1 | 0.174946027 | 0.526627236 | 1.589872233 | 3.19E-09 | 1.02E-08 |
| ANKS1B | 0.020257645 | 0.140019596 | 2.789090343 | 3.05E-06 | 6.75E-06 |
| TMC5 | 0.195931139 | 1.778510665 | 3.182251022 | 0.018555148 | 0.024629383 |
| MAD2L1 | 0.310793932 | 1.432782942 | 2.204789827 | 9.11E-24 | 3.40E-22 |
| C16orf59 | 0.249758304 | 1.735129264 | 2.796438597 | 9.79E-25 | 5.06E-23 |
| GUCY2D | 0.050327338 | 0.225280664 | 2.162309289 | 0.005201946 | 0.007492305 |
| ST3GAL6 | 9.358436756 | 3.738950475 | -1.323634199 | 1.82E-21 | 3.78E-20 |
| PDGFRA | 3.486518545 | 1.220675187 | -1.514107794 | 5.25E-20 | 8.23E-19 |
| TRIM11 | 1.148455189 | 2.496023584 | 1.119937 | 4.65E-21 | 8.96E-20 |
| FHL2 | 3.640047911 | 1.710652259 | -1.089410921 | 1.63E-08 | 4.75E-08 |
| KIF5A | 0.004459116 | 0.037836129 | 3.084934774 | 4.47E-09 | 1.40E-08 |
| PPP1R14D | 0.055204084 | 1.076356269 | 4.285236873 | 4.05E-12 | 1.89E-11 |
| KAT2A | 5.020622815 | 14.62010395 | 1.542015321 | 5.95E-25 | 3.27E-23 |
| PPT2-EGFL8 | 0.062986595 | 0.155731966 | 1.305948365 | 8.04E-18 | 8.74E-17 |
| ARHGAP4 | 2.523730296 | 5.271929729 | 1.062773398 | 2.63E-05 | 5.20E-05 |
| AKAP3 | 0.387111514 | 0.168808733 | -1.197359679 | 2.71E-13 | 1.49E-12 |
| ACAN | 0.013129245 | 0.16256202 | 3.630134388 | 1.07E-15 | 8.16E-15 |
| GCGR | 25.95494779 | 10.69695498 | -1.278809411 | 5.12E-14 | 3.10E-13 |
| SSUH2 | 0.061100968 | 0.927408304 | 3.923937513 | 1.64E-10 | 6.14E-10 |
| ACADSB | 85.83201097 | 33.29668531 | -1.366137236 | 1.43E-21 | 3.05E-20 |
| CSTL1 | 0.016100077 | 0.087226373 | 2.437696775 | 0.025325762 | 0.032953688 |
| SNAI2 | 2.507199052 | 5.457162217 | 1.12207439 | 0.044857381 | 0.056177343 |
| SFTA3 | 0.005149272 | 0.002331399 | -1.143172251 | 0.01996302 | 0.026387821 |
| RS1 | 0.007035846 | 0.035656853 | 2.341383648 | 9.04E-05 | 0.000167066 |
| PCDH17 | 0.303320288 | 0.811228021 | 1.419265487 | 2.02E-16 | 1.72E-15 |
| MDFI | 0.242277375 | 1.385171109 | 2.51533261 | 3.08E-10 | 1.12E-09 |
| DTYMK | 4.155101954 | 10.41169358 | 1.325248977 | 6.34E-22 | 1.47E-20 |
| RHOBTB2 | 0.836778917 | 1.742671626 | 1.058382338 | 7.12E-08 | 1.93E-07 |
| CD5L | 33.78201255 | 4.876266942 | -2.792406268 | 1.76E-24 | 8.28E-23 |
| HAS2 | 0.637076317 | 0.300262386 | -1.085242449 | 7.52E-14 | 4.44E-13 |
| CAPN8 | 0.055359029 | 0.437889299 | 2.983675643 | 3.23E-09 | 1.03E-08 |
| TGFB2 | 0.331203884 | 0.833618073 | 1.331666963 | 0.017161603 | 0.022914546 |
| FBXL18 | 0.261278765 | 0.682430294 | 1.385091811 | 1.80E-21 | 3.76E-20 |
| DNMT1 | 1.715297042 | 4.001786345 | 1.222185711 | 8.58E-19 | 1.08E-17 |
| GOLGA8A | 0.285593669 | 0.997497215 | 1.804348814 | 6.38E-10 | 2.23E-09 |
| BRCA2 | 0.096257983 | 0.267792526 | 1.476137598 | 1.27E-15 | 9.54E-15 |
| TMC7 | 0.085303601 | 0.419303395 | 2.297315957 | 8.04E-23 | 2.29E-21 |
| SPTBN2 | 7.657357133 | 3.385880402 | -1.177315531 | 3.68E-17 | 3.58E-16 |
| LPL | 0.187899972 | 1.103861323 | 2.554522181 | 5.49E-19 | 7.17E-18 |
| CAPS | 1.41536449 | 3.057201972 | 1.111038237 | 1.59E-09 | 5.26E-09 |
| C7 | 50.57499486 | 15.68140564 | -1.689369384 | 1.44E-19 | 2.06E-18 |
| METTL11B | 0.000789019 | 0.037222251 | 5.559962256 | 0.001941644 | 0.002979338 |
| C8A | 218.8435125 | 83.62686294 | -1.387861267 | 1.09E-22 | 3.01E-21 |
| PCNA | 19.30873948 | 47.46769711 | 1.297692074 | 1.01E-20 | 1.81E-19 |
| SLC6A9 | 0.232374499 | 1.036211313 | 2.156794579 | 9.81E-12 | 4.34E-11 |
| KCNJ5 | 0.201643005 | 0.942285808 | 2.224361358 | 1.44E-14 | 9.34E-14 |
| STIL | 0.108809295 | 0.612543598 | 2.493010729 | 1.76E-27 | 3.23E-25 |
| SCG2 | 0.008997902 | 0.13879049 | 3.947176284 | 2.79E-15 | 1.98E-14 |
| CCDC3 | 8.138621832 | 3.681256229 | -1.144586344 | 4.08E-18 | 4.66E-17 |
| CARMIL3 | 0.567133687 | 0.255703842 | -1.149215017 | 5.30E-11 | 2.13E-10 |
| SLC5A1 | 1.412650224 | 0.382055513 | -1.886550111 | 1.02E-24 | 5.24E-23 |
| CCDC155 | 0.008251755 | 0.06507887 | 2.979416351 | 0.035406478 | 0.044997077 |
| MAPK8IP2 | 0.177424124 | 1.734107719 | 3.288919428 | 1.15E-15 | 8.75E-15 |
| AUNIP | 0.182353497 | 0.367538383 | 1.011157055 | 9.38E-06 | 1.96E-05 |
| CACNB1 | 0.080407859 | 0.178063231 | 1.146981216 | 1.84E-13 | 1.03E-12 |
| CNR1 | 0.026189174 | 0.171150769 | 2.708225299 | 0.028280023 | 0.036534152 |
| TCAP | 0.306076595 | 1.237797947 | 2.0158112 | 3.16E-06 | 6.98E-06 |
| CCDC114 | 0.025650302 | 0.081330155 | 1.664814573 | 2.55E-08 | 7.27E-08 |
| ZCCHC12 | 0.027262209 | 0.141016267 | 2.370887201 | 1.00E-05 | 2.09E-05 |
| FAM81A | 0.044480696 | 0.199804689 | 2.167339161 | 2.75E-15 | 1.96E-14 |
| MYH13 | 0.001043053 | 0.019856668 | 4.250738989 | 0.007214697 | 0.010174607 |
| RNASE4 | 13.88855906 | 6.908769883 | -1.007396163 | 2.90E-17 | 2.89E-16 |
| TFAP2E | 0.056250758 | 0.195027846 | 1.793735687 | 1.11E-10 | 4.26E-10 |
| FPR1 | 3.090593071 | 0.859252659 | -1.846729396 | 8.73E-19 | 1.09E-17 |
| HIST1H2AG | 0.389289017 | 1.740379898 | 2.160488712 | 7.66E-05 | 0.000142817 |
| C22orf23 | 0.079157756 | 0.190657726 | 1.268182382 | 2.71E-13 | 1.49E-12 |
| KIF23 | 0.093844541 | 1.018396323 | 3.439882484 | 9.44E-27 | 1.07E-24 |
| GDF6 | 0.186522777 | 0.084244443 | -1.146698377 | 2.72E-13 | 1.49E-12 |
| NPBWR1 | 0.554423171 | 0.220663364 | -1.329140433 | 2.99E-11 | 1.24E-10 |
| RIMS3 | 0.111739649 | 0.268891217 | 1.266881442 | 1.65E-08 | 4.82E-08 |
| SBK3 | 0.024719784 | 0.178624168 | 2.853189245 | 7.07E-07 | 1.69E-06 |
| SLC9A9 | 2.083360748 | 0.936831531 | -1.153051135 | 1.73E-18 | 2.08E-17 |
| ARHGDIG | 0.007876357 | 0.064215738 | 3.027326535 | 0.031378892 | 0.040222108 |
| ANKRD30A | 0.001840931 | 0.016471006 | 3.161421166 | 0.031185874 | 0.03999456 |
| PRDM12 | 0.022504403 | 0.092960312 | 2.046407603 | 3.83E-10 | 1.38E-09 |
| ZNF559-ZNF177 | 0.016753703 | 0.034004816 | 1.021259052 | 0.00100601 | 0.001617726 |
| TTLL6 | 0.017910258 | 0.079464711 | 2.149528223 | 0.000874207 | 0.001417362 |
| STAC2 | 0.004891039 | 0.048265177 | 3.302769755 | 0.03056704 | 0.039273702 |
| NPAS4 | 0.015879128 | 0.005710126 | -1.4755372 | 0.000803656 | 0.001311549 |
| TMCO2 | 0.010139314 | 0.04041717 | 1.99500832 | 0.002655785 | 0.004003885 |
| IL12A | 0.046406678 | 0.117585951 | 1.341311362 | 0.000126807 | 0.000230449 |
| MFAP2 | 0.265128496 | 0.961985412 | 1.859323275 | 0.035460194 | 0.045050604 |
| REG1A | 0.06574318 | 55.37769839 | 9.718248126 | 1.43E-06 | 3.28E-06 |
| GALNT13 | 0.012402201 | 0.048168718 | 1.957500296 | 0.013195162 | 0.017915204 |
| PON3 | 72.59388923 | 30.72258737 | -1.24054839 | 1.96E-20 | 3.34E-19 |
| PLOD3 | 10.61279339 | 22.51773301 | 1.085257153 | 4.07E-22 | 9.85E-21 |
| NXPH3 | 0.060786645 | 0.192942603 | 1.666345433 | 2.46E-07 | 6.24E-07 |
| GRIN3B | 0.018727211 | 0.061958446 | 1.726164892 | 2.34E-05 | 4.66E-05 |
| TRIM31 | 0.586953588 | 3.047939771 | 2.376516059 | 4.71E-09 | 1.47E-08 |
| STMND1 | 0.037035066 | 0.371600336 | 3.32678798 | 2.53E-08 | 7.23E-08 |
| SLC38A6 | 0.763547694 | 1.73487319 | 1.184040031 | 2.08E-18 | 2.48E-17 |
| PLSCR4 | 13.62780002 | 3.943512641 | -1.78899951 | 5.40E-27 | 7.43E-25 |
| C12orf56 | 0.005254489 | 0.105530662 | 4.327968001 | 5.63E-05 | 0.000106829 |
| PRIM2 | 0.769773499 | 1.730847562 | 1.16897276 | 2.08E-23 | 6.90E-22 |
| NETO1 | 0.001807505 | 0.011136591 | 2.623236125 | 0.045378423 | 0.056802439 |
| RPS11 | 298.3789341 | 695.5287154 | 1.220964396 | 5.02E-18 | 5.65E-17 |
| C1orf105 | 0.152798957 | 0.359293943 | 1.233529917 | 0.000297416 | 0.000513734 |
| FAM162B | 0.277566921 | 0.794707791 | 1.517588845 | 7.85E-12 | 3.52E-11 |
| PTPN14 | 0.242722189 | 0.583895665 | 1.266404597 | 5.46E-10 | 1.92E-09 |
| ZNF648 | 0.084943681 | 0.429718627 | 2.338813778 | 0.007921191 | 0.011113754 |
| VASH2 | 0.046391541 | 0.271092413 | 2.546851065 | 2.06E-16 | 1.75E-15 |
| LTK | 0.961663687 | 0.361384171 | -1.411999126 | 1.35E-20 | 2.36E-19 |
| TK1 | 3.671830331 | 19.0615142 | 2.376091427 | 3.50E-22 | 8.65E-21 |
| RETREG1 | 14.05491557 | 3.167879655 | -2.149485353 | 2.09E-22 | 5.43E-21 |
| NAT8L | 0.030612668 | 0.365505939 | 3.577694174 | 0.005558438 | 0.0079799 |
| RPS16 | 121.7750449 | 264.0165855 | 1.116410047 | 2.13E-18 | 2.52E-17 |
| GAST | 0.004023165 | 2.893223767 | 9.490131247 | 0.024727703 | 0.032198996 |
| GNRH1 | 0.126686741 | 0.291070214 | 1.200101674 | 6.38E-13 | 3.32E-12 |
| MMP10 | 0.053567838 | 0.805329184 | 3.910139633 | 0.000131974 | 0.000239223 |
| CKB | 5.068427383 | 23.31532743 | 2.201668604 | 0.00722868 | 0.010191555 |
| CWH43 | 0.352248846 | 0.052143805 | -2.756027211 | 9.10E-19 | 1.14E-17 |
| TROAP | 0.118127485 | 2.344822046 | 4.311061859 | 7.38E-28 | 1.76E-25 |
| IL11 | 0.023501885 | 0.445386729 | 4.244210199 | 1.64E-07 | 4.24E-07 |
| AC093899.2 | 0.000865824 | 0.000160982 | -2.427173606 | 0.000977949 | 0.001575697 |
| RASA4 | 0.014928637 | 0.031641334 | 1.083727949 | 5.61E-07 | 1.36E-06 |
| KLK2 | 0.002207564 | 0.124869063 | 5.821816695 | 0.001713489 | 0.002652383 |
| SHOX2 | 0.010384286 | 0.084916355 | 3.031640469 | 4.07E-11 | 1.65E-10 |
| IGF2BP2 | 0.739908048 | 3.698892093 | 2.321675318 | 1.02E-05 | 2.13E-05 |
| RUSC1 | 1.53286508 | 4.02934532 | 1.394314732 | 7.60E-24 | 2.91E-22 |
| MS4A7 | 8.234136676 | 3.148155454 | -1.387110614 | 4.66E-17 | 4.44E-16 |
| NUP210L | 0.008551803 | 0.02674459 | 1.644946517 | 4.60E-05 | 8.82E-05 |
| CBLN1 | 0.332082591 | 0.993597164 | 1.581118964 | 1.49E-05 | 3.04E-05 |
| SPOCD1 | 0.014632599 | 0.100530483 | 2.780375035 | 1.73E-12 | 8.45E-12 |
| CCIN | 0.008439631 | 0.025667754 | 1.604705222 | 0.000317051 | 0.00054614 |
| CNTD1 | 0.104941868 | 0.238864068 | 1.18659947 | 1.43E-14 | 9.27E-14 |
| SIPA1L3 | 1.131731333 | 2.586864888 | 1.192673194 | 3.74E-17 | 3.63E-16 |
| CENPP | 0.131906574 | 0.306978923 | 1.218623131 | 2.58E-18 | 3.03E-17 |
| CLUL1 | 0.015705918 | 0.066089217 | 2.073106609 | 3.08E-06 | 6.83E-06 |
| SLCO2A1 | 1.048262194 | 2.455733056 | 1.228154132 | 0.000534758 | 0.000894887 |
| OR1J1 | 3.31E-05 | 0.015074727 | 8.830923963 | 0.046373006 | 0.057982097 |
| FAHD2A | 10.75898683 | 5.112777693 | -1.073363024 | 9.28E-24 | 3.44E-22 |
| PPP1R35 | 6.480451351 | 13.66901289 | 1.076742859 | 1.35E-18 | 1.64E-17 |
| KCNQ2 | 0.001290556 | 0.006034705 | 2.225290574 | 0.019175265 | 0.025411309 |
| ADAMTS14 | 0.083864723 | 0.235103758 | 1.487161609 | 1.27E-08 | 3.77E-08 |
| TMEM120B | 0.491093648 | 1.178433202 | 1.262799917 | 1.42E-24 | 6.95E-23 |
| KCP | 0.022189254 | 0.189134922 | 3.091482684 | 8.01E-17 | 7.28E-16 |
| ATP2A1 | 0.064129158 | 0.215065209 | 1.745721783 | 1.90E-12 | 9.24E-12 |
| LRRC4 | 0.573456568 | 0.160796544 | -1.834447826 | 1.11E-20 | 1.98E-19 |
| ZNF681 | 0.053614054 | 0.259578071 | 2.275485367 | 1.28E-09 | 4.29E-09 |
| AC105001.2 | 0.005223388 | 0.001385828 | -1.914237243 | 0.000479241 | 0.000806669 |
| C2CD4D | 0.064186457 | 0.176344274 | 1.458053894 | 1.27E-08 | 3.75E-08 |
| EDARADD | 0.08803744 | 0.448428952 | 2.348690335 | 0.03138038 | 0.040222108 |
| CCR3 | 0.010113444 | 0.142258345 | 3.814167047 | 0.000644091 | 0.001065027 |
| STEAP4 | 4.426452535 | 1.536913606 | -1.526114882 | 9.48E-23 | 2.66E-21 |
| TMEM132B | 0.007832154 | 0.044232671 | 2.497631266 | 0.002449026 | 0.003705365 |
| CYP1A2 | 173.1696992 | 24.98535164 | -2.793032085 | 1.13E-24 | 5.65E-23 |
| CFAP43 | 0.051963759 | 0.127154954 | 1.291009975 | 1.08E-05 | 2.24E-05 |
| SLC7A6 | 0.134544979 | 0.532407001 | 1.984440987 | 8.05E-22 | 1.83E-20 |
| BCAM | 16.44083494 | 38.43385421 | 1.225094092 | 5.39E-06 | 1.16E-05 |
| HOXA11 | 0.001575082 | 0.138207031 | 6.45526021 | 1.10E-08 | 3.30E-08 |
| TAP1 | 8.083075105 | 16.42539156 | 1.022951605 | 1.11E-08 | 3.32E-08 |
| KRTAP5-9 | 0.052247598 | 0.107565215 | 1.04177499 | 0.000727087 | 0.001194512 |
| ZC2HC1C | 1.876540128 | 0.753504489 | -1.316387129 | 2.18E-18 | 2.58E-17 |
| PTGES3L-AARSD1 | 0.004346061 | 0.009880747 | 1.184911636 | 0.013286089 | 0.018029517 |
| HS3ST3B1 | 14.49585093 | 5.398237914 | -1.425079558 | 8.40E-22 | 1.90E-20 |
| CLEC4C | 0.039892839 | 0.010894338 | -1.872551247 | 1.94E-08 | 5.62E-08 |
| GRM2 | 0.020184147 | 0.068359762 | 1.75992474 | 3.97E-08 | 1.11E-07 |
| TRIM7 | 0.029859227 | 0.187165527 | 2.648066019 | 1.90E-16 | 1.63E-15 |
| STAMBPL1 | 0.320291837 | 0.7389632 | 1.20611549 | 4.25E-09 | 1.33E-08 |
| PTP4A3 | 1.685784519 | 9.70136827 | 2.524768098 | 5.71E-19 | 7.42E-18 |
| OR8G5 | 0.001579792 | 0.069744361 | 5.464269748 | 0.015254476 | 0.020531978 |
| NAALADL2 | 0.525636899 | 0.220056486 | -1.256192658 | 5.80E-18 | 6.45E-17 |
| AC069503.2 | 0.024314906 | 0.069621857 | 1.51769931 | 7.36E-10 | 2.55E-09 |
| DNA2 | 0.295338626 | 0.783280053 | 1.407158166 | 5.36E-17 | 5.04E-16 |
| GOLGA6L9 | 0.086399111 | 0.217600769 | 1.332595278 | 1.43E-15 | 1.07E-14 |
| ST8SIA6 | 0.264591665 | 0.045195945 | -2.549502371 | 1.72E-23 | 5.87E-22 |
| MTRNR2L8 | 1.159971951 | 0.505957131 | -1.197002861 | 2.53E-08 | 7.23E-08 |
| CNTNAP1 | 0.22328555 | 0.686118663 | 1.619568219 | 4.70E-12 | 2.17E-11 |
| RPL27 | 112.6802716 | 230.5306016 | 1.032723326 | 2.34E-19 | 3.24E-18 |
| DCDC2B | 0.01220856 | 0.027426949 | 1.167701037 | 0.000363416 | 0.00062221 |
| CLEC2L | 0.003767794 | 0.446950411 | 6.890250709 | 9.15E-07 | 2.16E-06 |
| CBFA2T3 | 1.889624142 | 0.50333477 | -1.908509135 | 4.18E-23 | 1.30E-21 |
| APOE | 1560.368596 | 3430.514017 | 1.136537894 | 1.16E-13 | 6.69E-13 |
| HHIPL2 | 0.01770424 | 1.474086933 | 6.379582882 | 6.14E-17 | 5.71E-16 |
| FKBP6 | 0.007151896 | 0.020926487 | 1.548932426 | 0.049149995 | 0.06125322 |
| FAM198A | 2.898799941 | 0.837148305 | -1.791900639 | 1.82E-18 | 2.18E-17 |
| ZNF221 | 0.095919514 | 0.196231668 | 1.032661636 | 6.86E-06 | 1.46E-05 |
| CCDC137 | 3.83454044 | 7.808713266 | 1.026031153 | 3.20E-20 | 5.23E-19 |
| GPR82 | 0.176722124 | 0.074898306 | -1.238477667 | 1.69E-12 | 8.26E-12 |
| CFAP100 | 0.012599159 | 0.026339598 | 1.063905881 | 0.000459591 | 0.00077511 |
| NCAPG2 | 0.743302161 | 1.749602305 | 1.235006318 | 5.01E-17 | 4.75E-16 |
| OR8H2 | 0.000908753 | 6.95E-05 | -3.708145498 | 0.001026736 | 0.00164901 |
| TRIM67 | 0.019839978 | 0.055495148 | 1.483951253 | 0.00419559 | 0.006125081 |
| LUZP2 | 0.006545543 | 0.126046488 | 4.267299267 | 0.004302562 | 0.006271291 |
| KIFC2 | 0.728488917 | 2.831629846 | 1.958653759 | 4.79E-21 | 9.20E-20 |
| CPXM1 | 0.242465614 | 1.377480095 | 2.506179402 | 6.15E-15 | 4.19E-14 |
| IGFBP3 | 110.8292727 | 39.71583415 | -1.480552773 | 3.70E-22 | 9.10E-21 |
| MTHFD1L | 0.820253418 | 2.370390422 | 1.530983096 | 2.90E-18 | 3.39E-17 |
| EBF1 | 0.137661929 | 0.641266734 | 2.219794935 | 2.10E-22 | 5.44E-21 |
| C1orf162 | 5.601669357 | 2.537044591 | -1.142707953 | 1.70E-17 | 1.77E-16 |
| EVX1 | 0.00049982 | 0.145314996 | 8.183558016 | 5.45E-14 | 3.28E-13 |
| CCT6A | 17.87412356 | 35.91979935 | 1.006906789 | 1.52E-23 | 5.32E-22 |
| RDM1 | 0.035998402 | 0.469737959 | 3.705851413 | 1.32E-22 | 3.62E-21 |
| TNFRSF18 | 0.323131375 | 1.299101474 | 2.007321382 | 1.26E-10 | 4.81E-10 |
| STEAP3 | 58.98921445 | 22.08617246 | -1.417307776 | 1.94E-22 | 5.08E-21 |
| IL4I1 | 0.566760874 | 1.721627802 | 1.602961209 | 8.73E-05 | 0.000161482 |
| UBE2F-SCLY | 0.005237075 | 0.012808231 | 1.290238095 | 4.13E-05 | 8.00E-05 |
| ZNF696 | 0.830755849 | 1.903454873 | 1.196123917 | 3.18E-19 | 4.33E-18 |
| GLP1R | 0.001636655 | 0.178628148 | 6.770065426 | 1.73E-06 | 3.95E-06 |
| ERLIN1 | 23.38917597 | 10.799209 | -1.114915391 | 2.50E-23 | 8.15E-22 |
| RSAD2 | 3.419597858 | 1.174106613 | -1.54226326 | 1.11E-05 | 2.30E-05 |
| MMP14 | 7.501812184 | 24.84192299 | 1.727465807 | 3.44E-09 | 1.09E-08 |
| PDC | 0.007390617 | 0.014811532 | 1.002954189 | 0.027579784 | 0.035694795 |
| CEP131 | 1.05751666 | 3.636874707 | 1.782018833 | 5.48E-28 | 1.52E-25 |
| MYBPC1 | 0.007699842 | 0.102035606 | 3.72810005 | 2.13E-13 | 1.19E-12 |
| NFAM1 | 1.77441941 | 0.753926827 | -1.234850638 | 2.63E-16 | 2.19E-15 |
| IL1B | 1.275684231 | 0.362842708 | -1.813855081 | 6.03E-19 | 7.80E-18 |
| ACTN2 | 0.073734063 | 1.960382206 | 4.732659891 | 4.29E-06 | 9.33E-06 |
| FBLIM1 | 2.107391196 | 5.63681075 | 1.419420984 | 2.32E-10 | 8.54E-10 |
| GAP43 | 0.015913859 | 0.121385198 | 2.931236879 | 4.63E-09 | 1.44E-08 |
| TLL2 | 0.013883944 | 0.055837112 | 2.007806837 | 6.98E-06 | 1.48E-05 |
| SNRPB | 41.2975544 | 115.0747302 | 1.478442806 | 2.86E-25 | 1.79E-23 |
| S100A6 | 34.74718532 | 117.4626307 | 1.757233831 | 0.042486365 | 0.053392964 |
| MMAA | 4.703244888 | 2.160610182 | -1.122217648 | 3.14E-24 | 1.35E-22 |
| FCN2 | 52.02137022 | 2.134939213 | -4.606837501 | 1.55E-29 | 2.69E-26 |
| TRIM46 | 0.03603925 | 0.126194623 | 1.808009533 | 1.00E-13 | 5.82E-13 |
| IKBKE | 0.777808661 | 1.812344996 | 1.220370407 | 3.31E-08 | 9.35E-08 |
| LRRC3B | 0.01350508 | 0.045401566 | 1.74923991 | 0.000266553 | 0.000463829 |
| CEND1 | 0.028860474 | 0.157950896 | 2.452309207 | 7.36E-15 | 4.97E-14 |
| C3orf22 | 0.007041712 | 0.025413998 | 1.851625279 | 8.44E-07 | 2.00E-06 |
| MGAT4D | 0.002036428 | 0.00057151 | -1.833190261 | 0.007113742 | 0.010039514 |
| CHRM3 | 0.130691404 | 0.322366931 | 1.302539505 | 0.000198143 | 0.000350546 |
| RNF152 | 9.093778798 | 2.824376541 | -1.686947472 | 6.64E-21 | 1.25E-19 |
| FAM83H | 6.36016904 | 17.71091406 | 1.477501656 | 2.92E-18 | 3.41E-17 |
| MAGEA3 | 0.034363354 | 5.492042928 | 7.320328223 | 0.003136857 | 0.004665277 |
| C1orf194 | 0.007067061 | 0.034871779 | 2.302877762 | 9.21E-07 | 2.17E-06 |
| KCNA3 | 0.237465244 | 0.087777421 | -1.435794591 | 2.04E-14 | 1.30E-13 |
| TBKBP1 | 0.830744131 | 1.744394699 | 1.07025041 | 1.32E-11 | 5.72E-11 |
| LAGE3 | 10.86890707 | 24.10201707 | 1.148947012 | 7.54E-15 | 5.08E-14 |
| ENTPD3 | 0.016817449 | 0.116669023 | 2.794390764 | 0.02801575 | 0.036222807 |
| PLAG1 | 0.085385544 | 0.335571242 | 1.974555346 | 8.83E-06 | 1.85E-05 |
| TMEM78 | 0.001261091 | 0.011264978 | 3.159100517 | 0.006232078 | 0.008877644 |
| PYCR1 | 1.290300442 | 7.466942646 | 2.532810618 | 0.001886169 | 0.002899133 |
| OR4D11 | 0.001069319 | 0.000497872 | -1.102846244 | 0.000967621 | 0.001559864 |
| PRPF3 | 3.021491149 | 6.212854396 | 1.039995528 | 1.17E-21 | 2.56E-20 |
| WDR17 | 0.039774157 | 0.011748833 | -1.759313924 | 2.27E-21 | 4.62E-20 |
| CDY2B | 0.000149028 | 2.63E-05 | -2.504534747 | 0.000641173 | 0.00106056 |
| COX6A2 | 6.376274134 | 2.984115113 | -1.095410467 | 1.97E-10 | 7.33E-10 |
| HOXA3 | 0.089593126 | 0.681986981 | 2.928284254 | 7.56E-07 | 1.80E-06 |
| NFIL3 | 43.58773222 | 20.04715272 | -1.120524798 | 8.30E-16 | 6.40E-15 |
| MT2A | 1087.274636 | 334.1626145 | -1.702094157 | 1.09E-21 | 2.40E-20 |
| DPF3 | 0.823326069 | 0.251458976 | -1.711140856 | 8.41E-20 | 1.25E-18 |
| UBD | 11.52586449 | 76.17608345 | 2.724463151 | 5.83E-16 | 4.60E-15 |
| RBP7 | 3.292240989 | 12.6630613 | 1.94348437 | 6.21E-15 | 4.23E-14 |
| DNAH12 | 0.01209457 | 0.081438912 | 2.751358835 | 9.00E-05 | 0.000166259 |
| ACVR1C | 0.50261061 | 0.171418337 | -1.551919678 | 9.59E-18 | 1.03E-16 |
| MS4A6A | 11.19132518 | 3.459344574 | -1.69381025 | 9.63E-22 | 2.16E-20 |
| SMYD3 | 0.440993198 | 1.236311547 | 1.487214036 | 1.87E-15 | 1.37E-14 |
| PTPN5 | 0.007107508 | 0.025768856 | 1.858212796 | 3.40E-06 | 7.50E-06 |
| ZFP62 | 1.407227854 | 2.819659767 | 1.002665147 | 1.27E-21 | 2.74E-20 |
| TDGF1 | 0.331662539 | 1.823105548 | 2.458610114 | 0.014523355 | 0.019610721 |
| PIAS3 | 1.177271493 | 2.388849311 | 1.020868789 | 5.62E-20 | 8.74E-19 |
| LOXL4 | 3.531575491 | 8.252419143 | 1.224505162 | 0.00868866 | 0.012121428 |
| SKA1 | 0.073410064 | 1.806202172 | 4.620837724 | 3.35E-28 | 1.30E-25 |
| BEND4 | 0.034857051 | 0.013660061 | -1.351486627 | 1.72E-21 | 3.61E-20 |
| RAP2A | 2.875793052 | 6.17052766 | 1.101434004 | 2.72E-15 | 1.94E-14 |
| BIRC7 | 0.0818587 | 0.487793714 | 2.575063501 | 0.007863854 | 0.01104005 |
| STXBP6 | 0.486655625 | 1.534100394 | 1.656419763 | 4.68E-14 | 2.85E-13 |
| GAD1 | 0.004176104 | 0.113873156 | 4.769126109 | 1.50E-12 | 7.43E-12 |
| POU3F2 | 0.004024328 | 0.048758879 | 3.598845212 | 1.24E-07 | 3.26E-07 |
| PRSS50 | 0.111070032 | 0.281509013 | 1.341711498 | 0.011504338 | 0.015757064 |
| SHBG | 42.7492148 | 14.19002427 | -1.591020865 | 7.46E-18 | 8.15E-17 |
| TLCD1 | 3.134344165 | 9.357699047 | 1.577990228 | 8.80E-18 | 9.51E-17 |
| CSMD2 | 0.011141219 | 0.093306884 | 3.066076443 | 4.16E-15 | 2.90E-14 |
| SGMS2 | 5.319310397 | 1.702822348 | -1.643311295 | 3.27E-16 | 2.69E-15 |
| KCNK18 | 0.00084667 | 0.000261502 | -1.694979621 | 0.000367747 | 0.000628933 |
| KIAA1614 | 0.051895798 | 0.203548778 | 1.97168493 | 2.40E-19 | 3.31E-18 |
| LILRA1 | 0.503791259 | 0.156337304 | -1.688164022 | 6.12E-21 | 1.16E-19 |
| EPPIN-WFDC6 | 0.000454623 | 0.000177773 | -1.354631315 | 0.001482511 | 0.002319713 |
| DPT | 12.20685828 | 3.36100831 | -1.860725923 | 1.19E-20 | 2.11E-19 |
| RET | 1.208096771 | 0.252372439 | -2.25910975 | 1.54E-18 | 1.86E-17 |
| STUM | 0.045873083 | 0.151545233 | 1.724028709 | 1.84E-05 | 3.71E-05 |
| SIGLEC9 | 1.649082565 | 0.645751806 | -1.352611954 | 2.38E-21 | 4.82E-20 |
| ZNF687 | 2.910431856 | 5.957242313 | 1.033411404 | 3.91E-18 | 4.48E-17 |
| GBX2 | 0.001026003 | 0.020605805 | 4.327943552 | 5.52E-08 | 1.51E-07 |
| RNF125 | 5.257377303 | 1.492172242 | -1.8169292 | 6.58E-24 | 2.56E-22 |
| ADAMTS18 | 0.005325201 | 0.047519085 | 3.15759912 | 2.78E-08 | 7.92E-08 |
| ETS2 | 87.01220129 | 35.90792777 | -1.276915319 | 3.07E-20 | 5.06E-19 |
| CGB7 | 0.002848362 | 0.008754428 | 1.619880271 | 0.020309502 | 0.026779117 |
| CORO6 | 0.06936962 | 0.16368664 | 1.238560679 | 4.48E-07 | 1.10E-06 |
| ACOT12 | 28.29129778 | 13.84630938 | -1.030856869 | 2.16E-14 | 1.37E-13 |
| LAMA5 | 2.260326169 | 4.817884432 | 1.091868816 | 1.68E-07 | 4.35E-07 |
| FAM182B | 0.016648139 | 0.070556963 | 2.083427544 | 2.06E-11 | 8.73E-11 |
| LILRB5 | 2.997350268 | 0.838543244 | -1.837730596 | 1.82E-23 | 6.17E-22 |
| AR | 12.98040286 | 6.204018503 | -1.065060266 | 1.14E-15 | 8.68E-15 |
| SLC1A1 | 21.26584444 | 9.111815871 | -1.222727645 | 3.10E-15 | 2.19E-14 |
| SOX9 | 3.610824671 | 9.60436131 | 1.411361308 | 0.00158535 | 0.002467227 |
| BCO2 | 7.408876027 | 0.909590877 | -3.025965003 | 3.34E-27 | 5.30E-25 |
| AQP7 | 4.385793272 | 1.94048691 | -1.17641911 | 1.61E-19 | 2.28E-18 |
| LIFR | 6.261597678 | 0.912234085 | -2.779054834 | 5.86E-29 | 5.07E-26 |
| GSTM1 | 42.71214321 | 20.12961261 | -1.085326883 | 0.008942682 | 0.012453477 |
| SPAG4 | 1.064280045 | 2.789061233 | 1.38990179 | 1.47E-06 | 3.38E-06 |
| TSPAN8 | 9.73977808 | 36.91969285 | 1.922429745 | 2.02E-05 | 4.05E-05 |
| EMX1 | 0.02323213 | 0.338519149 | 3.865044111 | 1.34E-07 | 3.52E-07 |
| CYP2C9 | 295.3034864 | 117.9010221 | -1.324622164 | 5.26E-18 | 5.89E-17 |
| NECAB3 | 3.555994538 | 9.802091736 | 1.462836541 | 5.60E-23 | 1.70E-21 |
| AC010463.1 | 0.000612798 | 0.000170885 | -1.84238716 | 0.000818429 | 0.001334149 |
| HOXC4 | 0.035413571 | 0.162757387 | 2.200348778 | 0.000279586 | 0.000484928 |
| ASCL5 | 0.006899364 | 0.03902957 | 2.500032232 | 5.82E-05 | 0.000110327 |
| PALM2 | 0.696238138 | 0.219119063 | -1.667865843 | 2.75E-18 | 3.22E-17 |
| RPS27 | 240.2380519 | 625.9119654 | 1.381495075 | 1.24E-19 | 1.80E-18 |
| TMEM54 | 4.412302991 | 9.477039396 | 1.102904571 | 0.037607945 | 0.047631424 |
| TCF3 | 2.633031825 | 5.61474124 | 1.092494577 | 1.87E-22 | 4.94E-21 |
| PEX5L | 0.00511589 | 0.012433021 | 1.281119702 | 8.88E-06 | 1.86E-05 |
| CXorf67 | 0.002314373 | 0.182915539 | 6.304412365 | 0.044790211 | 0.056108785 |
| RASGRF1 | 0.01778317 | 0.331614754 | 4.220923778 | 2.87E-07 | 7.19E-07 |
| SERPINA11 | 268.7107015 | 91.31562385 | -1.557120151 | 7.81E-22 | 1.79E-20 |
| AC093155.3 | 0.081721015 | 0.036207217 | -1.174429851 | 2.82E-11 | 1.17E-10 |
| FAM180A | 3.511135369 | 0.644514231 | -2.4456535 | 1.02E-25 | 7.51E-24 |
| CTSA | 25.05233454 | 53.90648265 | 1.105513729 | 1.35E-19 | 1.94E-18 |
| ZNF93 | 0.131316962 | 0.31887464 | 1.279936082 | 9.49E-05 | 0.000174778 |
| PHEX | 0.014858191 | 0.139643436 | 3.232417373 | 3.56E-10 | 1.28E-09 |
| CATSPER1 | 0.03659114 | 0.081549414 | 1.156180146 | 6.25E-06 | 1.34E-05 |
| RASL12 | 0.261983464 | 1.295672068 | 2.306152962 | 2.18E-25 | 1.44E-23 |
| CHST6 | 0.004696753 | 0.032635921 | 2.796725178 | 6.38E-10 | 2.23E-09 |
| SLCO6A1 | 0.000721963 | 0.094258804 | 7.028558135 | 0.000599143 | 0.000994957 |
| EXTL1 | 0.012095083 | 0.071005417 | 2.553508404 | 8.91E-11 | 3.45E-10 |
| HTRA4 | 0.04367698 | 0.106970957 | 1.29227413 | 0.000459596 | 0.00077511 |
| BPIFA2 | 0.001592991 | 0.334840466 | 7.715591691 | 1.07E-05 | 2.23E-05 |
| CBX2 | 0.137059797 | 0.949420131 | 2.792241186 | 3.52E-24 | 1.48E-22 |
| ALKAL1 | 0.287072791 | 0.132769513 | -1.112492693 | 8.32E-21 | 1.53E-19 |
| BUB1B | 0.112319642 | 1.39227245 | 3.631759405 | 2.73E-26 | 2.59E-24 |
| LGALS1 | 53.04774244 | 122.4012974 | 1.20625559 | 6.91E-05 | 0.000129587 |
| C11orf88 | 0.002967164 | 0.010222553 | 1.784598678 | 0.000221505 | 0.000389047 |
| FOXN4 | 0.190838198 | 1.051467775 | 2.461982762 | 0.017190317 | 0.022946971 |
| TMEM215 | 0.002886852 | 0.00107768 | -1.421568485 | 0.000963239 | 0.001553605 |
| AC138969.1 | 0.013083795 | 0.027729505 | 1.083640805 | 6.90E-07 | 1.65E-06 |
| SCARA5 | 1.008250259 | 0.29731297 | -1.76179947 | 3.86E-21 | 7.52E-20 |
| NECTIN1 | 0.963554627 | 2.278250375 | 1.24148794 | 6.92E-13 | 3.59E-12 |
| FER1L5 | 0.005083582 | 0.03031221 | 2.57598165 | 2.70E-10 | 9.85E-10 |
| AL139392.1 | 0.056496133 | 0.525456948 | 3.217348548 | 2.47E-09 | 7.98E-09 |
| OSGIN1 | 29.41473049 | 62.41926122 | 1.085452464 | 0.000224759 | 0.00039441 |
| IDUA | 1.796777147 | 4.208326565 | 1.227835179 | 8.13E-17 | 7.38E-16 |
| SBSPON | 0.148108094 | 0.757330154 | 2.354271889 | 5.19E-05 | 9.89E-05 |
| POU5F1 | 0.168242905 | 0.704366763 | 2.065781168 | 4.98E-15 | 3.43E-14 |
| ARID3A | 0.445681112 | 2.616841177 | 2.553742641 | 1.92E-12 | 9.32E-12 |
| PI3 | 1.571671264 | 6.857707659 | 2.125426914 | 0.006839638 | 0.009674614 |
| PLG | 390.0929363 | 178.2907766 | -1.129585804 | 3.04E-19 | 4.16E-18 |
| CHAF1B | 0.200975802 | 1.263237999 | 2.652032759 | 3.85E-23 | 1.21E-21 |
| CBR4 | 11.5437763 | 5.41601885 | -1.091810583 | 3.26E-22 | 8.13E-21 |
| CCER2 | 0.141583891 | 0.521566009 | 1.881192717 | 1.18E-10 | 4.48E-10 |
| SLC36A1 | 0.600067111 | 1.247840556 | 1.056237839 | 2.31E-18 | 2.73E-17 |
| FXN | 8.539158937 | 3.89577314 | -1.13218431 | 1.87E-24 | 8.74E-23 |
| ANKRD7 | 0.003696039 | 0.03286934 | 3.152690697 | 0.02556858 | 0.033249412 |
| FIGNL2 | 0.117580225 | 0.348137297 | 1.566010933 | 1.38E-07 | 3.61E-07 |
| KIF2C | 0.172431095 | 2.807332856 | 4.025108258 | 6.40E-28 | 1.69E-25 |
| ADAMTS1 | 9.557580769 | 3.542226394 | -1.431989065 | 1.08E-16 | 9.64E-16 |
| CABP7 | 0.045432562 | 0.127847313 | 1.492623278 | 1.66E-09 | 5.47E-09 |
| DNM3 | 0.046992094 | 0.154173468 | 1.714064547 | 1.03E-21 | 2.29E-20 |
| ZNF341 | 0.493338427 | 1.126790727 | 1.191570025 | 4.79E-21 | 9.20E-20 |
| NPIPA3 | 0.030978787 | 0.080555475 | 1.378702006 | 0.001346417 | 0.002120413 |
| PIGU | 4.45775998 | 9.802012844 | 1.136759098 | 9.89E-23 | 2.76E-21 |
| CDCA2 | 0.062008524 | 0.626294539 | 3.336302835 | 5.10E-25 | 2.88E-23 |
| CYP4V2 | 20.76319381 | 8.537514724 | -1.28214031 | 3.30E-22 | 8.20E-21 |
| PKD1L1 | 0.025121241 | 0.059855744 | 1.252581955 | 2.65E-06 | 5.91E-06 |
| RHOF | 0.071531471 | 0.201806042 | 1.496319354 | 1.92E-09 | 6.29E-09 |
| PDE2A | 3.166855159 | 1.169006923 | -1.437767412 | 1.31E-21 | 2.83E-20 |
| OVOL3 | 0.019207639 | 0.054744767 | 1.511040846 | 0.00028564 | 0.000494824 |
| NT5DC2 | 0.892167497 | 5.691345908 | 2.673383371 | 1.40E-20 | 2.45E-19 |
| ZEB2 | 1.185645984 | 0.534702563 | -1.14886481 | 1.01E-22 | 2.81E-21 |
| KIF20A | 0.189863647 | 2.622892635 | 3.788123146 | 9.31E-27 | 1.07E-24 |
| FLJ22763 | 2.116340899 | 0.655972519 | -1.689864753 | 5.86E-18 | 6.51E-17 |
| FGD1 | 0.671576706 | 1.751469889 | 1.38294209 | 1.19E-16 | 1.05E-15 |
| KCNK17 | 0.950921536 | 0.367423509 | -1.371882367 | 2.38E-22 | 6.14E-21 |
| DKKL1 | 0.051672583 | 0.20955485 | 2.019857015 | 4.38E-06 | 9.51E-06 |
| FDCSP | 0.2067426 | 11.83958257 | 5.839638713 | 1.52E-06 | 3.48E-06 |
| CACNG8 | 0.009718896 | 0.026839349 | 1.465485292 | 1.74E-07 | 4.51E-07 |
| ADGRB2 | 0.021800084 | 0.184834694 | 3.083830009 | 1.98E-09 | 6.48E-09 |
| EPHA10 | 0.012559233 | 0.148223812 | 3.560956976 | 0.001949864 | 0.00299083 |
| CRYBG2 | 0.088691366 | 0.607955184 | 2.777099403 | 3.23E-20 | 5.27E-19 |
| NPFFR2 | 0.005977403 | 0.579539649 | 6.59924486 | 2.59E-06 | 5.79E-06 |
| ESYT3 | 0.026033713 | 0.131257744 | 2.333949567 | 2.23E-07 | 5.69E-07 |
| POTEF | 0.004691222 | 0.013567301 | 1.532097979 | 0.002872753 | 0.004302167 |
| C1orf198 | 4.488856495 | 10.47052568 | 1.221913995 | 1.51E-14 | 9.77E-14 |
| HOXA7 | 0.005913953 | 0.027372093 | 2.210511184 | 0.007429211 | 0.010456275 |
| HIST1H2BJ | 0.474727309 | 1.709435689 | 1.848349199 | 9.25E-09 | 2.79E-08 |
| WDHD1 | 0.158268849 | 0.671984254 | 2.086050098 | 2.72E-25 | 1.73E-23 |
| BMP8A | 0.057528472 | 0.12580199 | 1.128806693 | 6.38E-06 | 1.36E-05 |
| RTKN2 | 0.021571146 | 0.180743891 | 3.06677214 | 2.23E-19 | 3.09E-18 |
| TMEM74B | 0.775115873 | 2.034885633 | 1.392463812 | 5.95E-06 | 1.27E-05 |
| ABCC1 | 1.0255365 | 2.111602582 | 1.041959495 | 0.011069071 | 0.015201042 |
| SLC35D1 | 24.54534378 | 10.94157136 | -1.16562943 | 7.99E-23 | 2.29E-21 |
| C1orf106 | 0.232481931 | 1.315567692 | 2.500494987 | 0.002919255 | 0.004366254 |
| NEK2 | 0.162509981 | 2.654973086 | 4.030097007 | 1.73E-26 | 1.79E-24 |
| SIGLEC5 | 0.138087315 | 0.064525927 | -1.09762993 | 1.05E-07 | 2.78E-07 |
| SP8 | 0.000932736 | 0.127274259 | 7.092256135 | 0.000246289 | 0.000430745 |
| SYT15 | 0.02608145 | 0.068646967 | 1.396171908 | 0.029222013 | 0.037673008 |
| TUBA1B | 12.06374813 | 27.25726267 | 1.175962473 | 5.75E-13 | 3.01E-12 |
| GJD4 | 0.004463561 | 0.012714189 | 1.510172254 | 0.015463597 | 0.020797255 |
| ZNF300 | 0.133883155 | 0.592979614 | 2.147008056 | 6.33E-12 | 2.87E-11 |
| MIP | 0.337185907 | 0.115098218 | -1.550678744 | 5.74E-19 | 7.45E-18 |
| AVPR2 | 0.037141064 | 0.136663036 | 1.879536027 | 3.53E-07 | 8.77E-07 |
| ADAM32 | 0.006785107 | 0.023855561 | 1.813882131 | 0.002960434 | 0.004422784 |
| DNAH3 | 0.004525203 | 0.01942288 | 2.101702756 | 0.000383494 | 0.000654426 |
| NR6A1 | 0.405908906 | 0.983098528 | 1.276180019 | 5.62E-13 | 2.96E-12 |
| DQX1 | 0.01041736 | 0.772214008 | 6.211939052 | 9.09E-08 | 2.42E-07 |
| ASB16 | 0.091099566 | 0.412388379 | 2.178487588 | 5.21E-22 | 1.23E-20 |
| C16orf82 | 0.000646748 | 0.000277026 | -1.223181946 | 0.001680728 | 0.002604718 |
| GLI1 | 0.103955841 | 0.281046212 | 1.434836547 | 2.88E-08 | 8.18E-08 |
| CFAP45 | 0.072222736 | 0.172728661 | 1.257982506 | 0.000373767 | 0.000639019 |
| PAMR1 | 3.309594532 | 0.948474925 | -1.802972941 | 8.05E-27 | 9.93E-25 |
| TMPRSS15 | 0.001604609 | 0.761342768 | 8.890180607 | 0.009153279 | 0.012729446 |
| FER1L6 | 0.005203112 | 0.397897807 | 6.256879417 | 3.01E-07 | 7.53E-07 |
| OR1L8 | 0.026879084 | 0.011766094 | -1.191848567 | 1.65E-05 | 3.35E-05 |
| CENPH | 0.538823726 | 1.967090056 | 1.868177723 | 1.44E-23 | 5.09E-22 |
| RIPK4 | 12.26295524 | 5.210896826 | -1.234703099 | 6.52E-19 | 8.38E-18 |
| CLDN15 | 4.487720723 | 15.82387058 | 1.818047729 | 1.62E-08 | 4.74E-08 |
| MEF2B | 0.076939067 | 0.176538548 | 1.198194996 | 9.99E-11 | 3.85E-10 |
| EN2 | 0.005394699 | 0.087973341 | 4.027452069 | 0.006751548 | 0.009558703 |
| HMHB1 | 0.003066755 | 0.057454708 | 4.227640379 | 0.021076378 | 0.027729919 |
| HOXD10 | 0.005900615 | 0.172511093 | 4.869679992 | 2.25E-09 | 7.30E-09 |
| TAS2R10 | 0.011880874 | 0.038477228 | 1.695363847 | 4.32E-05 | 8.34E-05 |
| RIC3 | 0.234902758 | 0.092918031 | -1.338033165 | 8.48E-18 | 9.21E-17 |
| YEATS2 | 0.737468685 | 1.544527712 | 1.066512061 | 1.38E-24 | 6.77E-23 |
| PHLDA1 | 36.0429773 | 7.79713213 | -2.208702701 | 8.68E-21 | 1.58E-19 |
| ZNF114 | 0.026903767 | 0.061107671 | 1.183545303 | 0.000858906 | 0.001394433 |
| KCNU1 | 0.005030763 | 0.896546687 | 7.477457645 | 0.010514716 | 0.014494767 |
| OR6N1 | 0.000951826 | 0.000239473 | -1.990833064 | 0.00034504 | 0.000591855 |
| ELOA3D | 0.00021884 | 6.41E-05 | -1.770556964 | 0.00063255 | 0.001047302 |
| SYNPO2 | 1.381381906 | 0.66634383 | -1.051773534 | 1.67E-18 | 2.00E-17 |
| COLEC10 | 17.04877478 | 1.217954369 | -3.807136074 | 2.31E-29 | 3.13E-26 |
| CSAG1 | 0.172197427 | 4.204522909 | 4.609806612 | 8.16E-08 | 2.19E-07 |
| KRT86 | 0.106046529 | 0.5548855 | 2.387492703 | 0.000387097 | 0.00066014 |
| S100A7L2 | 0.0022372 | 0.000559467 | -1.999568494 | 0.001083457 | 0.001734199 |
| MAGEA6 | 0.013094615 | 4.372899319 | 8.383472665 | 0.000899589 | 0.001456107 |
| GDPD2 | 0.008092552 | 0.019340686 | 1.256972319 | 2.35E-07 | 5.98E-07 |
| WDR88 | 0.147905728 | 0.321605373 | 1.120613584 | 3.26E-11 | 1.34E-10 |
| SERPINB9 | 7.193100437 | 2.626620191 | -1.453406146 | 4.28E-15 | 2.98E-14 |
| UCK2 | 1.483103197 | 4.645859236 | 1.647326456 | 4.46E-24 | 1.80E-22 |
| CAPG | 4.777319859 | 12.51533929 | 1.389424024 | 2.13E-07 | 5.46E-07 |
| C5orf30 | 0.30459061 | 1.00153108 | 1.717263818 | 0.000230252 | 0.000403817 |
| SPRR2B | 0.042334169 | 0.007324396 | -2.531040915 | 0.000461816 | 0.000778686 |
| ASIC1 | 0.052107339 | 0.563789929 | 3.435599228 | 5.13E-11 | 2.06E-10 |
| TGM3 | 0.188158614 | 7.395240432 | 5.296575809 | 9.64E-19 | 1.20E-17 |
| ADH1B | 511.988112 | 224.5513646 | -1.189064823 | 6.27E-17 | 5.82E-16 |
| MCM7 | 6.708164992 | 19.39589773 | 1.531761474 | 7.56E-23 | 2.19E-21 |
| EPHB1 | 0.473927867 | 0.113197986 | -2.0658192 | 1.17E-17 | 1.24E-16 |
| GINS4 | 0.153570478 | 0.526983539 | 1.778856997 | 4.96E-14 | 3.01E-13 |
| CCDC185 | 0.002690915 | 0.052621339 | 4.289479461 | 0.003484058 | 0.005153314 |
| CDKL4 | 0.005235864 | 0.028744127 | 2.456767647 | 2.69E-05 | 5.32E-05 |
| STOX1 | 0.105109141 | 0.393459733 | 1.904327853 | 0.000338697 | 0.000581553 |
| TRIM17 | 0.017592707 | 0.199778185 | 3.505349692 | 5.25E-10 | 1.85E-09 |
| MAMSTR | 0.149207041 | 0.652222823 | 2.128049305 | 4.28E-19 | 5.70E-18 |
| MYOM3 | 0.016006777 | 0.043957912 | 1.45743998 | 3.00E-07 | 7.51E-07 |
| MCAM | 2.776375203 | 7.760134385 | 1.482879087 | 3.33E-17 | 3.27E-16 |
| PLPPR3 | 0.014499082 | 0.042678469 | 1.557546829 | 0.044076485 | 0.055270569 |
| EXPH5 | 1.440285467 | 0.551968293 | -1.383697484 | 6.85E-16 | 5.37E-15 |
| OLFML3 | 10.60743062 | 4.15325571 | -1.352760638 | 2.17E-21 | 4.44E-20 |
| KIR3DL1 | 0.050361139 | 0.020588118 | -1.290498951 | 7.44E-06 | 1.57E-05 |
| SRPX | 6.875977594 | 2.540266463 | -1.436585006 | 3.39E-24 | 1.43E-22 |
| LYPD1 | 0.161752431 | 2.938648327 | 4.183293416 | 2.85E-16 | 2.36E-15 |
| SIX4 | 0.018665602 | 0.209018038 | 3.485173538 | 4.22E-16 | 3.41E-15 |
| VN1R4 | 0.000508894 | 0.000143697 | -1.824338019 | 0.001405402 | 0.002206827 |
| LMNB1 | 2.654280433 | 6.624410086 | 1.319471182 | 1.36E-09 | 4.53E-09 |
| CACNG1 | 0.004561672 | 0.20962089 | 5.522075967 | 0.000173144 | 0.000309061 |
| AGPAT4 | 0.195606842 | 0.52330027 | 1.419682173 | 9.50E-07 | 2.24E-06 |
| KLK10 | 0.229974128 | 0.110377592 | -1.059024257 | 6.92E-17 | 6.38E-16 |
| RND3 | 41.13500671 | 8.992130607 | -2.193631783 | 3.38E-25 | 2.02E-23 |
| NKX3-1 | 1.674046434 | 0.58881898 | -1.507443464 | 4.19E-21 | 8.12E-20 |
| KCTD17 | 1.429324859 | 4.582363864 | 1.68075817 | 1.09E-05 | 2.26E-05 |
| TEK | 2.954471189 | 1.18015609 | -1.323922245 | 4.77E-20 | 7.52E-19 |
| ACTA1 | 0.077463688 | 0.220393493 | 1.508489535 | 1.94E-06 | 4.40E-06 |
| CDH8 | 0.001955565 | 0.019436935 | 3.313143075 | 4.60E-14 | 2.80E-13 |
| FBXL22 | 0.075991643 | 0.181663347 | 1.257354685 | 6.42E-14 | 3.84E-13 |
| GPR139 | 0.001328342 | 0.000377937 | -1.813409272 | 0.001976587 | 0.003029429 |
| CMC4 | 0.000458862 | 0.000202381 | -1.180982573 | 0.001787301 | 0.002758067 |
| HIST1H2AH | 0.006681973 | 0.078310606 | 3.55086168 | 1.80E-06 | 4.10E-06 |
| GINS2 | 0.764940216 | 2.584255061 | 1.756329565 | 5.57E-16 | 4.41E-15 |
| CHRM2 | 0.193825599 | 0.012069038 | -4.005376558 | 5.32E-28 | 1.50E-25 |
| SEZ6 | 0.009729986 | 1.639656336 | 7.396739963 | 2.92E-10 | 1.06E-09 |
| AXL | 5.223545778 | 2.162459618 | -1.272356258 | 2.78E-18 | 3.25E-17 |
| SAA4 | 292.5855664 | 114.8860359 | -1.348655148 | 1.31E-19 | 1.88E-18 |
| ADAMTSL2 | 12.5312918 | 4.775591108 | -1.391783922 | 1.30E-18 | 1.59E-17 |
| UMODL1 | 0.018637421 | 0.046842465 | 1.329614774 | 0.04137199 | 0.052063853 |
| CENPW | 1.289886556 | 7.830062359 | 2.601779609 | 6.34E-25 | 3.46E-23 |
| CTLA4 | 0.230647994 | 0.551688968 | 1.258162387 | 0.001295581 | 0.002045737 |
| MAGEA1 | 0.008240204 | 5.332074207 | 9.337801055 | 1.15E-11 | 5.02E-11 |
| MFSD2B | 0.045086262 | 0.179117928 | 1.990149952 | 4.16E-06 | 9.07E-06 |
| FOXP2 | 0.390175335 | 0.17360984 | -1.168273865 | 3.71E-13 | 2.00E-12 |
| WBSCR28 | 0.076611443 | 0.291024264 | 1.925507633 | 0.016430811 | 0.022010684 |
| IL1RN | 51.33776869 | 14.62968018 | -1.811122362 | 1.95E-16 | 1.67E-15 |
| PCDH11X | 0.002330002 | 0.000846331 | -1.461037388 | 0.000288336 | 0.000499158 |
| BCKDHB | 17.49007248 | 6.684574429 | -1.387628648 | 1.18E-24 | 5.83E-23 |
| C1QL4 | 0.012380644 | 0.215520321 | 4.121665668 | 3.22E-11 | 1.33E-10 |
| FAM13C | 0.141081029 | 0.294529071 | 1.061886041 | 0.00012092 | 0.000220163 |
| GIT1 | 2.691102118 | 5.398093809 | 1.004252911 | 3.81E-24 | 1.59E-22 |
| PROZ | 32.71215394 | 12.30929637 | -1.410078461 | 1.61E-18 | 1.94E-17 |
| HOXD8 | 0.039639275 | 0.418310453 | 3.399571552 | 2.88E-19 | 3.94E-18 |
| SLC39A2 | 0.053482846 | 0.018610907 | -1.522927832 | 7.09E-11 | 2.79E-10 |
| EGR1 | 139.7896977 | 33.26806538 | -2.071048163 | 5.64E-21 | 1.07E-19 |
| CD1D | 7.445276002 | 2.533355037 | -1.555276054 | 6.30E-22 | 1.46E-20 |
| DPYSL4 | 0.071130551 | 0.27692716 | 1.96096531 | 0.012572294 | 0.017115844 |
| TMEM154 | 0.837880507 | 0.241372341 | -1.795484143 | 8.05E-16 | 6.24E-15 |
| TSPAN11 | 0.251461831 | 0.112627874 | -1.158775508 | 9.45E-16 | 7.25E-15 |
| ADH4 | 736.0863876 | 222.2394071 | -1.727760437 | 8.62E-21 | 1.57E-19 |
| ADH1C | 461.8896962 | 230.8875738 | -1.000357833 | 1.30E-12 | 6.47E-12 |
| SPTY2D1-AS1 | 0.108603139 | 0.257734995 | 1.246822632 | 2.57E-11 | 1.08E-10 |
| BSG | 47.98019619 | 123.1165861 | 1.359514172 | 1.55E-22 | 4.22E-21 |
| STX16-NPEPL1 | 0.070787948 | 0.173123553 | 1.290226348 | 4.06E-09 | 1.28E-08 |
| YJEFN3 | 0.185927583 | 0.670649932 | 1.850819088 | 1.78E-15 | 1.31E-14 |
| AL020996.2 | 0.001333794 | 0.000247122 | -2.432239269 | 0.001266052 | 0.002001956 |
| HOMER3 | 1.499751432 | 3.671156256 | 1.291511112 | 6.71E-08 | 1.82E-07 |
| PDZD2 | 0.078710069 | 0.17505535 | 1.153191042 | 2.44E-06 | 5.47E-06 |
| C1orf35 | 1.815563041 | 4.419980201 | 1.283622882 | 3.74E-21 | 7.31E-20 |
| STMN1 | 2.799500041 | 12.5607489 | 2.165681377 | 3.43E-25 | 2.03E-23 |
| EPHB2 | 0.146461973 | 0.890532044 | 2.604141388 | 1.79E-05 | 3.62E-05 |
| NRCAM | 0.25231423 | 1.372182588 | 2.443178988 | 0.006198037 | 0.008832118 |
| HIST2H2BF | 0.043579938 | 0.196261324 | 2.171039856 | 2.43E-13 | 1.34E-12 |
| HOXA10 | 0.013437692 | 0.726019847 | 5.755651741 | 2.25E-15 | 1.63E-14 |
| KCNJ13 | 0.064694334 | 0.016073838 | -2.00892492 | 3.30E-08 | 9.32E-08 |
| CES5A | 1.800734155 | 0.686188982 | -1.391907343 | 8.15E-14 | 4.78E-13 |
| ANXA2R | 0.327348044 | 0.993971007 | 1.602378414 | 4.67E-16 | 3.74E-15 |
| ZNF385C | 0.070384826 | 0.288662254 | 2.03604613 | 1.95E-09 | 6.38E-09 |
| HOXA9 | 0.007811272 | 0.058600522 | 2.907284045 | 5.35E-07 | 1.30E-06 |
| CASS4 | 0.645592849 | 0.320159179 | -1.011835223 | 4.91E-13 | 2.61E-12 |
| AC007906.2 | 1.6349799 | 0.298777577 | -2.452129115 | 6.57E-26 | 5.16E-24 |
| IL36RN | 0.00828487 | 0.003353746 | -1.304705844 | 0.020070573 | 0.026505218 |
| HCN2 | 0.053496105 | 0.559765881 | 3.387317794 | 1.59E-12 | 7.83E-12 |
| CAMK2N2 | 0.358855408 | 1.790087142 | 2.318555251 | 1.75E-07 | 4.52E-07 |
| PPDPF | 58.17672528 | 125.0259118 | 1.103713131 | 1.06E-07 | 2.81E-07 |
| PIGC | 2.852255608 | 6.066291412 | 1.088711527 | 2.07E-24 | 9.47E-23 |
| PBLD | 45.94670135 | 16.80480039 | -1.451087884 | 2.96E-22 | 7.46E-21 |
| XKR3 | 0.00255591 | 0.03721201 | 3.863859397 | 0.025769338 | 0.033496479 |
| SLAIN1 | 1.631489262 | 0.553547879 | -1.55940948 | 1.27E-18 | 1.56E-17 |
| CCHCR1 | 2.2728025 | 6.016819639 | 1.404528787 | 1.12E-21 | 2.46E-20 |
| VEGFD | 0.402421165 | 2.400620921 | 2.576629519 | 0.030190957 | 0.038835131 |
| NUDT1 | 1.443795105 | 4.800153971 | 1.733214665 | 1.80E-21 | 3.76E-20 |
| RPLP0 | 142.2944453 | 303.1679987 | 1.091238131 | 9.27E-18 | 1.00E-16 |
| EFNB3 | 0.827193193 | 0.218631555 | -1.919722673 | 1.58E-26 | 1.64E-24 |
| MPP4 | 0.004142907 | 0.017991668 | 2.118613688 | 1.60E-10 | 6.01E-10 |
| BECN2 | 0.000721237 | 0.024655427 | 5.095288357 | 0.000289707 | 0.000501265 |
| SLC4A4 | 7.249854894 | 3.230682599 | -1.166113101 | 5.36E-17 | 5.04E-16 |
| SIK1 | 2.252928364 | 0.848114594 | -1.409470327 | 8.29E-05 | 0.000153803 |
| CLDN17 | 0.001308456 | 0.000272589 | -2.263067642 | 0.000635633 | 0.001051928 |
| PLEK2 | 12.28807538 | 5.98491976 | -1.037855161 | 2.41E-09 | 7.78E-09 |
| ASB18 | 0.001644259 | 0.00053221 | -1.627368677 | 5.81E-05 | 0.000110237 |
| TMEM220 | 25.68631755 | 10.34914024 | -1.311489156 | 1.02E-22 | 2.84E-21 |
| ATAT1 | 0.806529027 | 1.711626191 | 1.085569298 | 7.14E-17 | 6.56E-16 |
| C20orf204 | 0.033372221 | 1.134226942 | 5.086917802 | 6.05E-22 | 1.41E-20 |
| ZBTB21 | 3.793316797 | 1.468991518 | -1.368633797 | 1.31E-16 | 1.15E-15 |
| SAA2-SAA4 | 118.8369858 | 36.14100341 | -1.717275451 | 9.28E-08 | 2.47E-07 |
| RPL22L1 | 6.691263241 | 17.50774141 | 1.387642473 | 4.16E-07 | 1.03E-06 |
| CALCB | 0.020829935 | 0.059429945 | 1.512531735 | 0.000151656 | 0.000272456 |
| BHMT | 201.4394676 | 90.50418686 | -1.154289935 | 9.10E-15 | 6.09E-14 |
| HAND2 | 1.881087012 | 0.660004742 | -1.511018289 | 1.38E-23 | 4.90E-22 |
| TNFRSF25 | 0.560278786 | 1.64941897 | 1.557741132 | 1.49E-17 | 1.56E-16 |
| NSUN6 | 12.39070459 | 6.029051072 | -1.039255372 | 2.15E-19 | 2.99E-18 |
| RPE65 | 0.002339366 | 0.010167254 | 2.119740545 | 0.000952369 | 0.001536869 |
| SLC10A1 | 142.0926624 | 63.63842895 | -1.158861931 | 1.65E-14 | 1.06E-13 |
| SIGLEC7 | 2.17463286 | 0.540093871 | -2.00948977 | 1.04E-24 | 5.30E-23 |
| GPSM1 | 0.679336073 | 2.778761534 | 2.032244662 | 7.08E-12 | 3.19E-11 |
| IL5RA | 0.022752559 | 0.009708428 | -1.228719244 | 5.10E-11 | 2.05E-10 |
| ZBED2 | 0.025003177 | 0.152468834 | 2.608331026 | 6.79E-07 | 1.63E-06 |
| AMPD1 | 0.101600933 | 0.020574229 | -2.304003393 | 9.90E-17 | 8.88E-16 |
| GOLGA6B | 0.115151789 | 0.057480241 | -1.002398816 | 5.90E-07 | 1.43E-06 |
| FUT2 | 0.094206562 | 0.817473536 | 3.117272569 | 8.50E-14 | 4.97E-13 |
| DCX | 0.006467052 | 0.031854235 | 2.300305031 | 0.00025228 | 0.000440661 |
| CD4 | 31.56573983 | 9.988790839 | -1.659977612 | 8.94E-24 | 3.34E-22 |
| BRSK1 | 0.189221155 | 0.686975338 | 1.86018492 | 7.60E-20 | 1.14E-18 |
| HBA1 | 0.643087657 | 0.255437689 | -1.332043993 | 1.43E-13 | 8.12E-13 |
| GABRA3 | 0.002755001 | 0.219756464 | 6.317708993 | 0.000220991 | 0.000388188 |
| OR5P3 | 0.000429216 | 0.000161223 | -1.412644212 | 0.00141826 | 0.002225893 |
| TTC9 | 1.230278296 | 4.210037151 | 1.774848266 | 2.26E-07 | 5.76E-07 |
| LENEP | 0.001700004 | 0.040768534 | 4.583845667 | 1.18E-09 | 3.97E-09 |
| STX1A | 0.207225936 | 0.643805422 | 1.635420149 | 1.28E-21 | 2.77E-20 |
| CNBD2 | 0.030638551 | 0.080475549 | 1.393202457 | 2.37E-10 | 8.74E-10 |
| HIST2H3D | 0.024215151 | 0.10691917 | 2.142538644 | 2.11E-07 | 5.41E-07 |
| KIF4B | 0.001316915 | 0.004236238 | 1.685621472 | 0.016021586 | 0.021499684 |
| TSACC | 0.414124712 | 1.226225522 | 1.566087138 | 1.83E-11 | 7.81E-11 |
| DNMT3A | 0.828319 | 2.053169828 | 1.309594577 | 2.19E-19 | 3.04E-18 |
| SH3PXD2B | 0.691115383 | 1.744909763 | 1.336153934 | 4.39E-23 | 1.35E-21 |
| C1orf61 | 0.007288055 | 0.062713193 | 3.105163325 | 1.46E-05 | 2.99E-05 |
| ELFN2 | 0.057622437 | 0.816056415 | 3.823966314 | 4.59E-11 | 1.85E-10 |
| WNK4 | 0.107640961 | 1.48635374 | 3.787478425 | 2.29E-12 | 1.10E-11 |
| CNTN4 | 0.42621716 | 0.167074592 | -1.351096325 | 8.50E-24 | 3.21E-22 |
| ARHGAP28 | 0.046131559 | 0.118818767 | 1.364936774 | 0.001090363 | 0.001744715 |
| SLC22A16 | 0.007575339 | 0.019286496 | 1.348208724 | 0.009403193 | 0.013051583 |
| PRELID3A | 0.078718631 | 0.19508072 | 1.309294175 | 8.29E-05 | 0.000153803 |
| KLHL33 | 0.037333655 | 0.014748786 | -1.339880514 | 0.001578669 | 0.002457322 |
| ZNF251 | 1.082511353 | 2.585212005 | 1.25590044 | 2.49E-23 | 8.12E-22 |
| BMPER | 2.181111495 | 0.092491706 | -4.559595715 | 4.74E-30 | 2.52E-26 |
| CCSAP | 0.643833407 | 1.324260582 | 1.040427694 | 3.69E-13 | 1.99E-12 |
| BICD1 | 0.109972727 | 0.270984017 | 1.301061977 | 1.02E-12 | 5.18E-12 |
| RCL1 | 17.97467998 | 7.177126 | -1.324487932 | 6.59E-20 | 1.01E-18 |
| CCDC141 | 0.055597101 | 0.025406263 | -1.129825506 | 2.36E-11 | 9.94E-11 |
| CCDC183 | 0.1890323 | 0.562388406 | 1.572934081 | 9.41E-08 | 2.50E-07 |
| PTGER2 | 0.843918017 | 0.36389523 | -1.213579715 | 1.54E-16 | 1.34E-15 |
| DYDC2 | 0.080015261 | 0.900667841 | 3.492648054 | 7.54E-09 | 2.30E-08 |
| CALR | 261.112901 | 545.1371804 | 1.061945581 | 4.50E-20 | 7.11E-19 |
| ROBO1 | 0.838541266 | 6.513513954 | 2.957482381 | 2.57E-15 | 1.84E-14 |
| SPOCK1 | 0.033355848 | 0.774255565 | 4.536798204 | 6.88E-14 | 4.09E-13 |
| TIGD1 | 0.335238904 | 1.189593579 | 1.82720728 | 4.56E-22 | 1.08E-20 |
| HAPLN1 | 0.003576674 | 0.12312376 | 5.105346627 | 2.12E-05 | 4.23E-05 |
| RNASE1 | 18.68586195 | 39.48915557 | 1.079509403 | 0.001041713 | 0.001671683 |
| SNX22 | 0.614816051 | 2.145047562 | 1.802782902 | 7.25E-09 | 2.22E-08 |
| CDC25B | 5.826629192 | 12.39547504 | 1.089080157 | 9.96E-10 | 3.39E-09 |
| KIAA1324 | 0.112429137 | 0.370511774 | 1.720503418 | 2.16E-06 | 4.87E-06 |
| GABRR3 | 0.008271766 | 0.138931003 | 4.070029449 | 3.11E-05 | 6.12E-05 |
| PRKCB | 0.792843869 | 0.358221359 | -1.14618543 | 4.53E-16 | 3.63E-15 |
| GAS2L3 | 0.360198832 | 0.827175463 | 1.199399888 | 2.63E-10 | 9.63E-10 |
| NODAL | 0.016259111 | 0.054490091 | 1.744745504 | 7.82E-07 | 1.86E-06 |
| MAGEC2 | 0.010054734 | 4.770604678 | 8.89015346 | 1.64E-06 | 3.74E-06 |
| CEBPA | 36.33053268 | 73.23428664 | 1.011336725 | 4.97E-06 | 1.07E-05 |
| GIPC2 | 7.26537752 | 3.123969389 | -1.217657447 | 3.68E-21 | 7.20E-20 |
| ARL17A | 0.027986501 | 0.062551158 | 1.160305479 | 7.31E-10 | 2.53E-09 |
| HAUS5 | 1.346546074 | 2.724389654 | 1.016669464 | 5.97E-22 | 1.40E-20 |
| PRNT | 0.001331648 | 0.000301202 | -2.144408752 | 0.0091653 | 0.0127441 |
| KCNN3 | 0.082411751 | 0.260386192 | 1.65973098 | 4.72E-18 | 5.34E-17 |
| SERPINH1 | 9.001426295 | 22.11179664 | 1.296590729 | 1.65E-12 | 8.10E-12 |
| DDX39A | 5.069457729 | 14.31160054 | 1.497281687 | 1.62E-23 | 5.63E-22 |
| C2orf40 | 0.457016193 | 0.184808895 | -1.306211088 | 3.01E-17 | 2.98E-16 |
| AQP8 | 0.253851994 | 3.235001747 | 3.671706992 | 8.89E-06 | 1.86E-05 |
| KCNK9 | 0.003062503 | 0.275483812 | 6.491112273 | 7.95E-12 | 3.56E-11 |
| KIF11 | 0.364953889 | 1.731904449 | 2.246573238 | 3.84E-24 | 1.59E-22 |
| RD3L | 0.207901128 | 0.009851672 | -4.399385199 | 4.51E-26 | 3.83E-24 |
| NPIPB11 | 0.053933424 | 0.1107403 | 1.037928804 | 0.000324627 | 0.000558448 |
| KIR3DL2 | 0.032316884 | 0.016044229 | -1.010233634 | 0.001994486 | 0.003054755 |
| RXFP4 | 0.003349154 | 0.058007105 | 4.114360904 | 0.000102125 | 0.000187565 |
| HIST1H3B | 0.078894848 | 0.84434687 | 3.419832813 | 1.00E-15 | 7.67E-15 |
| TMEM98 | 3.677448008 | 8.834735264 | 1.26448196 | 9.22E-09 | 2.78E-08 |
| VSIG4 | 12.71709237 | 3.876736624 | -1.71385422 | 1.73E-19 | 2.44E-18 |
| CA5B | 0.215151837 | 0.539713168 | 1.326837728 | 5.44E-08 | 1.50E-07 |
| SLITRK4 | 0.01737343 | 0.059308446 | 1.771354956 | 0.044335393 | 0.055568359 |
| PDCD1LG2 | 1.160453772 | 0.573206427 | -1.017562361 | 6.89E-16 | 5.39E-15 |
| FTL | 4737.677836 | 12015.60668 | 1.34265749 | 4.03E-14 | 2.47E-13 |
| LCE2D | 0.129157923 | 0.063668899 | -1.020475411 | 1.71E-08 | 4.99E-08 |
| FANCI | 0.3295295 | 1.643793648 | 2.318549676 | 5.66E-26 | 4.58E-24 |
| FNIP2 | 8.31531012 | 3.733395024 | -1.155281904 | 3.47E-17 | 3.40E-16 |
| FAM90A1 | 0.040592973 | 0.10641115 | 1.390347403 | 0.001357961 | 0.002136862 |
| CACNA1S | 0.004264888 | 0.024034248 | 2.494511951 | 2.82E-07 | 7.08E-07 |
| RHO | 0.002441976 | 0.053083319 | 4.442137956 | 2.89E-11 | 1.20E-10 |
| AL163636.2 | 1.72752796 | 0.696693422 | -1.310113215 | 1.18E-18 | 1.45E-17 |
| RPS18 | 232.4745024 | 620.5850804 | 1.416556522 | 4.08E-19 | 5.45E-18 |
| POPDC3 | 0.005944268 | 0.600437254 | 6.658370463 | 6.20E-05 | 0.000117042 |
| TNNI2 | 0.314646149 | 1.986431504 | 2.658376856 | 2.00E-07 | 5.14E-07 |
| OTX1 | 0.012597017 | 0.281432416 | 4.481634481 | 9.86E-21 | 1.78E-19 |
| SLC10A4 | 0.011481129 | 0.055629633 | 2.276589075 | 8.33E-05 | 0.000154535 |
| TREX2 | 0.060228863 | 0.201109568 | 1.739454784 | 1.06E-06 | 2.48E-06 |
| DSCC1 | 0.351951125 | 1.512021641 | 2.103031785 | 5.33E-26 | 4.41E-24 |
| DYNC1I1 | 0.089798378 | 1.295225737 | 3.850370359 | 1.48E-06 | 3.40E-06 |
| GDAP1 | 0.634182296 | 1.292428782 | 1.027115276 | 2.60E-05 | 5.15E-05 |
| ILF2 | 24.45671096 | 55.97541507 | 1.194562923 | 8.70E-26 | 6.57E-24 |
| GPR148 | 0.000748804 | 0.000227099 | -1.721266797 | 0.000769313 | 0.001259494 |
| ACSM1 | 5.442769101 | 14.71331071 | 1.434709172 | 0.020915905 | 0.027530427 |
| HIST1H3A | 0.094144301 | 0.20057008 | 1.091160741 | 0.015076529 | 0.020313557 |
| CENPM | 0.33231002 | 3.828070477 | 3.526015691 | 3.17E-25 | 1.93E-23 |
| APLN | 0.184767674 | 2.646813421 | 3.840472226 | 1.25E-25 | 8.93E-24 |
| FAM72D | 0.01171756 | 0.180729317 | 3.947086412 | 1.65E-23 | 5.68E-22 |
| SPSB4 | 0.188805235 | 0.02849125 | -2.728307925 | 5.27E-25 | 2.96E-23 |
| MMP27 | 0.016879631 | 0.007533173 | -1.163953733 | 0.00020921 | 0.000368742 |
| FOXK1 | 0.667025765 | 1.565188994 | 1.230522478 | 3.20E-21 | 6.33E-20 |
| AC106782.1 | 0.004660494 | 0.016258468 | 1.802636529 | 0.000727065 | 0.001194512 |
| SOX12 | 2.14127564 | 5.713814947 | 1.415983791 | 2.90E-12 | 1.37E-11 |
| ALDH3A1 | 1.138482136 | 51.19720534 | 5.490881502 | 0.027064775 | 0.035060264 |
| C22orf29 | 0.961673593 | 2.082424623 | 1.114645067 | 1.58E-20 | 2.73E-19 |
| LRRC37A3 | 0.144644472 | 0.413915236 | 1.516824167 | 9.14E-13 | 4.66E-12 |
| TRIM50 | 0.064074545 | 2.731396745 | 5.413743742 | 0.000267197 | 0.000464787 |
| PRDM9 | 0.002563849 | 0.039274813 | 3.937221151 | 1.44E-06 | 3.32E-06 |
| HDAC11 | 1.328030081 | 4.362695309 | 1.715931894 | 1.13E-23 | 4.11E-22 |
| KAAG1 | 0.141302089 | 0.515096178 | 1.866059044 | 0.001953909 | 0.002996443 |
| CRLF1 | 0.152512643 | 0.8426612 | 2.466023858 | 3.88E-12 | 1.81E-11 |
| COL1A2 | 8.260190399 | 27.56262746 | 1.738466481 | 0.009072154 | 0.012626997 |
| ATP5J2-PTCD1 | 0.016029201 | 0.057797969 | 1.850316238 | 4.60E-12 | 2.13E-11 |
| LDLRAD1 | 0.070398749 | 1.321945469 | 4.230969055 | 5.37E-14 | 3.24E-13 |
| DKK2 | 0.068022859 | 0.233986946 | 1.782336507 | 0.000292515 | 0.000505829 |
| FAM227A | 0.012736247 | 0.036605104 | 1.523104669 | 1.43E-05 | 2.93E-05 |
| OR5B17 | 0.000853608 | 2.63E-05 | -5.022523958 | 0.000333854 | 0.000573744 |
| CYP4A22 | 73.17277217 | 20.09192362 | -1.864691221 | 2.98E-23 | 9.53E-22 |
| CLEC12A | 0.745061642 | 0.233686342 | -1.672786371 | 6.33E-20 | 9.74E-19 |
| ERVFRD-1 | 0.099123983 | 0.026592278 | -1.898226795 | 1.03E-20 | 1.85E-19 |
| CKM | 0.029401047 | 0.087576545 | 1.574677019 | 0.005026739 | 0.007254746 |
| PRND | 0.011972478 | 0.21685854 | 4.178960615 | 2.89E-14 | 1.81E-13 |
| GDAP1L1 | 0.005258908 | 0.05312846 | 3.336649715 | 2.37E-05 | 4.72E-05 |
| ZNF208 | 0.015428863 | 0.069917312 | 2.180017939 | 0.002754044 | 0.004139487 |
| HIST1H3H | 0.746169061 | 2.942983304 | 1.97970491 | 1.29E-08 | 3.81E-08 |
| TNFSF11 | 0.98807709 | 0.452875912 | -1.1255078 | 1.69E-12 | 8.26E-12 |
| FAM57B | 0.003013853 | 0.062140495 | 4.365852618 | 7.96E-22 | 1.82E-20 |
| TMEM25 | 2.725306106 | 1.340505549 | -1.02364109 | 3.30E-15 | 2.33E-14 |
| GDF3 | 0.008561572 | 0.021850922 | 1.351746448 | 0.023872146 | 0.031142282 |
| GAL3ST4 | 0.316294611 | 0.765646791 | 1.275410025 | 6.96E-05 | 0.000130534 |
| PYGB | 6.292025298 | 18.06904473 | 1.52192386 | 9.92E-21 | 1.79E-19 |
| C9orf50 | 0.015261443 | 0.050799087 | 1.734911169 | 3.01E-05 | 5.93E-05 |
| STARD6 | 0.00258093 | 0.025817293 | 3.322374776 | 0.000901535 | 0.001458984 |
| C11orf96 | 21.47983145 | 6.604451438 | -1.701472031 | 3.64E-15 | 2.56E-14 |
| ABCA4 | 0.131869746 | 0.326796055 | 1.309276954 | 0.000751842 | 0.001233097 |
| GJC3 | 1.28840536 | 0.633768085 | -1.023559652 | 4.50E-15 | 3.12E-14 |
| FRMD4B | 4.211207129 | 1.695958655 | -1.312132837 | 1.33E-24 | 6.54E-23 |
| SMYD2 | 5.687369951 | 11.65371935 | 1.034956916 | 5.99E-11 | 2.38E-10 |
| TICRR | 0.021952258 | 0.317043684 | 3.852240368 | 2.10E-27 | 3.71E-25 |
| ZMYND10 | 0.072035448 | 0.228957688 | 1.668302084 | 6.20E-12 | 2.81E-11 |
| SLC22A1 | 302.8734346 | 80.76771361 | -1.906864438 | 1.22E-20 | 2.16E-19 |
| BMPR1B | 0.172114314 | 0.083070943 | -1.050951238 | 1.58E-23 | 5.51E-22 |
| ZSCAN31 | 1.023009232 | 2.620103848 | 1.35680483 | 0.000173227 | 0.000309104 |
| UGT2A1 | 1.535132415 | 0.71073232 | -1.110984892 | 7.83E-06 | 1.65E-05 |
| SPINK14 | 0.059397177 | 0.160929062 | 1.437958617 | 0.010077571 | 0.013930418 |
| OR2W1 | 0.000440658 | 0.000151192 | -1.543280112 | 0.001164091 | 0.001853728 |
| HBA2 | 24.4021182 | 8.634190991 | -1.498873473 | 3.71E-20 | 5.97E-19 |
| PNRC1 | 61.12792347 | 29.4003119 | -1.056000099 | 2.45E-11 | 1.03E-10 |
| UBL4B | 0.002458889 | 0.053123493 | 4.433271415 | 0.00128982 | 0.002037614 |
| DKK1 | 0.128171956 | 8.535891875 | 6.057389367 | 1.10E-06 | 2.57E-06 |
| HIST1H1B | 0.011986963 | 0.132574733 | 3.467267712 | 5.42E-12 | 2.48E-11 |
| SLC28A1 | 22.84198935 | 10.86681301 | -1.071759411 | 1.61E-13 | 9.08E-13 |
| TRPC6 | 0.035223359 | 0.169099322 | 2.263266473 | 1.65E-23 | 5.68E-22 |
| ASF1B | 0.509150258 | 4.10653066 | 3.011756683 | 4.10E-25 | 2.36E-23 |
| OR8B2 | 0.000593444 | 0.000264912 | -1.163599072 | 0.001609365 | 0.002501848 |
| NTM | 0.035586243 | 0.284395958 | 2.998509427 | 1.65E-06 | 3.77E-06 |
| C4orf48 | 0.922590848 | 3.414002834 | 1.88770137 | 1.70E-05 | 3.45E-05 |
| SNCAIP | 0.164424665 | 0.495320013 | 1.590934186 | 0.034087215 | 0.043437537 |
| CCL25 | 0.223072519 | 21.77697438 | 6.609146918 | 3.49E-08 | 9.82E-08 |
| CPNE5 | 0.248837092 | 0.519911857 | 1.063065506 | 2.69E-07 | 6.78E-07 |
| HPDL | 0.059590125 | 0.326297217 | 2.453041506 | 4.30E-14 | 2.63E-13 |
| TNNC1 | 0.318638707 | 2.466470691 | 2.95245471 | 0.002710026 | 0.004078408 |
| CDC20 | 0.478595574 | 9.6848305 | 4.338847838 | 3.81E-27 | 5.71E-25 |
| RAB11FIP4 | 0.701697055 | 2.478616707 | 1.820614978 | 6.41E-19 | 8.25E-18 |
| CLIC5 | 0.133131868 | 0.37617212 | 1.498536975 | 2.04E-05 | 4.10E-05 |
| KRT71 | 0.004403001 | 0.002061176 | -1.095019365 | 0.000944928 | 0.001525337 |
| OCLM | 0.0666253 | 0.135190922 | 1.020856263 | 0.001168548 | 0.001860072 |
| DMD | 4.065629985 | 1.69990412 | -1.258025545 | 7.23E-22 | 1.67E-20 |
| SEC14L5 | 0.034212076 | 0.083568641 | 1.288456048 | 0.02899535 | 0.037399282 |
| ITGA9 | 4.519414969 | 1.324155194 | -1.771063811 | 1.49E-24 | 7.13E-23 |
| NTF3 | 2.159840692 | 0.193348158 | -3.481651976 | 3.10E-29 | 3.71E-26 |
| ABCB9 | 0.110939875 | 0.234992994 | 1.082839736 | 1.04E-09 | 3.54E-09 |
| INMT | 12.91904052 | 2.561555429 | -2.334406911 | 9.36E-25 | 4.87E-23 |
| PLPPR1 | 1.67067384 | 5.200406021 | 1.638194158 | 5.78E-05 | 0.000109548 |
| ZSWIM5 | 0.320324885 | 1.225418175 | 1.935666368 | 4.58E-08 | 1.27E-07 |
| GAS1 | 1.185787768 | 0.536449076 | -1.144332688 | 1.15E-14 | 7.58E-14 |
| ADRA1B | 5.638029861 | 1.981395888 | -1.508673956 | 1.06E-20 | 1.90E-19 |
| PSMG3 | 6.20394743 | 12.66474034 | 1.029559132 | 2.35E-16 | 1.98E-15 |
| KLHL31 | 0.072019092 | 0.210752143 | 1.54909599 | 5.21E-13 | 2.75E-12 |
| RPSA | 46.73053444 | 103.469868 | 1.146773252 | 3.14E-17 | 3.10E-16 |
| OR5C1 | 0.007587091 | 0.003584223 | -1.081886322 | 0.035323104 | 0.044902104 |
| CCDC36 | 0.01554103 | 0.041006728 | 1.399778504 | 0.000406501 | 0.000691411 |
| THBS1 | 30.61923085 | 11.98487539 | -1.353223134 | 6.84E-15 | 4.63E-14 |
| CYP4F2 | 73.35758786 | 29.50666284 | -1.313905437 | 4.73E-19 | 6.22E-18 |
| GALR2 | 0.047380009 | 0.144230214 | 1.606023037 | 1.72E-05 | 3.48E-05 |
| C5orf46 | 0.033245575 | 0.552867792 | 4.055700276 | 2.62E-16 | 2.19E-15 |
| SELENOM | 3.637301097 | 11.97030801 | 1.718520012 | 0.002202199 | 0.003353741 |
| STX11 | 2.742312863 | 1.114905816 | -1.298471334 | 4.42E-17 | 4.24E-16 |
| NUDT10 | 0.453112875 | 0.054900389 | -3.044982196 | 3.61E-27 | 5.57E-25 |
| RASGEF1B | 5.907292019 | 1.744083089 | -1.760028159 | 4.44E-24 | 1.80E-22 |
| ZNF692 | 1.65427312 | 5.26337907 | 1.669791859 | 5.01E-25 | 2.83E-23 |
| FOXC2 | 0.07596113 | 0.274062537 | 1.851171867 | 5.52E-09 | 1.71E-08 |
| TACC3 | 0.996493882 | 4.326114168 | 2.118138886 | 9.28E-24 | 3.44E-22 |
| NME1 | 7.37715674 | 16.48087414 | 1.159655971 | 3.25E-15 | 2.30E-14 |
| FCN1 | 1.569210228 | 0.660632309 | -1.248119212 | 1.58E-12 | 7.78E-12 |
| PAGE4 | 0.033410463 | 14.17247939 | 8.728576481 | 0.000155434 | 0.000278792 |
| PANX3 | 0.001075539 | 0.022075103 | 4.359288057 | 0.040339832 | 0.050830737 |
| PCDHB2 | 0.04438166 | 0.345283375 | 2.959745333 | 4.40E-06 | 9.56E-06 |
| DRGX | 0.001944897 | 0.131450437 | 6.078681321 | 8.22E-06 | 1.73E-05 |
| TLX1 | 0.150006825 | 1.103234936 | 2.878639997 | 7.24E-12 | 3.26E-11 |
| HCRTR2 | 0.007631018 | 0.003566597 | -1.097327452 | 0.000569643 | 0.000949791 |
| CHTF18 | 0.544291093 | 1.786401497 | 1.714606033 | 1.06E-23 | 3.85E-22 |
| CD163 | 16.34179319 | 4.669195203 | -1.80732049 | 1.17E-18 | 1.44E-17 |
| PPEF1 | 0.010388059 | 0.057069504 | 2.457793843 | 7.71E-09 | 2.35E-08 |
| PRG3 | 0.004818535 | 0.001488669 | -1.694571403 | 0.000201121 | 0.000355411 |
| ARL16 | 3.481286216 | 7.780540055 | 1.160249867 | 1.34E-20 | 2.35E-19 |
| ATP8B3 | 0.164886408 | 0.377965499 | 1.196782076 | 6.36E-06 | 1.36E-05 |
| SAPCD2 | 0.124385835 | 0.858780855 | 2.787467833 | 2.55E-19 | 3.52E-18 |
| SLC5A2 | 0.040144822 | 0.111344995 | 1.471750896 | 0.000174935 | 0.000311828 |
| HIST1H2AD | 0.229679791 | 0.96086626 | 2.064711715 | 4.02E-09 | 1.26E-08 |
| XCR1 | 0.20614227 | 0.101769737 | -1.018331756 | 1.56E-08 | 4.56E-08 |
| COL21A1 | 0.178029176 | 0.487766881 | 1.45407811 | 0.000178398 | 0.000317601 |
| MUT | 56.93825489 | 27.91904352 | -1.028148759 | 3.77E-20 | 6.06E-19 |
| COX4I2 | 0.449275412 | 3.056400794 | 2.766161729 | 8.70E-23 | 2.47E-21 |
| CRHBP | 26.58893538 | 1.398826214 | -4.24853737 | 5.25E-29 | 4.80E-26 |
| TTC34 | 0.023495529 | 0.049896465 | 1.086551327 | 0.044076376 | 0.055270569 |
| FPR2 | 0.390904796 | 0.054341345 | -2.846695102 | 1.23E-20 | 2.16E-19 |
| PARM1 | 0.647407076 | 1.481945214 | 1.194747075 | 0.009420052 | 0.013071456 |
| ANKS6 | 0.523339116 | 1.347028876 | 1.363962778 | 2.33E-07 | 5.94E-07 |
| Z83844.1 | 0.002462806 | 0.006072319 | 1.3019448 | 0.000137675 | 0.00024881 |
| GSTM5 | 0.551570062 | 0.241497538 | -1.191535674 | 1.52E-14 | 9.86E-14 |
| NKPD1 | 0.005950779 | 0.032842973 | 2.464434256 | 1.39E-06 | 3.20E-06 |
| HOXB7 | 0.325825556 | 1.068213403 | 1.71302822 | 0.000281592 | 0.00048819 |
| KRTAP9-3 | 0.000676609 | 0.000249611 | -1.438640454 | 0.002244751 | 0.003415199 |
| LSM4 | 15.46030925 | 32.18979924 | 1.058034401 | 1.08E-20 | 1.93E-19 |
| GDPD1 | 0.374959985 | 0.850416176 | 1.181432396 | 3.26E-11 | 1.34E-10 |
| ZNF705E | 0.010896764 | 0.024696403 | 1.180401169 | 0.00085323 | 0.001385521 |
| ACTL8 | 0.005666696 | 1.10563052 | 7.608145895 | 0.000220401 | 0.000387195 |
| BEND3 | 0.247321257 | 0.562868427 | 1.186411481 | 2.46E-12 | 1.18E-11 |
| DDX4 | 0.002857053 | 0.016623482 | 2.54062293 | 0.025690168 | 0.033399158 |
| QRFP | 0.028728433 | 0.074481348 | 1.374399866 | 1.01E-08 | 3.05E-08 |
| ACE | 0.401389969 | 0.826909071 | 1.042724131 | 4.16E-10 | 1.49E-09 |
| GOLM1 | 8.590129641 | 23.51279877 | 1.452694466 | 8.27E-06 | 1.74E-05 |
| COX6B2 | 0.008531609 | 0.048449886 | 2.505603519 | 1.42E-06 | 3.27E-06 |
| FOS | 124.0125334 | 21.46899004 | -2.530159705 | 9.89E-23 | 2.76E-21 |
| RGS5 | 5.257136795 | 15.23247052 | 1.534800767 | 2.75E-12 | 1.30E-11 |
| IDNK | 15.9085039 | 7.548571275 | -1.07552265 | 1.15E-19 | 1.67E-18 |
| CLEC18A | 0.004881874 | 0.016240102 | 1.734053798 | 1.14E-08 | 3.39E-08 |
| AC092821.1 | 0.006873315 | 0.040373788 | 2.55434098 | 6.68E-21 | 1.25E-19 |
| FLRT1 | 0.035780151 | 0.105904893 | 1.565537859 | 1.72E-08 | 5.00E-08 |
| OR13C2 | 0.001120071 | 0.000302035 | -1.890800189 | 0.003246383 | 0.004820312 |
| KPNA7 | 0.179425861 | 0.795317424 | 2.148142935 | 1.40E-09 | 4.67E-09 |
| CASQ1 | 0.030611734 | 0.104745296 | 1.774728802 | 1.34E-05 | 2.74E-05 |
| GPR83 | 0.091809078 | 0.02694684 | -1.768520719 | 5.31E-17 | 4.99E-16 |
| HIST1H2BF | 0.042306891 | 0.261558683 | 2.628170093 | 1.14E-09 | 3.86E-09 |
| SERPINI2 | 0.00096606 | 0.020576807 | 4.412762806 | 0.001387136 | 0.002179819 |
| CILP | 0.546050538 | 0.20139284 | -1.43902209 | 5.02E-20 | 7.88E-19 |
| KRT23 | 3.734221694 | 13.86081132 | 1.892132221 | 0.011851048 | 0.016190605 |
| MCC | 3.928317754 | 1.219334305 | -1.687817908 | 1.00E-24 | 5.16E-23 |
| COL17A1 | 0.038420607 | 0.084907634 | 1.144013956 | 0.000150535 | 0.00027063 |
| CHGA | 0.011553571 | 1.736954113 | 7.23207704 | 3.81E-11 | 1.55E-10 |
| KLHDC7B | 0.301320423 | 0.920370511 | 1.610916303 | 0.000323865 | 0.000557378 |
| LRRN2 | 0.18825663 | 0.686646175 | 1.866866203 | 8.73E-05 | 0.000161482 |
| TP73 | 0.041358832 | 0.494847396 | 3.580716345 | 1.03E-20 | 1.85E-19 |
| PERM1 | 0.076724063 | 0.249449048 | 1.700994138 | 2.43E-05 | 4.82E-05 |
| SLC25A47 | 257.2577009 | 56.17999523 | -2.195085857 | 2.05E-23 | 6.86E-22 |
| RFX6 | 0.005924528 | 0.073543776 | 3.633831032 | 0.017362849 | 0.023153442 |
| SOSTDC1 | 0.032805328 | 0.290762024 | 3.147836816 | 6.38E-05 | 0.00012023 |
| NRM | 2.32307031 | 6.66250591 | 1.520032088 | 1.36E-14 | 8.87E-14 |
| SNRPE | 15.34010301 | 36.86859425 | 1.265084239 | 2.22E-24 | 1.01E-22 |
| CCR8 | 0.024849965 | 0.073936863 | 1.573050013 | 5.82E-06 | 1.25E-05 |
| MT1G | 926.0807303 | 316.8411152 | -1.547378404 | 1.96E-23 | 6.58E-22 |
| SRD5A1 | 13.72159459 | 5.807142588 | -1.240547784 | 2.03E-19 | 2.83E-18 |
| ASPM | 0.128310165 | 1.83332643 | 3.836756309 | 8.71E-27 | 1.04E-24 |
| TUBB | 80.59545161 | 165.7689402 | 1.040403389 | 3.57E-13 | 1.93E-12 |
| VMO1 | 8.548432803 | 3.375199367 | -1.340687231 | 2.33E-20 | 3.92E-19 |
| IFIT1 | 18.62744403 | 9.230194499 | -1.012996774 | 3.60E-06 | 7.92E-06 |
| CDX1 | 0.065347547 | 0.17938965 | 1.456891675 | 4.31E-10 | 1.54E-09 |
| SLC13A4 | 0.039326943 | 0.094813362 | 1.269572354 | 7.93E-10 | 2.73E-09 |
| ERAS | 0.037873362 | 0.077471639 | 1.032484771 | 0.014036049 | 0.018993899 |
| ANKRD23 | 0.063659215 | 0.16774165 | 1.397799685 | 4.49E-17 | 4.30E-16 |
| RXFP2 | 0.000493296 | 0.000176143 | -1.485705836 | 0.004525495 | 0.006577081 |
| AC244197.3 | 0.056066075 | 0.152523377 | 1.443830403 | 8.97E-08 | 2.40E-07 |
| HSD17B1 | 0.200228613 | 0.418296042 | 1.062876196 | 2.79E-07 | 7.02E-07 |
| SLC47A2 | 0.037620127 | 0.09564191 | 1.346138215 | 1.61E-05 | 3.26E-05 |
| EPHA2 | 19.76661713 | 5.429615575 | -1.864144027 | 1.86E-21 | 3.86E-20 |
| ARHGEF39 | 0.098284951 | 0.814310683 | 3.050536896 | 1.46E-28 | 8.50E-26 |
| SPERT | 0.000649383 | 0.070940043 | 6.771387366 | 0.000413139 | 0.000702012 |
| DSTYK | 0.676111546 | 1.392458261 | 1.042300894 | 1.20E-18 | 1.47E-17 |
| AL121758.1 | 0.019809344 | 0.057938447 | 1.548339921 | 1.53E-06 | 3.51E-06 |
| KCNG2 | 0.023015641 | 0.071216179 | 1.629590413 | 1.14E-07 | 3.01E-07 |
| AC138894.1 | 0.008656226 | 0.028202612 | 1.70401872 | 0.000891627 | 0.001444219 |
| ANXA2 | 14.93772682 | 38.01419368 | 1.347577571 | 1.36E-13 | 7.74E-13 |
| LINGO1 | 0.272689957 | 1.616078541 | 2.567163842 | 1.87E-11 | 7.97E-11 |
| IRX5 | 0.064704684 | 0.182052781 | 1.492414725 | 7.27E-09 | 2.22E-08 |
| ASIC4 | 0.002980831 | 0.011951066 | 2.003352588 | 0.00020902 | 0.000368451 |
| ARID3C | 6.597782329 | 2.336001147 | -1.497940199 | 6.16E-20 | 9.50E-19 |
| CENPX | 16.73996673 | 37.04663295 | 1.146045766 | 5.66E-11 | 2.26E-10 |
| VSIG1 | 0.057431648 | 1.200763696 | 4.385962504 | 3.53E-07 | 8.77E-07 |
| KLHDC8A | 0.02855919 | 0.14994367 | 2.392393662 | 0.003996651 | 0.0058605 |
| AMN | 14.99659974 | 7.337266224 | -1.03132089 | 1.46E-06 | 3.35E-06 |
| CREG2 | 0.004282131 | 0.037867939 | 3.144576069 | 3.82E-17 | 3.70E-16 |
| MT1H | 164.2489466 | 25.71713701 | -2.675082171 | 5.00E-24 | 2.00E-22 |
| RGS20 | 0.008989515 | 0.061988011 | 2.785673984 | 1.58E-10 | 5.94E-10 |
| BBOX1 | 17.19335819 | 5.512195253 | -1.641152461 | 9.45E-17 | 8.49E-16 |
| PLK5 | 0.014774052 | 0.076342159 | 2.369414433 | 5.81E-08 | 1.59E-07 |
| MSH2 | 1.455288199 | 3.609998339 | 1.310693287 | 1.20E-25 | 8.63E-24 |
| PDE4C | 0.052881116 | 0.220826579 | 2.062089294 | 1.52E-17 | 1.59E-16 |
| GOLGA8B | 0.811071601 | 2.53276266 | 1.642810705 | 1.14E-16 | 1.02E-15 |
| KCNN1 | 0.015133426 | 0.103738585 | 2.777142085 | 1.16E-09 | 3.92E-09 |
| CNTN3 | 0.817501396 | 0.321695966 | -1.34552335 | 9.70E-15 | 6.47E-14 |
| CES3 | 13.85432596 | 6.81313946 | -1.02394488 | 7.77E-14 | 4.58E-13 |
| SMG5 | 9.748440508 | 26.3314427 | 1.433543219 | 1.13E-24 | 5.65E-23 |
| ACSM6 | 0.010585666 | 0.036521956 | 1.78665202 | 0.040884482 | 0.05148784 |
| FAM236A | 0.000816236 | 0.000229354 | -1.831409644 | 0.00058569 | 0.000974628 |
| FAM229A | 0.371922518 | 0.79633379 | 1.098371179 | 3.15E-10 | 1.14E-09 |
| DYDC1 | 0.006541436 | 0.065458146 | 3.322893528 | 3.56E-05 | 6.95E-05 |
| HEY1 | 0.74032501 | 1.882183632 | 1.346176716 | 1.37E-14 | 8.91E-14 |
| KIR3DL3 | 0.00693967 | 0.001533871 | -2.177689785 | 7.22E-06 | 1.53E-05 |
| STC1 | 1.508010169 | 3.313153043 | 1.13555869 | 1.13E-07 | 2.98E-07 |
| CTNNA3 | 0.746457264 | 0.166994023 | -2.160263201 | 1.53E-19 | 2.18E-18 |
| TXNRD1 | 9.604627631 | 27.59078761 | 1.522385054 | 5.00E-09 | 1.55E-08 |
| TMEM108 | 0.021514403 | 0.062817024 | 1.545852755 | 5.95E-08 | 1.63E-07 |
| HPSE2 | 0.003801099 | 0.03168915 | 3.059500532 | 2.79E-06 | 6.22E-06 |
| CSF3 | 0.093231961 | 0.013055324 | -2.836186354 | 1.50E-09 | 4.97E-09 |
| MAPK8IP3 | 1.217240877 | 2.441464324 | 1.004132009 | 4.63E-13 | 2.47E-12 |
| PODXL | 1.877410291 | 5.869751902 | 1.644555553 | 5.28E-17 | 4.97E-16 |
| CNDP1 | 9.5823342 | 1.122189518 | -3.094060789 | 7.84E-24 | 2.98E-22 |
| SIGLEC1 | 3.275247272 | 1.327794207 | -1.302572267 | 1.34E-15 | 1.00E-14 |
| RPS19 | 84.08798193 | 194.0491626 | 1.206450681 | 1.49E-15 | 1.10E-14 |
| PLEKHN1 | 0.099262569 | 0.386919123 | 1.962710342 | 1.53E-09 | 5.07E-09 |
| INHBC | 46.07518203 | 22.34683306 | -1.043919475 | 1.58E-13 | 8.95E-13 |
| ALX3 | 0.000587883 | 0.1045789 | 7.474846058 | 0.009331794 | 0.012960547 |
| DNMT3L | 0.713558945 | 0.324506197 | -1.136786583 | 1.49E-07 | 3.88E-07 |
| CCDC40 | 0.150598984 | 0.319138008 | 1.083468402 | 6.02E-11 | 2.39E-10 |
| SULT1A1 | 18.57891556 | 8.952205747 | -1.053351196 | 2.68E-17 | 2.69E-16 |
| SNRPC | 30.44603111 | 63.98000283 | 1.071366883 | 4.88E-25 | 2.78E-23 |
| SSR2 | 16.21953777 | 40.11748884 | 1.306498597 | 8.08E-25 | 4.26E-23 |
| FAM178B | 0.011709928 | 0.310921538 | 4.730746414 | 0.013104534 | 0.017799925 |
| CXCL12 | 38.84815603 | 7.34365892 | -2.403275162 | 4.42E-27 | 6.31E-25 |
| ANKFN1 | 0.002461516 | 0.6990774 | 8.149761171 | 0.001155996 | 0.001841591 |
| SHISA8 | 0.007070272 | 0.052792007 | 2.900481849 | 5.15E-05 | 9.82E-05 |
| TEX19 | 0.003732909 | 0.133133079 | 5.156424947 | 4.96E-20 | 7.81E-19 |
| ZIC5 | 0.003886867 | 0.45970031 | 6.885942024 | 1.55E-19 | 2.20E-18 |
| ASPDH | 60.73992156 | 23.26686543 | -1.384368185 | 7.47E-20 | 1.12E-18 |
| MAP2K3 | 28.85398237 | 13.73692065 | -1.070711813 | 1.14E-21 | 2.50E-20 |
| TTLL4 | 1.108154637 | 3.020171968 | 1.446471482 | 1.70E-11 | 7.30E-11 |
| AP000322.1 | 0.000502161 | 2.63E-05 | -4.257101087 | 0.001048691 | 0.001682015 |
| AC005020.2 | 0.016894037 | 0.04152883 | 1.297599102 | 0.041861936 | 0.05265912 |
| AC007040.2 | 0.020912091 | 0.009073304 | -1.20463745 | 7.52E-06 | 1.59E-05 |
| AGTR1 | 23.74146811 | 9.85355654 | -1.2686927 | 4.60E-21 | 8.87E-20 |
| PTGIR | 1.598350019 | 0.757102844 | -1.078022183 | 2.52E-17 | 2.54E-16 |
| TTC36 | 72.29140751 | 12.4768882 | -2.534566016 | 7.49E-26 | 5.77E-24 |
| TTC39B | 1.533509908 | 0.699018286 | -1.133435386 | 5.75E-20 | 8.92E-19 |
| FOXF2 | 0.049854471 | 0.292551339 | 2.552895022 | 2.32E-09 | 7.51E-09 |
| ATP1A1 | 36.30139029 | 83.40103486 | 1.200040483 | 1.98E-07 | 5.08E-07 |
| KLC2 | 1.166762519 | 2.584244492 | 1.147231621 | 3.16E-20 | 5.18E-19 |
| LRAT | 1.651795592 | 0.234474116 | -2.816534591 | 3.24E-28 | 1.30E-25 |
| NAT14 | 1.425488984 | 3.323911197 | 1.221424949 | 1.35E-06 | 3.13E-06 |
| TRPC7 | 0.000801379 | 0.006699291 | 3.063451626 | 0.031425602 | 0.040276753 |
| HK3 | 2.53018267 | 1.005448314 | -1.331402626 | 2.97E-18 | 3.44E-17 |
| AC011473.4 | 0.005383331 | 0.001094201 | -2.298620704 | 0.011772516 | 0.016093209 |
| CSPG4 | 0.32374069 | 1.447306067 | 2.160459435 | 4.36E-23 | 1.35E-21 |
| LINC00238 | 1.672166816 | 0.43992351 | -1.92639417 | 7.40E-19 | 9.45E-18 |
| MAGEA12 | 0.00932613 | 2.715864902 | 8.185917504 | 0.00018597 | 0.000330217 |
| NAA11 | 0.000504323 | 0.336294701 | 9.381161253 | 0.002901637 | 0.004340914 |
| FHAD1 | 0.013863522 | 0.068467539 | 2.304126307 | 1.45E-12 | 7.19E-12 |
| VN1R1 | 0.104204508 | 0.274407868 | 1.396904161 | 3.38E-06 | 7.46E-06 |
| C1QTNF6 | 1.159154643 | 2.636831098 | 1.185732113 | 1.01E-12 | 5.11E-12 |
| KCNT1 | 0.006542934 | 0.015038845 | 1.20068418 | 0.013104459 | 0.017799925 |
| LTBP2 | 1.387374236 | 3.477389238 | 1.325647564 | 0.000211059 | 0.000371874 |
| CHP2 | 0.006997338 | 0.565538027 | 6.336674094 | 0.006914834 | 0.00977387 |
| GPAT2 | 0.13014655 | 0.434829052 | 1.740311268 | 8.54E-06 | 1.79E-05 |
| KCNC1 | 0.00196225 | 0.03648162 | 4.21658883 | 2.56E-10 | 9.39E-10 |
| SMG9 | 1.229101206 | 2.762767135 | 1.168510252 | 3.96E-24 | 1.64E-22 |
| C1orf204 | 0.043534913 | 0.114343767 | 1.393132968 | 1.64E-06 | 3.74E-06 |
| AL136295.3 | 0.27775401 | 0.118350394 | -1.230743234 | 1.96E-12 | 9.50E-12 |
| ARHGAP11A | 0.206646086 | 1.273848691 | 2.623959977 | 1.92E-25 | 1.29E-23 |
| CDC20B | 0.004760809 | 0.119398358 | 4.648432393 | 1.03E-10 | 3.97E-10 |
| LRRK2 | 0.993307799 | 0.433553677 | -1.19603022 | 8.92E-19 | 1.12E-17 |
| GLYATL3 | 0.198118008 | 0.078140765 | -1.342212744 | 2.05E-14 | 1.30E-13 |
| ZNF581 | 3.181941637 | 7.324167478 | 1.202757406 | 6.25E-18 | 6.92E-17 |
| OR2A5 | 0.00143526 | 0.000340762 | -2.074475935 | 0.00262298 | 0.00395813 |
| SEMA3G | 0.959531365 | 2.092298746 | 1.124686989 | 1.02E-08 | 3.06E-08 |
| TMEM136 | 0.247348204 | 0.671862147 | 1.441621825 | 2.48E-12 | 1.18E-11 |
| GGN | 0.028602627 | 0.08308131 | 1.538376329 | 1.23E-10 | 4.68E-10 |
| IL1RAP | 9.805514146 | 2.597753412 | -1.916328787 | 7.61E-23 | 2.20E-21 |
| TRAF2 | 3.203379903 | 7.253895543 | 1.179161066 | 2.42E-21 | 4.90E-20 |
| FAM183A | 0.077354193 | 0.354365198 | 2.195685529 | 0.001497392 | 0.002341896 |
| POU6F2 | 0.158913981 | 0.046905831 | -1.760406857 | 3.28E-17 | 3.22E-16 |
| ISX | 0.014967724 | 2.140783401 | 7.160140178 | 9.60E-12 | 4.26E-11 |
| NAV3 | 0.103703249 | 0.547978736 | 2.401658816 | 0.001847324 | 0.002841684 |
| WNT8B | 0.009874206 | 0.037123051 | 1.91057867 | 0.001043976 | 0.001674985 |
| OR9Q2 | 0.000309582 | 6.60E-05 | -2.228728617 | 0.000944568 | 0.001524914 |
| AC092042.3 | 0.16027417 | 0.067538378 | -1.246762494 | 1.85E-10 | 6.89E-10 |
| ITGA2 | 0.285993963 | 1.165577323 | 2.026988115 | 1.49E-11 | 6.45E-11 |
| C8orf33 | 6.323061817 | 15.54504421 | 1.29775949 | 1.27E-23 | 4.57E-22 |
| IRAK1 | 11.19241916 | 29.51690518 | 1.399019566 | 7.50E-22 | 1.72E-20 |
| FABP4 | 1.448014627 | 10.42909912 | 2.84846646 | 0.000133296 | 0.000241478 |
| STARD5 | 2.084023856 | 0.766483469 | -1.443045211 | 4.21E-19 | 5.61E-18 |
| OR4F29 | 0.001176424 | 2.63E-05 | -5.485285986 | 0.001589145 | 0.002472887 |
| IL2RB | 3.239580444 | 1.556330889 | -1.057658161 | 9.55E-16 | 7.32E-15 |
| MT1M | 105.7055545 | 26.49524133 | -1.996246015 | 4.41E-24 | 1.80E-22 |
| TLE6 | 0.403805428 | 0.986375122 | 1.28847611 | 0.001558818 | 0.002429102 |
| MCM10 | 0.076892308 | 0.704856411 | 3.196418202 | 3.36E-24 | 1.43E-22 |
| MPP2 | 0.044735046 | 0.115515236 | 1.368605753 | 0.038118911 | 0.048247154 |
| SLN | 0.005082912 | 0.088533079 | 4.122489379 | 0.026058383 | 0.033838271 |
| CELF6 | 0.003310519 | 0.013569036 | 2.035189 | 0.016021591 | 0.021499684 |
| HIST1H2AA | 0.009574827 | 0.057740781 | 2.59227223 | 0.014251311 | 0.019271432 |
| NANOS3 | 0.036615583 | 0.102175239 | 1.48051594 | 0.003402645 | 0.005038389 |
| ECT2 | 0.439148124 | 2.392388585 | 2.445672195 | 2.58E-24 | 1.14E-22 |
| LYG1 | 0.318237685 | 0.749818552 | 1.236436836 | 2.73E-12 | 1.29E-11 |
| ADGRG6 | 14.98622817 | 6.661362309 | -1.169748165 | 7.99E-20 | 1.19E-18 |
| OSR1 | 0.076790537 | 0.25950721 | 1.756774181 | 9.18E-05 | 0.000169559 |
| OR1D2 | 0.001092217 | 4.60E-05 | -4.569738901 | 0.000596625 | 0.000991168 |
| DUSP5 | 18.3650368 | 7.093464566 | -1.372399446 | 4.35E-17 | 4.18E-16 |
| CRYGS | 0.320866216 | 1.692651752 | 2.399241382 | 2.64E-17 | 2.65E-16 |
| PRR21 | 0.000558759 | 0.000230122 | -1.279826808 | 0.001899693 | 0.002918766 |
| POC1A | 2.143951916 | 4.635048115 | 1.112311765 | 1.09E-13 | 6.28E-13 |
| ATOH8 | 7.072848394 | 2.333797315 | -1.599612066 | 3.85E-18 | 4.41E-17 |
| SGCA | 1.318648795 | 0.595885111 | -1.145954267 | 1.60E-15 | 1.18E-14 |
| C1QTNF12 | 0.189536745 | 0.603825787 | 1.671654802 | 7.70E-06 | 1.63E-05 |
| COLEC12 | 0.107325719 | 0.636767863 | 2.568771695 | 1.03E-06 | 2.41E-06 |
| MGAT3 | 0.073639318 | 0.246676232 | 1.744070541 | 1.43E-10 | 5.39E-10 |
| FSCN2 | 0.026156118 | 0.080600627 | 1.623642602 | 3.16E-05 | 6.21E-05 |
| STAB1 | 10.09249108 | 5.033152862 | -1.003747992 | 5.45E-17 | 5.11E-16 |
| EPS8L3 | 0.167006862 | 5.309732497 | 4.990659892 | 2.94E-18 | 3.42E-17 |
| SCIMP | 1.639685485 | 0.5318651 | -1.624286835 | 4.11E-21 | 7.99E-20 |
| ULBP1 | 0.026352011 | 0.0997122 | 1.919856954 | 0.000233605 | 0.000409282 |
| LCN2 | 5.240801629 | 136.2260433 | 4.700071227 | 1.63E-07 | 4.22E-07 |
| PDGFRL | 0.24898028 | 0.755718005 | 1.601816514 | 1.18E-09 | 3.97E-09 |
| RFPL4B | 0.002525336 | 0.574928733 | 7.830764244 | 1.60E-05 | 3.25E-05 |
| BRSK2 | 0.014112021 | 0.163738925 | 3.536400792 | 1.89E-06 | 4.28E-06 |
| DIRC3 | 0.018787798 | 0.039185741 | 1.060532773 | 0.007175648 | 0.010120454 |
| H2AFX | 5.861918421 | 17.0612893 | 1.541281878 | 5.52E-18 | 6.17E-17 |
| NKAIN4 | 0.00694659 | 0.064960153 | 3.225178082 | 0.000499249 | 0.000838034 |
| ELAVL3 | 0.00339743 | 0.011003291 | 1.695419387 | 3.58E-06 | 7.88E-06 |
| TRAPPC3L | 0.066998202 | 0.019889179 | -1.752138665 | 2.68E-17 | 2.69E-16 |
| C8orf44-SGK3 | 0.012527695 | 0.025640313 | 1.0332929 | 0.001709918 | 0.002647318 |
| KIAA1024 | 0.015034973 | 0.046543823 | 1.630267453 | 5.76E-05 | 0.000109276 |
| PRR19 | 0.157772091 | 0.635117825 | 2.00918224 | 1.03E-06 | 2.42E-06 |
| CARD18 | 0.001079473 | 0.153458163 | 7.151374936 | 0.00053841 | 0.000900542 |
| SCNM1 | 3.010496906 | 7.75527026 | 1.365175423 | 2.90E-23 | 9.33E-22 |
| IZUMO1 | 0.01871417 | 0.06841107 | 1.870098778 | 8.66E-12 | 3.86E-11 |
| DMP1 | 0.001342849 | 0.008697961 | 2.695380327 | 0.000990696 | 0.001594486 |
| CDHR4 | 0.007071554 | 0.027303148 | 1.948968172 | 0.000615654 | 0.001020928 |
| FASN | 33.76217555 | 87.33274233 | 1.371114772 | 2.11E-08 | 6.09E-08 |
| FOSB | 30.09081143 | 3.836705961 | -2.971382902 | 1.92E-24 | 8.91E-23 |
| GPR182 | 2.067120252 | 0.201063024 | -3.361902625 | 2.49E-26 | 2.42E-24 |
| ALDH6A1 | 77.98473611 | 28.72247785 | -1.44101156 | 8.76E-23 | 2.48E-21 |
| TMEM151B | 0.000717798 | 0.008650741 | 3.591173101 | 1.31E-14 | 8.55E-14 |
| KRT39 | 0.030664868 | 0.064136498 | 1.064558857 | 0.014253393 | 0.019271432 |
| OXTR | 0.182287916 | 0.589908737 | 1.694272849 | 5.51E-05 | 0.000104707 |
| AL669918.1 | 0.00560403 | 0.016516672 | 1.559386486 | 0.000428787 | 0.000726154 |
| AL049650.1 | 0.02362986 | 0.068465018 | 1.534755964 | 4.92E-06 | 1.06E-05 |
| HIST1H3I | 0.014123898 | 0.062103672 | 2.136540221 | 0.008829931 | 0.012306343 |
| SPRED3 | 0.025772918 | 0.09525178 | 1.885890186 | 3.34E-16 | 2.74E-15 |
| SNURF | 0.062361601 | 0.137578798 | 1.141528288 | 0.000287004 | 0.000497018 |
| AC112484.1 | 0.027427146 | 0.070027867 | 1.352324633 | 1.01E-08 | 3.05E-08 |
| SMCO2 | 0.036711947 | 0.09554192 | 1.379884241 | 0.000301678 | 0.000520922 |
| IQCE | 0.715888274 | 1.610227629 | 1.169458295 | 3.67E-20 | 5.92E-19 |
| ZFP41 | 0.401546069 | 1.530567646 | 1.930429389 | 6.62E-24 | 2.57E-22 |
| P2RY12 | 0.653101216 | 0.118020966 | -2.468263422 | 1.82E-20 | 3.13E-19 |
| FSTL4 | 0.015420089 | 0.406826956 | 4.721532305 | 9.19E-18 | 9.92E-17 |
| CMTM4 | 0.641521484 | 1.575769053 | 1.29648662 | 5.38E-13 | 2.83E-12 |
| C14orf180 | 1.13366873 | 0.156986324 | -2.85228834 | 4.78E-27 | 6.64E-25 |
| PSORS1C1 | 0.029176213 | 0.131402364 | 2.171126674 | 1.93E-11 | 8.22E-11 |
| KLF11 | 13.26847802 | 6.176943208 | -1.103037922 | 7.16E-19 | 9.16E-18 |
| AC009336.2 | 0.000534028 | 0.007485055 | 3.809025142 | 0.010571796 | 0.014565711 |
| KCNE1 | 0.098360289 | 0.038172862 | -1.365528625 | 7.45E-14 | 4.40E-13 |
| LILRA2 | 0.678766976 | 0.178711669 | -1.925282536 | 6.53E-23 | 1.92E-21 |
| CCNB2 | 0.264178854 | 3.661517499 | 3.792854792 | 5.47E-27 | 7.46E-25 |
| PLK3 | 5.535221698 | 1.970887544 | -1.489795641 | 3.74E-09 | 1.18E-08 |
| C2CD4B | 1.490697734 | 0.55691297 | -1.420463959 | 1.78E-14 | 1.14E-13 |
| KIAA1257 | 0.014140494 | 0.0378559 | 1.420685661 | 0.02697954 | 0.034961657 |
| IL37 | 0.003623947 | 0.031609109 | 3.124706557 | 1.60E-05 | 3.25E-05 |
| OSR2 | 0.028039589 | 0.566575759 | 4.336731776 | 3.36E-11 | 1.38E-10 |
| CPA5 | 0.011209411 | 0.068984996 | 2.621572085 | 2.46E-05 | 4.88E-05 |
| MSMB | 0.187371426 | 1.110002675 | 2.56659029 | 0.00456771 | 0.006632895 |
| CD34 | 1.1109034 | 4.737417927 | 2.092367578 | 3.82E-26 | 3.42E-24 |
| CXCR1 | 0.600985698 | 0.130573961 | -2.202463437 | 1.34E-17 | 1.42E-16 |
| OR2AP1 | 0.001092394 | 0.000144713 | -2.916228104 | 0.000401718 | 0.000683868 |
| SPATS2 | 0.817299639 | 2.451947872 | 1.584991305 | 4.68E-28 | 1.38E-25 |
| ESCO2 | 0.085016047 | 0.28887087 | 1.764617644 | 5.07E-14 | 3.07E-13 |
| RHPN1 | 0.492725363 | 1.800524618 | 1.869561684 | 2.85E-14 | 1.79E-13 |
| HIST1H2AE | 2.071944853 | 5.91940801 | 1.514467297 | 0.000222064 | 0.000389985 |
| NKX3-2 | 0.003000722 | 0.083154787 | 4.792417613 | 9.25E-10 | 3.16E-09 |
| FERMT1 | 0.191652463 | 0.945831488 | 2.303090636 | 0.000197661 | 0.000349774 |
| CPT1C | 0.159409084 | 0.339300621 | 1.089830225 | 2.29E-09 | 7.43E-09 |
| ARHGEF2 | 1.504309479 | 3.152144865 | 1.067232438 | 1.99E-09 | 6.50E-09 |
| AZGP1 | 547.6939155 | 264.8461797 | -1.048215155 | 2.96E-18 | 3.44E-17 |
| TUBA3C | 0.014031863 | 2.463531863 | 7.455877784 | 0.005362061 | 0.007709531 |
| GXYLT2 | 0.062572408 | 0.374596099 | 2.581737337 | 6.65E-05 | 0.000124882 |
| IFI27L1 | 2.014945065 | 4.077829011 | 1.017060776 | 1.87E-10 | 7.00E-10 |
| PAGE1 | 0.011467047 | 9.555320942 | 9.702666592 | 0.002036407 | 0.003114364 |
| WDR66 | 0.159770013 | 0.055203899 | -1.533154583 | 1.37E-23 | 4.89E-22 |
| FABP5 | 0.969468103 | 3.258653943 | 1.749010815 | 5.95E-14 | 3.57E-13 |
| ZNF66 | 0.049940774 | 0.117282549 | 1.231698289 | 0.018524475 | 0.024592865 |
| LYZ | 33.99940162 | 226.3908885 | 2.735234635 | 6.80E-05 | 0.000127687 |
| MSH4 | 0.007828905 | 0.044366237 | 2.502579733 | 3.07E-09 | 9.80E-09 |
| B3GNT5 | 0.395261233 | 1.295036414 | 1.712114297 | 5.10E-08 | 1.41E-07 |
| RPL30 | 76.57215833 | 165.9059371 | 1.115473687 | 4.31E-18 | 4.90E-17 |
| HAVCR1 | 0.019208005 | 0.771455422 | 5.327803191 | 3.88E-07 | 9.59E-07 |
| PNCK | 0.012858495 | 0.913901344 | 6.151244679 | 3.46E-08 | 9.72E-08 |
| FAM25G | 0.001020599 | 0.000485997 | -1.070397054 | 0.001672421 | 0.002592877 |
| CLN3 | 3.611117137 | 7.581351424 | 1.070009822 | 1.10E-17 | 1.17E-16 |
| ORAOV1 | 0.643979805 | 2.717366003 | 2.077121544 | 2.41E-15 | 1.73E-14 |
| KITLG | 0.554715153 | 1.228674942 | 1.147284247 | 2.69E-07 | 6.79E-07 |
| MEOX1 | 0.068595951 | 0.216859102 | 1.660562679 | 3.87E-05 | 7.51E-05 |
| EFNA3 | 0.392987757 | 1.651852244 | 2.071528371 | 1.29E-15 | 9.68E-15 |
| CLCN1 | 0.022294521 | 0.107758505 | 2.273040643 | 0.000557161 | 0.000930077 |
| ATXN7L2 | 0.381874379 | 0.770513014 | 1.012721196 | 3.96E-15 | 2.77E-14 |
| JPT1 | 5.589878326 | 13.77468 | 1.301130018 | 8.13E-16 | 6.29E-15 |
| CAMKV | 0.001377939 | 0.053492048 | 5.278740647 | 0.00392659 | 0.005765916 |
| ZSCAN16 | 1.282516523 | 2.802160532 | 1.127562195 | 2.35E-17 | 2.39E-16 |
| NPM3 | 6.970889969 | 14.26381541 | 1.032945177 | 4.47E-08 | 1.24E-07 |
| SORL1 | 10.71923842 | 4.485082909 | -1.256995849 | 1.36E-19 | 1.95E-18 |
| IGLON5 | 0.699424853 | 0.319002101 | -1.132603134 | 1.72E-19 | 2.43E-18 |
| MS4A5 | 0.001422172 | 0.00048446 | -1.553647116 | 0.00077665 | 0.001270971 |
| ACSM4 | 0.011410557 | 0.029749022 | 1.382473056 | 8.46E-05 | 0.000156831 |
| OXT | 6.598254382 | 2.198935716 | -1.58527897 | 1.68E-16 | 1.46E-15 |
| OR10J1 | 0.001313479 | 0.000308656 | -2.089322895 | 0.001799061 | 0.002775113 |
| CCNF | 0.292766206 | 1.360504049 | 2.216320315 | 2.86E-25 | 1.79E-23 |
| CPNE9 | 0.014070753 | 0.046160885 | 1.713971372 | 4.54E-07 | 1.11E-06 |
| IGDCC4 | 0.017882293 | 0.169725772 | 3.246601979 | 3.59E-09 | 1.14E-08 |
| ZP1 | 0.003959806 | 0.023170197 | 2.548768545 | 9.78E-07 | 2.30E-06 |
| DMBX1 | 0.00266142 | 0.091075641 | 5.096797114 | 3.28E-07 | 8.16E-07 |
| LAD1 | 10.42775429 | 24.08605016 | 1.207769333 | 0.020310681 | 0.026779117 |
| MYADML2 | 0.00229058 | 0.02527337 | 3.463833513 | 3.95E-08 | 1.10E-07 |
| CTAGE15 | 0.023723626 | 0.048811932 | 1.04090933 | 0.043178216 | 0.054214082 |
| MCM8 | 0.414534033 | 1.06146082 | 1.356488663 | 3.57E-18 | 4.10E-17 |
| TM4SF19 | 0.034088359 | 0.19822307 | 2.539773846 | 1.45E-07 | 3.79E-07 |
| NOBOX | 0.002595119 | 0.000968259 | -1.422335311 | 0.000305311 | 0.000526961 |
| MEGF10 | 0.089709454 | 0.024221315 | -1.888982834 | 1.21E-18 | 1.48E-17 |
| ATP8A2 | 0.009438111 | 0.075402163 | 2.998035802 | 5.65E-09 | 1.74E-08 |
| PRR7 | 0.688982687 | 1.608810798 | 1.223455034 | 1.76E-07 | 4.54E-07 |
| CTSK | 2.998887806 | 17.13919325 | 2.514799748 | 0.039806016 | 0.050219105 |
| GPR137C | 0.123304834 | 0.30855828 | 1.323313647 | 1.71E-09 | 5.62E-09 |
| USH2A | 1.080596925 | 0.477450247 | -1.178406172 | 2.61E-11 | 1.09E-10 |
| KCNG3 | 0.000588638 | 0.010224418 | 4.118494708 | 0.004978213 | 0.007191368 |
| ATP6V1C2 | 0.218237714 | 0.45193902 | 1.050227684 | 1.13E-14 | 7.45E-14 |
| ABCC4 | 0.530788574 | 1.622905802 | 1.612370043 | 1.34E-06 | 3.10E-06 |
| KCNE5 | 0.067543082 | 0.657233666 | 3.28252647 | 6.40E-11 | 2.53E-10 |
| PEA15 | 16.79144961 | 42.784189 | 1.349350959 | 1.40E-24 | 6.84E-23 |
| KCNH4 | 0.023361739 | 0.130087469 | 2.477262402 | 3.86E-13 | 2.08E-12 |
| ANO4 | 0.013618005 | 0.065186978 | 2.259068402 | 2.92E-09 | 9.33E-09 |
| PRICKLE4 | 0.041910724 | 0.140319955 | 1.743328843 | 1.59E-15 | 1.17E-14 |
| LRRN3 | 0.413153943 | 0.06522947 | -2.663083632 | 1.41E-26 | 1.50E-24 |
| HAUS7 | 0.349030859 | 0.701326572 | 1.006731793 | 0.000805503 | 0.001314317 |
| GLIS2 | 1.263212305 | 2.661882761 | 1.075349901 | 0.004506641 | 0.006551515 |
| PLEKHG2 | 0.716295567 | 1.606702897 | 1.16547626 | 1.06E-17 | 1.13E-16 |
| C9 | 382.7209189 | 83.70183145 | -2.192961663 | 5.73E-24 | 2.26E-22 |
| HES4 | 1.403978123 | 3.344772497 | 1.25238763 | 7.96E-08 | 2.14E-07 |
| POPDC2 | 0.247339995 | 0.516356124 | 1.061870872 | 9.29E-11 | 3.59E-10 |
| PHPT1 | 18.50761408 | 45.43920293 | 1.295818607 | 1.88E-19 | 2.64E-18 |
| ZNF541 | 0.074861153 | 0.409236069 | 2.450644129 | 1.89E-06 | 4.28E-06 |
| CYP26A1 | 9.056403085 | 0.701845278 | -3.689713243 | 6.03E-24 | 2.36E-22 |
| TRIM6 | 0.207635357 | 0.539447938 | 1.377431598 | 6.02E-05 | 0.000113907 |
| TFAP2A | 0.060389104 | 0.247889565 | 2.037337373 | 3.20E-05 | 6.28E-05 |
| AC009690.1 | 0.014167936 | 0.082853353 | 2.54793051 | 4.66E-08 | 1.29E-07 |
| ASB15 | 0.002455052 | 0.052013921 | 4.405072338 | 0.000344304 | 0.000590724 |
| FSD1L | 0.088998775 | 0.218734504 | 1.297323435 | 2.40E-15 | 1.72E-14 |
| FAAP24 | 0.379748035 | 1.083017815 | 1.511942569 | 2.44E-23 | 7.99E-22 |
| MT3 | 0.028934444 | 0.525998188 | 4.184197998 | 0.040282335 | 0.050762399 |
| SLC45A4 | 0.295276131 | 1.237185726 | 2.06692545 | 5.11E-10 | 1.81E-09 |
| TRAIP | 0.160262256 | 1.1909388 | 2.893592683 | 5.89E-27 | 7.76E-25 |
| SULT1A3 | 0.00632764 | 0.017286555 | 1.449911 | 1.74E-06 | 3.97E-06 |
| C6orf222 | 0.010896637 | 0.134279132 | 3.623280242 | 0.006826851 | 0.009659162 |
| DCST2 | 0.146703453 | 0.667891291 | 2.186710472 | 1.02E-14 | 6.80E-14 |
| ARRDC2 | 5.670452667 | 11.86087917 | 1.064675138 | 2.29E-05 | 4.57E-05 |
| AC068580.4 | 0.17834187 | 0.486624354 | 1.448163073 | 2.32E-07 | 5.90E-07 |
| ENAH | 1.672844011 | 5.090983125 | 1.60564136 | 3.46E-20 | 5.60E-19 |
| ZNF492 | 0.006889703 | 0.054594251 | 2.986235274 | 0.000967195 | 0.001559339 |
| BDH2 | 14.82110489 | 6.413970743 | -1.208363324 | 9.80E-22 | 2.18E-20 |
| PITPNM3 | 0.599413138 | 0.101966811 | -2.555451052 | 1.85E-26 | 1.90E-24 |
| SOCS2 | 9.923207239 | 2.399970597 | -2.047789753 | 6.30E-21 | 1.19E-19 |
| FAM83D | 0.931513058 | 4.943584681 | 2.407909647 | 3.61E-22 | 8.90E-21 |
| RNF224 | 0.009685031 | 0.064875488 | 2.743844963 | 3.24E-11 | 1.33E-10 |
| COL4A2 | 8.458145703 | 30.2918491 | 1.840516329 | 2.23E-16 | 1.89E-15 |
| RRM2 | 0.679612577 | 5.895850006 | 3.116915366 | 1.08E-24 | 5.47E-23 |
| HIST1H2BL | 0.024742986 | 0.171963331 | 2.797009449 | 6.28E-10 | 2.20E-09 |
| SLC27A6 | 0.021246155 | 0.004397448 | -2.272463304 | 3.91E-14 | 2.41E-13 |
| RHBDF2 | 1.534162827 | 3.271050529 | 1.092302435 | 3.37E-13 | 1.83E-12 |
| PPM1K | 1.747182633 | 0.756996405 | -1.206672066 | 1.53E-18 | 1.84E-17 |
| CDHR2 | 19.30615155 | 5.780624924 | -1.739763238 | 1.17E-17 | 1.24E-16 |
| SPATA21 | 0.073157786 | 0.49408682 | 2.755681256 | 2.38E-07 | 6.05E-07 |
| SLC39A10 | 0.603260305 | 1.468429977 | 1.283421912 | 9.74E-15 | 6.50E-14 |
| PLEKHS1 | 0.014512318 | 0.223502614 | 3.944941818 | 0.000796617 | 0.001301321 |
| PDGFRB | 4.170137771 | 10.07253779 | 1.272260266 | 9.25E-10 | 3.16E-09 |
| SLC35F4 | 0.001385486 | 0.005178535 | 1.902151964 | 0.029448321 | 0.037935758 |
| ACTG2 | 0.459104481 | 1.74044891 | 1.922565046 | 1.86E-11 | 7.94E-11 |
| GRIN2D | 0.117778239 | 0.252793184 | 1.101884559 | 0.002798759 | 0.004199353 |
| EME2 | 0.449909393 | 1.057957595 | 1.233575409 | 4.62E-18 | 5.23E-17 |
| FRRS1L | 0.006320888 | 0.250388661 | 5.307898164 | 0.000297413 | 0.000513734 |
| CCT8L2 | 0.001395802 | 0.00024295 | -2.522364362 | 0.001527112 | 0.002383747 |
| ANKRD13B | 0.177012812 | 0.615889574 | 1.798817921 | 6.48E-20 | 9.92E-19 |
| DNAJC25 | 15.6640772 | 7.111749011 | -1.139183466 | 1.84E-22 | 4.89E-21 |
| TLDC2 | 0.201681142 | 1.103035558 | 2.451331204 | 3.30E-17 | 3.23E-16 |
| PLA2G4B | 0.023979234 | 0.049068007 | 1.032997117 | 8.49E-05 | 0.000157213 |
| RGS9BP | 0.004358298 | 0.035676459 | 3.033135852 | 1.17E-12 | 5.89E-12 |
| NR1I2 | 13.83636038 | 5.761121007 | -1.26404303 | 1.06E-17 | 1.13E-16 |
| CAVIN3 | 2.194836151 | 5.447554308 | 1.31149543 | 8.41E-07 | 1.99E-06 |
| NMU | 0.023089923 | 0.091205698 | 1.981860594 | 0.000901573 | 0.001458984 |
| LRRC1 | 0.412980777 | 2.165591416 | 2.390614538 | 7.11E-14 | 4.21E-13 |
| CORIN | 0.024705227 | 0.129950827 | 2.395077566 | 0.01962395 | 0.025972772 |
| RFX5 | 3.253385085 | 7.550152991 | 1.214564282 | 1.00E-19 | 1.47E-18 |
| TMEM132A | 0.352141371 | 2.259776981 | 2.681953765 | 5.55E-19 | 7.24E-18 |
| SLC22A13 | 0.006989018 | 0.014900698 | 1.092218319 | 0.036382822 | 0.046173745 |
| ARHGAP33 | 0.373995172 | 1.134875948 | 1.601443056 | 4.00E-22 | 9.71E-21 |
| SPHKAP | 0.001280788 | 0.000371592 | -1.78524086 | 7.50E-05 | 0.000140094 |
| PAEP | 0.01417336 | 11.9473324 | 9.719291034 | 3.16E-10 | 1.15E-09 |
| C5orf67 | 0.01303236 | 0.047634626 | 1.86991236 | 0.002143956 | 0.003270955 |
| BRICD5 | 0.380170382 | 1.028446773 | 1.435749083 | 1.84E-15 | 1.35E-14 |
| OPN1MW | 0.000759016 | 2.63E-05 | -4.853080555 | 0.000133876 | 0.000242472 |
| PTPRN | 0.002835214 | 0.016767941 | 2.564175927 | 9.53E-08 | 2.54E-07 |
| NAT1 | 3.31717208 | 1.390019126 | -1.25484912 | 2.47E-22 | 6.34E-21 |
| E2F1 | 0.656916778 | 8.474095497 | 3.689276868 | 3.72E-26 | 3.37E-24 |
| SLC35F1 | 0.023046398 | 0.073153821 | 1.666391922 | 0.004091037 | 0.005983676 |
| ALS2CL | 1.298961981 | 3.545740429 | 1.44872772 | 2.87E-11 | 1.19E-10 |
| CBR3 | 0.360618094 | 1.38536012 | 1.94171736 | 1.87E-09 | 6.14E-09 |
| LCE2B | 0.002743051 | 0.000767388 | -1.837753472 | 0.004216514 | 0.006153896 |
| TDRKH | 0.953691766 | 2.221000723 | 1.219614896 | 1.18E-14 | 7.79E-14 |
| KLHL15 | 4.251089425 | 1.73673374 | -1.291456017 | 1.24E-23 | 4.44E-22 |
| ADAMTS10 | 0.936976381 | 1.88810329 | 1.010853105 | 2.10E-05 | 4.20E-05 |
| CYP19A1 | 0.003266461 | 0.359040541 | 6.780274483 | 1.28E-08 | 3.78E-08 |
| MCM4 | 2.19556828 | 7.015449414 | 1.675941125 | 6.26E-21 | 1.18E-19 |
| RGMA | 0.020854058 | 0.083580131 | 2.002831849 | 6.81E-17 | 6.28E-16 |
| DEPDC7 | 14.98283212 | 6.776355539 | -1.144728877 | 1.81E-18 | 2.17E-17 |
| AGO2 | 0.873283426 | 1.776841452 | 1.02479309 | 2.76E-15 | 1.97E-14 |
| CDCA7 | 0.075427178 | 1.13191817 | 3.907541409 | 7.43E-16 | 5.78E-15 |
| EDA2R | 0.200906341 | 0.471522925 | 1.230804814 | 0.012572301 | 0.017115844 |
| HES2 | 0.017107561 | 0.157789582 | 3.205295982 | 2.94E-11 | 1.22E-10 |
| AC073896.1 | 0.008161296 | 0.016405609 | 1.007318976 | 0.04004267 | 0.050494374 |
| WISP2 | 1.154983669 | 0.412273089 | -1.486200256 | 4.03E-10 | 1.45E-09 |
| CIART | 1.008171699 | 2.823219505 | 1.485599941 | 3.88E-10 | 1.39E-09 |
| RTL5 | 2.067964195 | 1.003067095 | -1.043793096 | 9.46E-15 | 6.32E-14 |
| TBX15 | 12.78265563 | 4.054599091 | -1.656556415 | 2.32E-17 | 2.36E-16 |
| CABYR | 0.099527038 | 1.981757576 | 4.315548167 | 4.96E-13 | 2.63E-12 |
| RASD2 | 0.065124596 | 0.434875217 | 2.739327063 | 5.66E-26 | 4.58E-24 |
| DGKI | 0.007993978 | 0.037803877 | 2.24154869 | 2.47E-21 | 4.99E-20 |
| ANGPT2 | 0.512758097 | 1.096452788 | 1.09649342 | 1.45E-08 | 4.25E-08 |
| KIF4A | 0.155853414 | 2.490053704 | 3.997915194 | 1.45E-27 | 2.79E-25 |
| AANAT | 0.013950658 | 0.05246822 | 1.911110649 | 1.50E-07 | 3.91E-07 |
| JDP2 | 3.463313064 | 1.580276197 | -1.131976073 | 5.76E-22 | 1.36E-20 |
| USP49 | 0.159947962 | 0.412883495 | 1.36813214 | 7.39E-25 | 3.98E-23 |
| HPX | 1059.04677 | 529.1739515 | -1.000952352 | 1.84E-19 | 2.59E-18 |
| GCDH | 32.21805318 | 14.602734 | -1.141630817 | 9.22E-19 | 1.15E-17 |
| ADGRG4 | 0.005963568 | 0.001203873 | -2.308492373 | 0.003030964 | 0.004519431 |
| GPR142 | 0.018542221 | 0.00892216 | -1.055349135 | 0.000807184 | 0.001316923 |
| COL22A1 | 0.026922324 | 0.697680727 | 4.695692117 | 5.90E-06 | 1.26E-05 |
| NDST3 | 0.2395547 | 0.011948397 | -4.325466078 | 1.28E-28 | 8.00E-26 |
| DAND5 | 0.010384282 | 0.087578354 | 3.076172892 | 1.16E-14 | 7.69E-14 |
| ZNF285 | 0.084602817 | 0.195359437 | 1.207353344 | 0.012334792 | 0.016816044 |
| CSPG5 | 0.057650888 | 0.359697552 | 2.641369617 | 1.39E-17 | 1.47E-16 |
| KRTAP5-1 | 0.022787357 | 0.136775892 | 2.585508431 | 3.63E-09 | 1.15E-08 |
| PLCD3 | 0.398328034 | 1.070422095 | 1.426150877 | 1.56E-09 | 5.16E-09 |
| ZP4 | 0.002082408 | 0.000767718 | -1.439604886 | 0.002922204 | 0.004370244 |
| GAREM1 | 3.212753831 | 1.35985859 | -1.240353807 | 2.28E-13 | 1.26E-12 |
| NR4A1 | 12.84292271 | 3.260955949 | -1.977606702 | 1.19E-19 | 1.72E-18 |
| GABRR1 | 0.00106927 | 0.012131192 | 3.504023852 | 0.001909746 | 0.002932601 |
| ABCC10 | 0.668536689 | 1.734246419 | 1.375230263 | 1.34E-27 | 2.65E-25 |
| AC104581.1 | 0.228357262 | 0.472839218 | 1.050057028 | 9.59E-09 | 2.89E-08 |
| SYT2 | 0.017356923 | 0.080438903 | 2.212382191 | 0.000899605 | 0.001456107 |
| CYP17A1 | 1.233486919 | 22.8169032 | 4.209288676 | 0.008471687 | 0.011833589 |
| PPBP | 0.915614892 | 0.250221875 | -1.871533008 | 5.81E-20 | 9.01E-19 |
| SIGLEC11 | 1.077725623 | 0.227466892 | -2.244261449 | 3.37E-23 | 1.07E-21 |
| TEDDM1 | 0.001420554 | 0.040316442 | 4.826842637 | 0.002682645 | 0.004041388 |
| TP53TG3 | 0.000485383 | 0.000185722 | -1.385980771 | 0.009631792 | 0.013350981 |
| SPIC | 0.505497544 | 0.14719226 | -1.780002268 | 2.45E-17 | 2.48E-16 |
| RNF144A | 0.429514217 | 0.965642599 | 1.168783437 | 1.44E-07 | 3.76E-07 |
| G6PD | 1.924250026 | 12.58937626 | 2.709838634 | 1.32E-21 | 2.84E-20 |
| NAALADL1 | 0.342737396 | 1.766479108 | 2.365701169 | 0.007109901 | 0.010035003 |
| FOXL1 | 0.062251375 | 0.210333014 | 1.756497717 | 3.86E-07 | 9.56E-07 |
| PGA3 | 0.002879412 | 0.000637431 | -2.175433226 | 4.94E-05 | 9.45E-05 |
| CYP4A11 | 244.9410581 | 72.8218655 | -1.749991021 | 3.54E-25 | 2.08E-23 |
| TUBG1 | 6.690983379 | 14.8653635 | 1.151664576 | 2.80E-20 | 4.66E-19 |
| DHDH | 0.124899616 | 0.50999153 | 2.029704247 | 1.25E-06 | 2.90E-06 |
| ARL2 | 4.631357621 | 9.490707682 | 1.035080505 | 3.49E-09 | 1.10E-08 |
| E2F5 | 0.40833142 | 0.934894654 | 1.195063225 | 1.06E-11 | 4.66E-11 |
| IRX3 | 0.326392956 | 1.791506118 | 2.456491142 | 2.59E-05 | 5.13E-05 |
| CCDC192 | 0.028356781 | 0.093689706 | 1.724196778 | 8.87E-07 | 2.10E-06 |
| PRR11 | 0.254674069 | 1.499959302 | 2.55819938 | 1.67E-22 | 4.46E-21 |
| ZNF71 | 0.309403069 | 0.687868554 | 1.152645398 | 6.65E-13 | 3.45E-12 |
| PLPPR4 | 0.016727719 | 0.107874708 | 2.689044016 | 2.35E-12 | 1.13E-11 |
| DIAPH3 | 0.042040737 | 0.594677226 | 3.822246972 | 2.68E-26 | 2.56E-24 |
| CD200 | 0.284463778 | 1.103631113 | 1.955941168 | 3.39E-21 | 6.70E-20 |
| RRAGD | 3.050689537 | 6.178330215 | 1.018081614 | 1.38E-08 | 4.07E-08 |
| RGS18 | 0.714943804 | 0.301444996 | -1.245935065 | 1.40E-14 | 9.11E-14 |
| GABRE | 0.268864235 | 2.059316408 | 2.937215753 | 1.03E-14 | 6.86E-14 |
| ZNF789 | 0.402701197 | 0.843461619 | 1.06661266 | 5.62E-20 | 8.74E-19 |
| PROCA1 | 0.101851258 | 0.328420241 | 1.689079251 | 1.26E-17 | 1.33E-16 |
| KIF19 | 0.339291683 | 0.064872551 | -2.386846 | 5.06E-26 | 4.23E-24 |
| KMT5C | 0.64519862 | 1.819653702 | 1.495848659 | 5.88E-24 | 2.31E-22 |
| RPL36A-HNRNPH2 | 0.019479169 | 0.0558952 | 1.520792254 | 0.001419127 | 0.002227031 |
| PEX11G | 10.69736736 | 5.151715147 | -1.054131061 | 3.06E-17 | 3.03E-16 |
| ETV4 | 0.761701659 | 5.947990714 | 2.965104451 | 1.42E-06 | 3.27E-06 |
| KISS1 | 0.985345798 | 3.102099928 | 1.654543141 | 5.57E-07 | 1.35E-06 |
| RELL2 | 0.192462804 | 0.513896534 | 1.416898269 | 1.08E-14 | 7.19E-14 |
| ARHGEF11 | 2.62682827 | 5.973080134 | 1.18515319 | 7.16E-21 | 1.34E-19 |
| ELOVL3 | 0.0404455 | 0.237689466 | 2.555026856 | 0.00022368 | 0.000392689 |
| POLN | 0.10528383 | 0.263412816 | 1.323041662 | 0.000592406 | 0.000984794 |
| RAB24 | 2.024376297 | 4.238075368 | 1.065931757 | 2.36E-20 | 3.96E-19 |
| RNF151 | 0.007228928 | 0.072605976 | 3.328234637 | 7.29E-06 | 1.54E-05 |
| CTXN1 | 0.137402943 | 0.75205816 | 2.452431335 | 4.63E-10 | 1.65E-09 |
| PAIP2B | 5.571578389 | 2.231958162 | -1.319776107 | 5.61E-19 | 7.31E-18 |
| MELK | 0.149476831 | 2.266451041 | 3.922441211 | 8.45E-28 | 1.91E-25 |
| CD109 | 0.246194156 | 1.397213222 | 2.504683778 | 1.29E-13 | 7.38E-13 |
| SHC3 | 0.032586442 | 0.122582209 | 1.911405865 | 2.87E-07 | 7.19E-07 |
| HIST1H4I | 5.287012823 | 10.89119785 | 1.042637905 | 2.64E-08 | 7.53E-08 |
| SPATC1 | 0.057162381 | 0.195114882 | 1.771185909 | 6.54E-14 | 3.91E-13 |
| ADRA1A | 7.868443908 | 0.959452974 | -3.03579435 | 2.23E-26 | 2.19E-24 |
| HOPX | 0.161653501 | 0.403723017 | 1.320461085 | 9.46E-06 | 1.98E-05 |
| SCRIB | 6.065700015 | 15.55890726 | 1.358994685 | 1.69E-22 | 4.50E-21 |
| KLF4 | 6.208375677 | 2.945330386 | -1.075786385 | 2.88E-14 | 1.80E-13 |
| LHX2 | 1.353594834 | 0.457265464 | -1.565692103 | 1.07E-24 | 5.42E-23 |
| TTC13 | 1.379478498 | 3.687450483 | 1.418500707 | 1.71E-27 | 3.20E-25 |
| CLDN18 | 0.009684189 | 0.202063719 | 4.383035266 | 8.17E-05 | 0.000151746 |
| H2AFZ | 17.11979905 | 44.26826687 | 1.370607125 | 1.73E-21 | 3.63E-20 |
| ACSBG2 | 0.004404053 | 0.02180556 | 2.307792433 | 2.00E-10 | 7.44E-10 |
| KRTAP13-3 | 0.001143851 | 2.63E-05 | -5.444777709 | 0.00029732 | 0.000513734 |
| ELOA3C | 0.00054787 | 0.000202634 | -1.434956081 | 0.001411396 | 0.002216016 |
| LVRN | 0.005759639 | 0.032514455 | 2.497031068 | 1.84E-06 | 4.18E-06 |
| HTR1B | 0.007426034 | 0.044249003 | 2.574981182 | 5.87E-05 | 0.000111127 |
| RPS5 | 62.17569294 | 130.9187165 | 1.074248778 | 1.82E-17 | 1.89E-16 |
| DLX5 | 0.004364186 | 0.243825664 | 5.803993438 | 7.57E-13 | 3.90E-12 |
| ATP6V0D2 | 0.046366733 | 0.427036591 | 3.203197701 | 8.88E-11 | 3.44E-10 |
| MYT1 | 0.025799201 | 0.075570518 | 1.550497091 | 0.000189178 | 0.000335717 |
| MDK | 5.365076297 | 79.43500783 | 3.888104365 | 7.85E-23 | 2.25E-21 |
| HMGB2 | 6.018206011 | 17.08084112 | 1.504973622 | 2.66E-18 | 3.12E-17 |
| RIPOR3 | 1.864691791 | 0.501696311 | -1.894050955 | 6.86E-27 | 8.73E-25 |
| SBF1 | 2.504160034 | 5.205563185 | 1.055727492 | 3.68E-17 | 3.58E-16 |
| IHH | 1.341663878 | 3.166450761 | 1.238843362 | 0.003336015 | 0.004946318 |
| CFAP53 | 0.130581607 | 0.371547844 | 1.508596301 | 7.46E-12 | 3.35E-11 |
| MEP1A | 0.028272968 | 3.316034274 | 6.873891767 | 5.30E-08 | 1.46E-07 |
| SYCP1 | 0.004929393 | 0.001227655 | -2.005505277 | 2.23E-08 | 6.42E-08 |
| PLXNC1 | 0.461954 | 1.72639147 | 1.901938537 | 5.05E-13 | 2.67E-12 |
| RUFY4 | 0.035811332 | 0.093455585 | 1.383864726 | 1.49E-05 | 3.04E-05 |
| FAM19A3 | 0.009987768 | 0.02449195 | 1.294073485 | 0.0011411 | 0.001819543 |
| SHISA2 | 0.024587683 | 0.136508926 | 2.47298761 | 7.29E-08 | 1.97E-07 |
| MBD3L5 | 0.000438438 | 8.87E-05 | -2.305534296 | 0.000768808 | 0.0012588 |
| P2RX3 | 0.440392808 | 0.185870595 | -1.244492359 | 1.09E-12 | 5.51E-12 |
| HSD17B3 | 0.553858687 | 1.313179254 | 1.245474028 | 0.000607357 | 0.001007814 |
| GYS2 | 48.40415282 | 12.95170666 | -1.901988613 | 4.19E-24 | 1.72E-22 |
| SYN3 | 0.022314178 | 0.142762108 | 2.677580558 | 4.10E-11 | 1.67E-10 |
| CACNA1B | 0.002085103 | 0.011980945 | 2.522550833 | 0.019495325 | 0.025809076 |
| RPLP2 | 135.8058067 | 309.0282802 | 1.186193703 | 5.25E-18 | 5.89E-17 |
| DIRAS3 | 4.289802196 | 0.638323573 | -2.748551293 | 3.68E-25 | 2.13E-23 |
| MT1HL1 | 0.331813556 | 0.051524493 | -2.687042507 | 1.94E-15 | 1.42E-14 |
| CPA2 | 0.005466923 | 3.014356096 | 9.106905071 | 0.014973903 | 0.020185771 |
| DLGAP5 | 0.117439075 | 1.660463754 | 3.821601814 | 3.00E-27 | 4.86E-25 |
| LRFN2 | 0.01104019 | 0.060347124 | 2.450519948 | 0.000937929 | 0.001514824 |
| DIRAS1 | 0.036838371 | 0.453496399 | 3.621809914 | 3.46E-06 | 7.61E-06 |
| PQLC2L | 0.007392143 | 0.089312144 | 3.594791746 | 0.021676705 | 0.02845239 |
| ONECUT3 | 0.003011053 | 0.02863019 | 3.249197374 | 0.004327528 | 0.006304799 |
| ORC1 | 0.154019559 | 1.263051222 | 3.035727668 | 2.34E-25 | 1.52E-23 |
| HBB | 49.20363626 | 13.31315334 | -1.885912611 | 1.84E-20 | 3.16E-19 |
| SLC27A5 | 113.5838261 | 48.3612581 | -1.231833734 | 1.05E-17 | 1.12E-16 |
| TIGD3 | 0.102594346 | 0.498690548 | 2.281193635 | 1.83E-15 | 1.35E-14 |
| ATP1A4 | 0.005297341 | 0.024160413 | 2.189304886 | 0.004541438 | 0.006599019 |
| AC110611.1 | 0.016943684 | 0.005061717 | -1.743048896 | 3.98E-06 | 8.69E-06 |
| SH3D21 | 0.168738626 | 0.419142126 | 1.312649265 | 1.18E-13 | 6.79E-13 |
| IFITM5 | 0.024715846 | 0.142121431 | 2.523615929 | 0.027840351 | 0.036010989 |
| TMPRSS5 | 0.062504219 | 0.181199498 | 1.535553482 | 1.26E-08 | 3.74E-08 |
| F13A1 | 0.665641145 | 2.470329708 | 1.891887089 | 0.000481262 | 0.000809895 |
| PDIA2 | 0.021699367 | 1.553348568 | 6.161584822 | 7.30E-13 | 3.78E-12 |
| SLC39A14 | 101.3241362 | 43.76077502 | -1.211267682 | 1.26E-19 | 1.81E-18 |
| SCGB3A1 | 1.879184576 | 0.456364094 | -2.041849585 | 1.00E-13 | 5.80E-13 |
| GCNT3 | 0.193818078 | 1.265380095 | 2.70679576 | 5.39E-08 | 1.48E-07 |
| SLC7A10 | 0.01165224 | 0.912514744 | 6.291168645 | 3.97E-05 | 7.70E-05 |
| RPS6KA6 | 0.145715119 | 0.059920764 | -1.282022665 | 3.12E-18 | 3.59E-17 |
| ADAMTS16 | 0.046034446 | 0.64634795 | 3.811525321 | 0.001233204 | 0.001953786 |
| USP17L10 | 0.000252501 | 2.63E-05 | -3.26524054 | 0.000689038 | 0.001134999 |
| CDKL2 | 0.022630971 | 0.010370291 | -1.125842056 | 6.16E-16 | 4.85E-15 |
| IFNW1 | 0.00220047 | 0.000814242 | -1.434282858 | 0.005425464 | 0.007795471 |
| B3GNT4 | 0.032717337 | 0.095071028 | 1.538950425 | 2.91E-10 | 1.06E-09 |
| BRINP3 | 0.001139893 | 0.130148335 | 6.835114897 | 4.76E-05 | 9.11E-05 |
| PSORS1C2 | 0.005055341 | 0.020604013 | 2.027044995 | 0.035296558 | 0.044872026 |
| XRCC2 | 0.061041049 | 0.507074937 | 3.054347302 | 1.30E-25 | 9.24E-24 |
| UCHL1 | 0.229793774 | 6.862163344 | 4.900251853 | 2.77E-05 | 5.47E-05 |
| ABCC5 | 0.633781064 | 1.378479563 | 1.121021418 | 1.77E-23 | 6.04E-22 |
| KLHL38 | 0.010667898 | 0.091595781 | 3.10200527 | 2.59E-09 | 8.32E-09 |
| NT5M | 0.642745195 | 1.550718009 | 1.270617539 | 2.78E-07 | 7.00E-07 |
| COBLL1 | 8.602372581 | 4.24915854 | -1.017557445 | 9.95E-17 | 8.92E-16 |
| MSH5-SAPCD1 | 0.06132675 | 0.239468997 | 1.965250494 | 2.95E-18 | 3.43E-17 |
| CDRT1 | 0.010830613 | 0.109870421 | 3.342616268 | 1.93E-05 | 3.88E-05 |
| AC008878.3 | 0.00444322 | 0.001562069 | -1.508147171 | 5.91E-05 | 0.000111932 |
| KISS1R | 0.010925753 | 0.241121163 | 4.463953669 | 4.16E-06 | 9.07E-06 |
| PLIN2 | 139.2770087 | 64.33407297 | -1.11430219 | 1.40E-15 | 1.05E-14 |
| ANKRD45 | 0.032562622 | 0.117565561 | 1.852176725 | 4.62E-06 | 1.00E-05 |
| HOXD1 | 0.001261518 | 0.118451804 | 6.552996051 | 1.41E-14 | 9.15E-14 |
| DNAJC12 | 16.76009049 | 6.876183832 | -1.285349918 | 1.97E-16 | 1.69E-15 |
| CCDC112 | 0.353400359 | 0.747086864 | 1.079972492 | 6.56E-09 | 2.02E-08 |
| PRTFDC1 | 0.508209477 | 1.213362709 | 1.255515695 | 1.16E-07 | 3.07E-07 |
| OR2V1 | 0.00680645 | 0.000975781 | -2.802273678 | 4.05E-05 | 7.85E-05 |
| ARMS2 | 0.024123485 | 0.072422098 | 1.585991616 | 0.000576488 | 0.000960395 |
| S100A1 | 0.568775345 | 3.418966636 | 2.58762951 | 0.000217283 | 0.000382062 |
| PPFIA4 | 0.013599055 | 0.084580906 | 2.636825611 | 2.99E-06 | 6.62E-06 |
| GPR158 | 0.040749154 | 0.440443751 | 3.434115775 | 8.94E-10 | 3.05E-09 |
| FBXL6 | 4.058240119 | 8.762594725 | 1.110503906 | 7.64E-13 | 3.94E-12 |
| MESP1 | 0.321238393 | 1.243656113 | 1.952871384 | 1.82E-16 | 1.57E-15 |
| EME1 | 0.072361869 | 0.738390152 | 3.351081727 | 3.66E-27 | 5.59E-25 |
| SLC22A15 | 0.193444127 | 0.673140432 | 1.798990587 | 4.45E-05 | 8.56E-05 |
| FAM155B | 0.064404432 | 0.424965325 | 2.722113257 | 1.81E-05 | 3.65E-05 |
| SPIRE2 | 0.927330866 | 2.233559669 | 1.268188715 | 1.84E-14 | 1.18E-13 |
| CBX8 | 1.180807697 | 2.704100936 | 1.195374974 | 6.77E-18 | 7.45E-17 |
| NCOR1 | 7.044843893 | 3.36635336 | -1.065381116 | 1.04E-22 | 2.89E-21 |
| GPAA1 | 23.48212224 | 67.48556422 | 1.523016129 | 1.12E-25 | 8.07E-24 |
| ATP6V1C1 | 6.028303494 | 12.43498978 | 1.044581366 | 3.56E-17 | 3.48E-16 |
| HCRT | 0.005788622 | 0.051320252 | 3.148236455 | 0.030559611 | 0.039273702 |
| AIFM3 | 0.166567379 | 0.418331604 | 1.328541111 | 2.14E-06 | 4.83E-06 |
| CYP2C19 | 4.586449874 | 0.391669836 | -3.549667942 | 1.86E-19 | 2.62E-18 |
| AC099329.3 | 0.019963984 | 0.042984784 | 1.106426424 | 0.010666393 | 0.014684284 |
| TSEN54 | 3.688891858 | 7.705018404 | 1.062610907 | 5.91E-21 | 1.12E-19 |
| SLC16A3 | 0.857736217 | 2.819151664 | 1.716655151 | 9.83E-05 | 0.000180885 |
| DEFB135 | 0.001738229 | 0.000719106 | -1.273341578 | 0.002038362 | 0.003117047 |
| APOF | 192.0845276 | 34.3660424 | -2.482685689 | 2.59E-27 | 4.28E-25 |
| PLCD4 | 0.189021445 | 0.410613507 | 1.119231161 | 8.34E-12 | 3.72E-11 |
| WNT2 | 0.5248215 | 0.141465878 | -1.891372708 | 4.88E-20 | 7.69E-19 |
| TEKT5 | 0.099057327 | 0.306536517 | 1.629723344 | 0.000120921 | 0.000220163 |
| NOL4 | 0.256611393 | 0.066752584 | -1.94268963 | 3.16E-15 | 2.23E-14 |
| PALM2-AKAP2 | 0.137689535 | 0.051635761 | -1.414976445 | 4.82E-12 | 2.22E-11 |
| UTS2 | 0.057767146 | 0.213816282 | 1.888050598 | 0.043243574 | 0.054291763 |
| COX6B1 | 128.3088725 | 260.4174163 | 1.021205001 | 3.58E-17 | 3.49E-16 |
| ASPH | 4.793876746 | 9.724667049 | 1.020456039 | 0.001575353 | 0.002452896 |
| TNNT1 | 0.054562901 | 0.672466583 | 3.623470328 | 0.031235591 | 0.040046422 |
| SLC6A12 | 18.56524263 | 9.001392975 | -1.044383987 | 4.26E-15 | 2.96E-14 |
| PWWP2B | 2.649046415 | 5.471120018 | 1.046363082 | 4.50E-08 | 1.25E-07 |
| GPR161 | 0.110169136 | 0.232023553 | 1.07455115 | 4.47E-09 | 1.40E-08 |
| IL31RA | 0.006611737 | 0.039832565 | 2.59084719 | 0.021995003 | 0.028848276 |
| VSIG8 | 0.009726839 | 0.025250482 | 1.376268053 | 0.001095094 | 0.001751926 |
| RNF183 | 0.009188201 | 0.095653676 | 3.379966122 | 4.80E-07 | 1.17E-06 |
| UPK3A | 0.171717963 | 9.174654308 | 5.739540928 | 0.000113854 | 0.000208026 |
| EPHX4 | 0.021054164 | 0.13853346 | 2.718056971 | 6.73E-19 | 8.65E-18 |
| SLC1A3 | 0.520615903 | 1.21657682 | 1.224536134 | 1.34E-07 | 3.50E-07 |
| F9 | 185.1245145 | 67.40654943 | -1.457535272 | 7.37E-21 | 1.37E-19 |
| RNF17 | 0.001770911 | 0.09948857 | 5.811967078 | 0.002117803 | 0.003233482 |
| SRD5A2 | 15.90489695 | 4.390894201 | -1.856884346 | 7.46E-21 | 1.38E-19 |
| ZIM2 | 0.004916999 | 0.02913974 | 2.567137951 | 0.021362358 | 0.028063816 |
| FOXS1 | 0.259413801 | 1.947829383 | 2.908540171 | 1.86E-21 | 3.86E-20 |
| ARMC12 | 0.193198272 | 0.416634334 | 1.108699544 | 3.82E-05 | 7.41E-05 |
| LZTS1 | 0.327797027 | 0.892248714 | 1.444643149 | 2.55E-14 | 1.61E-13 |
| SSC4D | 0.831978516 | 2.386703586 | 1.520401224 | 0.000267836 | 0.000465794 |
| CPA6 | 0.004698088 | 0.345976571 | 6.202456731 | 1.84E-14 | 1.18E-13 |
| RTN2 | 1.105115733 | 2.668170963 | 1.271653646 | 7.56E-05 | 0.000141128 |
| AURKA | 1.004713999 | 6.451276048 | 2.682799667 | 6.62E-26 | 5.17E-24 |
| MMP9 | 1.946944584 | 9.542302802 | 2.293125646 | 3.43E-05 | 6.71E-05 |
| JUN | 84.92544306 | 39.50742602 | -1.104072985 | 4.18E-14 | 2.56E-13 |
| BMP10 | 0.985572104 | 0.028258289 | -5.124215426 | 9.70E-28 | 2.10E-25 |
| HAMP | 156.4705286 | 15.07132506 | -3.376012781 | 1.11E-25 | 8.01E-24 |
| PIEZO2 | 0.855207256 | 1.881023591 | 1.137171945 | 3.68E-09 | 1.16E-08 |
| CD300A | 4.361262773 | 1.891587746 | -1.205148217 | 4.81E-16 | 3.85E-15 |
| OAS2 | 11.16004193 | 5.40919647 | -1.044856244 | 0.001227911 | 0.001946194 |
| ORC6 | 0.095296138 | 0.763087498 | 3.001358833 | 1.50E-26 | 1.58E-24 |
| ATP11C | 7.737975112 | 3.318143127 | -1.221579971 | 2.82E-19 | 3.86E-18 |
| ZNF219 | 1.702416062 | 4.157482655 | 1.288126577 | 4.04E-20 | 6.45E-19 |
| STATH | 0.00141478 | 0.000706271 | -1.002282809 | 0.003573714 | 0.005276128 |
| DISP3 | 0.007293546 | 0.038944527 | 2.416728383 | 0.002688222 | 0.004047583 |
| BMP5 | 0.887851604 | 0.148296163 | -2.58183729 | 9.19E-27 | 1.07E-24 |
| SLC39A7 | 33.10884895 | 69.73758441 | 1.074719537 | 6.52E-21 | 1.23E-19 |
| CYB5RL | 0.185062037 | 0.370625906 | 1.001954748 | 5.62E-13 | 2.96E-12 |
| PODNL1 | 0.040162727 | 0.145049815 | 1.85261933 | 8.58E-13 | 4.40E-12 |
| MRC1 | 11.21137259 | 5.290650021 | -1.083446025 | 3.47E-17 | 3.40E-16 |
| ENOX1 | 0.080020765 | 0.303773421 | 1.924549317 | 7.63E-06 | 1.61E-05 |
| MARCO | 44.50210757 | 1.839346179 | -4.596608725 | 7.89E-29 | 6.01E-26 |
| APC2 | 0.111677503 | 0.248911793 | 1.156296 | 2.64E-11 | 1.10E-10 |
| ZP3 | 0.26243363 | 0.831037281 | 1.662960589 | 2.21E-10 | 8.17E-10 |
| FAP | 0.060307774 | 0.347222233 | 2.525443448 | 8.43E-13 | 4.32E-12 |
| HJURP | 0.132738235 | 2.130517705 | 4.004548142 | 1.29E-27 | 2.60E-25 |
| OTOG | 0.001126705 | 0.085882017 | 6.252174743 | 0.003168177 | 0.004708233 |
| IQGAP3 | 0.229991345 | 2.60952603 | 3.504136319 | 1.35E-25 | 9.57E-24 |
| GRM8 | 0.459817436 | 0.168244974 | -1.450497768 | 1.12E-15 | 8.51E-15 |
| CA8 | 0.019364519 | 0.101975103 | 2.396729394 | 1.62E-09 | 5.37E-09 |
| STRC | 0.002808282 | 0.616749047 | 7.778851805 | 2.44E-09 | 7.87E-09 |
| PNMA3 | 0.117350253 | 1.650141785 | 3.813697134 | 1.74E-05 | 3.51E-05 |
| ARHGEF37 | 0.634621701 | 1.73808287 | 1.453528111 | 2.36E-07 | 5.99E-07 |
| TFPI2 | 3.178402013 | 0.876382687 | -1.858668724 | 2.03E-21 | 4.19E-20 |
| TMEM45A | 12.65931845 | 6.295640177 | -1.007774743 | 1.34E-17 | 1.42E-16 |
| RPS6KL1 | 0.174127317 | 0.744894362 | 2.096893292 | 2.46E-15 | 1.76E-14 |
| PELI2 | 0.816911717 | 0.287430052 | -1.506969268 | 6.65E-23 | 1.96E-21 |
| TCTEX1D1 | 1.436917473 | 0.343322071 | -2.065342694 | 2.86E-19 | 3.93E-18 |
| ALDH8A1 | 80.392416 | 26.50124039 | -1.600999522 | 1.94E-26 | 1.95E-24 |
| KIAA1549 | 0.150562403 | 0.440046348 | 1.547293924 | 0.002787528 | 0.004184116 |
| N4BP3 | 0.076488556 | 0.328438858 | 2.102309009 | 1.15E-20 | 2.03E-19 |
| SPP1 | 13.92180287 | 478.7171096 | 5.103755413 | 2.40E-05 | 4.77E-05 |
| CLRN3 | 23.65573104 | 9.14179961 | -1.371639645 | 3.00E-17 | 2.97E-16 |
| BCL2L2-PABPN1 | 0.061335267 | 0.140277977 | 1.193499788 | 1.07E-08 | 3.22E-08 |
| VPS72 | 3.990909557 | 8.530626861 | 1.095934175 | 7.70E-24 | 2.93E-22 |
| FANCB | 0.027735527 | 0.145883181 | 2.395006502 | 1.89E-20 | 3.24E-19 |
| MAST2 | 1.744785204 | 3.689576039 | 1.080405608 | 4.84E-22 | 1.15E-20 |
| ABAT | 66.40347352 | 27.16873111 | -1.289311523 | 2.11E-20 | 3.57E-19 |
| PHYHIPL | 0.6045723 | 2.418225077 | 1.999961745 | 0.000422874 | 0.000716987 |
| ADAM22 | 0.037863547 | 0.129362736 | 1.772540627 | 0.000140791 | 0.000254169 |
| ZNF530 | 0.18104736 | 0.428419546 | 1.242657161 | 1.06E-11 | 4.66E-11 |
| GJC1 | 0.100647163 | 0.539353215 | 2.421923881 | 4.30E-26 | 3.74E-24 |
| PSMD4 | 34.27117017 | 83.77766437 | 1.289570211 | 2.51E-26 | 2.42E-24 |
| ITGA6 | 3.611611978 | 10.1110736 | 1.485221384 | 7.04E-19 | 9.02E-18 |
| TMEM88B | 0.005852813 | 0.164432375 | 4.812220476 | 5.61E-06 | 1.21E-05 |
| NEU1 | 10.78482103 | 25.59227337 | 1.246706072 | 6.26E-23 | 1.86E-21 |
| LHX4 | 0.025255449 | 0.160379458 | 2.666822766 | 2.91E-21 | 5.80E-20 |
| HMGA1 | 10.20472697 | 37.41772698 | 1.874484338 | 3.92E-17 | 3.78E-16 |
| TBC1D31 | 0.259007487 | 0.710578683 | 1.456000607 | 1.38E-23 | 4.90E-22 |
| SLC7A2 | 57.55186547 | 22.13191841 | -1.378734182 | 1.92E-20 | 3.27E-19 |
| FAM72B | 0.030219971 | 0.156334703 | 2.371063885 | 2.63E-17 | 2.64E-16 |
| XDH | 21.28995406 | 9.71614586 | -1.131716783 | 3.11E-15 | 2.20E-14 |
| CLEC4D | 0.075488052 | 0.022288571 | -1.759944226 | 5.68E-12 | 2.59E-11 |
| CELA1 | 0.027583914 | 0.010647357 | -1.37333178 | 2.11E-05 | 4.22E-05 |
| B3GNTL1 | 0.318883271 | 0.89676228 | 1.491697182 | 1.75E-19 | 2.46E-18 |
| RIIAD1 | 0.005574623 | 0.02890385 | 2.374315582 | 2.21E-05 | 4.41E-05 |
| METTL12 | 0.320227373 | 0.70758785 | 1.14381264 | 1.01E-11 | 4.48E-11 |
| MMRN1 | 1.91401907 | 0.506170883 | -1.91890878 | 7.26E-20 | 1.10E-18 |
| TBCE | 5.906309754 | 13.07867174 | 1.146887104 | 1.47E-24 | 7.10E-23 |
| DEFB132 | 0.133312025 | 0.830077375 | 2.638438906 | 0.039334083 | 0.049668469 |
| TRIM74 | 0.039726004 | 0.156666839 | 1.979544244 | 4.09E-06 | 8.92E-06 |
| HIST1H2AJ | 0.010429951 | 0.083633525 | 3.003348969 | 7.46E-05 | 0.000139424 |
| TMCO3 | 1.609654903 | 3.954699163 | 1.296816533 | 3.88E-15 | 2.72E-14 |
| MEPE | 0.006535642 | 0.034081429 | 2.382584934 | 0.015306667 | 0.020600443 |
| PAGE2 | 0.051395493 | 7.707245144 | 7.228429625 | 0.012528329 | 0.017063445 |
| DRAXIN | 0.014157505 | 0.033609432 | 1.24729906 | 4.64E-06 | 1.01E-05 |
| CD300E | 0.735465024 | 0.234279612 | -1.650425324 | 3.23E-17 | 3.18E-16 |
| ANKRD65 | 0.527720755 | 2.963884924 | 2.489642803 | 6.82E-05 | 0.000128003 |
| TRIM54 | 0.029578239 | 0.635188061 | 4.424575752 | 6.29E-08 | 1.71E-07 |
| TMEM27 | 15.09216704 | 3.029139357 | -2.316820116 | 6.76E-25 | 3.68E-23 |
| ZNF99 | 0.004199734 | 0.087366831 | 4.378715753 | 1.17E-06 | 2.72E-06 |
| FAM180B | 0.01054211 | 0.038126984 | 1.854648705 | 0.001598723 | 0.002486094 |
| TRIM40 | 0.081336459 | 0.174995866 | 1.105346759 | 0.007526442 | 0.01058646 |
| BCAP31 | 55.23404346 | 120.4427459 | 1.124717855 | 2.85E-23 | 9.20E-22 |
| OR51S1 | 0.000489444 | 0.000139518 | -1.81068822 | 0.001617098 | 0.002512615 |
| MPDZ | 8.784702952 | 4.236836936 | -1.052005899 | 1.38E-16 | 1.21E-15 |
| ZNF580 | 1.781720671 | 3.827159041 | 1.103002679 | 2.06E-16 | 1.75E-15 |
| TOP2A | 0.43726838 | 7.213358387 | 4.044080173 | 3.57E-27 | 5.55E-25 |
| RAVER2 | 0.493101586 | 1.03989697 | 1.076483799 | 0.010044243 | 0.013885581 |
| LRRC4C | 0.207903305 | 0.057887614 | -1.844586105 | 1.15E-23 | 4.18E-22 |
| GLS | 1.751385761 | 3.839334653 | 1.13235943 | 2.55E-07 | 6.45E-07 |
| GLYATL1 | 50.42353288 | 14.27553971 | -1.820551913 | 5.54E-27 | 7.48E-25 |
| KLHL23 | 1.350847296 | 2.756739654 | 1.029098429 | 1.62E-10 | 6.10E-10 |
| KRTAP19-8 | 0.016839255 | 0.004829415 | -1.801908073 | 0.000116605 | 0.000212777 |
| GABRR2 | 0.022535335 | 0.046141166 | 1.033865535 | 0.003798831 | 0.005588333 |
| F8 | 3.114031789 | 1.307687657 | -1.25176568 | 1.55E-22 | 4.22E-21 |
| CPEB3 | 6.153211483 | 1.341302654 | -2.197704772 | 2.19E-27 | 3.82E-25 |
| AADAT | 15.67285969 | 2.493383093 | -2.652091974 | 4.20E-28 | 1.33E-25 |
| PGC | 0.04363745 | 75.69284411 | 10.76037441 | 3.62E-11 | 1.48E-10 |
| ZNF750 | 0.016212364 | 0.040167462 | 1.30893284 | 3.60E-06 | 7.92E-06 |
| CHRFAM7A | 0.002517918 | 0.008543808 | 1.76264797 | 0.015780532 | 0.021192516 |
| C17orf99 | 0.003466403 | 0.0236653 | 2.771262019 | 5.58E-06 | 1.20E-05 |
| KREMEN2 | 0.029537374 | 0.172013659 | 2.541909664 | 5.41E-10 | 1.91E-09 |
| TGM4 | 0.003704814 | 0.036528341 | 3.301543188 | 1.35E-05 | 2.76E-05 |
| IBSP | 0.00317588 | 0.67557473 | 7.732815035 | 6.02E-10 | 2.11E-09 |
| LRRC37A | 0.028532466 | 0.066605202 | 1.223030422 | 4.77E-06 | 1.03E-05 |
| DAPK2 | 0.292501011 | 0.943643969 | 1.689801031 | 3.76E-13 | 2.02E-12 |
| DCUN1D3 | 3.051685643 | 1.392046805 | -1.132398636 | 1.41E-26 | 1.50E-24 |
| C18orf54 | 0.084160272 | 0.238308457 | 1.501618872 | 4.24E-17 | 4.08E-16 |
| HIC2 | 0.413658797 | 0.999009691 | 1.27205741 | 1.05E-09 | 3.55E-09 |
| LILRB1 | 1.303788427 | 0.602077622 | -1.114688374 | 1.80E-14 | 1.16E-13 |
| TBX19 | 0.249081999 | 0.517274088 | 1.054308162 | 1.41E-14 | 9.18E-14 |
| SKAP1 | 8.308930168 | 2.876135036 | -1.53053132 | 4.24E-21 | 8.21E-20 |
| NDRG2 | 52.10184735 | 22.27450307 | -1.22594128 | 5.70E-24 | 2.26E-22 |
| MGAT5B | 0.016925769 | 0.063617953 | 1.910212537 | 0.02383762 | 0.031099849 |
| MSH5 | 0.069208879 | 0.359110573 | 2.375399082 | 1.56E-20 | 2.70E-19 |
| SMPD3 | 1.080752308 | 0.29648783 | -1.865991124 | 1.98E-23 | 6.65E-22 |
| MMP12 | 0.070652143 | 1.646746768 | 4.542741589 | 3.83E-09 | 1.21E-08 |
| SNCG | 1.834522022 | 22.21796368 | 3.598250469 | 8.40E-10 | 2.88E-09 |
| PI16 | 0.348169084 | 0.092490755 | -1.912407033 | 3.37E-16 | 2.77E-15 |
| GABRD | 0.085486643 | 2.076354102 | 4.602209673 | 1.50E-29 | 2.69E-26 |
| SH2D1B | 0.303869129 | 0.117723878 | -1.368043137 | 4.74E-15 | 3.28E-14 |
| RFC4 | 1.545572347 | 5.167154863 | 1.741228936 | 1.68E-25 | 1.16E-23 |
| C10orf35 | 1.515097439 | 3.153117496 | 1.057368351 | 1.94E-06 | 4.40E-06 |
| SLX1A | 0.004096452 | 0.019198958 | 2.228581053 | 1.11E-05 | 2.30E-05 |
| CPNE7 | 0.147201577 | 1.079916443 | 2.875054656 | 2.64E-09 | 8.47E-09 |
| LCAT | 81.64725156 | 16.90719883 | -2.271766667 | 7.18E-28 | 1.75E-25 |
| TEX45 | 0.020055705 | 0.102640897 | 2.355521065 | 5.88E-07 | 1.42E-06 |
| INSC | 0.040838992 | 0.166043548 | 2.023542505 | 7.57E-06 | 1.60E-05 |
| STAB2 | 3.676411885 | 0.127489649 | -4.849846388 | 9.32E-30 | 2.52E-26 |
| TGFBR3 | 5.476798694 | 2.598481941 | -1.075663821 | 2.77E-17 | 2.77E-16 |
| CERS1 | 0.010816494 | 0.24026849 | 4.473342602 | 1.41E-07 | 3.69E-07 |
| OR8J1 | 0.001014486 | 0.000104378 | -3.280854689 | 0.000544751 | 0.000910532 |
| ZFP1 | 5.474576413 | 1.746619403 | -1.648182071 | 3.26E-24 | 1.40E-22 |
| UNC5A | 0.026680653 | 0.214313777 | 3.005858693 | 7.81E-10 | 2.69E-09 |
| HOXC13 | 0.001123706 | 0.069353602 | 5.947634269 | 0.002838155 | 0.004254727 |
| DTL | 0.224422883 | 2.294772091 | 3.354059188 | 6.12E-26 | 4.88E-24 |
| AIF1L | 0.766361526 | 2.321460399 | 1.598935632 | 4.87E-07 | 1.19E-06 |
| LRRC55 | 0.385088611 | 0.053455153 | -2.848789526 | 3.79E-24 | 1.58E-22 |
| KIF15 | 0.061321902 | 0.695323964 | 3.503210955 | 1.75E-25 | 1.21E-23 |
| FAM133A | 0.023676042 | 1.055060552 | 5.477754098 | 0.033983548 | 0.043316077 |
| CDK1 | 0.328565026 | 3.896627727 | 3.56797528 | 1.99E-27 | 3.56E-25 |
| IQCC | 0.303254601 | 0.872022478 | 1.523835791 | 6.19E-22 | 1.44E-20 |
| ITGB1BP2 | 0.159468651 | 0.42649476 | 1.419255177 | 5.56E-09 | 1.72E-08 |
| C8orf4 | 69.43785826 | 19.66536397 | -1.820065562 | 1.55E-23 | 5.42E-22 |
| GPR150 | 0.055731106 | 0.215432934 | 1.950684136 | 0.000821744 | 0.001338991 |
| C10orf55 | 0.011120996 | 0.04384837 | 1.979237262 | 3.07E-09 | 9.80E-09 |
| TCF21 | 0.62582319 | 0.185642736 | -1.753226254 | 2.62E-25 | 1.67E-23 |
| GALNT10 | 0.925340404 | 1.927303446 | 1.058527645 | 7.69E-19 | 9.75E-18 |
| RPL10L | 0.011447138 | 0.410061271 | 5.162780649 | 0.000208507 | 0.000367642 |
| MT1X | 359.2217729 | 86.03659549 | -2.061852454 | 4.43E-22 | 1.06E-20 |
| ACAA2 | 119.9919283 | 52.79498714 | -1.184464503 | 7.29E-23 | 2.12E-21 |
| GNAT1 | 0.096007518 | 0.271481544 | 1.499634828 | 5.10E-06 | 1.10E-05 |
| PDLIM7 | 1.982668114 | 4.846442941 | 1.289483067 | 3.16E-16 | 2.60E-15 |
| NOCT | 6.978998464 | 2.214192322 | -1.656239476 | 1.49E-16 | 1.30E-15 |
| UNC119B | 1.204121699 | 2.756789027 | 1.195007654 | 2.34E-15 | 1.68E-14 |
| GPR37L1 | 0.035220503 | 0.105764454 | 1.586367414 | 4.33E-06 | 9.40E-06 |
| PFN4 | 0.187853119 | 0.562905315 | 1.583287196 | 7.45E-10 | 2.57E-09 |
| TAC4 | 0.042148805 | 0.110190642 | 1.386438064 | 8.49E-07 | 2.01E-06 |
| GJA5 | 0.826950543 | 2.440808183 | 1.561485966 | 9.75E-11 | 3.76E-10 |
| ZNF732 | 0.011198136 | 0.048222455 | 2.106446461 | 6.74E-08 | 1.83E-07 |
| ADIRF | 0.000714344 | 3.66E-05 | -4.284801308 | 0.001603079 | 0.002492574 |
| SLC50A1 | 13.62707676 | 34.52420947 | 1.341132267 | 4.04E-20 | 6.45E-19 |
| PTGS2 | 0.986960715 | 0.175568899 | -2.49095536 | 2.25E-24 | 1.01E-22 |
| ZNF519 | 0.037812261 | 0.078938714 | 1.061878922 | 8.85E-08 | 2.37E-07 |
| MAFA | 0.010906101 | 1.17408353 | 6.750255849 | 2.36E-12 | 1.13E-11 |
| ZNF83 | 0.622041197 | 1.691985194 | 1.443634907 | 0.009607022 | 0.013320208 |
| EPHX2 | 57.06322752 | 24.14110725 | -1.241069501 | 1.41E-21 | 3.01E-20 |
| MROH1 | 1.498772201 | 3.764358475 | 1.328622896 | 3.54E-19 | 4.78E-18 |
| PAFAH1B3 | 3.204417487 | 12.62843004 | 1.978541268 | 3.68E-14 | 2.27E-13 |
| UHRF1 | 0.116899893 | 1.209970551 | 3.371626421 | 3.21E-25 | 1.94E-23 |
| MYO18B | 0.002995721 | 0.391284115 | 7.029169363 | 1.53E-15 | 1.14E-14 |
| DCLK3 | 0.016855517 | 0.047951283 | 1.508348548 | 6.58E-08 | 1.79E-07 |
| SEPT3 | 0.03103425 | 0.284193323 | 3.194939468 | 2.28E-15 | 1.65E-14 |
| GLOD5 | 3.861839205 | 1.435810186 | -1.427423059 | 7.82E-21 | 1.44E-19 |
| DLGAP2 | 0.048674273 | 0.017549247 | -1.471750288 | 1.12E-19 | 1.64E-18 |
| ZNF74 | 0.618080858 | 1.236338017 | 1.000205742 | 4.22E-18 | 4.80E-17 |
| SLC34A3 | 0.006667204 | 0.073280977 | 3.458284909 | 3.01E-13 | 1.64E-12 |
| OR13F1 | 0.001863843 | 0.000692589 | -1.428208384 | 0.001379139 | 0.002168652 |
| NIPAL1 | 3.481976428 | 1.123377274 | -1.632063915 | 7.68E-21 | 1.42E-19 |
| PLK4 | 0.130662631 | 0.617433184 | 2.240436426 | 1.47E-25 | 1.03E-23 |
| ARIH2OS | 0.371938419 | 0.804037395 | 1.112198823 | 7.76E-15 | 5.22E-14 |
| ZNF578 | 0.008957179 | 0.02315871 | 1.37043845 | 0.004352785 | 0.00634029 |
| FERD3L | 0.001075902 | 0.000342659 | -1.650700703 | 0.003140103 | 0.004669636 |
| PRRT3 | 0.206682593 | 0.46522093 | 1.170499118 | 1.29E-14 | 8.48E-14 |
| DUOXA2 | 0.155555292 | 3.663652594 | 4.557783328 | 0.010351598 | 0.014290165 |
| FLAD1 | 6.34230037 | 13.06535075 | 1.042667746 | 9.76E-24 | 3.59E-22 |
| TMEM190 | 0.005418956 | 0.04277803 | 2.980783166 | 2.47E-05 | 4.89E-05 |
| CHAF1A | 1.426636977 | 3.964813251 | 1.474634641 | 3.98E-23 | 1.24E-21 |
| SKA3 | 0.113127101 | 1.418328984 | 3.64817571 | 1.06E-26 | 1.18E-24 |
| TNFRSF9 | 0.103659504 | 0.440819175 | 2.088334581 | 0.013586552 | 0.018417669 |
| CENPE | 0.057965871 | 0.559972345 | 3.272079952 | 3.43E-27 | 5.39E-25 |
| ANTXR2 | 6.395150234 | 2.793472391 | -1.194918689 | 2.41E-24 | 1.07E-22 |
| PAQR6 | 0.323290509 | 0.993532377 | 1.61973583 | 2.35E-11 | 9.86E-11 |
| SLC52A2 | 3.66004248 | 9.923710975 | 1.439019325 | 1.20E-17 | 1.27E-16 |
| FAM13A | 4.76552914 | 1.344431489 | -1.825640174 | 1.94E-22 | 5.08E-21 |
| COL9A2 | 0.293849209 | 1.067414843 | 1.860973056 | 0.008798956 | 0.012266501 |
| CHRNB2 | 0.007800513 | 0.039593058 | 2.343606582 | 9.47E-08 | 2.52E-07 |
| KCNJ10 | 0.550532803 | 0.263942615 | -1.060604225 | 1.18E-14 | 7.79E-14 |
| UBE2T | 0.894618768 | 8.385199072 | 3.228500105 | 4.47E-28 | 1.38E-25 |
| CENPI | 0.051816229 | 0.48895961 | 3.238239361 | 6.77E-27 | 8.70E-25 |
| FAM78B | 0.207128974 | 0.514930406 | 1.313848084 | 3.10E-09 | 9.87E-09 |
| HELLS | 0.131638593 | 0.764476504 | 2.537889645 | 1.87E-23 | 6.35E-22 |
| PRSS2 | 0.007000058 | 16.58794379 | 11.21048051 | 0.000608705 | 0.001009884 |
| UGT1A1 | 35.33912155 | 16.84280794 | -1.069133502 | 7.99E-13 | 4.11E-12 |
| LINC00282 | 0.021923348 | 0.052718357 | 1.265837268 | 0.000246298 | 0.000430745 |
| GRAMD1A | 2.772964048 | 7.843783245 | 1.500120757 | 1.17E-15 | 8.83E-15 |
| SFN | 1.698044715 | 23.24391966 | 3.774907018 | 8.07E-14 | 4.74E-13 |
| CFAP157 | 0.093216941 | 0.195113555 | 1.065649931 | 5.95E-16 | 4.69E-15 |
| AC025594.3 | 0.035924607 | 0.292612681 | 3.025948005 | 1.76E-20 | 3.03E-19 |
| GRPR | 0.031543658 | 0.566627853 | 4.166979644 | 3.47E-07 | 8.63E-07 |
| TMEM79 | 0.983650396 | 2.082242063 | 1.081920236 | 4.29E-21 | 8.28E-20 |
| GRIN1 | 0.003689135 | 0.028001534 | 2.924151527 | 9.38E-06 | 1.96E-05 |
| TNFRSF4 | 0.411227235 | 2.145972094 | 2.383623596 | 1.49E-24 | 7.13E-23 |
| MBNL2 | 22.09930402 | 9.993841319 | -1.144889719 | 1.44E-15 | 1.08E-14 |
| HMGCL | 50.0384831 | 23.97522278 | -1.061493835 | 4.69E-24 | 1.88E-22 |
| UNC13A | 0.008079895 | 0.094851348 | 3.553259813 | 1.67E-11 | 7.19E-11 |
| PVRIG | 0.013331029 | 0.030890002 | 1.212351809 | 3.84E-05 | 7.45E-05 |
| IDO2 | 1.304430532 | 0.375628357 | -1.796042232 | 2.06E-17 | 2.11E-16 |
| GAPDH | 285.6679209 | 794.825559 | 1.476299228 | 8.76E-20 | 1.30E-18 |
| APOA2 | 4784.092688 | 10895.19635 | 1.187374951 | 1.62E-10 | 6.09E-10 |
| LGI2 | 0.086892455 | 0.242680453 | 1.481755086 | 1.14E-06 | 2.66E-06 |
| FAM212A | 0.532808683 | 1.498600864 | 1.491926689 | 1.69E-17 | 1.76E-16 |
| ZHX1-C8orf76 | 0.287633004 | 0.830097273 | 1.529051179 | 7.84E-15 | 5.27E-14 |
| PRAM1 | 0.825620066 | 0.286187808 | -1.528515819 | 1.77E-24 | 8.30E-23 |
| BCAS1 | 0.067896415 | 0.408246608 | 2.588033597 | 0.004067268 | 0.005954507 |
| GNAO1 | 3.961454518 | 0.868433119 | -2.189543586 | 2.02E-21 | 4.17E-20 |
| CENPA | 0.088967603 | 1.619079212 | 4.185749669 | 5.73E-27 | 7.62E-25 |
| GRIN2B | 0.126304993 | 0.02829114 | -2.158489438 | 8.08E-19 | 1.02E-17 |
| PIP5KL1 | 0.036832931 | 0.103018765 | 1.483839039 | 1.76E-06 | 4.02E-06 |
| C1orf146 | 0.012013678 | 0.028202813 | 1.23116117 | 0.013987448 | 0.018932275 |
| BDKRB2 | 0.343974198 | 0.928399543 | 1.432445462 | 0.000333161 | 0.000572616 |
| PABPC4L | 0.033640952 | 0.098944019 | 1.556393971 | 1.16E-14 | 7.65E-14 |
| FBXO43 | 0.020957393 | 0.369270969 | 4.139148687 | 1.59E-27 | 3.02E-25 |
| SLC46A3 | 25.69773017 | 11.05661922 | -1.216730614 | 2.78E-17 | 2.78E-16 |
| SPATA45 | 0.071975504 | 0.243567158 | 1.758741719 | 3.92E-08 | 1.09E-07 |
| MAP1A | 0.099286812 | 0.369068228 | 1.894213547 | 3.57E-10 | 1.29E-09 |
| MBD3L2B | 0.00325868 | 0.000911447 | -1.838056298 | 0.0039863 | 0.005846974 |
| MOGAT2 | 27.09269739 | 6.471518556 | -2.065727849 | 3.03E-23 | 9.67E-22 |
| IRX6 | 0.001890402 | 0.115229457 | 5.929672319 | 7.12E-06 | 1.51E-05 |
| CENPF | 0.128366966 | 2.056358697 | 4.001746053 | 4.20E-28 | 1.33E-25 |
| OR7A10 | 0.001260918 | 8.73E-05 | -3.851808321 | 0.001012749 | 0.00162789 |
| HIST1H2BG | 0.31530877 | 1.510395067 | 2.260088753 | 4.27E-11 | 1.73E-10 |
| STXBP4 | 0.17986184 | 0.396533025 | 1.140551892 | 1.56E-15 | 1.16E-14 |
| HOXC8 | 0.005523638 | 0.090936074 | 4.041161989 | 0.016819308 | 0.022490313 |
| ADGRE1 | 0.786142006 | 0.196911022 | -1.997246075 | 1.73E-21 | 3.63E-20 |
| EDNRB | 10.37087541 | 4.064268549 | -1.35147004 | 4.90E-22 | 1.16E-20 |
| SYCP2L | 0.017145224 | 0.047873159 | 1.481410251 | 0.000779111 | 0.001274864 |
| CPT1B | 0.169011644 | 0.385405292 | 1.189253735 | 7.39E-10 | 2.56E-09 |
| OR2W3 | 0.027768881 | 0.012838009 | -1.113047547 | 1.54E-05 | 3.13E-05 |
| TRIM65 | 1.803230959 | 3.730039545 | 1.048606736 | 2.74E-20 | 4.56E-19 |
| KIAA1524 | 0.148584473 | 0.719962805 | 2.276639017 | 3.13E-22 | 7.80E-21 |
| PTH1R | 9.777457554 | 1.125479573 | -3.118919496 | 2.41E-29 | 3.13E-26 |
| CENPJ | 0.367994598 | 0.900297955 | 1.290717956 | 6.60E-21 | 1.24E-19 |
| PLA2G6 | 0.895580644 | 2.230054722 | 1.316183859 | 1.21E-15 | 9.11E-15 |
| RFX8 | 0.023798431 | 0.192464542 | 3.015654316 | 1.52E-23 | 5.32E-22 |
| ZNF707 | 0.682070332 | 1.368007155 | 1.004083359 | 1.42E-20 | 2.47E-19 |
| HES7 | 0.002592589 | 0.016057301 | 2.630763952 | 0.006954171 | 0.009826794 |
| CCDC74B | 0.016434407 | 0.053578409 | 1.704932363 | 0.028591066 | 0.036908397 |
| DNASE1L3 | 32.69447003 | 5.012812101 | -2.705354575 | 2.31E-27 | 3.96E-25 |
| HIST1H3J | 0.0025467 | 0.056647523 | 4.475311855 | 5.68E-12 | 2.59E-11 |
| UPK1B | 0.134892552 | 0.055889024 | -1.2711738 | 5.77E-11 | 2.30E-10 |
| SLC12A5 | 0.047462052 | 0.170256279 | 1.842861624 | 1.47E-06 | 3.38E-06 |
| EHD3 | 4.954912603 | 1.162021453 | -2.092222908 | 7.73E-25 | 4.15E-23 |
| TPM2 | 2.990750792 | 11.71752057 | 1.970087721 | 1.16E-17 | 1.23E-16 |
| AMDHD1 | 51.85954356 | 23.65187007 | -1.132655251 | 4.09E-17 | 3.94E-16 |
| SF3B4 | 15.10752597 | 35.5326725 | 1.233878779 | 3.30E-24 | 1.41E-22 |
| CCNA2 | 0.412357407 | 4.896754353 | 3.569858599 | 3.75E-26 | 3.37E-24 |
| NMUR1 | 0.419765595 | 0.207445018 | -1.016854912 | 4.81E-19 | 6.31E-18 |
