# Supplementary material for: Construction of circRNA-Based ceRNA Network to Reveal the Role of circRNAs in the Progression and Prognosis of Hepatocellular Carcinoma
Source: Front Genet. 2021 Feb 26;12:626764. doi: 10.3389/fgene.2021.626764 (PMC7953168; doi:10.3389/fgene.2021.626764)
Supplement: Supplementary Table 3 — 46 hub miRNAs in blue module. [file Table_3.docx]

**Table S3. 46 hub miRNAs in blue module**

| miRNA | R | P.value |
| --- | --- | --- |
| hsa-miR-142-5p | 0.813905504 | 6.40E-47 |
| hsa-miR-7850-5p | 0.312758801 | 9.50E-06 |
| hsa-let-7c-3p | 0.741518906 | 6.09E-35 |
| hsa-miR-6502-5p | 0.68167305 | 1.02E-27 |
| hsa-miR-3614-5p | 0.547438163 | 1.75E-16 |
| hsa-miR-4800-3p | 0.560450155 | 2.35E-17 |
| hsa-miR-130a-3p | 0.853166155 | 7.03E-56 |
| hsa-miR-490-3p | 0.779091489 | 1.36E-40 |
| hsa-let-7c-5p | 0.786862206 | 6.69E-42 |
| hsa-miR-4536-3p | 0.596148064 | 5.88E-20 |
| hsa-miR-130a-5p | 0.641736094 | 8.67E-24 |
| hsa-miR-1294 | 0.421849887 | 9.96E-10 |
| hsa-miR-122-3p | 0.728562531 | 3.24E-33 |
| hsa-miR-335-5p | 0.682405218 | 8.50E-28 |
| hsa-miR-4683 | 0.574090758 | 2.59E-18 |
| hsa-miR-6503-5p | 0.756774998 | 4.12E-37 |
| hsa-miR-4442 | 0.607451638 | 7.53E-21 |
| hsa-miR-199a-3p | 0.89272708 | 4.82E-68 |
| hsa-miR-551a | 0.614365541 | 2.05E-21 |
| hsa-miR-144-3p | 0.622927727 | 3.94E-22 |
| hsa-miR-133b | 0.588198749 | 2.38E-19 |
| hsa-miR-33b-3p | 0.74911194 | 5.30E-36 |
| hsa-miR-424-3p | 0.87924781 | 2.01E-63 |
| hsa-miR-199b-3p | 0.892961816 | 3.96E-68 |
| hsa-miR-1248 | 0.726495132 | 5.99E-33 |
| hsa-miR-139-5p | 0.849066682 | 7.92E-55 |
| hsa-miR-4454 | 0.491820694 | 3.77E-13 |
| hsa-miR-92b-3p | 0.265364143 | 0.000191709 |
| hsa-miR-1258 | 0.802454172 | 1.06E-44 |
| hsa-miR-139-3p | 0.91885048 | 4.62E-79 |
| hsa-miR-4686 | 0.566095443 | 9.54E-18 |
| hsa-miR-4791 | 0.7146987 | 1.78E-31 |
| hsa-miR-542-5p | 0.806839196 | 1.56E-45 |
| hsa-miR-10a-3p | 0.848676332 | 9.94E-55 |
| hsa-miR-214-3p | 0.86917635 | 2.55E-60 |
| hsa-miR-503-3p | 0.727835823 | 4.02E-33 |
| hsa-miR-195-5p | 0.832317105 | 7.85E-51 |
| hsa-miR-150-3p | 0.655455143 | 4.52E-25 |
| hsa-miR-4685-3p | 0.61011895 | 4.58E-21 |
| hsa-miR-4751 | 0.311563079 | 1.03E-05 |
| hsa-miR-450a-1-3p | 0.44889996 | 5.85E-11 |
| hsa-miR-101-3p | 0.728862754 | 2.96E-33 |
| hsa-miR-511-5p | 0.796661565 | 1.24E-43 |
| hsa-miR-424-5p | 0.892708265 | 4.90E-68 |
| hsa-miR-450a-5p | 0.851068711 | 2.45E-55 |
| hsa-miR-33b-5p | 0.771187957 | 2.58E-39 |
