# Supplementary material for: Construction of circRNA-Based ceRNA Network to Reveal the Role of circRNAs in the Progression and Prognosis of Hepatocellular Carcinoma
Source: Front Genet. 2021 Feb 26;12:626764. doi: 10.3389/fgene.2021.626764 (PMC7953168; doi:10.3389/fgene.2021.626764)
Supplement: Supplementary Table 4 — 119 hub genes in yellow module. [file Table_4.docx]

**Table S4. 119 hub genes in yellow module**

| gene | R | P.value |
| --- | --- | --- |
| VNN1 | 0.269170352 | 1.80E-08 |
| ECM1 | 0.791692936 | 2.37E-92 |
| TRIB1 | 0.64254245 | 9.64E-51 |
| INS-IGF2 | 0.667195699 | 6.09E-56 |
| DUSP6 | 0.582034823 | 8.27E-40 |
| IER2 | 0.451035694 | 1.23E-22 |
| ADAMTS13 | 0.8131215 | 3.20E-101 |
| LDLR | 0.484532754 | 2.40E-26 |
| SLC38A2 | 0.603124373 | 2.35E-43 |
| TMEM150B | -0.30103768 | 2.49E-10 |
| DBH | 0.649376975 | 3.90E-52 |
| ATF3 | 0.58838342 | 7.53E-41 |
| CLEC4M | 0.818714181 | 1.00E-103 |
| ADGRG7 | 0.440741125 | 1.41E-21 |
| RCAN1 | 0.691037223 | 1.82E-61 |
| CFP | 0.793358869 | 5.29E-93 |
| CCL23 | 0.653902678 | 4.45E-53 |
| MAFF | 0.48561677 | 1.79E-26 |
| PZP | 0.534926631 | 9.33E-33 |
| COL5A3 | -0.225659085 | 2.68E-06 |
| SERTAD1 | 0.57143375 | 4.02E-38 |
| DTX1 | 0.34683543 | 1.98E-13 |
| SERPINB8 | 0.50327664 | 1.32E-28 |
| SERPINE1 | 0.566790797 | 2.11E-37 |
| TMEM45B | -0.274509652 | 9.11E-09 |
| FAM46A | 0.44957588 | 1.74E-22 |
| ID1 | 0.449160176 | 1.93E-22 |
| SOCS3 | 0.610388687 | 1.22E-44 |
| KLF10 | 0.48518018 | 2.02E-26 |
| PDE7B | 0.29284786 | 7.87E-10 |
| CLEC4G | 0.822865538 | 1.21E-105 |
| C21orf91 | 0.545000083 | 3.59E-34 |
| OIT3 | 0.816708843 | 8.10E-103 |
| CSRNP1 | 0.832384843 | 3.13E-110 |
| LY6E | 0.322793094 | 9.74E-12 |
| CCDC187 | -0.14534264 | 0.002700702 |
| ZFP36 | 0.76913861 | 4.44E-84 |
| CEACAM20 | -0.076390597 | 0.116268439 |
| PIM1 | 0.328972504 | 3.70E-12 |
| NR4A2 | 0.324536299 | 7.43E-12 |
| EGR2 | 0.536334399 | 5.95E-33 |
| ADRA2B | 0.535168477 | 8.64E-33 |
| DBNDD1 | -0.185335312 | 0.000123876 |
| NAT2 | 0.620110594 | 2.07E-46 |
| DUSP1 | 0.517029422 | 2.34E-30 |
| GDF2 | 0.850398391 | 9.19E-120 |
| CLEC1B | 0.819105653 | 6.64E-104 |
| IL18R1 | 0.462308001 | 7.69E-24 |
| SPRY2 | 0.584164399 | 3.72E-40 |
| CDC37L1 | 0.606865161 | 5.18E-44 |
| NR4A3 | 0.56864658 | 1.09E-37 |
| SLC51B | -0.12846582 | 0.008086268 |
| SLC19A3 | 0.230214902 | 1.66E-06 |
| AQP3 | 0.432050471 | 1.04E-20 |
| FCN3 | 0.792712474 | 9.48E-93 |
| TIMD4 | 0.534066843 | 1.23E-32 |
| VIPR1 | 0.804149521 | 2.25E-97 |
| KLF6 | 0.469962162 | 1.11E-24 |
| CETP | 0.540933311 | 1.35E-33 |
| CCDC71L | 0.538778739 | 2.72E-33 |
| NPM2 | -0.190604681 | 7.83E-05 |
| KBTBD11 | 0.496911606 | 8.00E-28 |
| GADD45B | 0.62695455 | 1.08E-47 |
| TMPRSS2 | 0.365575792 | 7.47E-15 |
| ANGPTL6 | 0.819506348 | 4.35E-104 |
| CLTCL1 | -0.219055905 | 5.30E-06 |
| CCL14 | 0.537722776 | 3.82E-33 |
| CD5L | 0.57336532 | 2.00E-38 |
| PLSCR4 | 0.645640398 | 2.28E-51 |
| ZC2HC1C | 0.453919031 | 6.10E-23 |
| IGFBP3 | 0.457949529 | 2.27E-23 |
| FCN2 | 0.865905877 | 4.92E-129 |
| NFIL3 | 0.550263281 | 6.25E-35 |
| RBP7 | -0.328462291 | 4.01E-12 |
| TDGF1 | -0.173472526 | 0.000332308 |
| SGMS2 | 0.511597054 | 1.18E-29 |
| ETS2 | 0.683916367 | 9.22E-60 |
| BCO2 | 0.676702259 | 4.40E-58 |
| LIFR | 0.780314867 | 4.69E-88 |
| RIPK4 | 0.478697807 | 1.14E-25 |
| CLDN15 | -0.304453501 | 1.52E-10 |
| PHLDA1 | 0.728176794 | 3.10E-71 |
| COLEC10 | 0.877611439 | 7.75E-137 |
| RND3 | 0.759434989 | 8.35E-81 |
| NKX3-1 | 0.377412248 | 8.42E-16 |
| IL1RN | 0.521179203 | 6.70E-31 |
| EGR1 | 0.65507106 | 2.53E-53 |
| SIK1 | 0.183678579 | 0.000142715 |
| ZBTB21 | 0.569493292 | 8.06E-38 |
| ACSM1 | -0.232334419 | 1.32E-06 |
| C11orf96 | 0.540192663 | 1.72E-33 |
| FRMD4B | 0.570862829 | 4.94E-38 |
| PNRC1 | 0.554061155 | 1.74E-35 |
| RASGEF1B | 0.614365307 | 2.35E-45 |
| CRHBP | 0.838876617 | 1.57E-113 |
| FOS | 0.649729233 | 3.30E-52 |
| EPHA2 | 0.64973961 | 3.28E-52 |
| MAP2K3 | 0.442600682 | 9.12E-22 |
| AGTR1 | 0.362267216 | 1.35E-14 |
| IL1RAP | 0.487775864 | 9.98E-27 |
| DUSP5 | 0.376805324 | 9.43E-16 |
| FOSB | 0.621342584 | 1.22E-46 |
| CYP26A1 | 0.377013696 | 9.07E-16 |
| SOCS2 | 0.550341956 | 6.09E-35 |
| CDHR2 | 0.248323315 | 2.22E-07 |
| NR4A1 | 0.479459063 | 9.30E-26 |
| SCGB3A1 | 0.333598862 | 1.76E-12 |
| PLIN2 | 0.428465956 | 2.32E-20 |
| CYP2C19 | 0.295461269 | 5.47E-10 |
| APOF | 0.659183616 | 3.37E-54 |
| JUN | 0.536013307 | 6.60E-33 |
| HAMP | 0.539902643 | 1.89E-33 |
| MARCO | 0.783004838 | 4.77E-89 |
| DCUN1D3 | 0.502215547 | 1.78E-28 |
| C8orf4 | 0.68592413 | 3.08E-60 |
| MBNL2 | 0.480446558 | 7.16E-26 |
| PTH1R | 0.650663181 | 2.11E-52 |
| DNASE1L3 | 0.801160663 | 3.89E-96 |
| EHD3 | 0.547595603 | 1.52E-34 |
| VNN1 | 0.269170352 | 1.80E-08 |
| ECM1 | 0.791692936 | 2.37E-92 |
| TRIB1 | 0.64254245 | 9.64E-51 |
| INS-IGF2 | 0.667195699 | 6.09E-56 |
| DUSP6 | 0.582034823 | 8.27E-40 |
| IER2 | 0.451035694 | 1.23E-22 |
| ADAMTS13 | 0.8131215 | 3.20E-101 |
| LDLR | 0.484532754 | 2.40E-26 |
| SLC38A2 | 0.603124373 | 2.35E-43 |
| TMEM150B | -0.30103768 | 2.49E-10 |
| DBH | 0.649376975 | 3.90E-52 |
| ATF3 | 0.58838342 | 7.53E-41 |
| CLEC4M | 0.818714181 | 1.00E-103 |
| ADGRG7 | 0.440741125 | 1.41E-21 |
| RCAN1 | 0.691037223 | 1.82E-61 |
| CFP | 0.793358869 | 5.29E-93 |
| CCL23 | 0.653902678 | 4.45E-53 |
| MAFF | 0.48561677 | 1.79E-26 |
| PZP | 0.534926631 | 9.33E-33 |
| COL5A3 | -0.225659085 | 2.68E-06 |
| SERTAD1 | 0.57143375 | 4.02E-38 |
| DTX1 | 0.34683543 | 1.98E-13 |
| SERPINB8 | 0.50327664 | 1.32E-28 |
| SERPINE1 | 0.566790797 | 2.11E-37 |
| TMEM45B | -0.274509652 | 9.11E-09 |
| FAM46A | 0.44957588 | 1.74E-22 |
| ID1 | 0.449160176 | 1.93E-22 |
| SOCS3 | 0.610388687 | 1.22E-44 |
| KLF10 | 0.48518018 | 2.02E-26 |
| PDE7B | 0.29284786 | 7.87E-10 |
| CLEC4G | 0.822865538 | 1.21E-105 |
| C21orf91 | 0.545000083 | 3.59E-34 |
