# Supplementary material for: Construction of circRNA-Based ceRNA Network to Reveal the Role of circRNAs in the Progression and Prognosis of Hepatocellular Carcinoma
Source: Front Genet. 2021 Feb 26;12:626764. doi: 10.3389/fgene.2021.626764 (PMC7953168; doi:10.3389/fgene.2021.626764)
Supplement: Supplementary Table 5 — 167 hub genes in brown module. [file Table_5.docx]

**Table S5. 167 hub genes in brown module**

| gene | R | P.value |
| --- | --- | --- |
| MFAP3L | 0.708332134 | 7.98E-66 |
| CYP8B1 | 0.674262607 | 1.59E-57 |
| CYP39A1 | 0.672495233 | 3.99E-57 |
| GLYAT | 0.72487592 | 2.66E-70 |
| ACSL1 | 0.756415028 | 8.11E-80 |
| ATAD3C | 0.191242557 | 7.40E-05 |
| SLC41A2 | 0.573761362 | 1.73E-38 |
| MRAP2 | -0.331271354 | 2.56E-12 |
| CYP2C8 | 0.727118352 | 6.20E-71 |
| ACAA1 | 0.642144996 | 1.16E-50 |
| C1RL | 0.649007129 | 4.65E-52 |
| FAM149A | 0.576610021 | 6.14E-39 |
| IGFALS | 0.541474507 | 1.14E-33 |
| MTHFD1 | 0.589985513 | 4.08E-41 |
| KDM8 | 0.610795219 | 1.03E-44 |
| PLIN1 | 0.270043627 | 1.61E-08 |
| AGL | 0.689529744 | 4.21E-61 |
| KLKB1 | 0.688855384 | 6.12E-61 |
| PLPP3 | 0.637742101 | 8.74E-50 |
| GBP7 | 0.524169628 | 2.69E-31 |
| GRAMD1C | 0.519924174 | 9.80E-31 |
| LARP1B | 0.495813267 | 1.09E-27 |
| N4BP2L1 | 0.6292647 | 3.90E-48 |
| ACACB | 0.576258741 | 6.98E-39 |
| ABCC9 | 0.629707137 | 3.21E-48 |
| PPP1R3B | 0.484923418 | 2.16E-26 |
| FAM151A | 0.195123376 | 5.23E-05 |
| GPR146 | 0.547979178 | 1.34E-34 |
| TSLP | 0.547086951 | 1.80E-34 |
| HAAO | 0.572562573 | 2.68E-38 |
| C6 | 0.763363972 | 4.12E-82 |
| TMEM56 | 0.666678265 | 7.92E-56 |
| DMGDH | 0.792166502 | 1.55E-92 |
| ALDH2 | 0.691127401 | 1.73E-61 |
| GSTZ1 | 0.676542082 | 4.79E-58 |
| PANK1 | 0.619568043 | 2.61E-46 |
| GPD1 | 0.648869914 | 4.96E-52 |
| CYP2B6 | 0.566580022 | 2.27E-37 |
| ACADS | 0.528886663 | 6.25E-32 |
| MFSD2A | 0.570345376 | 5.94E-38 |
| AVPR1A | 0.490100553 | 5.29E-27 |
| ESR1 | 0.667493588 | 5.23E-56 |
| UGT2B7 | 0.607462933 | 4.06E-44 |
| GNE | 0.765355026 | 8.77E-83 |
| ACADL | 0.654952274 | 2.68E-53 |
| DBT | 0.613299951 | 3.66E-45 |
| ARHGEF26 | 0.525900918 | 1.58E-31 |
| GHR | 0.751749172 | 2.55E-78 |
| ACSM3 | 0.587723657 | 9.69E-41 |
| RDH16 | 0.673207315 | 2.75E-57 |
| CISH | 0.35668414 | 3.64E-14 |
| CYP7A1 | 0.233666759 | 1.15E-06 |
| CD302 | 0.548904525 | 9.84E-35 |
| TMEM82 | 0.438073075 | 2.62E-21 |
| MANF | -0.478904827 | 1.08E-25 |
| HAO1 | 0.70515869 | 5.31E-65 |
| HSD17B13 | 0.674732572 | 1.24E-57 |
| UROC1 | 0.615895787 | 1.24E-45 |
| CYP3A43 | 0.437311457 | 3.12E-21 |
| ACSM5 | 0.756191632 | 9.58E-80 |
| SARDH | 0.701126962 | 5.69E-64 |
| TUBE1 | 0.692055111 | 1.03E-61 |
| GNMT | 0.38163345 | 3.78E-16 |
| SHMT1 | 0.52516227 | 1.98E-31 |
| MASP1 | 0.620794153 | 1.55E-46 |
| TDO2 | 0.515428545 | 3.78E-30 |
| THRSP | 0.451785227 | 1.02E-22 |
| SLC38A4 | 0.658171839 | 5.55E-54 |
| FOXO1 | 0.507174418 | 4.28E-29 |
| LIPG | 0.493195725 | 2.25E-27 |
| SCP2 | 0.797158162 | 1.64E-94 |
| ACSL4 | -0.328219703 | 4.17E-12 |
| ANXA10 | 0.619637109 | 2.54E-46 |
| ACADM | 0.667373423 | 5.56E-56 |
| ASCL1 | 0.209370974 | 1.38E-05 |
| C8B | 0.626935715 | 1.09E-47 |
| IYD | 0.600449914 | 6.84E-43 |
| AGXT2 | 0.528054348 | 8.10E-32 |
| CPT2 | 0.598014703 | 1.80E-42 |
| FOLH1 | 0.499651101 | 3.70E-28 |
| KCNN2 | 0.627560098 | 8.26E-48 |
| TPPP2 | 0.623478152 | 4.88E-47 |
| LPA | 0.561611781 | 1.30E-36 |
| EPB41L4B | 0.527126167 | 1.08E-31 |
| SPRYD4 | 0.597942673 | 1.85E-42 |
| MAT1A | 0.692762044 | 6.89E-62 |
| NR3C2 | 0.48882415 | 7.50E-27 |
| GLS2 | 0.560205776 | 2.12E-36 |
| EFHD1 | 0.345722286 | 2.39E-13 |
| ADGRA3 | 0.55203128 | 3.45E-35 |
| CPED1 | 0.774267591 | 7.09E-86 |
| AKR1D1 | 0.675980833 | 6.44E-58 |
| ETFDH | 0.762402652 | 8.65E-82 |
| HAO2 | 0.657529512 | 7.61E-54 |
| GCGR | 0.527333101 | 1.01E-31 |
| ACADSB | 0.596682504 | 3.03E-42 |
| SPTBN2 | 0.501882844 | 1.96E-28 |
| C8A | 0.736763302 | 9.98E-74 |
| TCAP | 0.152462107 | 0.001640854 |
| RETREG1 | 0.574323268 | 1.41E-38 |
| FAHD2A | 0.550624592 | 5.54E-35 |
| FAM198A | 0.524969202 | 2.10E-31 |
| STEAP3 | 0.632875634 | 7.85E-49 |
| RNF152 | 0.579096084 | 2.46E-39 |
| RNF125 | 0.641016158 | 1.95E-50 |
| GSTM1 | 0.384186389 | 2.32E-16 |
| PLG | 0.733183057 | 1.12E-72 |
| CBR4 | 0.593346978 | 1.12E-41 |
| CYP4V2 | 0.698622668 | 2.43E-63 |
| SLC35D1 | 0.685722866 | 3.44E-60 |
| BCKDHB | 0.687818559 | 1.09E-60 |
| ADH4 | 0.691452331 | 1.44E-61 |
| PBLD | 0.646347127 | 1.63E-51 |
| BHMT | 0.615284859 | 1.60E-45 |
| SLC10A1 | 0.661759534 | 9.41E-55 |
| GIPC2 | 0.471811703 | 6.87E-25 |
| AQP8 | 0.108762184 | 0.025117783 |
| FNIP2 | 0.420614667 | 1.32E-19 |
| AL163636.2 | 0.553952254 | 1.80E-35 |
| CYP4A22 | 0.719920777 | 6.30E-69 |
| TMEM25 | 0.445881682 | 4.21E-22 |
| SLC22A1 | 0.714090299 | 2.40E-67 |
| SLC28A1 | 0.556632582 | 7.24E-36 |
| CYP4F2 | 0.695437305 | 1.51E-62 |
| MUT | 0.631814537 | 1.26E-48 |
| SLC25A47 | 0.651159476 | 1.67E-52 |
| SRD5A1 | 0.569599329 | 7.76E-38 |
| ARID3C | 0.56035738 | 2.01E-36 |
| BBOX1 | 0.507440711 | 3.96E-29 |
| CNDP1 | 0.695728435 | 1.28E-62 |
| INHBC | 0.538899512 | 2.61E-33 |
| TTC36 | 0.706220896 | 2.82E-65 |
| ALDH6A1 | 0.766571262 | 3.38E-83 |
| SORL1 | 0.59075525 | 3.04E-41 |
| OXT | 0.145041772 | 0.00275688 |
| BDH2 | 0.458952656 | 1.77E-23 |
| DNAJC25 | 0.650881637 | 1.90E-52 |
| NR1I2 | 0.723763852 | 5.44E-70 |
| NAT1 | 0.526659629 | 1.25E-31 |
| KLHL15 | 0.463894317 | 5.17E-24 |
| GCDH | 0.553833852 | 1.88E-35 |
| CYP4A11 | 0.710255085 | 2.50E-66 |
| ADRA1A | 0.762919911 | 5.80E-82 |
| GYS2 | 0.874170018 | 1.84E-134 |
| DIRAS3 | 0.58941734 | 5.07E-41 |
| SLC39A14 | 0.438152534 | 2.57E-21 |
| F9 | 0.721709237 | 2.03E-69 |
| SRD5A2 | 0.542233738 | 8.87E-34 |
| ATP11C | 0.562459079 | 9.67E-37 |
| ALDH8A1 | 0.697494997 | 4.66E-63 |
| ABAT | 0.781938742 | 1.18E-88 |
| XDH | 0.649046132 | 4.56E-52 |
| TMEM27 | 0.547045359 | 1.83E-34 |
| MPDZ | 0.683794604 | 9.85E-60 |
| GLYATL1 | 0.878232527 | 2.84E-137 |
| CPEB3 | 0.7108366 | 1.76E-66 |
| AADAT | 0.858905647 | 1.03E-124 |
| NDRG2 | 0.614351631 | 2.36E-45 |
| LCAT | 0.639416196 | 4.07E-50 |
| TGFBR3 | 0.367777945 | 5.01E-15 |
| ZFP1 | 0.672249892 | 4.53E-57 |
| ACAA2 | 0.622192623 | 8.50E-47 |
| EPHX2 | 0.71855342 | 1.49E-68 |
| FAM13A | 0.508069599 | 3.30E-29 |
| GNAO1 | 0.556588803 | 7.35E-36 |
| SLC46A3 | 0.587857058 | 9.21E-41 |
| MOGAT2 | 0.747166675 | 7.00E-77 |
| MFAP3L | 0.708332134 | 7.98E-66 |
| CYP8B1 | 0.674262607 | 1.59E-57 |
| CYP39A1 | 0.672495233 | 3.99E-57 |
| GLYAT | 0.72487592 | 2.66E-70 |
| ACSL1 | 0.756415028 | 8.11E-80 |
| ATAD3C | 0.191242557 | 7.40E-05 |
| SLC41A2 | 0.573761362 | 1.73E-38 |
| MRAP2 | -0.331271354 | 2.56E-12 |
| CYP2C8 | 0.727118352 | 6.20E-71 |
| ACAA1 | 0.642144996 | 1.16E-50 |
| C1RL | 0.649007129 | 4.65E-52 |
| FAM149A | 0.576610021 | 6.14E-39 |
| IGFALS | 0.541474507 | 1.14E-33 |
| MTHFD1 | 0.589985513 | 4.08E-41 |
| KDM8 | 0.610795219 | 1.03E-44 |
| PLIN1 | 0.270043627 | 1.61E-08 |
| AGL | 0.689529744 | 4.21E-61 |
| KLKB1 | 0.688855384 | 6.12E-61 |
| PLPP3 | 0.637742101 | 8.74E-50 |
| GBP7 | 0.524169628 | 2.69E-31 |
| GRAMD1C | 0.519924174 | 9.80E-31 |
| LARP1B | 0.495813267 | 1.09E-27 |
| N4BP2L1 | 0.6292647 | 3.90E-48 |
| ACACB | 0.576258741 | 6.98E-39 |
| ABCC9 | 0.629707137 | 3.21E-48 |
| PPP1R3B | 0.484923418 | 2.16E-26 |
| FAM151A | 0.195123376 | 5.23E-05 |
| GPR146 | 0.547979178 | 1.34E-34 |
| TSLP | 0.547086951 | 1.80E-34 |
| HAAO | 0.572562573 | 2.68E-38 |
| C6 | 0.763363972 | 4.12E-82 |
| TMEM56 | 0.666678265 | 7.92E-56 |
| DMGDH | 0.792166502 | 1.55E-92 |
| ALDH2 | 0.691127401 | 1.73E-61 |
| GSTZ1 | 0.676542082 | 4.79E-58 |
