# Supplementary material for: Construction of circRNA-Based ceRNA Network to Reveal the Role of circRNAs in the Progression and Prognosis of Hepatocellular Carcinoma
Source: Front Genet. 2021 Feb 26;12:626764. doi: 10.3389/fgene.2021.626764 (PMC7953168; doi:10.3389/fgene.2021.626764)
Supplement: Supplementary Table 6 — Basic information about hsa_circ_0077210 via circbase database. [file Table_6.docx]

**Table S6. Basic information about hsa_circ_0077210 via circbase database**

| hsa_circ_0077210 | information |
| --- | --- |
| Position | chr6:83933478-83938650 |
| Strand | - |
| Host gene Symbol | ME1 |
| bestTranscript | NM_002395 |
| Length | 423 |
| Expression (Log _2_ FC) | -2.641235 |
| P.adjust | 0.000000478 |
